# Global, regional, and national burden of tuberculosis, 1990–2016: results from the Global Burden of Diseases, Injuries, and Risk Factors 2016 Study

**DOI:** 10.1016/S1473-3099(18)30625-X

**Published:** 2018-12

**Authors:** GBD Tuberculosis Collaborators, Hmwe Hmwe Kyu, Emilie R Maddison, Nathaniel J Henry, Jorge R Ledesma, Kirsten E Wiens, Robert Reiner, Molly H Biehl, Chloe Shields, Aaron Osgood-Zimmerman, Jennifer M Ross, Austin Carter, Tahvi D Frank, Haidong Wang, Vinay Srinivasan, Sanjay Kumar Agarwal, Fares Alahdab, Kefyalew Addis Alene, Beriwan Abdulqadir Ali, Nelson Alvis-Guzman, Jason R Andrews, Carl Abelardo T Antonio, Suleman Atique, Sachin R Atre, Ashish Awasthi, Henok Tadesse Ayele, Hamid Badali, Alaa Badawi, Aleksandra Barac, Neeraj Bedi, Masoud Behzadifar, Meysam Behzadifar, Bayu Begashaw Bekele, Saba Abraham Belay, Isabela M Bensenor, Zahid A Butt, Félix Carvalho, Kelly Cercy, Devasahayam J Christopher, Alemneh Kabeta Daba, Lalit Dandona, Rakhi Dandona, Ahmad Daryani, Feleke Mekonnen Demeke, Kebede Deribe, Samath Dhamminda Dharmaratne, David Teye Doku, Manisha Dubey, Dumessa Edessa, Ziad El-Khatib, Shymaa Enany, Eduarda Fernandes, Florian Fischer, Alberto L Garcia-Basteiro, Abadi Kahsu Gebre, Gebremedhin Berhe Gebregergs, Teklu Gebrehiwo Gebremichael, Tilayie Feto Gelano, Demeke Geremew, Philimon N Gona, Amador Goodridge, Rahul Gupta, Hassan Haghparast Bidgoli, Gessessew Bugssa Hailu, Hamid Yimam Hassen, Mohammad T. Tadesse Hedayati, Andualem Henok, Sorin Hostiuc, Mamusha Aman Hussen, Olayinka Stephen Ilesanmi, Seyed Sina Naghibi Irvani, Kathryn H Jacobsen, Sarah C Johnson, Jost B. Jonas, Amaha Kahsay, Surya Kant, Amir Kasaeian, Tesfaye Dessale Kassa, Yousef Saleh Khader, Morteza Abdullatif Khafaie, Ibrahim Khalil, Ejaz Ahmad Khan, Young-Ho Khang, Yun Jin Kim, Sonali Kochhar, Ai Koyanagi, Kristopher J Krohn, G Anil Kumar, Ayenew Molla Lakew, Cheru Tesema Leshargie, Rakesh Lodha, Erlyn Rachelle King Macarayan, Reza Majdzadeh, Francisco Rogerlândio Martins-Melo, Addisu Melese, Ziad A Memish, Walter Mendoza, Desalegn Tadese Mengistu, Getnet Mengistu, Tomislav Mestrovic, Babak Moazen, Karzan Abdulmuhsin Mohammad, Shafiu Mohammed, Ali H Mokdad, Mahmood Moosazadeh, Seyyed Meysam Mousavi, Ghulam Mustafa, Jean B Nachega, Long Hoang Nguyen, Son Hoang Nguyen, Trang Huyen Nguyen, Dina Nur Anggraini Ningrum, Yirga Legesse Nirayo, Vuong Minh Nong, Richard Ofori-Asenso, Felix Akpojene Ogbo, In-Hwan Oh, Olanrewaju Oladimeji, Andrew T Olagunju, Eyal Oren, David M. Pereira, Swayam Prakash, Mostafa Qorbani, Anwar Rafay, Rajesh Kumar Rai, Usha Ram, Salvatore Rubino, Saeid Safiri, Joshua A Salomon, Abdallah M. Samy, Benn Sartorius, Maheswar Satpathy, Seyedmojtaba Seyedmousavi, Mehdi Sharif, João Pedro Silva, Dayane Gabriele Alves Silveira, Jasvinder A Singh, Chandrashekhar T Sreeramareddy, Bach Xuan Tran, Afewerki Gebremeskel Tsadik, Kingsley Nnanna Ukwaja, Irfan Ullah, Olalekan A Uthman, Vasily Vlassov, Stein Emil Vollset, Giang Vu, Fitsum Weldegebreal, Andrea Werdecker, Ebrahim M Yimer, Naohiro Yonemoto, Marcel Yotebieng, Mohsen Naghavi, Theo Vos, Simon I Hay, Christopher J L Murray

## Abstract

**Background:**

Although a preventable and treatable disease, tuberculosis causes more than a million deaths each year. As countries work towards achieving the Sustainable Development Goal (SDG) target to end the tuberculosis epidemic by 2030, robust assessments of the levels and trends of the burden of tuberculosis are crucial to inform policy and programme decision making. We assessed the levels and trends in the fatal and non-fatal burden of tuberculosis by drug resistance and HIV status for 195 countries and territories from 1990 to 2016.

**Methods:**

We analysed 15 943 site-years of vital registration data, 1710 site-years of verbal autopsy data, 764 site-years of sample-based vital registration data, and 361 site-years of mortality surveillance data to estimate mortality due to tuberculosis using the Cause of Death Ensemble model. We analysed all available data sources, including annual case notifications, prevalence surveys, population-based tuberculin surveys, and estimated tuberculosis cause-specific mortality to generate internally consistent estimates of incidence, prevalence, and mortality using DisMod-MR 2.1, a Bayesian meta-regression tool. We assessed how the burden of tuberculosis differed from the burden predicted by the Socio-demographic Index (SDI), a composite indicator of income per capita, average years of schooling, and total fertility rate.

**Findings:**

Globally in 2016, among HIV-negative individuals, the number of incident cases of tuberculosis was 9·02 million (95% uncertainty interval [UI] 8·05–10·16) and the number of tuberculosis deaths was 1·21 million (1·16–1·27). Among HIV-positive individuals, the number of incident cases was 1·40 million (1·01–1·89) and the number of tuberculosis deaths was 0·24 million (0·16–0·31). Globally, among HIV-negative individuals the age-standardised incidence of tuberculosis decreased annually at a slower rate (–1·3% [–1·5 to −1·2]) than mortality did (–4·5% [–5·0 to −4·1]) from 2006 to 2016. Among HIV-positive individuals during the same period, the rate of change in annualised age-standardised incidence was −4·0% (–4·5 to −3·7) and mortality was −8·9% (–9·5 to −8·4). Several regions had higher rates of age-standardised incidence and mortality than expected on the basis of their SDI levels in 2016. For drug-susceptible tuberculosis, the highest observed-to-expected ratios were in southern sub-Saharan Africa (13·7 for incidence and 14·9 for mortality), and the lowest ratios were in high-income North America (0·4 for incidence) and Oceania (0·3 for mortality). For multidrug-resistant tuberculosis, eastern Europe had the highest observed-to-expected ratios (67·3 for incidence and 73·0 for mortality), and high-income North America had the lowest ratios (0·4 for incidence and 0·5 for mortality).

**Interpretation:**

If current trends in tuberculosis incidence continue, few countries are likely to meet the SDG target to end the tuberculosis epidemic by 2030. Progress needs to be accelerated by improving the quality of and access to tuberculosis diagnosis and care, by developing new tools, scaling up interventions to prevent risk factors for tuberculosis, and integrating control programmes for tuberculosis and HIV.

**Funding:**

Bill & Melinda Gates Foundation.

## Introduction

Although tuberculosis is a preventable and treatable disease, it is the cause of more than a million deaths each year.^1^, ^2^ Tuberculosis was the leading cause of death from a single infectious pathogen in 2016.^1^ The ambitious Sustainable Development Goal (SDG) target 3 aims to end the tuberculosis epidemic by 2030, and numerical milestones (eg, annual reduction in global tuberculosis incidence of 10% by 2025) have been set to achieve this target.^3^ Robust assessment, monitoring, and evaluation of progress towards this SDG target are therefore crucial to inform policy and programme decision making.

Accurately assessing the tuberculosis burden over time is difficult because of the paucity of high-quality data from many low-income and middle-income countries.^2^ The completeness of vital registration data is gradually improving, but many countries still do not have good-quality vital registration systems.^1^ Notification data can be of use as a proxy for tuberculosis incidence in countries with high-quality health and surveillance systems where under-reporting is minimal;^4^ however, in most low-income and middle-income countries, these data are prone to under-reporting and cannot be interpreted without additional information on case detection rate.^4^, ^5^ To deal with the lack of high-quality data in these countries, various methods have been used to estimate tuberculosis incidence (eg, adjusting for under-reporting in notification data by use of expert opinion case detection rates,^4^ back-calculating incidence from prevalence survey data by use of different assumptions of the average duration of disease,^4^ or using a statistical triangulation approach^2^, ^6^). For the Global Burden of Diseases, Injury, and Risk Factors Study (GBD) 2015, we used a statistical triangulation approach that modelled tuberculosis incidence, prevalence, and mortality simultaneously to generate consistent estimates for these parameters.^2^

**Research in context**

**Evidence before this study**

Tuberculosis causes more than a million deaths each year and was the leading cause of death from a single infectious pathogen in 2016. The global burden of tuberculosis has been estimated by several groups, including the WHO Global Tuberculosis Programme and the Global Burden of Diseases, Injuries, and Risk Factors Study (GBD) 2015. Nevertheless, trends in the burden of drug-resistant tuberculosis by HIV status and how the observed burdens differ from the levels expected on the basis of sociodemographic development have not been comprehensively assessed. We searched PubMed with the search terms (“tuberculosis”[MeSH] AND “drug-sensitive” OR “drug-susceptible”) OR “tuberculosis, multidrug-resistant”[MeSH] AND (“burden” OR “estimates”) AND “trend”, with no language restrictions, for publications up to June 7, 2018. We identified ten studies that provided population-based time trends for the burden of multidrug-resistant tuberculosis (incidence, prevalence, or deaths). Of these studies, the most recent period assessed was 1999–2013 in Lebanon. None of these studies assessed the trends in the burden of drug-susceptible or multidrug-resistant tuberculosis by HIV status and compared these burdens with those expected on the basis of a country's socio-demographic position.

**Added value of this study**

We found that, although HIV infection and drug-resistant tuberculosis have become the main challenges to tuberculosis control efforts, more than three-quarters of incident cases of tuberculosis and deaths due to tuberculosis in 2016 were estimated to occur in HIV-negative individuals who were susceptible to first-line tuberculosis drugs. During the past decade, the global rate of decline for incidence of both drug-susceptible and multidrug-resistant tuberculosis was slower than the corresponding rate of decline for mortality, for HIV-positive and HIV-negative individuals alike. Many countries had higher burdens of drug-susceptible or multidrug-resistant tuberculosis than expected on the basis of their level of socio-demographic development.

**Implications of all the available evidence**

If current trends in tuberculosis incidence continue, few countries will meet the Sustainable Development Goal target to end the tuberculosis epidemic by 2030. The pace of progress needs to be increased through interventions including improving the quality of and access to tuberculosis diagnosis and care, and integrating control programmes for tuberculosis and HIV.

The burden of tuberculosis varies by several factors including age, sex, location, HIV status, and drug-resistance status. Therefore, these factors should be taken into account when investigating tuberculosis trends. Additionally, the burden of disease in many countries has shifted from communicable to non-communicable diseases in line with sociodemographic development (the epidemiological transition).^7^, ^8^, ^9^ As such, comparing the observed tuberculosis burden to that expected on the basis of a country's socio-demographic level could be useful for guiding investment in research and interventions.^2^ For example, countries with a lower tuberculosis burden than expected relative to their socio-demographic development could provide insight into successful programmatic strategies, and countries with a higher burden than expected might need to investigate the reasons why. GBD 2015^2^ examined the difference between the observed and expected burden of tuberculosis but did not provide a detailed assessment by drug-resistance type and HIV status. For GBD 2016, we assessed the levels and trends in the fatal and non-fatal burden of tuberculosis by drug-resistance type and HIV status from 1990 to 2016, for 195 countries and territories. We also aimed to analyse the association between these burdens and the country or territory's Socio-demographic Index (SDI),^1^, ^10^, ^11^, ^12^ which is a composite indicator of income, education, and fertility rate.

## Methods

### Overview

GBD is a systematic, scientific effort to quantify the comparative magnitude of health loss due to diseases, injuries, and risk factors by age, sex, and location over time. The conceptual and analytical framework for GBD and detailed methods have been published elsewhere.^1^, ^11^, ^13^ We describe here the methods we used for the analysis of the burden of tuberculosis for GBD 2016.

### Selection of input data

The input data we used to model mortality due to tuberculosis among HIV-negative individuals included 15 943 site-years of vital registration data, 1710 site-years of verbal autopsy data, 764 site-years of sample-based vital registration data, and 361 site-years of mortality surveillance data. We assessed and improved the quality and comparability of data on cause of death through multiple steps,^12^ including redistribution of garbage codes to underlying causes of death using GBD algorithms and adjusting for misclassified HIV deaths (ie, deaths caused by HIV being assigned to other underlying causes of death, such as tuberculosis, because of stigma or misdiagnosis). GBD 2016^1^ also assessed the overall quality of data for each country (on the basis of completeness, garbage coding, detail of cause list, and time periods covered), and assigned a quality score from zero stars (lowest) to five stars (highest); a score of four to five is considered high quality (quality scores by country are in the appendix p 19). We removed verbal autopsy data for countries with a high prevalence of HIV (using an arbitrary cutoff value of 5% age-standardised prevalence of HIV), because verbal autopsy studies have a poor ability to distinguish deaths due to HIV from deaths due to tuberculosis among people who are HIV positive (HIV-tuberculosis deaths).^2^

Our input data for the estimation of mortality due to HIV-tuberculosis included 382 site-years of high-quality vital-registration data from countries where data on cause of death directly coded for HIV-tuberculosis and tuberculosis were available, and the number of tuberculosis cases (new and re-treatment) recorded as HIV-positive, and the number of tuberculosis cases (new and re-treatment) with an HIV test result recorded in the WHO tuberculosis register.

In GBD 2016, we included multidrug-resistant tuberculosis (without extensive drug resistance) and extensively drug-resistant tuberculosis by HIV status as new outcomes (case definitions are in the appendix p 3). Input data included the number of cases of tuberculosis that were multidrug resistant, extensively drug resistant, had a drug-sensitivity testing result for isoniazid and rifampicin, and that were multidrug resistant with a drug-sensitivity result for second-line drugs from routine surveillance and surveys reported to WHO (for data availability by country see appendix p 16); relative risks of mortality for cases of tuberculosis that were multidrug resistant compared with cases that were drug susceptible, and relative risks for cases that were extensively drug resistant compared with multidrug resistant were extracted from studies identified via a systematic review (for details of systematic review see appendix p 37); and the risk of multidrug-resistant tuberculosis associated with HIV infection extracted from a meta-analysis.^14^

Our input data for modelling non-fatal tuberculosis included annual case notification data, data from prevalence surveys of tuberculosis, data from population-based tuberculin surveys, and estimated cause-specific mortality rates of tuberculosis among individuals who were HIV positive and HIV negative. Links to data sources and code we used in analyses are in the appendix (pp 40–41).

### Fatal tuberculosis

We modelled tuberculosis mortality among people who are HIV negative using the Cause of Death Ensemble modelling (CODEm) strategy,^15^, ^16^, ^17^, ^18^ which evaluates a large number of potential models that apply different functional forms (mixed-effects models and spatiotemporal Gaussian process regression models) to mortality or cause fractions with varying combinations of predictive covariates. These covariates included alcohol (L per capita), diabetes (fasting plasma glucose in mmol/L), education (years per capita), lag-distributed income (LDI), indoor air pollution, outdoor air pollution, population density (people per km^2^), smoking prevalence, SDI, the summary exposure variable scalar (which indicates exposure to risk factors associated with tuberculosis; appendix p 9), and four new covariates added for GBD 2016 (ie, prevalence of tuberculosis, prevalence of latent tuberculosis infection, proportion of adults who are underweight, and the Healthcare Access and Quality [HAQ] Index^19^). We then selected the ensemble of CODEm models that performed best on out-of-sample predictive validity tests (appendix pp 20–23). We estimated HIV-tuberculosis mortality using a population-attributable fraction approach, like in GBD 2015^2^ (detailed methods and equations are in the appendix pp 34–36).

To split tuberculosis deaths and HIV-tuberculosis deaths by drug-resistance type, we first estimated the proportions of tuberculosis cases that were multidrug resistant for all locations and years using a spatiotemporal Gaussian process regression. Second, we estimated the proportions of tuberculosis cases that were multidrug resistant by HIV status on the basis of the risk of multidrug-resistant tuberculosis associated with HIV from a meta-analysis by Mesfin and colleagues.^14^ Third, we used the estimated proportions of cases of tuberculosis that are multidrug resistant by HIV status and the relative risk of death in multidrug-resistant cases compared with drug-susceptible cases to calculate the fraction of tuberculosis deaths among HIV-negative individuals attributable to multidrug-resistant tuberculosis, and the fraction of HIV-tuberculosis deaths attributable to multidrug-resistant tuberculosis (detailed methods and equations are in the appendix pp 23–24, 35–36). Finally, we applied the fraction of tuberculosis deaths attributable to multidrug-resistant tuberculosis to the number of tuberculosis deaths we estimated using CODEm, and the fraction of HIV-tuberculosis deaths attributable to multidrug-resistant tuberculosis to our estimated number of HIV-tuberculosis deaths, to generate the number of multidrug-resistant tuberculosis deaths by HIV status by location, year, age, and sex.

To distinguish extensively drug-resistant tuberculosis from multidrug-resistant tuberculosis, we aggregated the cases of extensively drug-resistant tuberculosis and multidrug-resistant tuberculosis (with drug-sensitivity testing for second-line drugs) up to the GBD super-region level (for analytical purposes we grouped 21 GBD regions into seven super-regions:^13^ central Europe, eastern Europe and central Asia; high-income; Latin America and Caribbean; north Africa and Middle East; south Asia; southeast Asia, east Asia, and Oceania; and sub-Saharan Africa) and calculated the proportion of cases of extensively drug-resistant tuberculosis among the cases of multidrug-resistant tuberculosis at the super-region level. We then used these proportions and the relative risk of mortality among people with extensively drug-resistant tuberculosis compared with those with multidrug-resistant tuberculosis to calculate the fraction of extensively drug-resistant tuberculosis deaths among all multidrug-resistant tuberculosis deaths at the super-region level (detailed methods and equations are in the appendix p 24). These fractions were then applied to the estimated number of multidrug-resistant tuberculosis deaths and multidrug-resistant HIV-tuberculosis deaths in countries within the super-regions to calculate the number of deaths due to extensively drug-resistant tuberculosis by HIV status by location, year, age, and sex.

We linearly extrapolated mortality for extensively drug-resistant tuberculosis back from 2016 assuming mortality was zero in 1992, 1 year before extensively drug-resistant tuberculosis was first recorded in USA surveillance data in 1993.^20^ Next, we subtracted the number of deaths due to extensively drug-resistant tuberculosis from the number of deaths due to multidrug-resistant tuberculosis to generate the number of deaths due to multidrug-resistant tuberculosis (without extensive drug resistance) by location, year, age, and sex.

### Non-fatal tuberculosis

We made several improvements to the statistical trian-gulation approach we used in GBD 2015^2^ to model non-fatal tuberculosis. First, we estimated the prevalence of latent tuberculosis infection by location, year, age, and sex using data from population-based tuberculin surveys and cohort studies that reported the risk of developing active tuberculosis disease as a function of induration size.^11^ Next, we divided the inputs on prevalence (from tuberculosis prevalence surveys in low-income and middle-income countries), incidence (notification data from countries with a rating of four or five stars and estimated incidence from countries with ratings of zero to three stars), and cause-specific mortality rate by the risk-weighted prevalence of latent tuberculosis infection to model tuberculosis among individuals at risk in each country. A detailed explanation of how we prepared each of these data sources is in the appendix (pp 6–10).

To generate initial estimates of incidence for countries with a rating of zero to three stars, we did a regression analysis using mortality-to-incidence ratios (logit transformed) from locations with a rating of four or five stars as input data, with SDI as a covariate. We calibrated the lowest end of the SDI scale with a datapoint from a community-based cohort study,^21^ which reported that 49·2% of people with untreated pulmonary tuberculosis had died at the end of a 5 year follow-up period, to predict mortality-to-incidence ratios as a function of SDI for all locations and years. We then used the predicted mortality-to-incidence ratios and estimates of cause-specific mortality to calculate the age-sex specific incidence input for modelling in DisMod-MR 2.1,^22^ the GBD Bayesian meta-regression tool. In locations where our estimated mortality-to-incidence ratios were greater than notification-based mortality-to-incidence ratios, we used the notification-based ratios to calculate the incidence input. We then generated a final incidence estimate that is consistent with prevalence data and cause-specific mortality estimates using a Bayesian meta-regression.

We used DisMod-MR 2.1 to simultaneously model age-sex specific tuberculosis incidence, prevalence, and mortality among the population who are latently infected and generate consistent trends in all parameters. We then multiplied the DisMod-MR 2.1 outputs by the prevalence of latent tuberculosis infection to get population-level estimates of incidence and prevalence. To distinguish HIV-tuberculosis from all forms of tuberculosis, we applied the proportion of cases of HIV-tuberculosis among all cases of tuberculosis (estimated from a mixed-effects regression using the adult HIV mortality rate covariate as in GBD 2015^2^) to the number of incident and prevalent cases of tuberculosis. We then applied the estimated proportion of cases of tuberculosis that are multidrug resistant to our predicted number of cases of tuberculosis, and the estimated proportion of cases of HIV-tuberculosis with multidrug-resistant tuberculosis (as described earlier for fatal tuberculosis) to our predicted number of HIV-tuberculosis cases, to generate the number of cases of multidrug-resistant tuberculosis by HIV status. To distinguish extensively drug-resistant tuberculosis from multidrug-resistant tuberculosis, we calculated the proportions of cases of extensively drug-resistant tuberculosis among the cases of multidrug-resistant tuberculosis at the super-region level and applied these proportions to multidrug-resistant tuberculosis cases.

Similar to our estimation for fatal tuberculosis with extensive drug resistance, we linearly extrapolated the prevalence and incidence of extensively drug-resistant tuberculosis back from 2016, assuming incidence and prevalence were zero in 1992 and in earlier years. Finally, we subtracted the number of cases of extensively drug-resistant tuberculosis from the number of cases of multidrug-resistant tuberculosis to generate the number of cases of multidrug-resistant tuberculosis (without extensive drug resistance) by location, year, age, and sex. We used the GBD world population age standard to calculate age-standardised rates.

### SDI

SDI, initially developed for GBD 2015^9^ and updated for GBD 2016,^1^, ^10^, ^11^ was calculated on the basis of the geometric mean of three indicators: income per capita, average years of schooling, and total fertility rates. SDI scores were scaled from 0 (lowest income, lowest average years of schooling, highest fertility) to 1 (highest income, highest average years of schooling, lowest fertility), and each location was assigned an SDI score for each year. We estimated the average association between SDI and tuberculosis incidence and mortality using a Gaussian process regression, and we then used these associations to estimate expected values at each SDI level.

### Role of the funding source

The sponsor of the study had no role in study design, data collection, data analysis, data interpretation, or writing of the report. The corresponding author had full access to all the data in the study and had final responsibility for the decision to submit for publication.

## Results

### Global burden of tuberculosis in 2016

**Figure 1:**
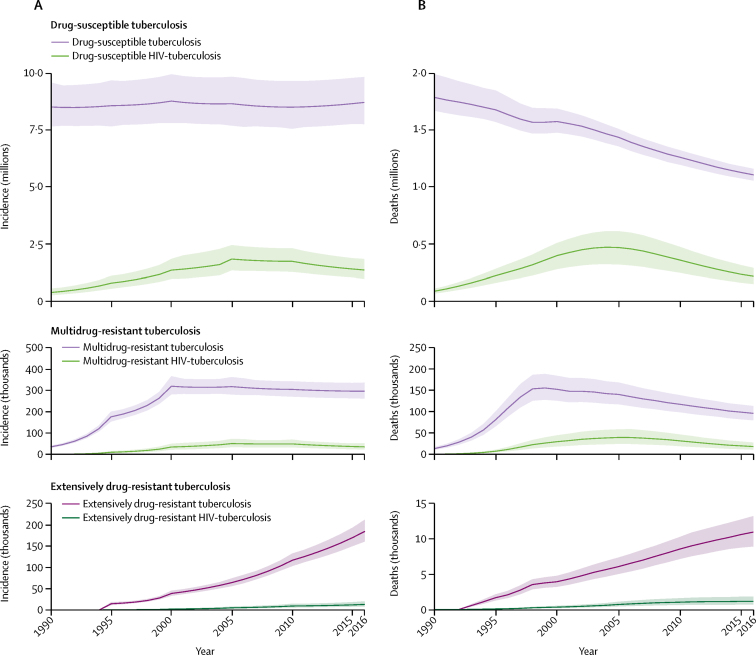
Global tuberculosis incidence (A) and mortality (B) by drug-resistance type and HIV status, 1990–2016 Dark lines are estimates and shaded areas are 95% uncertainty intervals. HIV-tuberculosis=tuberculosis in individuals with HIV/AIDS. Multidrug-resistant tuberculosis=multidrug-resistant tuberculosis without extensive drug resistance.

**Figure 2:**
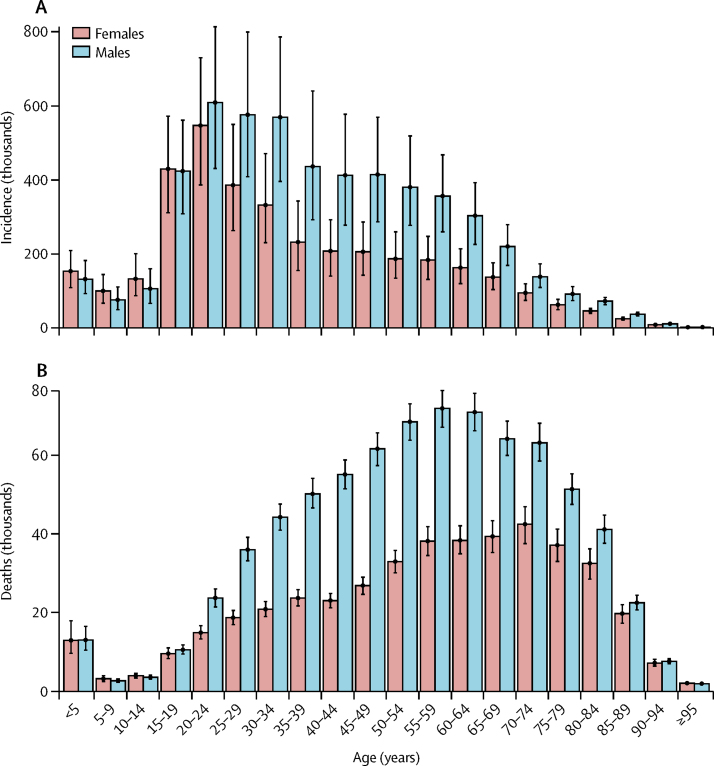
Global age-sex distribution of incident cases of tuberculosis (A) and deaths due to tuberculosis (B) among HIV-negative individuals in 2016 Error bars are 95% uncertainty intervals.

Globally in 2016, among HIV-negative individuals, we estimated 9·02 million (95% uncertainty interval [UI] 8·05–10·16; figure 1, table 1) incident cases of tuberculosis and 1·21 million (1·16–1·27) deaths due to tuberculosis (figure 1, table 2). Among HIV-positive individuals, we estimated 1·40 million (1·01–1·89; figure 1, appendix pp 75–87) incident cases of tuberculosis and 0·24 million (0·16–0·31) deaths due to tuberculosis (figure 1, appendix pp 88–100). Almost two-thirds of HIV-negative tuberculosis incident cases (59·6% [59·2–59·7]) and deaths (63·1% [62·4–63·6]) were in males (figure 2). Most incident cases (89·5% [87·9–91·0]) and deaths (64·3% [63·6–65·0]) were in people younger than 65 years for both sexes. Among HIV-positive individuals, 53·8% (52·4–54·9) of incident cases of tuberculosis and 56·9% (56·2–57·6) of deaths due to tuberculosis were in males (appendix p 46). Most incident cases of HIV-tuberculosis (82·4% [82·2–82·5]) and deaths due to HIV-tuberculosis (73·7% [72·7–74·8]) were among people aged 20–54 years for both sexes (appendix p 46).

**Table 1:** Incident cases of tuberculosis, drug-susceptible tuberculosis, multidrug-resistant tuberculosis, and extensively drug-resistant tuberculosis in HIV-negative individuals in 2016, and annualised rates of change of age-standardised incidence during the 1990–2006 and 2006–16 for 21 Global Burden of Disease regions for both sexes combined

		**Drug-susceptible tuberculosis**			**Multidrug-resistant tuberculosis**			**Extensively drug-resistant tuberculosis**			**All HIV-negative tuberculosis**		
		Number of incident cases, 2016	Annualised rate of change of age-standardised incidence		Number of incident cases, 2016	Annualised rate of change of age-standardised incidence		Number of incident cases, 2016	Annualised rate of change of age-standardised incidence		Number of incident cases, 2016	Annualised rate of change of age-standardised incidence	
			1990–2006	2006–16		1990–2006	2006–16		1990–2006	2006–16		1990–2006	2006–16
**Global**		**8 705 207 (7 754 638 to 9 817 655)**	**−1·8 (−2·0 to–1·5)**	**−1·3 (−1·5 to–1·1)**	**295 637 (261 369 to 335 586)**	**11·5 (10·8 to 12·3)**	**−2·1 (−2·9 to–1·3)**	**18 452 (16 087 to 21 187)**	**43·9 (43·1 to 44·8)**	**7·9 (6·6 to 9·1)**	**9 019 296 (8 051 800 to 10 156 811)**	**−1·6 (−1·8 to–1·3)**	**−1·3 (−1·5 to–1·2)**
High-income		**122 022 (107 600 to 138 800)**	**−5·3 (−5·7 to–5·0)**	**−2·6 (−2·8 to–2·4)**	**1715 (1352 to 2225)**	**1·6 (0·1 to 3·0)**	**−3·2 (−5·3 to–0·6)**	**216 (170 to 280)**	**29·7 (28·3 to 31·1)**	**4·4 (2·3 to 7·0)**	**123 952 (109 362 to 140 956)**	**−5·3 (−5·6 to–5·0)**	**−2·6 (−2·8 to–2·4)**
	High-income North America	12 179 (11 023 to 13 394)	−2·5 (−3·3 to–1·7)	−2·2 (−2·6 to–1·8)	139 (124 to 155)	−3·5 (−4·4 to–2·6)	−2·2 (−2·8 to–1·5)	17 (16 to 20)	20·4 (19·6 to 21·0)	5·5 (4·8 to 6·1)	12 335 (11 163 to 13 563)	−2·5 (−3·3 to–1·7)	−2·2 (−2·6 to–1·8)
	Australasia	1396 (1194 to 1603)	−3·5 (−4·3 to–2·6)	−1·5 (−2·1 to–0·9)	27 (12 to 49)	6·5 (−1·8 to 14·5)	−0·2 (−11·0 to 11·3)	3 (2 to 6)	25·0 (18·4 to 30·6)	7·5 (−3·4 to 18·9)	1426 (1228 to 1640)	−3·4 (−4·2 to–2·5)	−1·4 (−2·0 to–0·9)
	High-income Asia Pacific	69 366 (61 928 to 77 057)	−6·4 (−6·8 to–6·1)	−3·1 (−3·5 to–2·7)	872 (627 to 1210)	2·5 (0·1 to 4·8)	−5·2 (−8·6 to–1·6)	110 (79 to 152)	36·7 (34·8 to 38·5)	2·5 (−0·9 to 6·0)	70 347 (62 801 to 78 134)	−6·4 (−6·7 to–6·1)	−3·1 (−3·5 to–2·7)
	Western Europe	30 930 (24 845 to 38 614)	−3·6 (−4·2 to–3·1)	−1·8 (−2·2 to–1·4)	512 (401 to 656)	1·0 (0·0 to 2·2)	−0·8 (−2·0 to 0·4)	64 (50 to 83)	27·1 (25·6 to 28·5)	6·9 (5·6 to 8·1)	31 506 (25 320 to 39 267)	−3·6 (−4·2 to–3·0)	−1·8 (−2·2 to–1·4)
	Southern Latin America	8152 (6651 to 9877)	−4·6 (−5·3 to–3·9)	−0·9 (−1·7 to–0·3)	165 (39 to 519)	8·3 (0·2 to 18·2)	−0·7 (−15·2 to 11·4)	21 (5 to 65)	31·6 (26·4 to 35·8)	7·0 (−7·6 to 19·1)	8338 (6857 to 10 068)	−4·4 (−5·2 to–3·8)	−0·9 (−1·6 to–0·3)
**Central Europe, eastern Europe, and central Asia**		**190 982 (167 765 to 218 563)**	**0·4 (0 to 0·8)**	**−3·3 (−3·9 to–2·7)**	**34 818 (27 860 to 42 503)**	**14·2 (11·2 to 17·5)**	**−2·3 (−5·0 to 0·2)**	**7629 (6104 to 9312)**	**55·4 (54·1 to 56·7)**	**9·0 (6·2 to 11·5)**	**233 428 (206 001 to 263 867)**	**1·3 (0·9 to 1·7)**	**−2·9 (−3·2 to–2·6)**
	Eastern Europe	102 960 (88 487 to 119 678)	1·3 (0·7 to 1·8)	−3·1 (−3·9 to–2·3)	20 668 (15 832 to 26 250)	10·9 (7·7 to 14·4)	−1·1 (−4·2 to 1·6)	4529 (3469 to 5752)	55·3 (53·9 to 56·8)	10·1 (7·1 to 12·9)	128 157 (110 861 to 146 360)	2·1 (1·5 to 2·6)	−2·5 (−3·0 to–2·1)
	Central Europe	26 337 (23 435 to 29 369)	−1·4 (−1·9 to–1·0)	−3·8 (−4·1 to–3·4)	440 (252 to 757)	7·6 (1·0 to 14·2)	−5·1 (−12·9 to 2·6)	97 (55 to 166)	37·5 (33·7 to 41·1)	6·2 (−1·6 to 13·8)	26 873 (23 937 to 29 909)	−1·3 (−1·8 to–0·9)	−3·8 (−4·1 to–3·5)
	Central Asia	61 685 (53 120 to 71 399)	−0·7 (−1·3 to–0·2)	−4·1 (−5·3 to–2·8)	13 709 (10 092 to 18 011)	31·6 (24·9 to 38·6)	−4·5 (−9·2 to 0·1)	3004 (2211 to 3947)	61·0 (58·9 to 63·0)	6·7 (2·1 to 11·4)	78 397 (69 544 to 88 584)	0·7 (0·4 to 1·0)	−3·9 (−4·2 to–3·7)
**Latin America and Caribbean**		**161 862 (140 835 to 184 477)**	**−3·2 (−3·5 to–3·0)**	**−2·3 (−2·5 to–2·1)**	**3491 (2856 to 4329)**	**10·3 (7·1 to 14·0)**	**−3·3 (−5·0 to–1·5)**	**276 (226 to 342)**	**34·5 (33·3 to 35·8)**	**6·5 (4·8 to 8·3)**	**165 629 (143 934 to 188 555)**	**−3·1 (−3·4 to–2·8)**	**−2·3 (−2·5 to–2·1)**
	Central Latin America	48 806 (41 842 to 56 204)	−2·4 (−2·7 to–2·2)	−1·8 (−2·0 to–1·5)	991 (776 to 1261)	17·5 (15·7 to 19·3)	−2·9 (−4·8 to −1·0)	78 (61 to 100)	31·8 (30·5 to 33·1)	6·9 (5·0 to 8·8)	49 875 (42 743 to 57 514)	−2·3 (−2·5 to −2·1)	−1·8 (−2·0 to −1·6)
	Andean Latin America	31 412 (27 884 to 35 278)	−6·0 (−6·3 to–5·7)	−3·8 (−4·3 to–3·4)	1417 (1007 to 2070)	6·3 (2·0 to 11·3)	−3·7 (−7·6 to–0·1)	112 (80 to 163)	43·4 (41·3 to 45·6)	6·0 (2·2 to 9·7)	32 940 (29 161 to 36 850)	−5·8 (−6·0 to–5·5)	−3·8 (−4·2 to–3·4)
	Caribbean	16 519 (13 885 to 19 541)	−3·0 (−3·7 to–2·5)	−0·9 (−1·6 to–0·2)	94 (30 to 277)	2·7 (−5·2 to 10·6)	−3·7 (−13·8 to 7·9)	7 (2 to 22)	27·9 (20·8 to 33·6)	6·1 (−4·0 to 17·7)	16 620 (13 957 to 19 656)	−3·0 (−3·7 to–2·4)	−1·0 (−1·6 to–0·2)
	Tropical Latin America	65 126 (56 191 to 74 662)	−1·8 (−2·3 to–1·3)	−2·2 (−2·4 to–2·0)	989 (832 to 1150)	22·8 (22·1 to 23·5)	−3·2 (−3·9 to–2·5)	78 (66 to 91)	32·5 (31·5 to 33·4)	6·6 (5·9 to 7·3)	66 193 (57 040 to 75 915)	−1·7 (−2·2 to–1·2)	−2·2 (−2·4 to–2·0)
**Southeast Asia, east Asia, and Oceania**		**2 381 270 (2 144 141 to 2 637 352)**	**−3·6 (−3·9 to–3·3)**	**−1·9 (−2·1 to–1·8)**	**71 140 (59 963 to 86 643)**	**5·9 (4·8 to 6·9)**	**−4·0 (−5·9 to–1·8)**	**6487 (5468 to 7900)**	**45·0 (44·1 to 46·0)**	**7·9 (6·0 to 10·1)**	**2 458 896 (2 215 080 to 2 722 674)**	**−3·4 (−3·7 to–3·2)**	**−2·0 (−2·2 to–1·8)**
	East Asia	1 207 570 (1 136 842 to 1 285 280)	−4·1 (−4·2 to–3·9)	−2·0 (−2·2 to–1·8)	50 864 (44 680 to 58 560)	4·8 (3·9 to 5·7)	−4·2 (−5·4 to–2·9)	4638 (4074 to 5339)	45·1 (44·3 to 45·9)	7·7 (6·6 to 9·0)	1 263 072 (1 188 367 to 1 344 259)	−3·8 (−4·0 to–3·6)	−2·1 (−2·3 to–1·9)
	Southeast Asia	1 161 747 (993 911 to 1 345 961)	−3·3 (−3·9 to–2·9)	−2·2 (−2·5 to–2·0)	19 831 (12 086 to 32 586)	11·8 (7·5 to 16·2)	−3·3 (−9·8 to 2·9)	1808 (1102 to 2971)	44·4 (42·0 to 47·0)	8·6 (2·1 to 14·8)	1 183 386 (1 015 931 to 1 372 615)	−3·2 (−3·8 to–2·8)	−2·3 (−2·5 to–2·0)
	Oceania	11 953 (10 254 to 13 622)	−1·3 (−3·4 to–0·7)	−2·0 (−3·2 to 1·5)	444 (84 to 1373)	22·1 (6·4 to 39·3)	−7·1 (−29·7 to 23·5)	40 (8 to 125)	48·5 (30·5 to 58·9)	4·9 (−17·8 to 35·4)	12 438 (10 886 to 14 063)	−0·9 (−1·4 to–0·6)	−2·2 (−2·6 to–1·7)
**North Africa and Middle East**		**264 890 (215 268 to 327 017)**	**−2·6 (−2·9 to–2·3)**	**−2·2 (−2·6 to–1·8)**	**7721 (4118 to 14 403)**	**15·3 (9·4 to 21·1)**	**−1·2 (−10·0 to 7·4)**	**273 (145 to 508)**	**34·5 (30·7 to 38·0)**	**7·0 (−1·8 to 15·6)**	**272 884 (221 272 to 336 385)**	**−2·4 (−2·7 to–2·1)**	**−2·1 (−2·5 to–1·8)**
**South Asia**		**3 460 801 (3 161 059 to 3 815 348)**	**−1·7 (−1·8 to–1·5)**	**−1·8 (−1·9 to–1·6)**	**134 673 (121 505 to 149 109)**	**24·9 (24·4 to 25·4)**	**−1·5 (−2·1 to–0·9)**	**3301 (2978 to 3654)**	**42·6 (41·9 to 43·2)**	**8·2 (7·6 to 8·8)**	**3 598 775 (3 290 577 to 3 966 147)**	**−1·4 (−1·6 to–1·2)**	**−1·7 (−1·9 to–1·6)**
**Sub-Saharan Africa**		**2 123 380 (1 768 596 to 2 579 273)**	**−0·9 (−1·4 to–0·4)**	**−1·9 (−2·4 to–1·4)**	**42 079 (30 717 to 57 455)**	**15·9 (13·8 to 18·0)**	**−2·2 (−5·4 to 1·1)**	**271 (198 to 371)**	**30·4 (28·4 to 32·5)**	**9·0 (5·7 to 12·2)**	**2 165 731 (1 80 1974 to 2 625 879)**	**−0·8 (−1·3 to–0·2)**	**−1·9 (−2·3 to–1·4)**
	Southern sub-Saharan Africa	378 324 (290 890 to 490 853)	0·9 (−0·1 to 1·9)	−1·0 (−2·0 to–0·2)	9732 (5716 to 15 799)	13·9 (10·1 to 17·9)	−0·5 (−4·8 to 4·0)	63 (37 to 102)	34·4 (31·2 to 37·3)	10·6 (6·4 to 15·2)	388 119 (299 438 to 504 411)	1·1 (0·0 to 2·0)	−1·0 (−2·0 to–0·2)
	Western sub-Saharan Africa	545 758 (444 535 to 672 322)	−1·6 (−2·3 to–1·0)	−2·9 (−3·4 to–2·3)	13 358 (7551 to 22 859)	17·4 (13·8 to 21·1)	−3·9 (−11·0 to 3·3)	86 (49 to 147)	30·6 (27·3 to 34·0)	7·3 (0·1 to 14·4)	559 202 (457 442 to 686 867)	−1·5 (−2·1 to–0·9)	−2·9 (−3·4 to–2·3)
	Eastern sub-Saharan Africa	809 654 (678 742 to 983 108)	−0·8 (−1·2 to–0·3)	−1·5 (−2·1 to–0·8)	16 166 (10 561 to 24 674)	21·8 (17·9 to 25·5)	−0·8 (−4·9 to 3·5)	104 (68 to 159)	29·4 (27·1 to 31·6)	10·3 (6·2 to 14·7)	825 924 (693 500 to 1 004 959)	−0·6 (−1·1 to–0·2)	−1·5 (−2·1 to–0·8)
	Central sub-Saharan Africa	389 644 (329 875 to 455 126)	−0·9 (−1·3 to–0·6)	−1·6 (−2·0 to–1·1)	2824 (1782 to 4300)	8·6 (5·5 to 11·2)	−2·1 (−6·2 to 2·1)	18 (11 to 28)	28·1 (25·3 to 30·7)	9·0 (4·9 to 13·3)	392 486 (332 258 to 458 256)	−0·9 (−1·2 to–0·6)	−1·6 (−2·0 to–1·1)

Data in parentheses are 95% uncertainty intervals. Multidrug-resistant tuberculosis=multidrug-resistant tuberculosis without extensive drug resistance.

**Table 2:** Mortality from tuberculosis for drug-susceptible tuberculosis, multidrug-resistant tuberculosis, and extensively drug-resistant tuberculosis in HIV-negative individuals in 2016, and annualised rates of change of age-standardised mortality during the periods 1990–2006 and 2006–16 for 21 Global Burden of Disease regions for both sexes

		**Drug-susceptible tuberculosis**			**Multidrug-resistant tuberculosis**			**Extensively drug-resistant tuberculosis**			**All HIV-negative tuberculosis**		
		Number of deaths, 2016	Annualised rate of change of age-standardised mortality		Number of deaths, 2016	Annualised rate of change of age-standardised mortality		Number of deaths, 2016	Annualised rate of change of age-standardised mortality		Number of deaths, 2016	Annualised rate of change of age-standardised mortality	
			1990–2006	2006–16		1990–2006	2006–16		1990–2006	2006–16		1990–2006	2006–16
**Global**		1 105 898 (1 055 638 to 1 158 544)	−3·7 (−4·3 to–3·4)	−4·4 (−4·9 to–4·1)	96 238 (79 994 to 113 348)	11·9 (10·9 to 12·7)	−5·5 (−6·5 to–4·5)	10 920 (8896 to 13 162)	43·9 (42·7 to 45·1)	3·1 (1·8 to 4·5)	1 213 057 (1 161 548 to 1 265 425)	−3·2 (−3·7 to–2·9)	−4·5 (−5·0 to–4·1)
High-income		12 759 (11 633 to 13 971)	−6·3 (−6·6 to–5·9)	−3·5 (−4·4 to–2·7)	457 (347 to 600)	−0·6 (−1·8 to 0·5)	−5·1 (−7·2 to–2·7)	152 (115 to 202)	25·9 (24·4 to 27·3)	2·3 (0·3 to 4·7)	13 367 (12 202 to 14 591)	−6·1 (−6·4 to–5·7)	−3·5 (−4·4 to–2·7)
	High-income North America	1041 (991 to 1095)	−7·0 (−7·1 to–6·7)	−2·5 (−2·9 to–2·0)	31 (26 to 38)	−8·7 (−9·1 to–8·4)	−3·2 (−3·7 to–2·6)	10 (8 to 13)	16·0 (14·8 to 17·3)	4·3 (3·7 to 4·8)	1082 (1033 to 1136)	−7·0 (−7·2 to–6·8)	−2·5 (−2·9 to–2·0)
	Australasia	83 (74 to 93)	−5·0 (−5·7 to–4·4)	−3·6 (−4·8 to–2·4)	4 (2 to 8)	4·3 (−3·9 to 12·2)	−3·4 (−13·7 to 8·1)	1 (1 to 3)	19·1 (12·1 to 24·8)	4·1 (−6·3 to 15·6)	89 (80 to 99)	−4·7 (−5·3 to–4·2)	−3·5 (−4·4 to–2·5)
	High-income Asia Pacific	7092 (6222 to 8028)	−7·1 (−7·6 to–6·3)	−4·0 (−5·5 to–2·5)	217 (156 to 304)	0·6 (−1·4 to 2·6)	−6·6 (−9·7 to–3·4)	72 (50 to 102)	31·6 (29·8 to 33·4)	0·8 (−2·2 to 4·0)	7381 (6469 to 8328)	−6·9 (−7·4 to–6·1)	−4·0 (−5·6 to–2·6)
	Western Europe	3428 (3123 to 3777)	−6·3 (−6·6 to–6·0)	−4·4 (−5·2 to–3·6)	154 (121 to 191)	−1·5 (−2·5 to–0·5)	−4·6 (−6·0 to–3·2)	51 (40 to 65)	23·7 (22·4 to 24·9)	2·9 (1·5 to 4·3)	3633 (3322 to 4005)	−6·1 (−6·4 to–5·8)	−4·3 (−5·1 to–3·5)
	Southern Latin America	1116 (955 to 1273)	−6·1 (−6·7 to–5·6)	−3·7 (−5·2 to–2·3)	50 (15 to 141)	5·7 (−2·2 to 14·9)	−5·1 (−16·6 to 5·0)	17 (5 to 46)	32·6 (27·9 to 36·2)	2·4 (−9·2 to 12·4)	1183 (1049 to 1333)	−5·8 (−6·3 to–5·3)	−3·7 (−4·9 to–2·4)
**Central Europe, eastern Europe, and central Asia**		15 636 (12 358 to 19 998)	1·0 (0·1 to 1·7)	−7·8 (−10·2 to–5·2)	6536 (5013 to 8358)	13·5 (10·8 to 16·6)	−8·2 (−10·9 to–5·5)	3780 (2873 to 4801)	54·1 (52·8 to 55·1)	2·9 (0·2 to 5·6)	25 952 (21 354 to 31 882)	3·2 (2·6 to 3·8)	−6·9 (−8·9 to–4·6)
	Eastern Europe	10 165 (7287 to 14 072)	3·1 (1·9 to 4·1)	−8·1 (−11·4 to–4·7)	4660 (3317 to 6321)	11·7 (8·8 to 15·1)	−7·4 (−11·1 to–4·0)	2695 (1893 to 3686)	55·3 (54·1 to 56·6)	3·7 (0·1 to 7·1)	17 520 (13 136 to 23 086)	5·0 (4·0 to 5·9)	−6·8 (−9·7 to–3·7)
	Central Europe	2156 (1944 to 2435)	−4·2 (−4·7 to–3·9)	−6·2 (−7·4 to–5·0)	79 (45 to 135)	4·3 (−1·6 to 10·6)	−8·6 (−16·2 to–1·5)	46 (26 to 80)	33·8 (30·3 to 37·2)	2·5 (−5·1 to 9·6)	2281 (2075 to 2569)	−4·0 (−4·3 to–3·6)	−6·2 (−7·2 to–5·0)
	Central Asia	3315 (2564 to 4188)	−1·3 (−2·8 to–0·1)	−8·3 (−11·4 to–5·0)	1797 (1330 to 2282)	30·5 (24·1 to 37·6)	−10·6 (−14·5 to–7·0)	1039 (766 to 1322)	58·7 (56·9 to 60·1)	0·5 (−3·4 to 4·1)	6150 (5598 to 6926)	2·4 (1·8 to 2·9)	−8·1 (−9·2 to–6·8)
**Latin America and Caribbean**		15 076 (14 239 to 16 158)	−7·1 (−7·5 to–6·7)	−4·5 (−5·0 to–4·1)	865 (693 to 1085)	6·7 (3·6 to 10·3)	−6·6 (−8·2 to–4·8)	180 (140 to 232)	34·8 (33·3 to 36·4)	3·1 (1·4 to 4·9)	16 121 (15 263 to 17 292)	−6·7 (−7·1 to–6·3)	−4·6 (−5·0 to–4·2)
	Central Latin America	4988 (4703 to 5312)	−7·7 (−8·0 to–7·5)	−4·4 (−5·0 to–3·9)	277 (219 to 349)	13·5 (11·8 to 15·1)	−6·2 (−7·9 to–4·5)	58 (45 to 74)	33·1 (31·6 to 34·6)	3·4 (1·7 to 5·1)	5323 (5030 to 5657)	−7·3 (−7·6 to–7·0)	−4·5 (−5·0 to–4·0)
	Andean Latin America	2800 (2299 to 3434)	−9·8 (−10·7 to–8·7)	−5·9 (−7·4 to–4·3)	335 (214 to 504)	2·8 (−1·3 to 7·7)	−7·1 (−10·9 to–3·3)	70 (44 to 107)	43·9 (41·6 to 46·4)	2·5 (−1·3 to 6·3)	3205 (2723 to 3929)	−9·1 (−9·9 to–7·9)	−5·9 (−7·3 to–4·5)
	Caribbean	1890 (1490 to 2306)	−5·5 (−6·4 to–4·3)	−3·1 (−4·4 to–1·7)	28 (7 to 86)	0·4 (−8·7 to 10·0)	−7·4 (−18·1 to 4·1)	6 (2 to 18)	28·9 (21·5 to 35·5)	2·2 (−8·5 to 13·7)	1923 (1522 to 2335)	−5·4 (−6·3 to–4·2)	−3·1 (−4·4 to–1·8)
	Tropical Latin America	5315 (5065 to 5662)	−4·4 (−4·7 to–4·1)	−4·3 (−4·8 to–3·8)	224 (189 to 263)	19·9 (19·3 to 20·5)	−6·1 (−6·9 to–5·3)	47 (39 to 56)	31·8 (30·5 to 33·0)	3·5 (2·7 to 4·3)	5586 (5330 to 5940)	−4·1 (−4·3 to–3·8)	−4·4 (−4·8 to–3·9)
**Southeast Asia, east Asia, and Oceania**		194 147 (183 457 to 205 231)	−6·1 (−6·7 to–5·6)	−6·4 (−7·0 to–5·7)	11 293 (8191 to 15 536)	3·3 (1·5 to 5·1)	−9·5 (−12·9 to–5·9)	2720 (1951 to 3788)	43·7 (41·8 to 45·5)	2·3 (−1·2 to 5·8)	208 159 (198 135 to 219 944)	−5·7 (−6·3 to–5·3)	−6·5 (−7·1 to–6·0)
	East Asia	38 192 (35 902 to 42 306)	−8·5 (−9·0 to–8·1)	−8·3 (−8·9 to–7·4)	4297 (3483 to 5259)	−0·2 (−1·1 to 0·8)	−11·3 (−12·6 to–10·0)	1035 (822 to 1293)	40·4 (38·9 to 41·8)	0·5 (−0·8 to 1·8)	43 523 (41 277 to 48 120)	−7·8 (−8·2 to–7·4)	−8·5 (−9·1 to–7·6)
	Southeast Asia	155 084 (145 733 to 165 219)	−5·1 (−5·8 to–4·5)	−5·9 (−6·6 to–5·1)	6938 (4218 to 10 905)	9·1 (4·4 to 14·0)	−8·1 (−13·9 to–2·3)	1671 (1012 to 2659)	48·7 (46·0 to 51·4)	3·6 (−2·1 to 9·5)	163 693 (154 881 to 173 618)	−4·8 (−5·5 to–4·1)	−5·9 (−6·6 to–5·2)
	Oceania	573 (457 to 678)	−3·4 (−6·1 to–2·2)	−4·1 (−6·7 to 0·4)	44 (10 to 119)	16·9 (1·4 to 33·3)	−8·6 (−28·3 to 18·6)	11 (2 to 29)	44·2 (27·9 to 52·5)	3·2 (−16·5 to 30·3)	628 (547 to 721)	−2·7 (−3·6 to–1·9)	−4·4 (−5·6 to–3·4)
**North Africa and Middle East**		35 401 (23 520 to 52 168)	−4·4 (−5·1 to–3·7)	−4·7 (−6·0 to–3·5)	3392 (1305 to 7231)	16·5 (10·7 to 22·3)	−4·8 (−14·9 to 4·8)	316 (121 to 684)	39·8 (33·8 to 44·1)	3·2 (−6·9 to 12·8)	39 109 (25 473 to 57 682)	−3·9 (−4·4 to–3·3)	−4·7 (−5·3 to–3·9)
**South Asia**		451 816 (420 616 to 480 353)	−4·7 (−5·2 to–4·3)	−4·9 (−5·6 to–4·3)	52 768 (43 568 to 62 706)	22·0 (21·3 to 22·7)	−5·0 (−5·9 to–4·2)	3416 (2777 to 4170)	46·8 (45·5 to 48·2)	4·5 (3·6 to 5·4)	508 000 (474 024 to 538 330)	−4·0 (−4·5 to–3·6)	−4·9 (−5·6 to–4·2)
**Sub-Saharan Africa**		381 032 (353 681 to 415 691)	−1·3 (−2·2 to–0·5)	−3·8 (−4·4 to–3·3)	20 924 (15 874 to 27 461)	16·1 (14·1 to 18·1)	−4·5 (−7·1 to–1·8)	357 (269 to 473)	37·3 (35·6 to 38·9)	6·7 (4·1 to 9·3)	402 312 (374 030 to 438 809)	−1·0 (−1·8 to–0·2)	−3·8 (−4·4 to–3·4)
	Southern sub-Saharan Africa	35 286 (31 658 to 38 511)	2·9 (0·1 to 4·0)	−5·7 (−6·8 to–4·0)	2604 (1717 to 4034)	15·7 (11·6 to 19·7)	−5·6 (−9·6 to–1·1)	44 (29 to 68)	38·2 (35·6 to 40·9)	5·6 (1·6 to 10·1)	37 935 (34 180 to 41 177)	3·3 (0·5 to 4·3)	−5·7 (−6·8 to–4·1)
	Western sub-Saharan Africa	85 821 (75 972 to 100 174)	−2·8 (−3·5 to–2·1)	−4·4 (−5·6 to–3·3)	5677 (3510 to 8928)	15·7 (12·5 to 19·0)	−6·4 (−12·2 to–0·5)	97 (59 to 154)	36·7 (33·8 to 39·5)	4·8 (−1·0 to 10·7)	91 594 (80 902 to 106 901)	−2·3 (−3·0 to–1·7)	−4·6 (−5·6 to–3·5)
	Eastern sub-Saharan Africa	164 799 (147 918 to 184 516)	−1·7 (−2·8 to 0·2)	−3·7 (−4·5 to–3·0)	10 563 (7041 to 15 603)	21·2 (16·6 to 25·6)	−3·0 (−6·9 to 0·9)	180 (118 to 268)	38·0 (35·9 to 40·0)	8·1 (4·2 to 12·0)	175 543 (157 583 to 196 713)	−1·3 (−2·4 to 0·6)	−3·6 (−4·4 to–3·0)
	Central sub-Saharan Africa	95 003 (75 102 to 114 664)	−0·6 (−1·8 to 0·7)	−2·9 (−3·8 to–2·1)	2072 (1209 to 3262)	8·7 (5·5 to 11·9)	−3·5 (−7·4 to 0·7)	35 (21 to 56)	36·1 (32·8 to 39·3)	7·7 (3·8 to 11·8)	97 110 (76 824 to 117 021)	−0·4 (−1·6 to 0·8)	−3·0 (−3·8 to–2·1)

Data in parentheses are 95% uncertainty intervals. Multidrug-resistant tuberculosis=multidrug-resistant tuberculosis without extensive drug resistance.

Globally in 2016, among HIV-negative individuals, we estimated that 0·30 million (95% UI 0·26–0·34) incident cases of tuberculosis were multidrug resistant (table 1) and 0·10 million (0·08–0·11) deaths were due to multidrug-resistant tuberculosis (table 2). In individuals who are HIV positive, we estimated that 35 815 (23 524–51 741) incident cases of tuberculosis were multidrug-resistant (appendix pp 75–87) and 18 375 (11 208–27 747) deaths were due to multidrug-resistant tuberculosis (appendix pp 88–100). Among HIV-negative individuals in 2016, we estimated 18 452 (16 087–21 187) incident cases of tuberculosis were extensively drug resistant and 10 920 (8896–13 162) deaths were due to extensively drug-resistant tuberculosis (Table 1, Table 2). Among HIV-positive individuals in 2016, we estimated that 1303 (793–2019) incident cases of tuberculosis were extensively drug resistant and 1151 (689–1802) deaths were due to extensively drug-resistant tuberculosis (appendix pp 75–100). Estimated tuberculosis prevalence by drug-resistance type and HIV status are available online.

### Changes in the burden of tuberculosis over time

Globally, the annualised rate of change in age-standardised incidence of tuberculosis among HIV-negative individuals was −1·3% (95% UI −1·5 to −1·2) from 2006 to 2016 (table 1), which is a slower rate of change than in 1990–2006 (–1·6% [–1·8 to −1·3]; table 1). These rates of change are small compared with the decrease in the annualised rate of change in age-standardised tuberculosis mortality (–4·5% [–5·0 to −4·1]) from 2006 to 2016, which is larger than the annualised rate of change from the period 1990–2006 (–3·2% [–3·7 to −2·9]; table 2).

Globally, the annualised rate of change in age-standardised incidence of tuberculosis among HIV-positive individuals decreased from 2006 to 2016 (–4·0% [95% UI −4·5 to −3·7]), whereas in 1990–2006 the rate of change increased (8·1% [7·5–8·8]; appendix pp 75–87). Mortality among HIV-positive individuals has decreased, with an annualised rate of change of −8·9% (–9·5 to −8·4) for 2006–16, which is a substantial change from the annualised increase of 9·2% (8·3–10·1) for 1990–2006 (appendix pp 88–100).

Globally from 2006 to 2016, the annualised rate of change in age-standardised incidence of multidrug-resistant tuberculosis among HIV-negative individuals was −2·1% (95% UI −2·9 to −1·3; table 1), and the rate of change in mortality was −5·5% (–6·5 to −4·5; table 2). By contrast, we estimated that the burden of extensively drug-resistant tuberculosis has increased globally. From 2006 to 2016, the annualised age-standardised rate of change in the incidence of extensively drug-resistant tuberculosis was 7·9% (6·6–9·1; table 1), and the rate of change for mortality was 3·1% (1·8–4·5; table 2). For the same time period, the annualised age-standardised rates of change for multidrug-resistant tuberculosis among HIV-positive individuals was −4·6% (–6·6 to −2·7) for incidence and −9·1% (–11·2 to −7·2) for mortality. For extensively drug-resistant tuberculosis among HIV-positive individuals, the annualised rate of change was 7·2% (5·4 to 8·7) for incidence and 2·3% (0·9 to 3·7) for mortality (appendix pp 75–100).

### Region-specific and country-specific tuberculosis incidence and mortality

**Figure 3:**
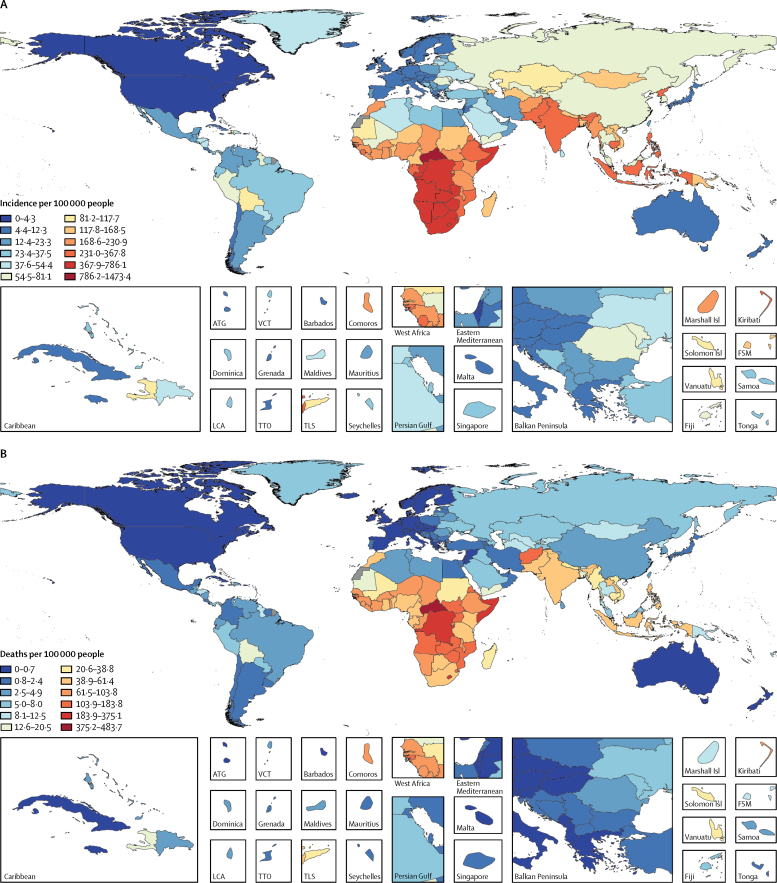
Age-standardised tuberculosis incidence (A) and mortality (B) in HIV-negative individuals, 2016 ATG=Antigua and Barbuda. VCT=Saint Vincent and the Grenadines. LCA=Saint Lucia. TTO=Trinidad and Tobago. Isl=Islands. FSM=Federated States of Micronesia. TLS=Timor-Leste.

Although we observed a global decrease in the burden of tuberculosis, this trend was not uniform across all regions and countries. During 2006–16, among HIV-negative individuals the annual percentage change in the incidence of tuberculosis varied from −6·2% (95% UI −6·7 to −5·6) in Kazakhstan, to 1·2% (0·7–1·8) in the Philippines, and 1·3% (0·6–2·1) in Uruguay (appendix pp 48–61). In 2016, the age-standardised incidence rate (per 100 000 population) of tuberculosis in HIV-negative individuals varied from 3·1 (2·8–3·4) in the USA and 3·8 (2·5–5·4) in Palestine, to 729·6 (537·3–1013·1) in Lesotho and 842·7 (670·9–1040·0) in Central African Republic (figure 3; appendix pp 101–07).

Age-standardised rates of tuberculosis mortality among HIV-negative individuals decreased at varying rates across countries and territories from 2006 to 2016, with the highest annual decreases seen in Kazakhstan (–13·7% [95% UI −16·2 to −11·3]), Estonia (–9·4% [–11·8 to −7·1]), Kuwait (–9·0% [–12·1 to −6·2]), China (–8·8% [–9·5 to −7·9]), and Myanmar (–8·8% [–10·4 to −7·0]; appendix pp 62–74). Among HIV-negative individuals in 2016, age-standardised mortality rates (per 100 000 population) of tuberculosis were the highest (ie, >150 deaths per 100 000 population) in Burundi, Central African Republic, Democratic Republic of the Congo, Lesotho, Somalia, and Zambia (figure 3). The corresponding age-standardised incidence and mortality rates of tuberculosis among HIV-positive individuals, by country and with annual percentage changes, are in the appendix (pp 47, 75–100).

Trends in the annualised rate of change in age-standardised incidence and mortality for multidrug-resistant tuberculosis varied largely across countries, with no consistent pattern worldwide for HIV-negative (appendix pp 48–74) or for HIV-positive individuals (pp 75, 88). Among HIV-negative individuals, Kyrgyzstan, Lesotho, Namibia, Somalia, Swaziland (eSwatini), and Turkmenistan had the highest age-standardised incidence of multidrug-resistant tuberculosis (ie, >20 per 100 000 population) in 2016, whereas age-standardised mortality for multidrug-resistant tuberculosis was highest (ie, >15 per 100 000 population) in Somalia, Lesotho, eSwatini, and Afghanistan in the same year (appendix pp 108–14). More detailed results for HIV-negative individuals broken down by age, sex, and year, and data for HIV-positive individuals, are available online.

### Observed versus expected tuberculosis burden

**Figure 4:**
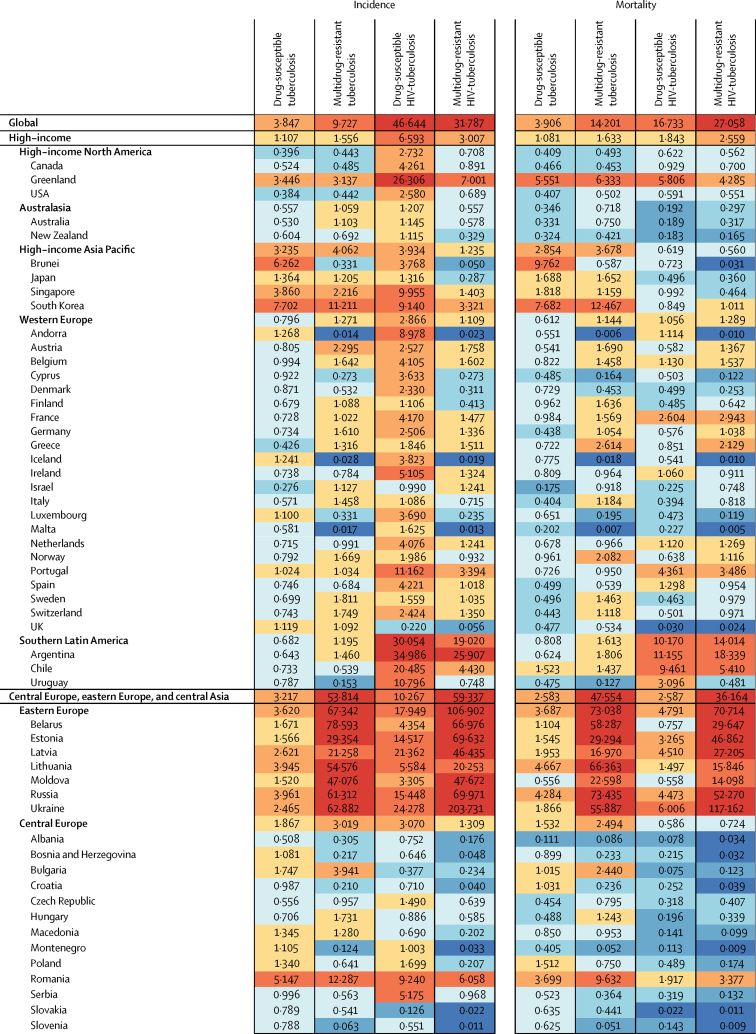

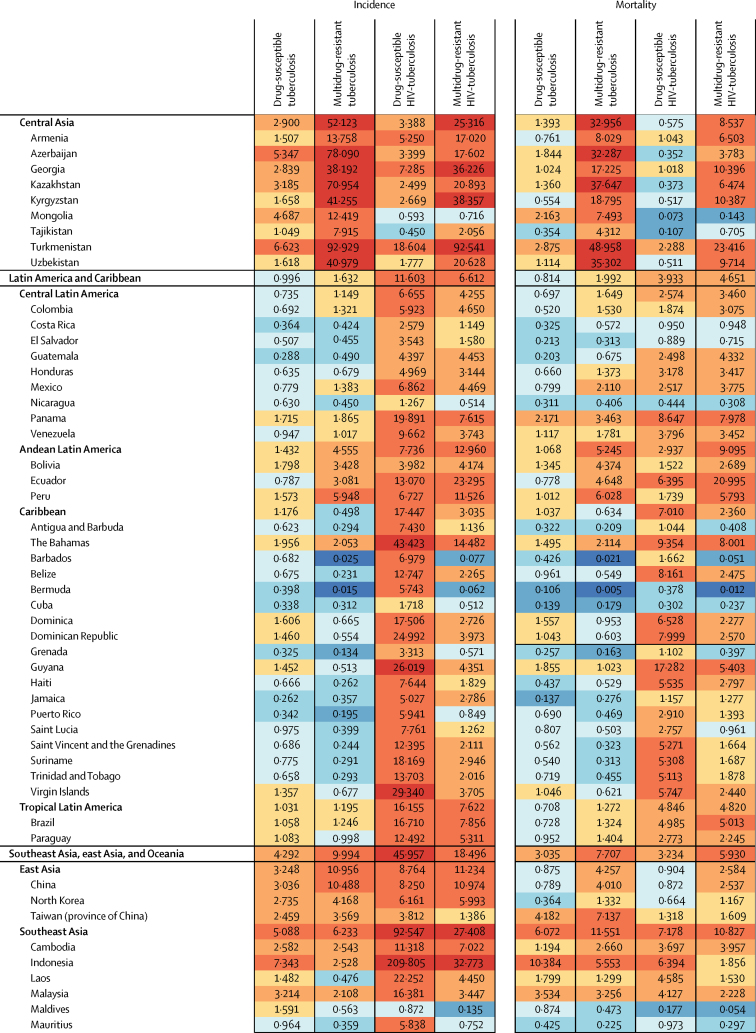

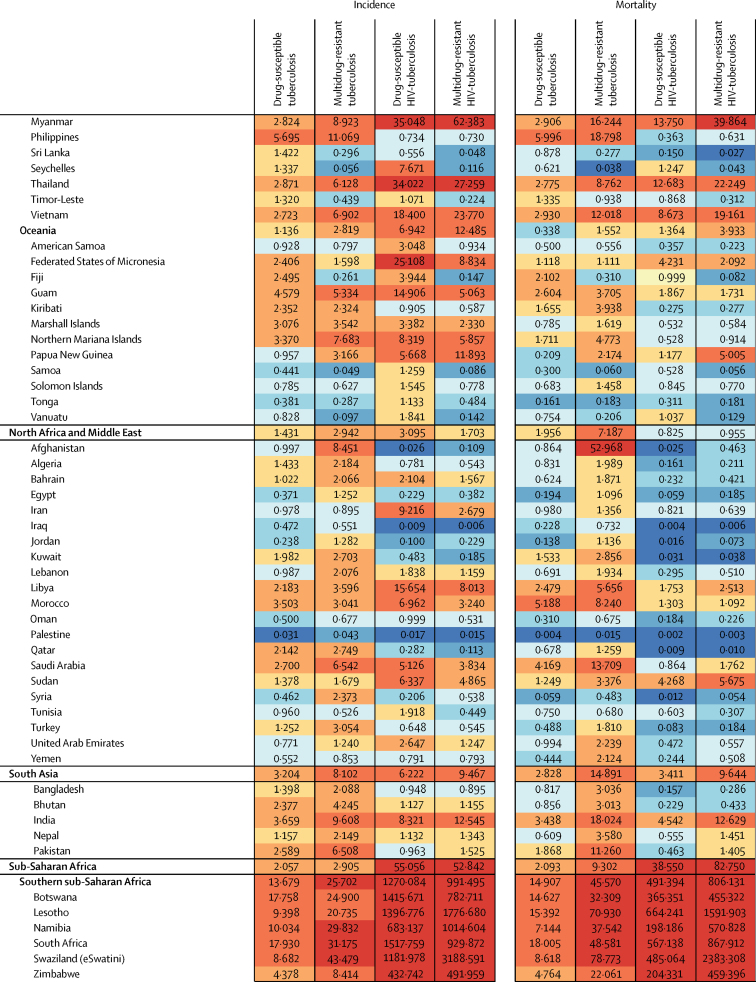

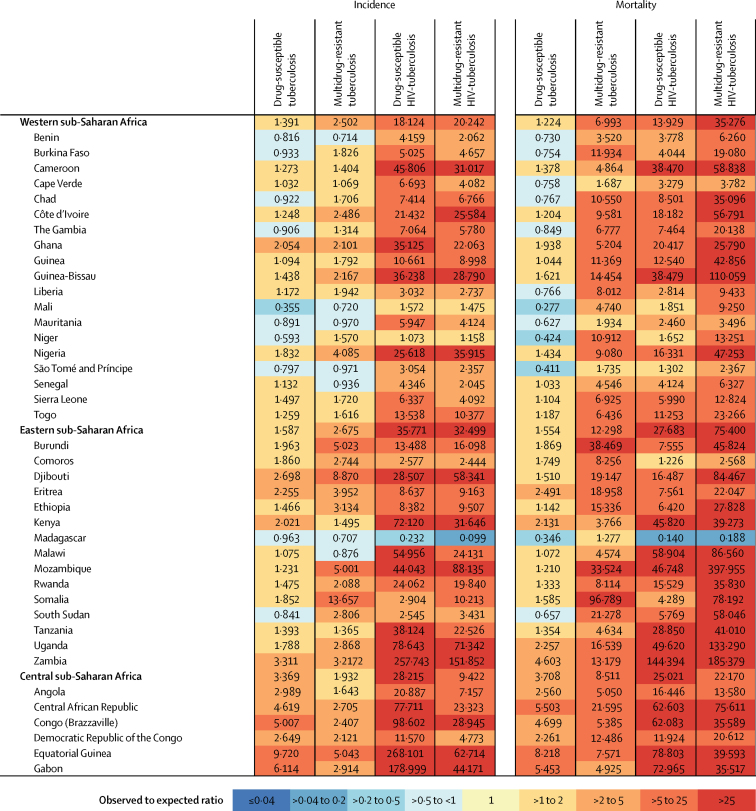
Ratio of observed to expected age-standardised incidence and mortality on the basis of SDI by GBD region and country in 2016, for drug-susceptible tuberculosis and multidrug-resistant tuberculosis, by HIV status Ratio of observed age-standardised incidence or mortality to that expected on the basis of a country's SDI for a given year. A ratio of one means that observed and expected values are equal. A ratio higher than one means the observed rate is greater than expected, and a ratio of less than one means the observed rate is lower than expected. GBD=Global Burden of Disease. Multidrug-resistant tuberculosis=multidrug-resistant tuberculosis without extensive drug resistance. SDI=Socio-demographic Index.

In 2016, among HIV-negative individuals, several regions (eg, eastern Europe, central Asia, southeast Asia, south Asia, and sub-Saharan Africa) had higher than expected (on the basis of SDI) age-standardised incidence and mortality of drug-susceptible tuberculosis (figure 4). At the regional level, the highest observed-to-expected ratios were in southern sub-Saharan Africa (13·7 for incidence and 14·9 for mortality), and the lowest ratios were in high-income North America (0·4 for incidence) and Oceania (0·3 for mortality). For multidrug-resistant tuberculosis, eastern Europe had the highest observed-to-expected ratios for incidence (67·3) and mortality (73·0), and high-income North America had the lowest ratios (0·4 for incidence and 0·5 for mortality). We found no association between SDI and incidence of or mortality due to extensively drug-resistant tuberculosis (data not shown).

In 2016, among HIV-negative individuals, observed-to-expected ratios were greater than two for incidence of drug-susceptible tuberculosis in 54 countries, for drug-susceptible tuberculosis mortality in 38 countries, for incidence of multidrug-resistant tuberculosis in 83 countries, and for multidrug-resistant tuberculosis mortality in 96 countries (figure 4). These countries were located mainly in sub-Saharan Africa and southeast Asia. For HIV-positive individuals in 2016, observed-to-expected ratios were greater than two in 138 countries for incidence of drug-susceptible tuberculosis, 82 countries for drug-susceptible tuberculosis mortality, 105 countries for incidence of multidrug-resistant tuberculosis, and 95 countries for multidrug-resistant tuberculosis mortality. Most of these countries were in sub-Saharan Africa and eastern Europe. Across tuberculosis drug-resistance types and by HIV-status, the highest observed-to-expected ratios were 17·9 for the incidence of drug-susceptible tuberculosis in South Africa to 3188·6 for the incidence of multidrug-resistant HIV-tuberculosis in eSwatini.

## Discussion

This study provides a comprehensive assessment of levels and trends in the burden of tuberculosis by drug-resistance type and HIV status for 195 countries and territories over the past 27 years. Despite the fact that HIV and drug-resistant tuberculosis have emerged as big challenges to tuberculosis control efforts, most incident cases of tuberculosis and deaths due to tuberculosis in 2016 occurred in HIV-negative individuals who were susceptible to first-line tuberculosis drugs. More than half of these cases and deaths occurred in south and southeast Asia. HIV-tuberculosis comprises 13% (1·40 million of 10·42 million) of incident cases of tuberculosis and 16% (0·24 million of 1·45 million) of deaths due to tuberculosis, most of which occurred in sub-Saharan Africa. Over the past decade, the global rate of decrease in the incidence of tuberculosis is about a third for HIV-negative individuals, and a half for HIV-positive individuals, compared with the rate of decrease in tuberculosis mortality, with substantial variations between countries. Trends in multidrug-resistant tuberculosis also vary largely across countries, with no consistent pattern worldwide. Several regions had a higher burden of tuberculosis than expected given their level of socio-demographic development in 2016.

We estimated that the incidence of tuberculosis among HIV-negative individuals has decreased by only 1·3% (95% UI 1·2–1·5) annually during 2006–16. This rate is much lower than the 10% or more annual reduction needed by 2025 to reach the SDG target to end the tuberculosis epidemic by 2030.^3^ We identified the countries with the fastest and slowest improvements in tuberculosis incidence during 2006–16. The fastest annual decrease in incidence was observed in Kazakhstan, where improvements were attributable to advances in diagnostics and effective treatment of newly diagnosed tuberculosis cases.^23^ We saw little to no improvement in some countries, including the Philippines and Uruguay. In the Philippines, a high proportion of smear-negative individuals who are positive for tuberculosis by Xpert MTB/RIF assay (Cepheid, USA) has been documented in high-risk populations, including prison inmates and indigenous populations, suggesting that sputum-smear microscopy alone as a routine diagnostic test is inadequate.^24^ In Uruguay, a decrease in treatment success rate for new cases of tuberculosis (from 84% in 2010 to 77% in 2015)^25^ probably contributes to the country's the lack of progress.

Despite improvements in socio-demographic conditions, several regions have fallen behind in their progress to reduce the burden of tuberculosis. In 2016, most countries in Asia, sub-Saharan Africa, and eastern Europe had a higher burden of tuberculosis (both drug susceptible and multidrug resistant) than expected given their level of socio-demographic development. In many countries, providing treatment services for multidrug-resistant tuberculosis remains a challenge, partly because of the high cost of second-line drugs and poor adherence to regimens.^26^, ^27^ Globally in 2016, only 22% of people with newly diagnosed drug-resistant tuberculosis were estimated to begin treatment, with a treatment success rate of 54%.^28^ Evidence suggests that alcohol abuse and HIV infection are associated with increased risk of unsuccessful outcomes in patients with multidrug-resistant tuberculosis, but the association is unclear for other factors and comorbidities such as smoking and chronic kidney disease.^29^ A more comprehensive understanding of the key drivers of unsuccessful treatment outcomes in these patients is crucial to improving their treatment outcomes.

Although the burden of tuberculosis remains far off from the expected level in many countries with a high burden of tuberculosis, the prevalence of diabetes—an important risk factor for both tuberculosis and adverse outcomes from tuberculosis treatment—has increased over time worldwide as a consequence of several factors including population ageing and exposure to lifestyle-related risk factors,^11^, ^30^ creating additional challenges for tuberculosis care and prevention. Because of the interaction of tuberculosis with diabetes and HIV/AIDS, integrating control programmes for the three diseases could help prevent tuberculosis among people with HIV/AIDS and diabetes and reduce the burden of all three diseases.^31^ Additionally, efforts to prevent other risk factors for tuberculosis, including smoking and alcohol misuse, could have a complementary effect on the burden of tuberculosis.^2^

In countries with a higher burden of tuberculosis than expected on the basis of SDI, an important first step is to identify the reasons for falling behind so that appropriate measures can be taken. Gaps in case detection and delays in diagnosis and treatment most likely contribute to the burden being higher than expected, and country-specific reasons should also be investigated. Although national tuberculosis programmes were notified of about 6·3 million new or relapsed cases of tuberculosis globally in 2016,^28^ we estimated that the number of incident cases in 2016 was 10·4 million, implying a global case detection rate of 61%. Evidence suggests that a routine passive case-finding strategy is insufficient for detecting all tuberculosis cases.^32^, ^33^ Active case finding has been recommended as a complementary strategy to passive case finding to increase case detection; nevertheless, the effect of active case finding on treatment outcomes and rate of transmission, and longer-term effects on the epidemiology of tuberculosis, have yet to be determined.^32^

Also, despite advances in tuberculosis diagnostics, smear microscopy remains the most commonly used diagnostic test in many countries that are endemic for tuberculosis.^34^ The Xpert MTB/RIF assay has higher sensitivity for the detection of tuberculosis than smear microscopy,^35^ but few countries use Xpert for general tuberculosis case finding.^36^ Policies on the use of Xpert MTB/RIF vary largely between countries, with only a subset of patients with tuberculosis being eligible for the test (eg, patients with suspected drug resistance, HIV-positive individuals).^36^ A scale-up of Xpert MTB/RIF could help in detecting additional cases, but it has been impeded by several factors, including high costs, reliance on funding from international donors, and the lack of subsidised pricing in the private sector, which is relied on for most tuberculosis cases in some countries.^26^

Overall, even with differences in methods used, both GBD 2016 and WHO estimated 10·4 million incident cases of tuberculosis in 2016, although our estimated number of all tuberculosis deaths (1·45 million) is lower than WHO's estimate (1·7 million) for 2016.^28^ The 20 countries with the highest burden, as assessed by the number of incident cases, differ between our estimates and WHO's: WHO includes Angola, Brazil, and Thailand; instead, we include Uganda, Zambia, and Zimbabwe. The most notable difference between our and WHO's estimates is between the estimated numbers of tuberculosis deaths among children. We estimated 39 311 deaths (95% UI 34 415–44 847) among children who are HIV negative and younger than 15 years for 2016, which is substantially lower than the estimates from WHO (201 000 deaths)^28^ and Dodd and colleagues (200 000 deaths).^37^ The input data and the methods used to generate estimates of deaths in children due to tuberculosis are very different between studies: we used vital registration and verbal autopsy data and the CODEm strategy to estimate tuberculosis deaths in children and WHO used the method of Dodd and colleagues from their 2017 study^37^ in which child mortality due to tuberculosis was back-calculated from the incidence and case-fatality ratio. WHO estimated the incidence of tuberculosis in children by combining results from two approaches: the case detection rate adjustment approach (ie, incidence=notifications/estimated case detection rate); and the method of Dodd and colleagues from their 2014 study^38^ in which incidence was estimated from the annual risk of infection in children, WHO adult smear-positive tuberculosis prevalence data, and demographic information by use of a mathematical model. Both our method and the method used by WHO and Dodd and colleagues have limitations. Specifically, concerns have been raised about the misclassification of tuberculosis deaths in children as deaths due to pneumonia in countries with a high burden of tuberculosis.^39^ In this study**,** we did not redistribute pneumonia deaths to tuberculosis deaths because of a lack of evidence on whether tuberculosis is a cause or comorbidity of acute severe pneumonia in children.^40^ The back-calculation approach used by WHO and Dodd and colleagues most likely has substantial uncertainty due to assumptions in the process of estimating annual risk of infection, the prevalence of adult tuberculosis, and case detection rates.

Our study has several limitations. First, our assessment of the trends in the burden of multidrug-resistant tuberculosis was restricted by a paucity of time-series data for many countries in Asia and Africa. We assumed that these countries have a similar age-sex distribution of multidrug-resistant tuberculosis to other countries in the same region and used this common distribution to generate trend estimates for countries and years with little data; the lack of data in a particular country is reflected in wide uncertainty intervals. Second, verbal autopsy studies have modest sensitivity in identifying tuberculosis deaths.^41^, ^42^, ^43^ However, at the typical range of the cause fraction of deaths due to tuberculosis in India and sub-Saharan Africa (3–5%),^43^ and at the reported level of sensitivity and specificity of attributing tuberculosis as the cause of death in a large, multicentre, verbal autopsy validation study,^43^ we estimate that the false positives and false negatives largely cancel out. Third, as noted in our previous publication,^2^ the main challenge in our statistical triangulation approach has been the shortage of data from surveys on cause of death and prevalence, particularly from countries in sub-Saharan Africa with a high prevalence of HIV. We applied sophisticated modelling methods and covariates, using distributions across geographies and time to help predict for those locations. Accordingly, the estimates for a location with sparse data are coupled with wider uncertainty intervals. Fourth, to inform the case-fatality ratio among patients with untreated tuberculosis for our mortality-to-incidence ratio regression, we used data from a single community-based follow-up study done in Bangalore, India;^21^ two other community-based studies,^44^ done in India^45^ and the USA,^46^, ^47^ were not included because of a lack of information about the treatment of tuberculosis or any systematic follow-up of cases.

Despite these limitations, we made several improvements in our methods compared with GBD 2015. First, we no longer used case-detection rates based on expert opinion in the process of estimating the incidence of tuberculosis. Instead, we used a mortality-to-incidence ratio approach to better reflect higher mortality and incidence in low-income and middle-income countries. Second, we strengthened our statistical triangulation approach by incorporating population-based surveys of latent tuberculosis infection, and modelling incidence, prevalence, and mortality simultaneously among the population who are latently infected to enhance comparability across countries. Because we used Bayesian meta-regression to generate an incidence estimate that is consistent with prevalence data or cause-specific mortality estimates, our estimated incidence might differ from countries' official statistics (even from those with a four star or five star quality ratings). Third, we improved our estimates of tuberculosis mortality by including additional covariates that have proximal or strong associations with tuberculosis mortality (ie, prevalence of latent tuberculosis infection, prevalence of active tuberculosis disease, proportion of adults who are underweight, and HAQ Index). These improvements, together with substantial efforts to collate data for the estimation of tuberculosis burden, have resulted in changes in GBD 2016 compared with GBD 2015, especially in estimates of mortality. The global number of deaths in 2016 due to tuberculosis was 11% higher than the GBD 2015 estimate for 2015. The increase mainly occurred in some African countries—notably, Burundi, Central African Republic, Congo, Democratic Republic of the Congo, Gabon, Nigeria, Uganda, and Zambia had more than twice the number of estimated deaths in 2016 compared with in 2015. Fourth, in GBD 2015, we did not separately examine the burden of multidrug-resistant tuberculosis. Given their epidemiological and clinical importance, we included estimates of multidrug-resistant and extensively drug-resistant tuberculosis in GBD 2016. Further estimation and mapping of the burden of tuberculosis by drug-resistance type and HIV status at a finer spatial resolution could better inform surveillance and the targeting of resources for interventions.^48^

As countries work towards achieving the SDG target to end the tuberculosis epidemic by 2030, contemporary information on the levels and trends of the burden of tuberculosis is essential to track and monitor the progress of control efforts in individual countries. Locations with the greatest improvements in controlling tuberculosis could provide insight into successful programmatic strategies for countries with stagnant progress. Our findings suggest that, if current trends in tuberculosis incidence continue, few countries will meet the SDG target. Progress needs to be accelerated by improving the quality of and access to tuberculosis diagnosis and care, scaling up of interventions to prevent risk factors for tuberculosis, and integrating control programmes for tuberculosis, HIV, and diabetes.

Correspondence to: Prof Christopher J L Murray, Institute for Health Metrics and Evaluation, Seattle, WA 98121, USA cjlm@uw.edu

## Data sharing

To download the data used in these analyses, please visit the Global Health Data Exchange at http://ghdx.healthdata.org/node/373720.
