# Supplementary material for: Global, regional, and national burden of tuberculosis, 1990–2016: results from the Global Burden of Diseases, Injuries, and Risk Factors 2016 Study
Source: Lancet Infect Dis. 2018 Dec;18(12):1329–49. doi: 10.1016/S1473-3099(18)30625-X (PMC6250050; doi:10.1016/S1473-3099(18)30625-X)
Supplement: Supplementary appendix [file mmc1.pdf]

THE LANCET Infectious Diseases

Supplementary webappendix

This webappendix formed part of the original submission and has been peer reviewed. We post it as supplied by the authors.

Supplement to: GBD Tuberculosis Collaborators. Global, regional, and national burden of tuberculosis, 1990–2016: results from the Global Burden of Diseases, Injuries, and Risk Factors 2016 Study. *Lancet Infect Dis* 2018; **18:** 1329–49.

Appendix

Contents

Methods....3

Case Definition....3

Methods Overview....4

Non-fatal Tuberculosis....5

Flowchart....5

Modelling non-fatal TB....6

HIV-TB incidence and prevalence....13

MDR-TB, XDR-TB and drug-susceptible TB among HIV negative individuals....13

MDR HIV-TB, XDR HIV-TB and drug-susceptible HIV-TB....15

Fatal tuberculosis among HIV-negative individuals....18

Flowchart....18

Mortality from TB among HIV-negative individuals....19

Mortality from MDR-TB, XDR-TB, and drug-susceptible TB among HIV-negative individuals....23

Fatal tuberculosis among HIV-positive individuals....34

Flowchart....34

HIV-TB mortality....35

Mortality from MDR-HIV-TB, XDR-HIV-TB and drug-susceptible HIV-TB....36

Systematic review....37

eTable 4. Links to the code and data sources used....40

eTable 5. A comparison of methods used by GBD and WHO for estimating the burden of tuberculosis....42

Results....46

eFigure 9. Global age-sex distribution of tuberculosis incidence and deaths in HIV-positive individuals in 2016 46

eFigure 10. Age-standardized Incidence and deaths rates, per 100,000, for HIV-positive Tuberculosis in 2016 .47

eTable 6. Tuberculosis, drug-susceptible tuberculosis, multidrug-resistant tuberculosis, and extensively drug-resistant tuberculosis **incident cases in HIV-negative individuals**, and annualized rates of change of age-standardized rates for **195 countries and territories**....48

eTable 7. Tuberculosis, drug-susceptible tuberculosis, multidrug-resistant tuberculosis, and extensively drug-resistant tuberculosis **deaths in HIV-negative individuals**, and annualized rates of change of age-standardized rates for **195 countries and territories**....62

eTable 8. Tuberculosis, drug-susceptible tuberculosis, multidrug-resistant tuberculosis, and extensively drug-resistant tuberculosis **incident cases in HIV-positive individuals**, and annualized rates of change of age-standardized rates for **195 countries and territories**........75

eTable 9. Tuberculosis, drug-susceptible tuberculosis, multidrug-resistant tuberculosis, and extensively drug-resistant tuberculosis **deaths in HIV-positive individuals**, and annualized rates of change of age-standardized rates for **195 countries and territories** 88

eTable 10. Age-standardized incidence and mortality rates per 100,000 for tuberculosis among HIV-negative individuals in 2016, both sexes (Input data for Figure 3) 101

eTable 11. Age-standardized incidence and mortality rates per 100,000 for multi-drug resistant tuberculosis among HIV-negative individuals in 2016, both sexes 108

References 115

Methods

Case Definition

TB is an infectious disease caused by Mycobacterium tuberculosis. The case definition includes all forms of TB including pulmonary TB and extrapulmonary TB which are bacteriologically confirmed or clinically diagnosed. For TB, the ICD 10 codes are A10-A19.9, B90-B90.9, K67.3, K93.0, M49.0, P37.0, and ICD 9 codes are 010-019.9, 137-137.9, 138.0, 138.9, 139.9, 320.4, 730.4-730.6. For HIV-TB, the ICD 10 code is B20.0.

We separately estimated the incidence, prevalence, and mortality of multidrug-resistant tuberculosis (MDR-TB) and extensively drug-resistant tuberculosis (XDR-TB) by HIV status in GBD 2016. The case definitions of the new causes are shown below.

(1) MDR-TB without extensive drug resistance: a form of TB (among HIV-negative individuals) that is resistant to the two most effective first-line anti-tuberculosis drugs (isoniazid and rifampicin), but is not resistant to any fluoroquinolone and any second-line injectable drugs (amikacin, kanamycin, or capreomycin).

(2) XDR-TB: a form of TB (among HIV-negative individuals) that is resistant to isoniazid and rifampicin, plus any fluoroquinolone and any second-line injectable drugs.

(3) Drug-susceptible TB: TB (among HIV-negative individuals) that is susceptible to isoniazid and rifampicin

(4) MDR-HIV-TB without extensive drug resistance: a form of TB (among HIV-positive individuals) that is resistant to the two most effective first-line anti-tuberculosis drugs (isoniazid and rifampicin), but is not resistant to any fluoroquinolone and any second-line injectable drugs (amikacin, kanamycin, or capreomycin).

(5) XDR-HIV-TB: a form of TB (among HIV-positive individuals) that is resistant to isoniazid and rifampicin, plus any fluoroquinolone and any second-line injectable drugs

(6) Drug-susceptible HIV-TB: TB (among HIV-positive individuals) that is susceptible to isoniazid and rifampicin

Methods Overview

We analysed vital registration data, verbal autopsy data, sample-based vital registration data, and mortality surveillance data, using the Cause of Death Ensemble model (CODEm) to estimate TB mortality among HIV-negative individuals. We estimated HIV-TB mortality using a population attributable fraction approach taking into account baseline risk. We also used a population attributable fraction approach to estimate the fraction of HIV-negative TB deaths attributable to MDR-TB, and the fraction of HIV-TB deaths attributable to MDR-TB, respectively, and used these fractions to split TB deaths and HIV-TB deaths by drug-resistance type. For estimating non-fatal TB, we analysed all available data sources, including annual case notifications, prevalence surveys, population-based tuberculin surveys, and estimated TB cause-specific mortality, to generate internally consistent estimates of incidence, prevalence, and mortality using DisMod-MR 2.1, a Bayesian meta-regression tool. To distinguish HIV-TB from all forms of TB, we applied the proportions of HIV-TB cases among all TB cases estimated from a mixed-effects regression to TB incident and prevalent cases. We then applied the estimated proportions of TB cases with MDR-TB and HIV-TB cases with MDR-TB to our predicted TB cases and HIV-TB cases, respectively, to generate MDR-TB cases by HIV status.

Non-fatal Tuberculosis

Flowchart

eFigure 1. Non-fatal tuberculosis (TB) by drug-resistance type and HIV status: input data, analytical process, and output

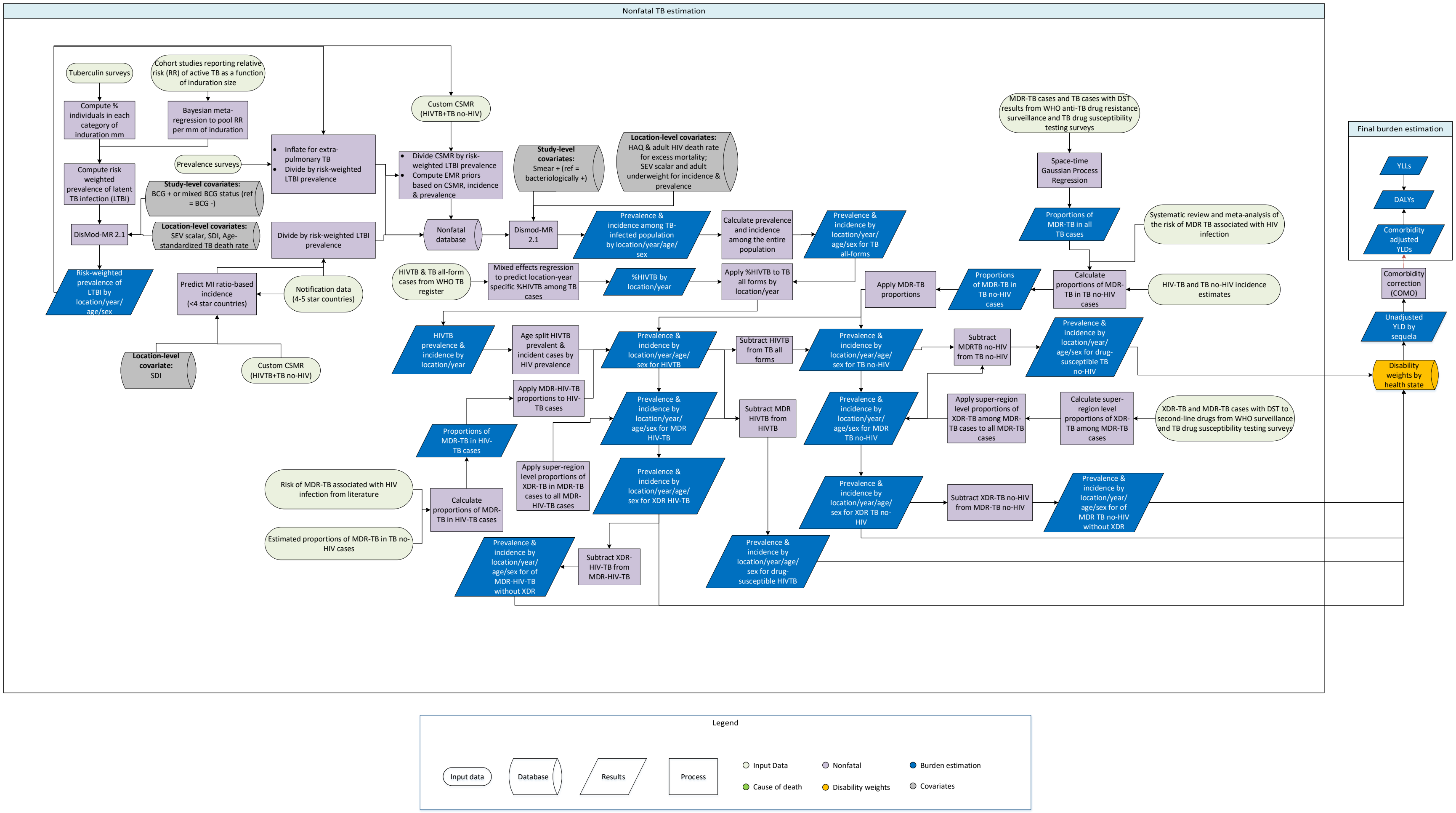

MI ratio=Mortality to incidence ratio; SDI=Sociodemographic index; HAQ=Healthcare access and quality index; SEV=Summary Exposure Variable

Modelling non-fatal TB

Input data include annual case notifications, data from prevalence surveys, and estimated cause-specific mortality (CSMR) of TB among HIV-positive and HIV-negative individuals. We divided the inputs on prevalence, incidence, and CSMR by the estimated latent TB infection (LTBI) prevalence weighted by the risk of progression to active TB in order to model TB among those at risk in each country. From these inputs, we calculated priors (expected values) on excess mortality to give more guidance to the model (The computation of excess mortality rate was provided in detail on page 10 of the Appendix). We used DisMod-MR 2.1, the GBD Bayesian meta-regression tool that adjusts for differences in methods between data sources and imposes consistency between data for different parameters. We then multiplied the DisMod-MR 2.1 outputs by the prevalence of LTBI to get population-level estimates of incidence and prevalence. We explain in more detail below the preparation of each of the input data sources and the modelling in DisMod-MR 2.1.

Modeling TB incidence

Incidence inputs were from two different sources: (1) notification data for countries with a 4 to 5-star rating, and (2) estimated incidence for countries with a zero to three-star rating (see page 18 for more details about the quality assessment). We used the age and sex-specific notifications (all new and relapse cases combined) in our analysis. Starting from 2013, notified new cases and relapse cases are aggregated and reported together, and we used the data as they were reported for countries with a 4 to 5-star rating. Prior to 2013, notification data were available by case type (new pulmonary smear-positive, new pulmonary smear-negative, and new extra-pulmonary). We imputed the missing age-groups for the three forms of TB notifications. Smear-positive age-specific notifications were inflated with the proportion smear-unknown and relapsed cases only reported at the country-year level. Missing smear-negative and extra-pulmonary cases were predicted from the adjusted smear-positive cases using a seemingly unrelated regression. All three types of notifications were added together to

represent TB-all form incidence for countries with a four or five-star rating. To run a seemingly unrelated regression, we first generated age-standardized smear positive rate from the adjusted smear positive age-sex-specific data. The smear positive age-standardized rate as well as age and sex dummies were included as independent variables in the seemingly unrelated regression using the following Stata command:

sureg (y1_snsp sex_2 age0 age5 age15 age25 age45 age55 age65 sp_agestd_rt) (y2_epsp sex_2 age0 age5 age15 age25 age45 age55 age65 sp_agestd_rt)

where *y1_snsp* is the ratio of smear negative TB to smear positive TB (log-transformed), *y2_epsp* is the ratio of extrapulmonary TB to smear positive TB (log-transformed), *sex_2* is female (reference is male), *sp_agestd_rt* is smear positive age-standardized rate, and age0 to age65 are age dummies representing seven age groups (0 to 4 years, 5 to 14 years, 15 to 24 years, 25 to 34 years, 45 to 54 years, 55 to 64 years, and 65 years and older). We used the 35-44 year age group as the reference category. All the variables were derived from notification data. The Stata-regression output is shown below.

	Coef.	Std. Err.	z	P>|z|	[95% Conf.	Interval]
**y1_snsp**						
sex_2	.1196044	.0241798	4.95	0.000	.0722129	.166996
age0	2.598042	.0537174	48.37	0.000	2.492757	2.703326
age5	1.44353	.0491285	29.38	0.000	1.34724	1.53982
age15	.0100879	.0466424	0.22	0.829	-.0813296	.1015054
age25	-.0312117	.0465922	-0.67	0.503	-.1225308	.0601074
age45	.0770768	.0465922	1.65	0.098	-.0142422	.1683959
age55	.2340705	.0466047	5.02	0.000	.1427269	.3254141
age65	.3323278	.0464807	7.15	0.000	.2412274	.4234283
sp_agestd_rt	-148.4488	11.89105	-12.48	0.000	-171.7548	-125.1428
_cons	-.5148792	.0357868	-14.39	0.000	-.58502	-.4447384
**y2_epsp**						
sex_2	.3724358	.0254211	14.65	0.000	.3226113	.4222603
age0	2.617413	.0564751	46.35	0.000	2.506724	2.728102
age5	1.731977	.0516506	33.53	0.000	1.630744	1.83321
age15	.0415483	.0490369	0.85	0.397	-.0545622	.1376589
age25	-.0110172	.0489841	-0.22	0.822	-.1070243	.0849899
age45	-.0753961	.0489841	-1.54	0.124	-.1714032	.0206109
age55	.040747	.0489973	0.83	0.406	-.0552858	.1367799
age65	.0737702	.0488668	1.51	0.131	-.0220071	.1695475
sp_agestd_rt	-265.1473	12.5015	-21.21	0.000	-289.6498	-240.6448
_cons	-.9195986	.037624	-24.44	0.000	-.9933402	-.845857

To generate initial incidence estimates for countries with a zero to three-star rating, we ran a regression using mortality to incidence (MI) ratios (logit transformed) from locations with high quality vital registration data as input data with SDI as a covariate anchoring the lower end of the SDI scale with a data point from a cohort study in the 1960s[1] reporting that 49.2% of 126 untreated new pulmonary TB cases were dead at the end of the 5-year follow up period, in order to predict age-sex specific MI ratios for all locations and years. The MI ratios were then adjusted for HIV using the following formula:

$$MIadjusted_{c,y,a,s} = MI_{c,y,a,s} * (HIVpr_{c,y,a,s} * RRhiv + (1 - HIVpr_{c,y,a,s}) * 1))$$

where $MI_{c,y,a,s}$ is country-year-age-sex specific MI ratio, $HIVpr_{c,y,a,s}$ is the proportion of HIV-TB cases among all TB cases by country, year, age, and sex, and $RRhiv$ is the median relative risk of TB death associated with HIV infection (Input data and methods used to derive the median relative risk (1.726) was described in detail on page 34 of the Appendix).

We then used the MI ratios and cause specific mortality estimates to compute the incidence input for DisMod-MR 2.1. In locations where estimated MI ratios were greater than notification-based MI ratios, we used the latter to compute the incidence input. Notification-based MI ratios were computed using notification data and estimated CSMR for 2010. For other years, we assumed a similar proportional difference between predicted MI ratios and notifications-based MI ratios as in 2010 and adjusted the predicted MI ratios accordingly, which were then used to compute the incidence input. For South Africa, a country with the highest income inequality in the world, we decided that the Health Care Access and Quality (HAQ) index would be a better health-related index than SDI for TB, a health outcome that differentially affects the poor. We therefore used the HAQ index instead, to compute the incidence input for South Africa. A final incidence estimate that is consistent with prevalence data and cause-specific mortality estimates was then generated using a Bayesian meta-regression.

We computed the age-sex specific incidence of TB among the latent TB-infected population, using TB incidence as the numerator and our estimated risk-weighted LTBI prevalence as the denominator. The method used to estimate LTBI prevalence has been reported in detail elsewhere.[2] We included location-level covariates, namely, the age-standardized adult underweight prevalence, and the log-transformed age-standardized summary exposure variable (SEV) scalar (log transformed) for TB to help inform variation over year and geography with priors that as the scalar increases, prevalence increases. The SEV scalar reflects the exposure to the risk factors related to TB.[3] It was computed using the following formula:

$$\log \textit{SEV scalar} = \log\left(1/\left(1 - \textit{DALY PAF}\right)\right)$$

where *DALY PAF* is age-standardised TB disability-adjusted life-years attributable to TB risk factors for a given location, year, and sex.

Modeling TB prevalence

We used data from prevalence surveys reporting on pulmonary smear-positive TB and bacteriologically positive TB, which were adjusted to account for extra-pulmonary TB. We ran a spatiotemporal Gaussian process regression to predict location-year-age-sex specific proportions of extra-pulmonary TB among all TB cases using notification data. We then computed the extra-pulmonary inflation factor as *1+(proportion of extrapulmonary TB /(1- proportion of extrapulmonary TB))*, and applied it to data from prevalence surveys. We then computed the prevalence of TB among the TB-infected population, using TB prevalence as the numerator and our estimated risk-weighted LTBI prevalence as the denominator. We included a study covariate indicating whether it was bacteriologically positive TB (reference category) or smear-positive TB. We found no statistically significant difference in the data between studies that used both symptoms and chest X-ray as screening methods and studies that used only one of the methods. We therefore did not adjust them for systematic bias but added more uncertainty to data points from studies that used only one of the

screening methods. We also added more uncertainty to data points from sub-national surveys. We included location-level covariates, namely, the age-standardized adult underweight prevalence, and the log-transformed age-standardized SEV scalar for TB to help inform variation over year and geography.

Modeling TB excess mortality

We matched each prevalence data point and TB CSMR (TB and HIV-TB combined) by location, year, age, and sex to calculate excess mortality rate (EMR) as *EMR=CSMR/prevalence*. We also matched each incidence data point and TB CSMR by location, year, age, and sex to calculate EMR for countries with high quality vital registration data. To reflect a gradient in EMR, we added the HAQ index, and adult HIV death rates as country-level covariates.

DisMod-MR 2.1

DisMod MR 2.1 description

For GBD 2016, the computational engine (DisMod-MR 2.1) remained substantively unchanged from GBD 2015. The sequence of estimation occurs at five levels: global, super-region, region, country and, where applicable, subnational location. The super-region priors are generated at the global level with mixed-effects, nonlinear regression using all available data; the super-region fit, in turn, informs the region fit, and so on down the cascade. Subnational estimation was informed by the country fit and country covariates, plus an adjustment based on the average of the residuals between the subnational location's available data and its prior. This mimicked the impact of a random effect on estimates between subnationals. At each level of the cascade, the DisMod-MR 2.1 enforces consistency between all parameters. Analysts have the choice to branch the cascade in terms of time and sex at different levels depending on data density. We used the default option to model TB, which is to branch by sex after the global fit but to retain all years of data until the lowest level in the cascade.

The coefficients for country covariates were re-estimated at each level of the cascade. For a given location, country coefficients were calculated using both data and prior information available for

that location. In GBD 2016, we generated model fits for the years 1990, 1995, 2000, 2005, 2010, and 2016, and log-linearly interpolated estimates for the intervening years. The 95% uncertainty intervals were computed based on 1000 draws from the posterior distribution of the model using the 2.5th and 97.5th percentiles of the ordered 1000 values. We expanded the set of locations where subnational units are modeled in GBD 2016; the set now includes: Brazil, China, England, India, Indonesia, Japan, Kenya, Mexico, Saudi Arabia, South Africa, Sweden, and the United States.

DisMod-MR 2.1 likelihood estimation

Analysts have the choice of using a Gaussian, log-Gaussian, Laplace or Log-Laplace likelihood function in DisMod-MR 2.1. We used the default log-Gaussian equation for the data likelihood, which is:

$$-log\left[p\left(y_j|\Phi\right)\right] = \log\left(\sqrt{2\pi}\right) + \log\left(\delta_j + s_j\right) + \frac{1}{2}\left(\frac{\log\left(a_j + \eta_j\right) - \log\left(m_j + \eta_j\right)}{\delta_j + s_j}\right)^2$$

where, y_j is a ‘measurement value’ (i.e., data point); Φ denotes all model random variables; η_j is the offset value, eta, for a particular ‘integrand’ (prevalence, incidence, remission, excess mortality rate, cause-specific mortality rate) and a_j is the adjusted measurement for data point j, defined by:

$$a_j = e^{(-u_j - c_j)} y_j$$

where u_j is the total ‘area effect’ (i.e., the sum of the random effects at three levels of the cascade: super-region, region and country) and c_j is the total covariate effect (i.e., the mean combined fixed effects for sex, study level and country level covariates), defined by:

$$c_j = \sum_{k=0}^{K[I(j)]-1} \beta_{I(j),k} \hat{X}_{k,j}$$

with standard deviation

$$s_j = \sum_{l=0}^{L[I(j)]-1} \zeta_{I(j),l} \hat{Z}_{k,j}$$

where k denotes the mean value of each data point in relation to a covariate (also called x-covariate); I(j) denotes a data point for a particular integrand, j; $\beta_{I(j),k}$ is the multiplier of the kth x-covariate for the ith integrand; $\hat{X}_{k,j}$ is the covariate value corresponding to the data point j for covariate k; l denotes the standard deviation of each data point in relation to a covariate (also called z-covariate); $\zeta_{I(j),k}$ is the multiplier of the lth z-covariate for the ith integrand; and δ_j is the standard deviation for adjusted measurement j, defined by:

$$\delta_j = log\left[y_j + e^{(-u_j - c_j)}\eta_j + c_j\right] - log\left[y_j + e^{(-u_j - c_j)}\eta_j\right]$$

Where m_j denotes the model for the jth measurement, not counting effects or measurement noise and defined by:

$$m_j = \frac{1}{B(j) - A(j)} \int_{A(j)}^{B(j)} I_j(\mathrm{a})\, \mathrm{da}$$

where A(j) is the lower bound of the age range for a data point; B(j) is the upper bound of the age range for a data point; and I_j denotes the function of age corresponding to the integrand for data point j.

Internally consistent modelling in DisMod-MR 2.1

For each location, we included the following as input in the DisMod model: TB case notifications for locations with a 4 to 5-star rating, predicted MI-ratio-based incidence for locations with a zero to three-star rating, prevalence survey data where available, excess mortality estimates, and CSMR (TB and HIV-TB combined) by age and sex. Beta coefficients and exponentiated values for covariates from the DisMod model are shown in eTable 1.

eTable 1. Beta coefficients and exponentiated values from the DisMod model

Covariate	Parameter	Beta (95% CI)	Exponentiated beta (95% CI)
Smear positive TB	Prevalence	-0.75 (-0.76 — -0.75)	0.47 (0.47 — 0.47)
Sex (male)	Prevalence	0.51 (0.44 — 0.58)	1.66 (1.55 — 1.79)
Sex (male)	Incidence	0.13 (0.13 — 0.13)	1.14 (1.14 — 1.14)
Age-standardized proportion adult underweight	Incidence	2.23 (2.17 — 2.27)	9.35 (8.73 — 9.72)
Age-standardized proportion adult underweight	Prevalence	2.95 (2.85 — 3.00)	19.13 (17.32 — 20.07)

Age-standardized SEV scalar (log-transformed)	Prevalence	0.78 (0.75 — 0.87)	2.19 (2.12 — 2.39)
Age-standardized SEV scalar (log-transformed)	Incidence	0.75 (0.75 — 0.75)	2.12 (2.12 — 2.12)
HAQ (log-transformed)	Excess mortality	-1.58 (-1.64 — -1.52)	0.21 (0.19 — 0.22)
Adult HIV death rate	Excess mortality	0.96 (0.037 — 1.95)	2.61 (1.04 — 7.02)

HAQ=Healthcare access and quality index; SEV=Summary Exposure Variable

The output from the DisMod model was for all forms of TB in TB-infected population including both HIV-negative and HIV-positive individuals. We computed the incidence and prevalence of TB among the entire population, by multiplying the prevalence of LTBI with the DisMod model estimates.

HIV-TB incidence and prevalence

To distinguish HIV-TB from all forms of TB, we first estimated the proportions of HIV-TB cases among all TB cases using data on the number of TB cases recorded as HIV-positive and the number of TB cases with an HIV test result recorded in the WHO TB notifications register. We ran a mixed effects regression using the adult HIV death rate as a covariate to predict location-year specific HIV-TB proportions, which were then applied to TB incident and prevalent cases from DisMod, to generate HIV-TB incident and prevalent cases by location and year. These cases were then age-sex split based on the age-sex pattern of estimated HIV prevalence by location-year to generate location-year-age-sex specific HIV-TB incident and prevalent cases.

MDR-TB, XDR-TB and drug-susceptible TB among HIV negative individuals

Input data for MDR-TB and XDR-TB include: (i) the number of drug-resistant cases by type [MDR-TB, XDR-TB, TB cases with a drug sensitivity testing (DST) result for isoniazid and rifampicin, and MDR-TB cases with DST for second-line drugs] from routine surveillance and surveys reported to the World Health Organization (eFigure 2), and (ii) the risk of MDR-TB associated with HIV infection from a meta-analysis[4].

We ran a spatiotemporal Gaussian process regression to predict the proportions of MDR-TB cases among all TB cases for all locations and years. The input data for this regression (i.e., weighted

average of the proportions of new and previously treated cases with MDR-TB) were based on the number of MDR-TB cases among new TB cases, MDR-TB cases among previously treated TB cases, and the number of new and previously treated TB cases with drug sensitivity testing for isoniazid and rifampicin from routine surveillance and surveys reported to the World Health Organization. We then used the predicted proportions to MDR-TB cases among all TB cases, along with the HIV-TB and TB no-HIV incidence estimates, and the relative risk of MDR-TB associated with HIV infection from a meta-analysis[4] to compute the proportions of MDR-TB cases among HIV negative TB cases $(PnoHIV_{c,y,a,s})$ by location, year, age, and sex using the following formula:

$$PnoHIV_{c,y,a,s} = \frac{MDR_{c,y}}{\left(1 + \left(RR\,\frac{HIVTB_{c,y,a,s}}{TBnoHIV_{c,y,a,s}}\right)\right) TBnoHIV_{c,y,a,s}}$$

where $MDR_{c,y}$ is the number of all MDR-TB cases among HIV-positive and HIV-negative individuals by location and year, RR is the relative risk of MDR-TB associated with HIV infection, $HIVTB_{c,y,a,s}$ is the number of HIV-TB incident cases by location, year, age, and sex, and $TBnoHIV_{c,y,a,s}$ is the number of TB no-HIV incident cases by location, year, age, and sex.

We applied the predicted proportions of MDR-TB cases among HIV negative TB cases to our predicted HIV-negative TB incident and prevalent cases to generate MDR-TB incident and prevalent cases by location, year, age, and sex. Next, we subtracted MDR-TB cases from all HIV-negative TB cases to generate drug-susceptible TB cases by location, year, age, and sex. To distinguish XDR-TB from MDR-TB, we aggregated the XDR-TB cases and MDR-TB cases (with drug sensitivity testing for second-line drugs) up to the super-region level and calculated the super-region level proportions of XDR-TB among MDR-TB cases, which were then applied to MDR-TB cases in corresponding countries within the super-regions to produce XDR-TB cases by location, year, age, and sex. We

linearly extrapolated XDR-TB prevalence and incidence back assuming the rates were zero in 1992, one year before 1993 when XDR-TB was first recorded in USA surveillance data[5]. Finally, we subtracted XDR-TB cases from MDR-TB cases to generate MDR-TB (without XDR) cases by location, year, age, and sex.

MDR HIV-TB, XDR HIV-TB and drug-susceptible HIV-TB

To split HIV-TB into MDR-HIV-TB and drug-susceptible HIV-TB, we first calculated the proportions of MDR-HIV-TB among all HIV-TB cases ($PHIV_{c,y,a,s}$) for each location, year, age, and sex using the following formula:

$$PHIV_{c,y,a,s} = PnoHIV_{c,y,a,s}RR$$

where $PnoHIV_{c,y,a,s}$ is the proportions of MDR-TB among all HIV-negative TB cases for each location, year, age, and sex and RR is the relative risk of MDR-TB associated with HIV infection. We then applied the predicted proportions of MDR-TB cases among HIV-TB cases to all HIV-TB case estimates to generate MDR-HIV-TB cases by location, year, age, and sex. Next, we subtracted MDR-HIV-TB cases from all HIV-TB cases to generate drug-susceptible HIV-TB cases by location, year, age, and sex. The remaining steps (separating XDR- HIV-TB from MDR-HIV-TB, extrapolation, and subtraction) to generate MDR-HIV-TB (without extensive drug resistance) cases by location, year, age, and sex are the same as those for generating HIV-negative MDR-TB and XDR-TB.

eFigure 2. Data availability (site-years) by GBD 2016 location for MDR-TB (A) and XDR-TB (B)

A

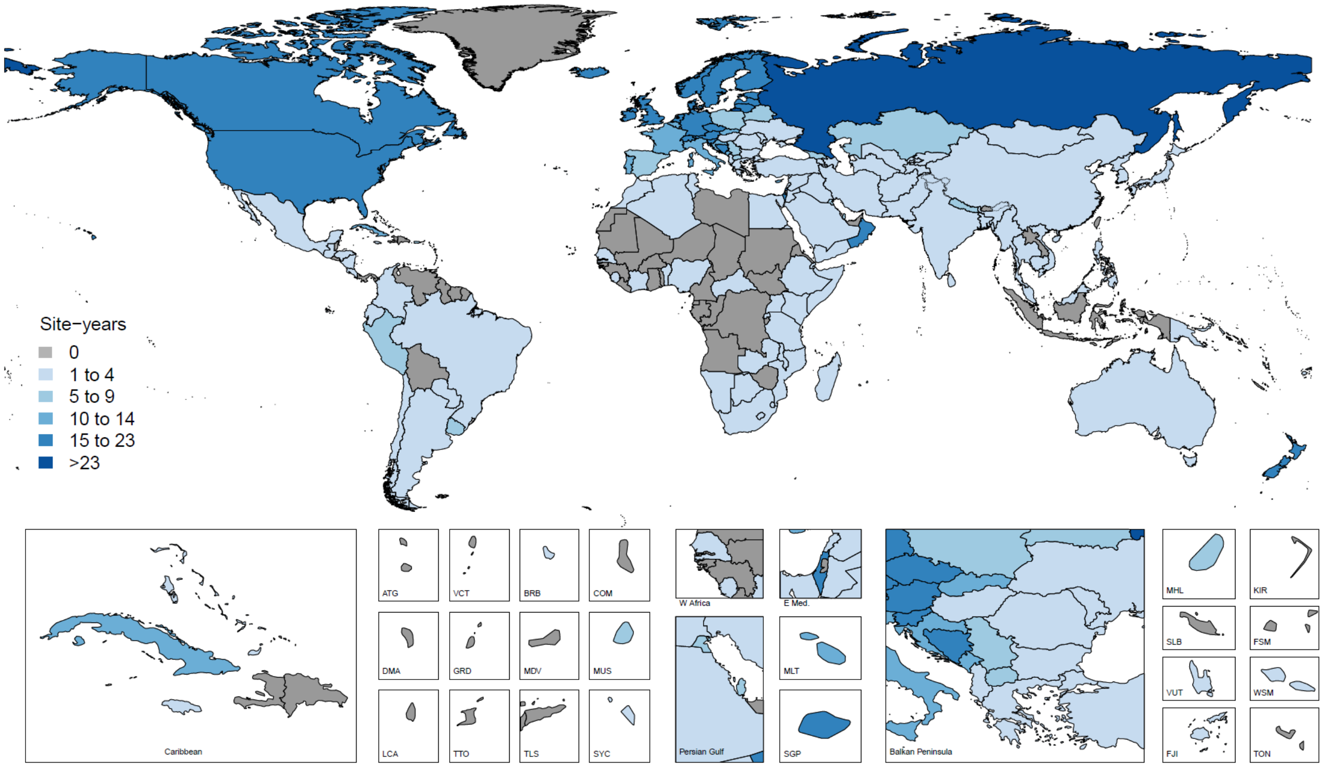

B

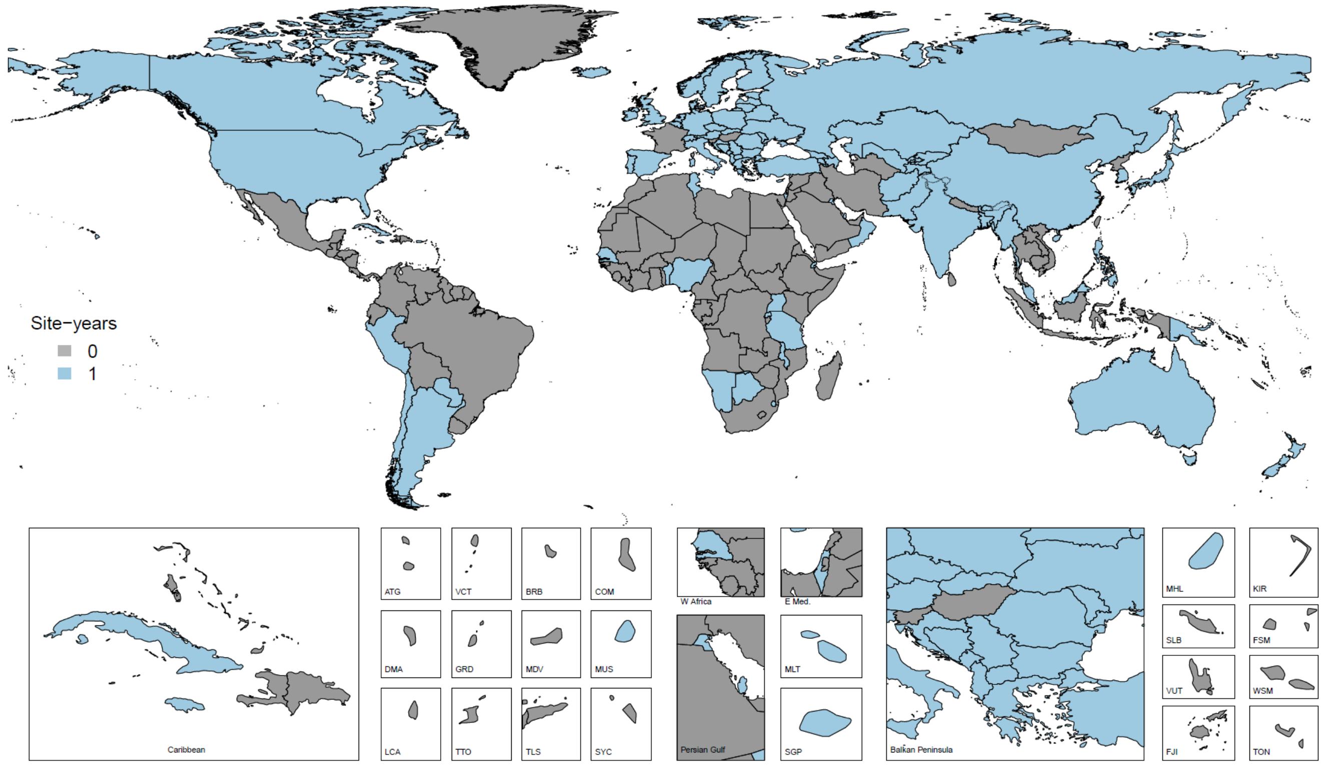

eFigure 2 Footnote: ATG=Antigua and Barbuda. BRB=Barbados. COM=Comoros. DMA=Dominica. E Med=Eastern Mediterranean. FJI=Fiji. FSM=Federated States of Micronesia. GRD=Grenada. KIR=Kiribati. LCA=Saint Lucia. MDV=Maldives. MHL=Marshall Islands. MLT=Malta. MUS=Mauritius. SGP=Singapore. SLB=Solomon Islands. SYC=Seychelles. TLS=Timor-Leste.

Fatal tuberculosis among HIV-negative individuals

Flowchart

eFigure 3. Tuberculosis mortality: input data, analytical process, and output

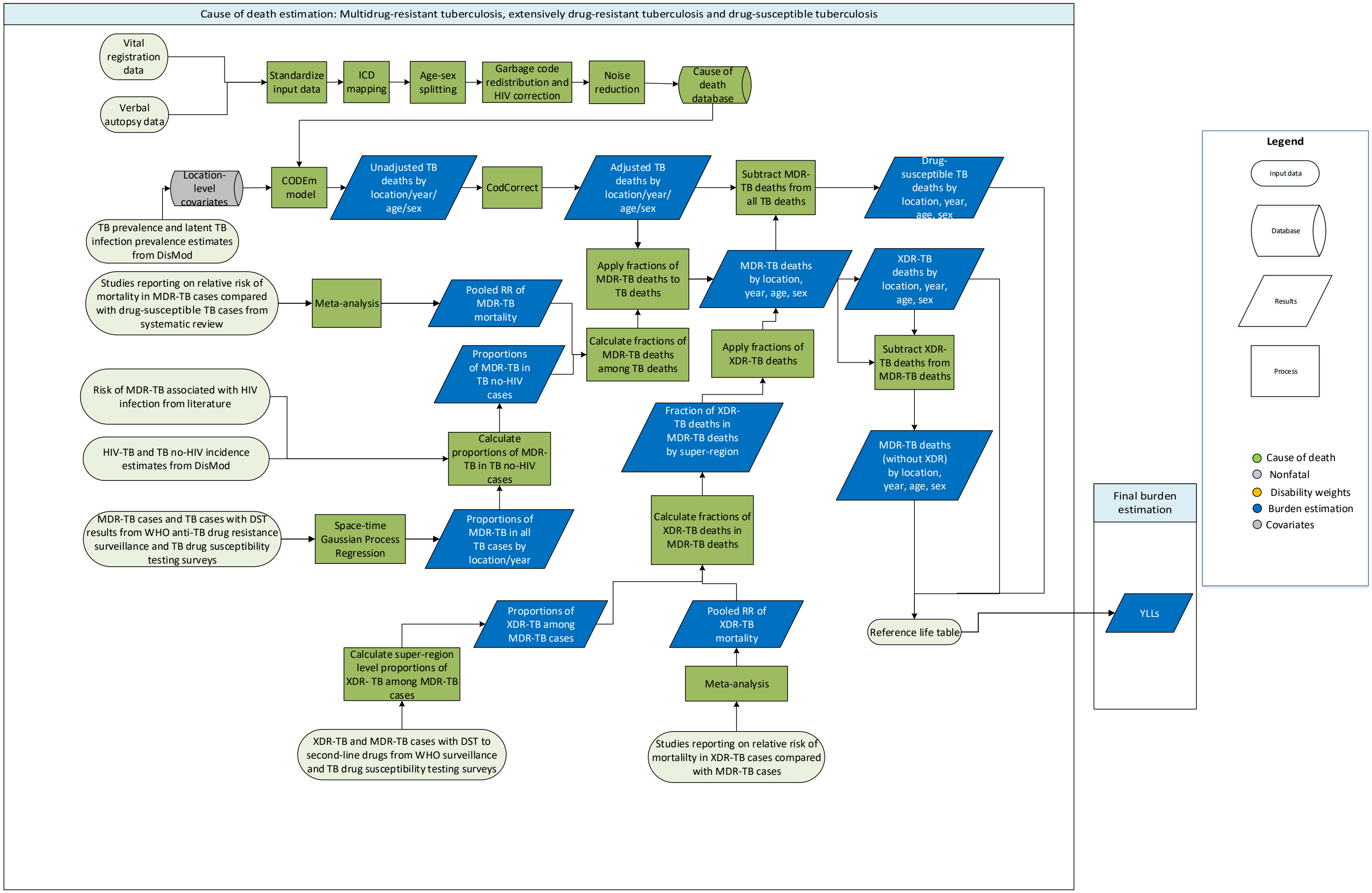

Mortality from TB among HIV-negative individuals

Input data for modelling TB mortality among HIV-negative individuals include 15,943 site-years of vital registration data, 1,710 site-years of verbal autopsy data, 764 site-years of sample-based vital registration data, and 361 site-years of mortality surveillance data. The quality and comparability of the cause of death data were assessed and enhanced through multiple steps,[6] including redistribution of garbage codes to underlying causes of death following GBD algorithms and adjustment for misclassified HIV deaths (ie, HIV deaths being assigned to other underlying causes of death such as TB because of stigma or misdiagnosis). GBD 2016 also assessed the overall data quality for each country (based on completeness, garbage coding, cause list detail, and time periods covered), and assigned a quality score ranging from 0 stars (poorest) to 5 stars (best); a quality score of 4 to 5 is considered high quality (eFigure 4). Verbal autopsy data in countries with high HIV prevalence (using an arbitrary cutoff value of 5% age-standardised HIV prevalence) were removed because verbal autopsy studies have a poor ability to distinguish HIV deaths from HIV-TB deaths.

eFigure 4. Overall data quality by country

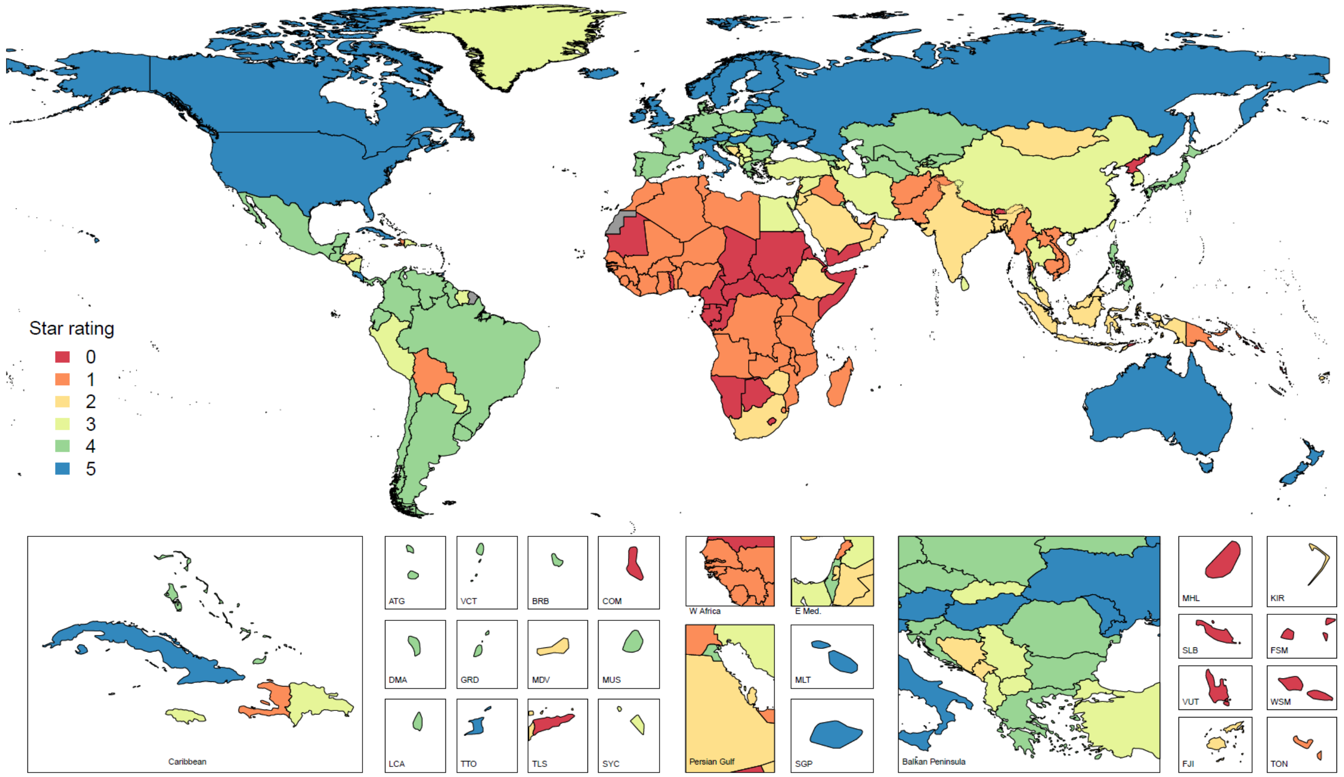

ATG=Antigua and Barbuda. BRB=Barbados. COM=Comoros. DMA=Dominica. E Med=Eastern Mediterranean. FJI=Fiji. FSM=Federated States of Micronesia. GRD=Grenada. KIR=Kiribati. LCA=Saint Lucia. MDV=Maldives. MHL=Marshall Islands. MLT=Malta. MUS=Mauritius. SGP=Singapore. SLB=Solomon Islands. SYC=Seychelles. TLS=Timor-Leste.

The Cause of Death Ensemble modelling (CODEm)

TB mortality trends among HIV-negative individuals was modelled using the Cause of Death Ensemble modelling (CODEm) strategy,[7] which is based on five general principles: identifying all available data, enhancing the comparability and quality of the dataset, developing a diverse set of possible models, assessing the predictive validity of all models, and selecting the models with the best performance in out-of-sample predictive validity tests. Possible models were identified using a covariate selection algorithm that yielded many plausible combinations of covariates which were then run through four classes of models. These model classes include modeling natural log rates and logit cause fractions using mixed effects linear models and spatiotemporal Gaussian Process Regression models. This generated a large variety of models that competed in predictive validity tests. An ensemble of CODEm models that performed best on out-of-sample predictive validity tests was then selected. We explain in detail below how the candidate models were developed and evaluated as well as how the best ensemble model was selected.

We first identified a set of plausible covariates for tuberculosis based on the published literature. We categorized these covariates into three groups based on the strength of epidemiological evidence: 1) level 1 covariates for which there is convincing or probable evidence for a causal relationship, 2) level 2 covariates for which there is strong evidence for a relationship but inadequate evidence of a causal effect, and 3) level 3 covariates where there is general correlation evidence for a relationship as observed in previous time series or cross-sectional studies. The candidate covariates, priors, and levels for the CODEm model are shown in eTable 2.

eTable 2: Covariates used in CODEm for tuberculosis

	Covariate	Direction
Level 1	Summary Exposure Variable (SEV) scalar<br>Alcohol per capita<br>Smoking prevalence<br>Fasting plasma glucose<br>Latent tuberculosis infection<br>Tuberculosis prevalence	+<br>+<br>+<br>+<br>+<br>+
Level 2	Adult underweight proportion<br>Indoor air pollution<br>Outdoor air pollution<br>Population density<br>Health Care Access and Quality index	+<br>+<br>+<br>+<br>-
Level 3	Lag-distributed income<br>Education (years per capita)<br>Sociodemographic Index	-<br>-<br>-

In the first step, we ran models for all possible combinations of level 1 covariates. Separate models were run for males and females. We ran models where the dependent variable was the rate in logarithmic scale by age and models where the dependent variable was logit cause fraction by age. For each type of model, we assessed a list of 2^n possible combinations of level 1 covariates (where n is the number of level 1 covariates). Models were retained if all covariates in the model were in the expected direction and the coefficients were significant at the $p<0{\cdot}05$ level. For each level 1 model that was retained, we generated a list of 2^m plausible level 2 models (where m is the number of level 2 covariates), and tested each of the m plausible models (in which one level 2 covariate was added to the retained level 1 model). If the addition of the level 2 covariate did not change the direction or statistical significance of level 1 coefficients, the model was retained. However, if a level 2 covariate did not fit the priors on the direction and statistical significance of level 1 covariates, all possible level 2 models that included that covariate were dropped. Next, we took each of the retained level 2 models and repeated the same process for the level 3 covariates. Ultimately, we obtained a total pool of 308 and 278 component models that fulfilled the priors on covariate direction for males and females respectively. Uncertainty intervals for predicted death rates were generated by sampling

the posterior distribution of each of the component models in proportion to the weight of each model in the ensemble; mixed effects component model uncertainty includes uncertainty in the betas and the hierarchical random effects; spatiotemporal Gaussian Process Regression component models include uncertainty from the mean function prior and the data variance.

The ability of each of these models to make accurate predictions was formally evaluated by creating train-test-test splits. For each of these datasets, we randomly assigned 70% of the data to the train set, 15% to the test 1 set, and the remaining 15% to test 2. The assignment of the data to train and test was implemented so that the pattern of holding out the data for the test datasets mimics the pattern of missingness in the full dataset. For each train dataset, we re-estimated each of the proposed models including both the mixed effects model and the spatial-temporal model. We used the results of the models estimated on the training data alone to predict for the first test set. The test data have not been included in the model estimation; the performance of each model was therefore being evaluated out-of-sample. In this way, the out-of-sample predictions for the test set are a fair test of how each model will perform for tuberculosis mortality where the data are sparse or missing.

Predictive validity was evaluated using three metrics. First, we evaluated how well each model predicted age-specific death rates using the root mean squared error (RMSE) of the natural log of the death rate. Second, to evaluate whether predicted trends matched data trends, we computed the log death rate in year t minus the log death rate in year t-1 for the test data. We also computed the same metric for the prediction. We then counted the percentage of cases for which the model predicted a trend in the same direction as the test data. We ranked each component model on the median of these two metrics across hold-outs (lower being better for RMSE, higher being better for the trend test). We then summed up the ranks across the two metrics for each component model, and assigned overall rank 1 to the model with the smallest sum of ranks, rank N to the model with the highest sum of ranks, etc. These ranks were then used in the ensemble weighting equation described below.

$$W_i = \frac{\psi^{(N-rank_i)}}{\sum_{j=1}^{N}\left(\psi^{(N-j)}\right)}$$

where N is the number of models and ψ is a para-meter influencing the relative weighting of models. We used the weights to determine how many draws from each model to add to the final pool. We developed and tested ensembles that include ψ values from 1.0 to 1.2 in intervals of .01. We then compared these different ensemble models using predictive validity performance on the second set of test data. This enabled us to choose an ensemble model that weighted the component models adaptively. The ranking, weights, and approximate number of draws contributed by each of the top 32 submodels are shown in eTable 3 (pages 24-32).

Finally, an important property of the models is that they generate plausible prediction intervals. We therefore computed the percent of data in the test set that were included in the 95% prediction interval. The prediction interval was based both on the uncertainty in the predicted death rate and the data variance for each observation. Both male and female models had data coverage greater than 95%.

Mortality from MDR-TB, XDR-TB, and drug-susceptible TB among HIV-negative individuals

Input data include: (i) estimated proportions of MDR-TB and XDR-TB from our non-fatal TB modeling, (ii) the relative risk of death in MDR-TB cases compared with drug- susceptible TB cases and the relative risk of death in XDR-TB cases compared with MDR-TB cases from our systematic review and meta-analysis (see page 36). Appendix eFigure 3 shows the input data, analytical process, and output from this analysis.

We computed the fraction of MDR-TB deaths among all HIV-negative TB deaths ($D_{c,y,a,s}$) using the following formula:

$$D_{c,y,a,s} = \frac{P_{c,y,a,s}RR}{P_{c,y,a,s}RR + 1 - P_{c,y,a,s}}$$

where $P_{c,y,a,s}$ is the estimated proportions of MDR-TB cases among HIV negative TB cases, and RR is the relative risk of death in MDR-TB cases compared with drug- susceptible TB cases. We then applied the predicted fractions of MDR-TB deaths among HIV-negative TB deaths to our CODEm TB death estimates to generate MDR-TB deaths by location, year, age, and sex. Next, we subtracted MDR-TB deaths from all TB deaths to generate drug- susceptible TB deaths by location, year, age, and sex.

We computed the super-region-year-specific fractions of XDR-TB deaths among all MDR-TB deaths ($D_{XDRsr,y}$) using the following formula:

$$D_{XDRsr,y} = \frac{P_{XDRsr,y} RR_{XDR}}{P_{XDRsr,y} RR_{XDR} + 1 - P_{XDRsr,y}}$$

where $P_{XDRsr,y}$ is the super-region level proportion of XDR-TB among MDR-TB cases, and RR_{XDR} is the pooled relative risk of mortality in XDR-TB cases compared with MDR-TB cases. These fractions were then applied to MDR-TB deaths in corresponding countries within the super-regions to produce XDR-TB deaths by location, year, age, and sex. Similar to our estimation for non-fatal XDR-TB, we linearly extrapolated XDR-TB mortality rates back assuming the mortality rates were zero in 1992 and earlier years. Finally, we subtracted XDR-TB deaths from MDR-TB deaths to generate MDR-TB (without extensive drug resistance) deaths by location, year, age, and sex.

eTable 3. Top 32 ranking CODEm sub-models for tuberculosis by sex

Females							Males					
Covariate name	Rank	Weight	Submodel type	Cause of death data type	Number of draws		Covariate name	Rank	Weight	Submodel type	Cause of death data type	Number of draws
Tuberculosis infection risk-weighted prevalence (age-standardized)	1	0.166667	spacetime	cf	167		Tuberculosis prevalence (age-standardized)	1	0.166667	spacetime	cf	167
Tuberculosis prevalence (age-standardized)	1	0.166667	spacetime	cf	167		Healthcare access and quality index	1	0.166667	spacetime	cf	167
Age-standardized proportion adult underweight	1	0.166667	spacetime	cf	167		Population Density (over 1000 ppl/sqkm, proportion)	1	0.166667	spacetime	cf	167
Healthcare access and quality index	1	0.166667	spacetime	cf	167		Tuberculosis prevalence (age-standardized)	2	0.138889	spacetime	cf	139
Tuberculosis infection risk-weighted prevalence (age-standardized)	2	0.138889	spacetime	cf	139		Alcohol (liters per capita)	2	0.138889	spacetime	cf	139
Healthcare access and quality index	2	0.138889	spacetime	cf	139		Healthcare access and quality index	2	0.138889	spacetime	cf	139
Tuberculosis infection risk-weighted prevalence (age-standardized)	3	0.115741	spacetime	cf	116		Population Density (over 1000 ppl/sqkm, proportion)	2	0.138889	spacetime	cf	139
Tuberculosis prevalence (age-standardized)	3	0.115741	spacetime	cf	116		Smoking Prevalence	2	0.138889	spacetime	cf	139
Age-standardized proportion adult underweight	3	0.115741	spacetime	cf	116		Tuberculosis infection risk-weighted prevalence (age-standardized)	3	0.115741	spacetime	cf	116
Alcohol (liters per capita)	3	0.115741	spacetime	cf	116		Tuberculosis prevalence (age-standardized)	3	0.115741	spacetime	cf	116
Healthcare access and quality index	3	0.115741	spacetime	cf	116		Age-standardized proportion adult underweight	3	0.115741	spacetime	cf	116
Tuberculosis prevalence (age-standardized)	4	0.096451	spacetime	cf	96		Alcohol (liters per capita)	3	0.115741	spacetime	cf	116

Females							Males					
**Covariate name**	**Rank**	**Weight**	**Submodel type**	**Cause of death data type**	**Number of draws**		**Covariate name**	**Rank**	**Weight**	**Submodel type**	**Cause of death data type**	**Number of draws**
Age-standardized proportion adult underweight	4	0.096451	spacetime	cf	96		Healthcare access and quality index	3	0.115741	spacetime	cf	116
Healthcare access and quality index	4	0.096451	spacetime	cf	96		Population Density (over 1000 ppl/sqkm, proportion)	3	0.115741	spacetime	cf	116
Indoor Air Pollution (All Cooking Fuels)	4	0.096451	spacetime	cf	96		Tuberculosis infection risk-weighted prevalence (age-standardized)	4	0.096451	spacetime	cf	96
Population Density (over 1000 ppl/sqkm, proportion)	4	0.096451	spacetime	cf	96		Tuberculosis prevalence (age-standardized)	4	0.096451	spacetime	cf	96
Tuberculosis infection risk-weighted prevalence (age-standardized)	5	0.080376	spacetime	rate	80		Healthcare access and quality index	4	0.096451	spacetime	cf	96
Tuberculosis prevalence (age-standardized)	5	0.080376	spacetime	rate	80		Population Density (over 1000 ppl/sqkm, proportion)	4	0.096451	spacetime	cf	96
Age-standardized proportion adult underweight	5	0.080376	spacetime	rate	80		Tuberculosis infection risk-weighted prevalence (age-standardized)	5	0.080376	spacetime	cf	80
Alcohol (liters per capita)	5	0.080376	spacetime	rate	80		Tuberculosis prevalence (age-standardized)	5	0.080376	spacetime	cf	80
Healthcare access and quality index	5	0.080376	spacetime	rate	80		Alcohol (liters per capita)	5	0.080376	spacetime	cf	80
Log-transformed SEV scalar: TB	6	0.06698	spacetime	rate	67		Healthcare access and quality index	5	0.080376	spacetime	cf	80
Tuberculosis infection risk-weighted prevalence (age-standardized)	6	0.06698	spacetime	rate	67		Indoor Air Pollution (All Cooking Fuels)	5	0.080376	spacetime	cf	80
Tuberculosis prevalence (age-standardized)	6	0.06698	spacetime	rate	67		Population Density (over 1000 ppl/sqkm, proportion)	5	0.080376	spacetime	cf	80
Age-standardized proportion adult underweight	6	0.06698	spacetime	rate	67		Smoking Prevalence	5	0.080376	spacetime	cf	80
Healthcare access and quality index	6	0.06698	spacetime	rate	67		Log-transformed SEV scalar: TB	6	0.06698	spacetime	cf	67
Tuberculosis infection risk-weighted prevalence (age-standardized)	7	0.055816	spacetime	rate	56		Tuberculosis infection risk-weighted prevalence (age-standardized)	6	0.06698	spacetime	cf	67
Tuberculosis prevalence (age-standardized)	7	0.055816	spacetime	rate	56		Tuberculosis prevalence (age-standardized)	6	0.06698	spacetime	cf	67

Females							Males					
Covariate name	Rank	Weight	Submodel type	Cause of death data type	Number of draws		Covariate name	Rank	Weight	Submodel type	Cause of death data type	Number of draws
Age-standardized proportion adult underweight	7	0.055816	spacetime	rate	56		Age-standardized proportion adult underweight	6	0.06698	spacetime	cf	67
Healthcare access and quality index	7	0.055816	spacetime	rate	56		Alcohol (liters per capita)	6	0.06698	spacetime	cf	67
Log-transformed SEV scalar: TB	8	0.046514	spacetime	rate	47		Healthcare access and quality index	6	0.06698	spacetime	cf	67
Tuberculosis prevalence (age-standardized)	8	0.046514	spacetime	rate	47		Tuberculosis prevalence (age-standardized)	7	0.055816	spacetime	cf	56
Healthcare access and quality index	8	0.046514	spacetime	rate	47		Alcohol (liters per capita)	7	0.055816	spacetime	cf	56
Tuberculosis infection risk-weighted prevalence (age-standardized)	9	0.038761	spacetime	cf	39		Healthcare access and quality index	7	0.055816	spacetime	cf	56
Tuberculosis prevalence (age-standardized)	9	0.038761	spacetime	cf	39		Log-transformed SEV scalar: TB	8	0.046514	spacetime	cf	47
Education (years per capita)	9	0.038761	spacetime	cf	39		Tuberculosis infection risk-weighted prevalence (age-standardized)	8	0.046514	spacetime	cf	47
Socio-demographic Index	9	0.038761	spacetime	cf	39		Tuberculosis prevalence (age-standardized)	8	0.046514	spacetime	cf	47
Tuberculosis infection risk-weighted prevalence (age-standardized)	10	0.032301	spacetime	cf	32		Healthcare access and quality index	8	0.046514	spacetime	cf	47
Tuberculosis prevalence (age-standardized)	10	0.032301	spacetime	cf	32		Log-transformed SEV scalar: TB	9	0.038761	spacetime	cf	39
Indoor Air Pollution (All Cooking Fuels)	10	0.032301	spacetime	cf	32		Tuberculosis prevalence (age-standardized)	9	0.038761	spacetime	cf	39
Socio-demographic Index	10	0.032301	spacetime	cf	32		Healthcare access and quality index	9	0.038761	spacetime	cf	39
Smoking Prevalence	10	0.032301	spacetime	cf	32		Log-transformed SEV scalar: TB	10	0.032301	spacetime	cf	32
Tuberculosis infection risk-weighted prevalence (age-standardized)	11	0.026918	spacetime	cf	27		Tuberculosis infection risk-weighted prevalence (age-standardized)	10	0.032301	spacetime	cf	32
Tuberculosis prevalence (age-standardized)	11	0.026918	spacetime	cf	27		Age-standardized proportion adult underweight	10	0.032301	spacetime	cf	32
Education (years per capita)	11	0.026918	spacetime	cf	27		Alcohol (liters per capita)	10	0.032301	spacetime	cf	32
Socio-demographic Index	11	0.026918	spacetime	cf	27		Healthcare access and quality index	10	0.032301	spacetime	cf	32

Females							Males					
Covariate name	Rank	Weight	Submodel type	Cause of death data type	Number of draws		Covariate name	Rank	Weight	Submodel type	Cause of death data type	Number of draws
Smoking Prevalence	11	0.026918	spacetime	cf	27		Log-transformed SEV scalar: TB	11	0.026918	spacetime	cf	27
Tuberculosis infection risk-weighted prevalence (age-standardized)	12	0.022431	spacetime	rate	22		Alcohol (liters per capita)	11	0.026918	spacetime	cf	27
Age-standardized proportion adult underweight	12	0.022431	spacetime	rate	22		Healthcare access and quality index	11	0.026918	spacetime	cf	27
Healthcare access and quality index	12	0.022431	spacetime	rate	22		Tuberculosis infection risk-weighted prevalence (age-standardized)	12	0.022431	spacetime	cf	22
Indoor Air Pollution (All Cooking Fuels)	12	0.022431	spacetime	rate	22		Alcohol (liters per capita)	12	0.022431	spacetime	cf	22
Tuberculosis infection risk-weighted prevalence (age-standardized)	13	0.018693	spacetime	cf	19		Healthcare access and quality index	12	0.022431	spacetime	cf	22
Tuberculosis prevalence (age-standardized)	13	0.018693	spacetime	cf	19		Tuberculosis prevalence (age-standardized)	13	0.018693	spacetime	cf	19
Alcohol (liters per capita)	13	0.018693	spacetime	cf	19		Alcohol (liters per capita)	13	0.018693	spacetime	cf	19
Education (years per capita)	13	0.018693	spacetime	cf	19		Education (years per capita)	13	0.018693	spacetime	cf	19
Socio-demographic Index	13	0.018693	spacetime	cf	19		Socio-demographic Index	13	0.018693	spacetime	cf	19
Tuberculosis infection risk-weighted prevalence (age-standardized)	14	0.015577	spacetime	cf	16		Smoking Prevalence	13	0.018693	spacetime	cf	19
Tuberculosis prevalence (age-standardized)	14	0.015577	spacetime	cf	16		Tuberculosis infection risk-weighted prevalence (age-standardized)	14	0.015577	spacetime	cf	16
Age-standardized proportion adult underweight	14	0.015577	spacetime	cf	16		Tuberculosis prevalence (age-standardized)	14	0.015577	spacetime	cf	16
Alcohol (liters per capita)	14	0.015577	spacetime	cf	16		Alcohol (liters per capita)	14	0.015577	spacetime	cf	16
Education (years per capita)	14	0.015577	spacetime	cf	16		Education (years per capita)	14	0.015577	spacetime	cf	16
Indoor Air Pollution (All Cooking Fuels)	14	0.015577	spacetime	cf	16		Socio-demographic Index	14	0.015577	spacetime	cf	16
Socio-demographic Index	14	0.015577	spacetime	cf	16		Smoking Prevalence	14	0.015577	spacetime	cf	16
Tuberculosis infection risk-weighted prevalence (age-standardized)	15	0.012981	spacetime	cf	13		Tuberculosis infection risk-weighted prevalence (age-standardized)	15	0.012981	spacetime	rate	13
Tuberculosis prevalence (age-standardized)	15	0.012981	spacetime	cf	13		Tuberculosis prevalence (age-standardized)	15	0.012981	spacetime	rate	13

Females							Males					
Covariate name	Rank	Weight	Submodel type	Cause of death data type	Number of draws		Covariate name	Rank	Weight	Submodel type	Cause of death data type	Number of draws
Age-standardized proportion adult underweight	15	0.012981	spacetime	cf	13		Alcohol (liters per capita)	15	0.012981	spacetime	rate	13
Alcohol (liters per capita)	15	0.012981	spacetime	cf	13		Healthcare access and quality index	15	0.012981	spacetime	rate	13
Education (years per capita)	15	0.012981	spacetime	cf	13		Tuberculosis infection risk-weighted prevalence (age-standardized)	16	0.010818	spacetime	cf	11
Indoor Air Pollution (All Cooking Fuels)	15	0.012981	spacetime	cf	13		Tuberculosis prevalence (age-standardized)	16	0.010818	spacetime	cf	11
Socio-demographic Index	15	0.012981	spacetime	cf	13		Age-standardized proportion adult underweight	16	0.010818	spacetime	cf	11
Smoking Prevalence	15	0.012981	spacetime	cf	13		Education (years per capita)	16	0.010818	spacetime	cf	11
Tuberculosis infection risk-weighted prevalence (age-standardized)	16	0.010818	spacetime	cf	11		Socio-demographic Index	16	0.010818	spacetime	cf	11
Tuberculosis prevalence (age-standardized)	16	0.010818	spacetime	cf	11		Smoking Prevalence	16	0.010818	spacetime	cf	11
Age-standardized proportion adult underweight	16	0.010818	spacetime	cf	11		Tuberculosis infection risk-weighted prevalence (age-standardized)	17	0.009015	spacetime	cf	9
Education (years per capita)	16	0.010818	spacetime	cf	11		Tuberculosis prevalence (age-standardized)	17	0.009015	spacetime	cf	9
Socio-demographic Index	16	0.010818	spacetime	cf	11		Education (years per capita)	17	0.009015	spacetime	cf	9
Smoking Prevalence	16	0.010818	spacetime	cf	11		Indoor Air Pollution (All Cooking Fuels)	17	0.009015	spacetime	cf	9
Socio-demographic Index	17	0.009015	spacetime	cf	9		Socio-demographic Index	17	0.009015	spacetime	cf	9
Smoking Prevalence	17	0.009015	spacetime	cf	9		Smoking Prevalence	17	0.009015	spacetime	cf	9
Tuberculosis prevalence (age-standardized)	18	0.007512	spacetime	cf	8		Tuberculosis infection risk-weighted prevalence (age-standardized)	18	0.007512	spacetime	rate	8
Age-standardized proportion adult underweight	18	0.007512	spacetime	cf	8		Tuberculosis prevalence (age-standardized)	18	0.007512	spacetime	rate	8
Indoor Air Pollution (All Cooking Fuels)	18	0.007512	spacetime	cf	8		Alcohol (liters per capita)	18	0.007512	spacetime	rate	8
Socio-demographic Index	18	0.007512	spacetime	cf	8		Education (years per capita)	18	0.007512	spacetime	rate	8
Smoking Prevalence	18	0.007512	spacetime	cf	8		Healthcare access and quality index	18	0.007512	spacetime	rate	8

Females							Males					
Covariate name	Rank	Weight	Submodel type	Cause of death data type	Number of draws		Covariate name	Rank	Weight	Submodel type	Cause of death data type	Number of draws
Tuberculosis prevalence (age-standardized)	19	0.00626	spacetime	cf	6		Log-transformed SEV scalar: TB	19	0.00626	spacetime	rate	6
Age-standardized proportion adult underweight	19	0.00626	spacetime	cf	6		Tuberculosis prevalence (age-standardized)	19	0.00626	spacetime	rate	6
Alcohol (liters per capita)	19	0.00626	spacetime	cf	6		Alcohol (liters per capita)	19	0.00626	spacetime	rate	6
Education (years per capita)	19	0.00626	spacetime	cf	6		Healthcare access and quality index	19	0.00626	spacetime	rate	6
Socio-demographic Index	19	0.00626	spacetime	cf	6		Tuberculosis prevalence (age-standardized)	20	0.005217	spacetime	rate	5
Smoking Prevalence	19	0.00626	spacetime	cf	6		Alcohol (liters per capita)	20	0.005217	spacetime	rate	5
Age-standardized proportion adult underweight	20	0.005217	spacetime	cf	5		Healthcare access and quality index	20	0.005217	spacetime	rate	5
Indoor Air Pollution (All Cooking Fuels)	20	0.005217	spacetime	cf	5		Population Density (over 1000 ppl/sqkm, proportion)	20	0.005217	spacetime	rate	5
Socio-demographic Index	20	0.005217	spacetime	cf	5		Log-transformed SEV scalar: TB	21	0.004347	spacetime	cf	4
Smoking Prevalence	20	0.005217	spacetime	cf	5		Tuberculosis infection risk-weighted prevalence (age-standardized)	21	0.004347	spacetime	cf	4
Tuberculosis prevalence (age-standardized)	21	0.004347	spacetime	cf	4		Tuberculosis prevalence (age-standardized)	21	0.004347	spacetime	cf	4
Age-standardized proportion adult underweight	21	0.004347	spacetime	cf	4		Alcohol (liters per capita)	21	0.004347	spacetime	cf	4
Alcohol (liters per capita)	21	0.004347	spacetime	cf	4		Education (years per capita)	21	0.004347	spacetime	cf	4
Education (years per capita)	21	0.004347	spacetime	cf	4		Socio-demographic Index	21	0.004347	spacetime	cf	4
Indoor Air Pollution (All Cooking Fuels)	21	0.004347	spacetime	cf	4		Tuberculosis prevalence (age-standardized)	22	0.003623	spacetime	cf	4
Socio-demographic Index	21	0.004347	spacetime	cf	4		Alcohol (liters per capita)	22	0.003623	spacetime	cf	4
Tuberculosis prevalence (age-standardized)	22	0.003623	spacetime	cf	4		Education (years per capita)	22	0.003623	spacetime	cf	4
Alcohol (liters per capita)	22	0.003623	spacetime	cf	4		Socio-demographic Index	22	0.003623	spacetime	cf	4
Education (years per capita)	22	0.003623	spacetime	cf	4		Log-transformed SEV scalar: TB	23	0.003019	spacetime	rate	3

**Females**							**Males**					
**Covariate name**	**Rank**	**Weight**	**Submodel type**	**Cause of death data type**	**Number of draws**		**Covariate name**	**Rank**	**Weight**	**Submodel type**	**Cause of death data type**	**Number of draws**
Socio-demographic Index	22	0.003623	spacetime	cf	4		Tuberculosis prevalence (age-standardized)	23	0.003019	spacetime	rate	3
Tuberculosis prevalence (age-standardized)	23	0.003019	spacetime	cf	3		Alcohol (liters per capita)	23	0.003019	spacetime	rate	3
Education (years per capita)	23	0.003019	spacetime	cf	3		Education (years per capita)	23	0.003019	spacetime	rate	3
Socio-demographic Index	23	0.003019	spacetime	cf	3		Healthcare access and quality index	23	0.003019	spacetime	rate	3
Tuberculosis prevalence (age-standardized)	24	0.002516	spacetime	cf	3		Tuberculosis prevalence (age-standardized)	24	0.002516	spacetime	rate	3
Education (years per capita)	24	0.002516	spacetime	cf	3		Healthcare access and quality index	24	0.002516	spacetime	rate	3
Socio-demographic Index	24	0.002516	spacetime	cf	3		Population Density (over 1000 ppl/sqkm, proportion)	24	0.002516	spacetime	rate	3
Smoking Prevalence	24	0.002516	spacetime	cf	3		Tuberculosis infection risk-weighted prevalence (age-standardized)	25	0.002097	spacetime	rate	2
Log-transformed SEV scalar: TB	25	0.002097	spacetime	cf	2		Tuberculosis prevalence (age-standardized)	25	0.002097	spacetime	rate	2
Tuberculosis prevalence (age-standardized)	25	0.002097	spacetime	cf	2		Healthcare access and quality index	25	0.002097	spacetime	rate	2
Age-standardized proportion adult underweight	25	0.002097	spacetime	cf	2		Population Density (over 1000 ppl/sqkm, proportion)	25	0.002097	spacetime	rate	2
Education (years per capita)	25	0.002097	spacetime	cf	2		Tuberculosis prevalence (age-standardized)	26	0.001747	spacetime	rate	2
Indoor Air Pollution (All Cooking Fuels)	25	0.002097	spacetime	cf	2		Education (years per capita)	26	0.001747	spacetime	rate	2
Population Density (over 1000 ppl/sqkm, proportion)	25	0.002097	spacetime	cf	2		Healthcare access and quality index	26	0.001747	spacetime	rate	2
Tuberculosis prevalence (age-standardized)	26	0.001747	spacetime	cf	2		Population Density (over 1000 ppl/sqkm, proportion)	26	0.001747	spacetime	rate	2
Alcohol (liters per capita)	26	0.001747	spacetime	cf	2		Tuberculosis infection risk-weighted prevalence (age-standardized)	27	0.001456	spacetime	cf	1

Females							Males					
Covariate name	Rank	Weight	Submodel type	Cause of death data type	Number of draws		Covariate name	Rank	Weight	Submodel type	Cause of death data type	Number of draws
Education (years per capita)	26	0.001747	spacetime	cf	2		Tuberculosis prevalence (age-standardized)	27	0.001456	spacetime	cf	1
Socio-demographic Index	26	0.001747	spacetime	cf	2		Alcohol (liters per capita)	27	0.001456	spacetime	cf	1
Smoking Prevalence	26	0.001747	spacetime	cf	2		Education (years per capita)	27	0.001456	spacetime	cf	1
Tuberculosis infection risk-weighted prevalence (age-standardized)	27	0.001456	spacetime	cf	1		Socio-demographic Index	27	0.001456	spacetime	cf	1
Age-standardized proportion adult underweight	27	0.001456	spacetime	cf	1		Tuberculosis prevalence (age-standardized)	28	0.001213	spacetime	rate	1
Education (years per capita)	27	0.001456	spacetime	cf	1		Alcohol (liters per capita)	28	0.001213	spacetime	rate	1
Socio-demographic Index	27	0.001456	spacetime	cf	1		Education (years per capita)	28	0.001213	spacetime	rate	1
LDI (I$ per capita)	28	0.001213	spacetime	cf	1		Healthcare access and quality index	28	0.001213	spacetime	rate	1
Tuberculosis infection risk-weighted prevalence (age-standardized)	28	0.001213	spacetime	cf	1		Population Density (over 1000 ppl/sqkm, proportion)	28	0.001213	spacetime	rate	1
Tuberculosis prevalence (age-standardized)	28	0.001213	spacetime	cf	1		Tuberculosis prevalence (age-standardized)	29	0.001011	spacetime	rate	1
Education (years per capita)	28	0.001213	spacetime	cf	1		Education (years per capita)	29	0.001011	spacetime	rate	1
LDI (I$ per capita)	29	0.001011	spacetime	cf	1		Healthcare access and quality index	29	0.001011	spacetime	rate	1
Tuberculosis infection risk-weighted prevalence (age-standardized)	29	0.001011	spacetime	cf	1		Smoking Prevalence	29	0.001011	spacetime	rate	1
Tuberculosis prevalence (age-standardized)	29	0.001011	spacetime	cf	1		Tuberculosis infection risk-weighted prevalence (age-standardized)	30	0.000843	spacetime	cf	1
Age-standardized proportion adult underweight	29	0.001011	spacetime	cf	1		Tuberculosis prevalence (age-standardized)	30	0.000843	spacetime	cf	1
Education (years per capita)	29	0.001011	spacetime	cf	1		Age-standardized proportion adult underweight	30	0.000843	spacetime	cf	1
Smoking Prevalence	29	0.001011	spacetime	cf	1		Alcohol (liters per capita)	30	0.000843	spacetime	cf	1
LDI (I$ per capita)	30	0.000843	spacetime	cf	1		Indoor Air Pollution (All Cooking Fuels)	30	0.000843	spacetime	cf	1
Tuberculosis infection risk-weighted prevalence (age-standardized)	30	0.000843	spacetime	cf	1		Population Density (over 1000 ppl/sqkm, proportion)	30	0.000843	spacetime	cf	1

Females							Males					
Covariate name	Rank	Weight	Submodel type	Cause of death data type	Number of draws		Covariate name	Rank	Weight	Submodel type	Cause of death data type	Number of draws
Tuberculosis prevalence (age-standardized)	30	0.000843	spacetime	cf	1		Socio-demographic Index	30	0.000843	spacetime	cf	1
Alcohol (liters per capita)	30	0.000843	spacetime	cf	1		Tuberculosis infection risk-weighted prevalence (age-standardized)	31	0.000702	spacetime	cf	1
Education (years per capita)	30	0.000843	spacetime	cf	1		Tuberculosis prevalence (age-standardized)	31	0.000702	spacetime	cf	1
Smoking Prevalence	30	0.000843	spacetime	cf	1		Education (years per capita)	31	0.000702	spacetime	cf	1
Tuberculosis infection risk-weighted prevalence (age-standardized)	31	0.000702	spacetime	cf	1		Smoking Prevalence	31	0.000702	spacetime	cf	1
Tuberculosis prevalence (age-standardized)	31	0.000702	spacetime	cf	1		Tuberculosis prevalence (age-standardized)	32	0.000585	spacetime	rate	1
Indoor Air Pollution (All Cooking Fuels)	31	0.000702	spacetime	cf	1		Healthcare access and quality index	32	0.000585	spacetime	rate	1
Smoking Prevalence	31	0.000702	spacetime	cf	1		Smoking Prevalence	32	0.000585	spacetime	rate	1
Tuberculosis infection risk-weighted prevalence (age-standardized)	32	0.000585	spacetime	cf	1							
Education (years per capita)	32	0.000585	spacetime	cf	1							
Socio-demographic Index	32	0.000585	spacetime	cf	1							

cf=cause fraction; spacetime= spatiotemporal Gaussian Process Regression

Fatal tuberculosis among HIV-positive individuals

Flowchart

eFigure 5. HIV-AIDS-Tuberculosis mortality: input data, analytical process, and output

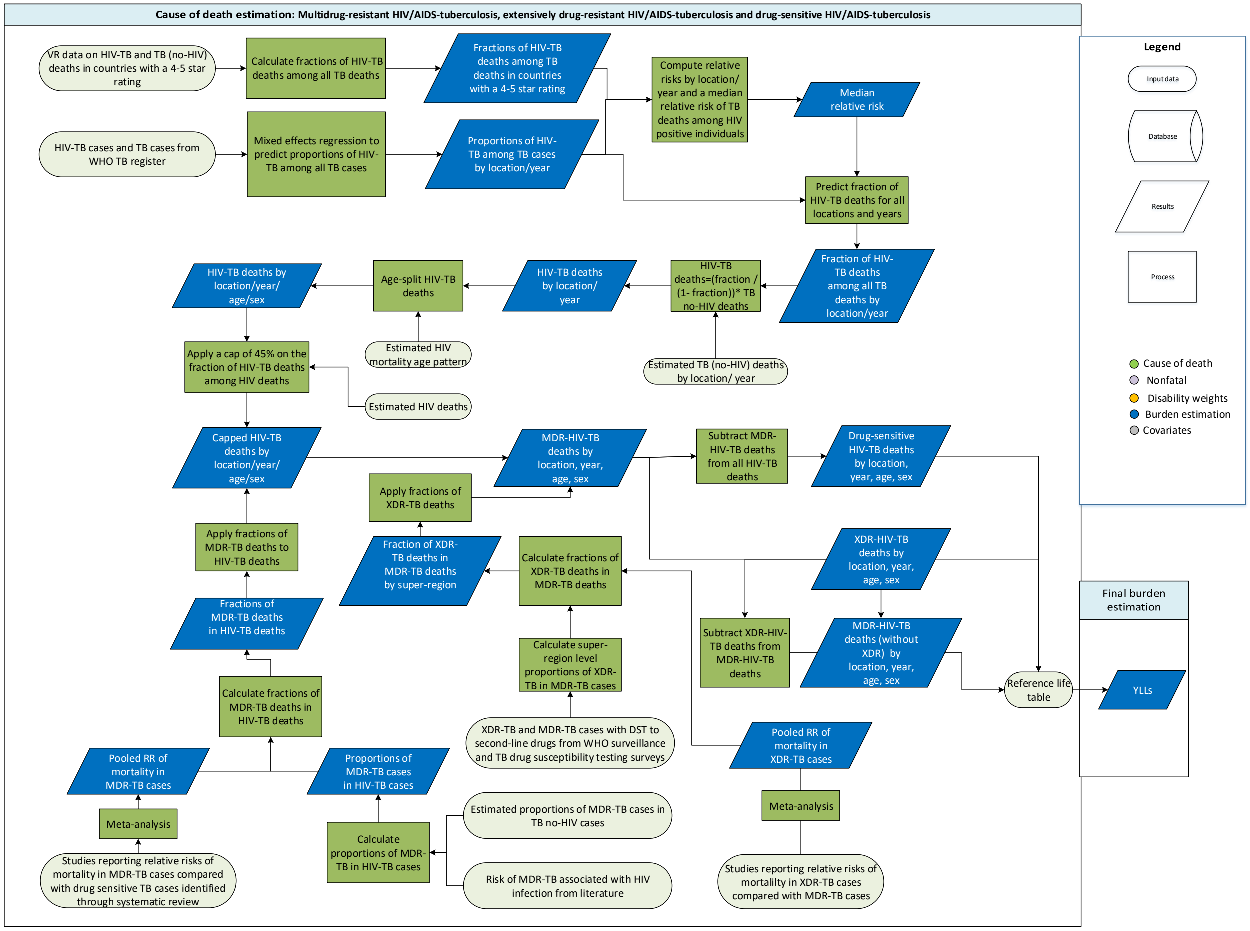

HIV-TB mortality

To determine TB deaths in HIV-positive individuals, we first computed the fraction of HIV-TB deaths among all TB deaths using 382 site-years of high-quality vital registration data (Appendix eFigure 4). Second, we calculated the proportion of HIV-TB cases among all TB cases with an HIV test result as reported in the WHO TB register. We used a mixed-effects regression on the logit of the proportion of HIV-TB among all TB cases to predict for all locations and years, using an adult HIV death rate covariate and country random effects. Third, we assumed the fraction of HIV-TB deaths among all TB deaths in each location and year ($D_{c,y}$) is a function of the prevalence of HIV-TB among TB cases ($P_{c,y}$) and a relative risk (*RR*) of TB death among HIV-positive TB patients that can be generalised over time and between locations:

$$D_{c,y} = \frac{P_{c,y}RR}{P_{c,y}RR + 1 - P_{c,y}}$$

Solving the equation for RR gives:

$$RR = \frac{D_{c,y}P_{c,y} - D_{c,y}}{D_{c,y}P_{c,y} - P_{c,y}}$$

We took the relative risk (*RR*) from each location and year for which we had data on the fraction of HIV-TB deaths among all TB deaths to estimate a median *RR*. We then applied that median *RR* to the predicted proportions of HIV-TB cases among all TB cases to estimate the fraction of HIV-TB deaths among all TB deaths for all locations and years. Next, location-year-specific HIV-TB deaths were calculated using the following equation:

$$Deaths_{HIV-TB} = \frac{D_{c,y}}{1 - D_{c,y}} Deaths_{TB}$$

where $Deaths_{TB}$ is location-year-specific deaths from the CODEm TB HIV-negative model. Finally, we applied the age-sex pattern of the HIV mortality estimates to these HIV-TB deaths to generate location-

year-age-sex-specific HIV-TB deaths. As the HIV-TB deaths were estimated based on the fraction of HIV-TB deaths among all TB deaths, the total number of HIV-TB deaths could exceed the total number of HIV deaths in some locations. To avoid this, we applied a cap of 45% on the fraction of HIV-TB deaths among HIV deaths, based on the largest fraction reported in a review by Cox and colleagues, 2010,[8] and a systematic review and meta-analysis by Ford and colleagues, 2016.[9]

Mortality from MDR-HIV-TB, XDR-HIV-TB and drug-susceptible HIV-TB

We computed the fraction of MDR-HIV-TB deaths among all HIV-TB deaths ($D_{MDR-HIVc,y,a,s}$) using the following formula:

$$D_{MDR-HIVc,y,a,s} = \frac{P_{MDR-HIVc,y,a,s} RR_{MDR}}{P_{MDR-HIVc,y,a,s} RR_{MDR} + 1 - P_{MDR-HIVc,y,a,s}}$$

where $P_{MDR-HIVc,y,a,s}$ is the proportion of MDR-HIV-TB among all HIV-TB cases for each location, year, age, and sex, and RR_{MDR} is the pooled relative risk of mortality in MDR-TB cases compared with drug-susceptible TB cases. We then applied the predicted MDR-HIV-TB death fractions to all HIV-TB death estimates to generate MDR-HIV-TB deaths by location, year, age, and sex. Next, we subtracted MDR-HIV-TB deaths from all HIV-TB deaths to generate drug- susceptible HIV-TB deaths by location, year, age, and sex. The method used to separate out XDR-HIV-TB from MDR-HIV-TB is the same as that for HIV-negative individuals described above.

Systematic review

We conducted a systematic review of studies reporting the risk of death among multi-drug resistant TB cases compared with drug-susceptible TB cases, and the risk of mortality in XDR-TB cases compared with MDR-TB cases. We searched PubMed using the following search terms: ("tuberculosis"[MeSH] OR ("tuberculosis, multidrug-resistant"[MeSH])) AND ("risk"[MeSH] OR "risk"[Title/Abstract]) AND ("mortality"[MeSH] OR "cause specific mortality"[Title/Abstract] OR "cause-specific mortality"[Title/Abstract] OR death*[Title/Abstract]) NOT (animals[MESH] NOT humans[MESH]) ("1980/01/01"[PDAT] : "2016/11/28"[PDAT]). We also searched Google scholar using the following query up to January 5, 2017: ("tuberculosis") AND ("mortality" OR “death”) AND “risk factors” -autopsy -autopsies -nosocomial -qualitative -prison -cancer -malignant -homeless -pneumonia -asthma -maternal -neonatal -hepatitis –contacts

eFigure 6. Flow chart of selecting studies for inclusion in the meta- analysis

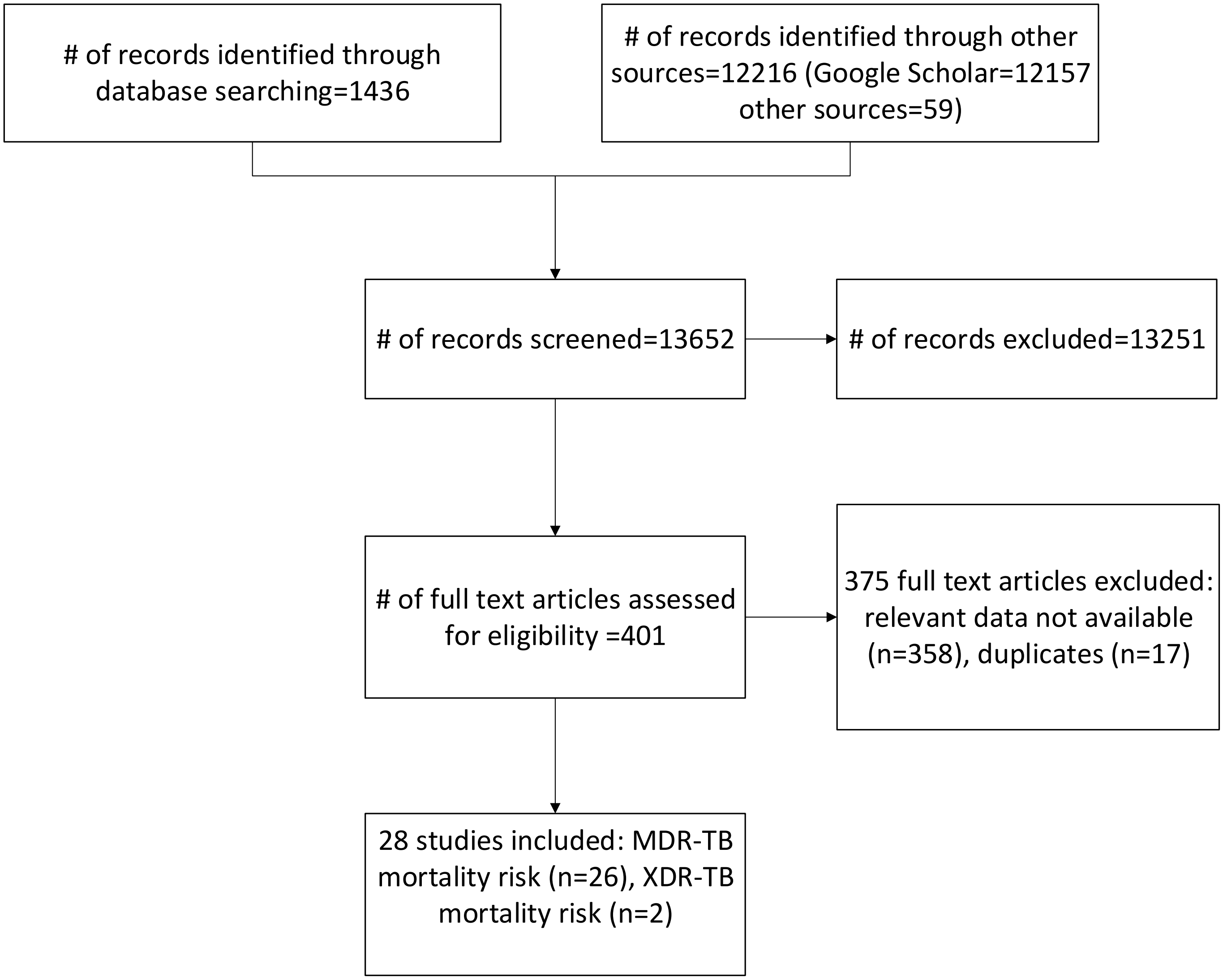

eFigure 7. Meta-analysis of studies examining the risk of mortality in MDR-TB cases compared with drug-susceptible TB cases

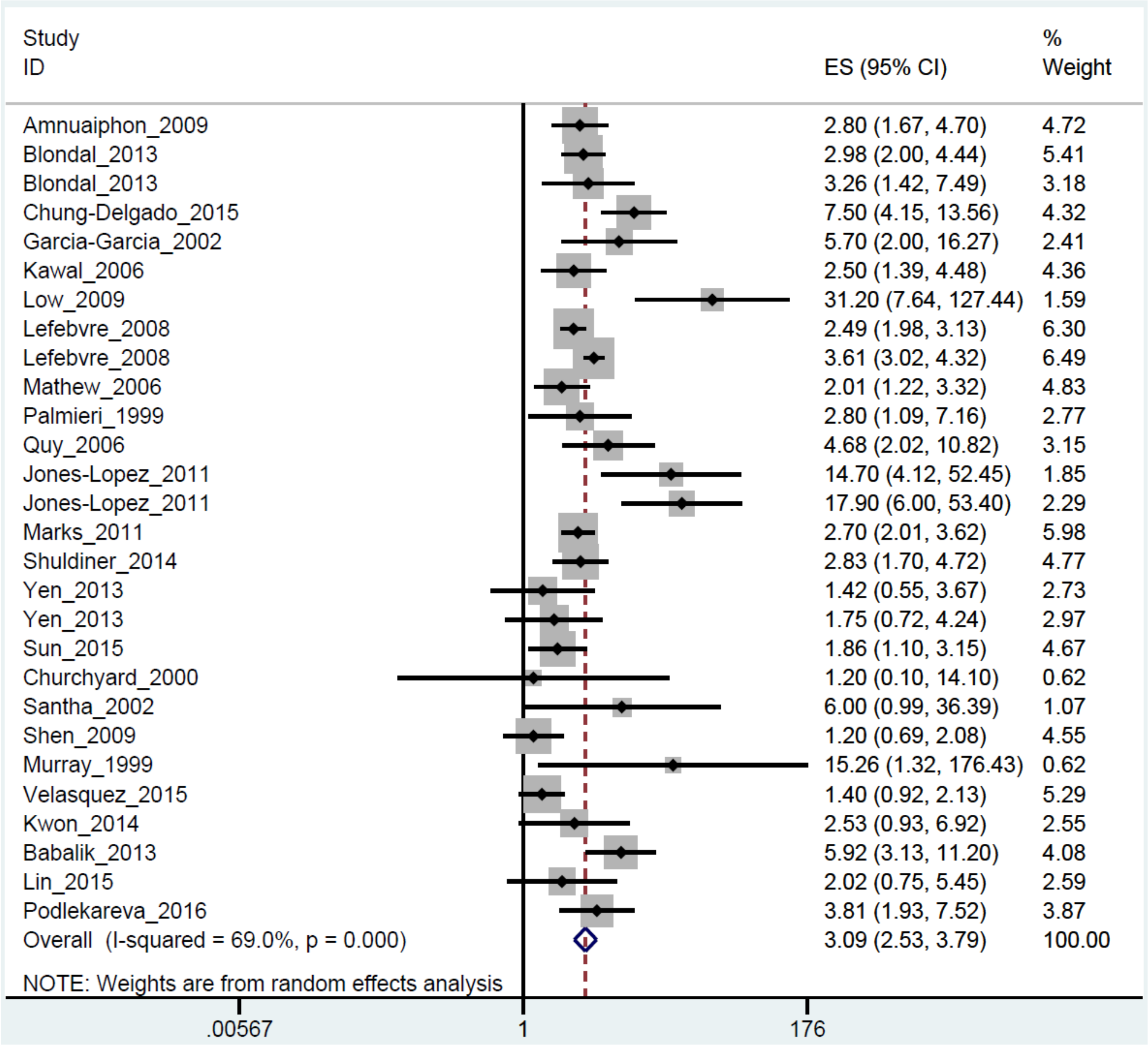

eFigure 8. Meta-analysis of studies examining the risk of mortality in XDR-TB cases compared with MDR-TB cases

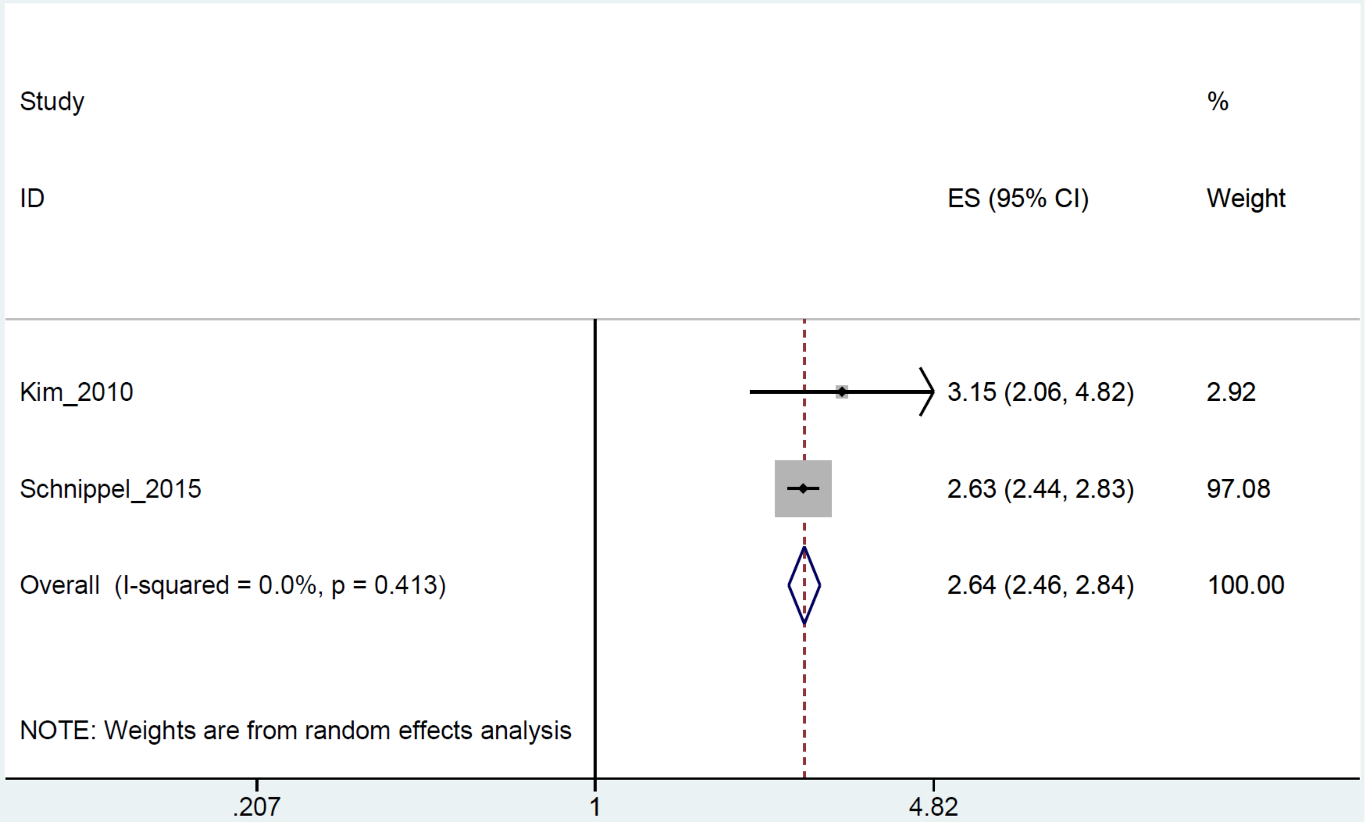

eTable 4. Links to the code and data sources used

Cause name	Links
Mortality input data	
TB	http://ghdx.healthdata.org/gbd-2016/data-input-sources?components=4&causes=297<br><br>https://vizhub.healthdata.org/cod/<br><br>Note. Of the 3470 data sources cited in the input sources tool, the raw input data is not available for download from visualizations for 8.1% of the sources due to confidentiality requirements. These sources include: Burkina Faso - Health and Demographic Surveillance System, United States NVSS Custom Mortality Data and United States Military Deaths, China CDC Disease Surveillance Points and Death Registration, Ghana - Health and Demographic Surveillance System, Ghana - Births and Deaths Registry, Ghana National Tuberculosis Prevalence Survey, Taiwan Vital Statistics, Indonesia Cause of Death Survey, Indonesia Mortality Registration System Strengthening Project and Indonesia Sample Registration System, Philippines Vital Registration, Sri Lanka Vital Statistics, Thailand Burden of Disease and Injuries, Tonga Vital Statistics, Mexico Vital Registration, Brazil Mortality Information System, Brazil Information System for Notifiable Diseases, Iran Vital Registration, Jordan Vital Registration, Palestine - West Bank Vital Registration, Greenland Vital Registration, Saudi Arabia Vital Registration, Turkey Verbal Autopsy Survey, Bangladesh - Health and Demographic Surveillance System, Bangladesh - District Verbal Autopsy Study, Russia Vital Registration, India Annual Rate of Tuberculosis Infection Study and other subnational level Tuberculosis Surveys, India – subnational level Mortality Survey, Japan Vital Registration, Angola - Health and Demographic Surveillance System, Australia Vital Registration, New Zealand Mortality Collection, Ethiopia Demographic Surveillance Verbal Autopsy Data, Ethiopia - Health and Demographic Surveillance System and Addis Ababa Mortality Surveillance Program, Mozambique - Health and Demographic Surveillance System, Tanzania - Health and Demographic Surveillance System, Sweden case reports of tuberculosis, United Kingdom - Mortality Statistics, South Africa - Agincourt Health and Socio-Demographic Surveillance System, and Uruguay Vital Registration.
MDR-TB	http://ghdx.healthdata.org/gbd-2016/data-input-sources?page=29&components=4&causes=946
XDR-TB	http://ghdx.healthdata.org/gbd-2016/data-input-sources?components=4&causes=947
Drug-susceptible TB	http://ghdx.healthdata.org/gbd-2016/data-input-sources?components=4&causes=934

MDR-HIV-TB	http://ghdx.healthdata.org/gbd-2016/data-input-sources?components=4&causes=949
XDR-HIV-TB	http://ghdx.healthdata.org/gbd-2016/data-input-sources?components=4&causes=950
Drug-susceptible HIV-TB	http://ghdx.healthdata.org/gbd-2016/data-input-sources?components=4&causes=948
Mortality code	
TB	http://ghdx.healthdata.org/global-burden-disease-study-2016-gbd-2016-causes-death-8
MDR-TB, XDR-TB, Drug-susceptible-TB	http://ghdx.healthdata.org/global-burden-disease-study-2016-gbd-2016-causes-death-6
HIV-TB, MDR-HIV-TB, XDR-HIV-TB, Drug-susceptible-HIV-TB	http://ghdx.healthdata.org/global-burden-disease-study-2016-gbd-2016-causes-death-6
Non-fatal input data	
TB	http://ghdx.healthdata.org/gbd-2016/data-input-sources?components=5&causes=297<br><br>WHO notification data are downloadable at: http://www.who.int/tb/country/data/download/en/
Latent TB	http://ghdx.healthdata.org/gbd-2016/data-input-sources?components=5&causes=954
MDR-TB	http://ghdx.healthdata.org/gbd-2016/data-input-sources?components=5&causes=946
XDR-TB	http://ghdx.healthdata.org/gbd-2016/data-input-sources?components=5&causes=947
MDR-HIV-TB	http://ghdx.healthdata.org/gbd-2016/data-input-sources?components=5&causes=949
XDR-HIV-TB	http://ghdx.healthdata.org/gbd-2016/data-input-sources?components=5&causes=950
Non-fatal code	
Latent TB; Active TB by drug-resistance type and HIV status	http://ghdx.healthdata.org/global-burden-disease-study-2016-gbd-2016-nonfatal-code-12

eTable 5. A comparison of methods used by GBD and WHO for estimating the burden of tuberculosis

	GBD 2016	WHO global TB report (2017)
**Mortality**		
Key data sources or inputs	• Vital registration (VR) data (15943 site-years)<br>• Verbal autopsy (VA) data (764 site-years)<br>• Number of HIV-TB cases and TB all-forms cases with an HIV test result recorded in the WHO TB notifications register<br>• HIV-TB deaths and TB deaths from high-quality VR data (382 site-years)	• VR data and mortality survey (1526 country-years)<br>• WHO TB and HIV-TB incidence estimates<br>• WHO TB case fatality rate (CFR) estimates<br>• GBD 2015 mortality estimates published by the Institute for Health Metrics and Evaluation (for 18 countries)
Kay adjustments to data	• VR and VA data adjusted based on detailed analysis of garbage coding<br>• VR data adjusted for misclassification of HIV deaths as TB deaths<br>• VR data adjusted for estimated completeness in each country-year<br>• Excluded VA data in countries with age-standardized HIV prevalence > 5% because of a high probability of misclassification	• TB mortality adjusted upwards for incomplete coverage and ill-defined causes of death<br>• Estimates for 18 countries adjusted for HIV/TB miscoding using GBD 2015 estimates<br>• VR data interpolated for missing data and trailing missing values predicted using a Kalman smoother or using the last observation carried forward or in the case of leading missing values, the next observation carried backwards
Modeling strategy	*Estimating TB no-HIV* mortality<br>• Used the Cause of Death Ensemble Modeling (CODEm)<br>*Estimating HIV-TB mortality*<br>• Used a population attributable fraction approach taking into account baseline risk	*Estimating TB no-HIV* mortality<br>Countries with VR:<br>• TB mortality directly from VR data (126 countries)<br>Countries without VR<br>• Mortality back-calculated based on estimated incidence and CFR<br>*Estimating HIV-TB mortality*<br>• All countries: HIV-TB mortality back-calculated based on estimated HIV-TB incidence and CFR

	GBD 2016	WHO global TB report (2017)
Uncertainty	• CODEm generates uncertainty intervals for predicted death rates by sampling the posterior distribution of each of the component models in proportion to the weight of each model in the ensemble; mixed effects component model uncertainty includes uncertainty in the betas and the hierarchical random effects; spatiotemporal Gaussian Process Regression component models include uncertainty from the mean prior and the data variance<br>• Uncertainty interval coverage evaluated objectively with out-of-sample predictive validity<br>• Uncertainty for HIV-TB mortality estimates include uncertainty from the variance-covariance matrix of the fixed effects and the hierarchical random effects in estimating the proportion of TB cases with HIV, as well as uncertainty from TB no-HIV mortality estimates from CODEm	• Incorporated: sampling uncertainty in the underlying measurements of TB mortality rates from data sources, parameter uncertainty in models, uncertainty in imputation methods, and in the models themselves.<br>• Propagated uncertainty based on methods outlined by Ku[1] and Lab[2]
**Morbidity**		
Key data sources and inputs	• TB prevalence surveys<br>• VR data from countries with a four or five-star rating on mortality data<br>• WHO notification data<br>• Data from an untreated cohort of TB cases in the 1960s[3]<br>• Tuberculin surveys and cohort studies reporting the risk of progression to active TB as a function of induration size<br>• GBD 2016 TB cause-specific mortality estimates	• WHO TB case notifications<br>• TB prevalence surveys<br>• Expert opinion of case detection rate (CDR)<br>• Duration estimates<br>• Inventory studies<br>• Tuberculin surveys<br>• Sero-surveys of newly diagnosed TB cases and HIV sentinel surveillance systems<br>• UNAIDs estimates of HIV prevalence

	GBD 2016	WHO global TB report (2017)
	• GBD 2016 HIV prevalence estimates<br>• Number of HIV-TB cases and TB all-forms cases with an HIV test result recorded in the WHO TB notifications register	
Key adjustments to data	• Correction of case notifications for missing age groups, smear-unknown and relapsed cases<br>• Prevalence surveys (bacteriologically-confirmed) adjusted for extra-pulmonary TB	• Case notification data adjusted by a standard factor for underreporting, misclassification and over-reporting<br>• For 54 countries, notification data adjusted using expert opinion case detection rates<br>• Bacteriologically-confirmed pulmonary TB among those aged > 15 years (from prevalence surveys) adjusted for pulmonary TB in children and extra-pulmonary TB in all ages
Modeling strategy	All countries<br>• Model risk-weighted prevalence of LTBI in DisMod MR 2.1<br>• Mortality to incidence ratio regression using input data from high quality cause of death data with SDI as a covariate<br>○ Anchoring the lower end of the SDI scale with data from an untreated cohort of TB cases in the 1960s[3].<br>• Divide the inputs on prevalence, incidence, and cause-specific mortality by the risk-weighted LTBI prevalence to model those at risk<br>• Compute location-year-age-sex specific prevalence-based excess mortality rate (EMR) for countries with prevalence surveys, and incidence-based	Countries with national prevalence surveys<br>• Incidence estimated from prevalence survey results combined with either a dynamic model or duration estimates/assumptions (24 countries)<br>High-income and selected upper-middle income countries<br>• Notifications adjusted by a standard factor (134 countries, excluding the Netherlands and the United Kingdom)<br>Other countries<br>• Case notification data adjusted for expert opinion about CDR (54 countries)<br>• Capture-recapture modeling (5 countries)<br>All countries<br>• TB incidence among children (<15 years) estimated by an ensemble approach in which estimates derived from notifications adjusted for CDR

	GBD 2016	WHO global TB report (2017)
	EMR for four-to-five star countries<br>• DisMod-MR 2.1 to ensure consistency of estimates between incidence, prevalence, mortality, and EMR<br>• Multiply outputs by risk-weighted prevalence of LTBI to get population level estimates<br>• Estimated the proportions of HIV-TB cases among all TB cases for all locations and years, using the adult HIV death rate as a covariate in a mixed effects regression<br>• HIV-TB incident and prevalent cases were age-sex splitted based on the age-sex pattern of estimated HIV prevalence	were combined with estimates derived from dynamic modeling<br>• Different HIV-TB data sources were combined using local polynomial regression fitting by weighted least squares
Uncertainty	• DisMod-MR generates posterior distributions for incidence, prevalence, remission, and excess mortality that is a function of data variance and model parameter uncertainty	• Propagated uncertainty based on methods outlined by Ku[1] and Lab[2]

[1] Ku HH. Notes on the use of propagation of error formulas. *Journal of Research of the National Bureau of Standards, Section C: Engineering and Instrumentation* 1966; **70C**: 263

[2] Lab AS. An Introduction To Error Propagation: Derivation, Meaning, and Examples of Equation C_Y=F_X C_X F^X_T. Swiss Federal Institute of Technology, Lausanne, 1998 http://www.nada.kth.se/~kai-a/papers/arrasTR-9801-R3.pdf.

[3] Bangalore National Tuberculosis Institute. Tuberculosis in a rural population of South India: a five-year epidemiological study. *Bull WHO* 1974; **51**(5): 473.

Results

eFigure 9. Global age-sex distribution of tuberculosis incidence and deaths in HIV-positive individuals in 2016

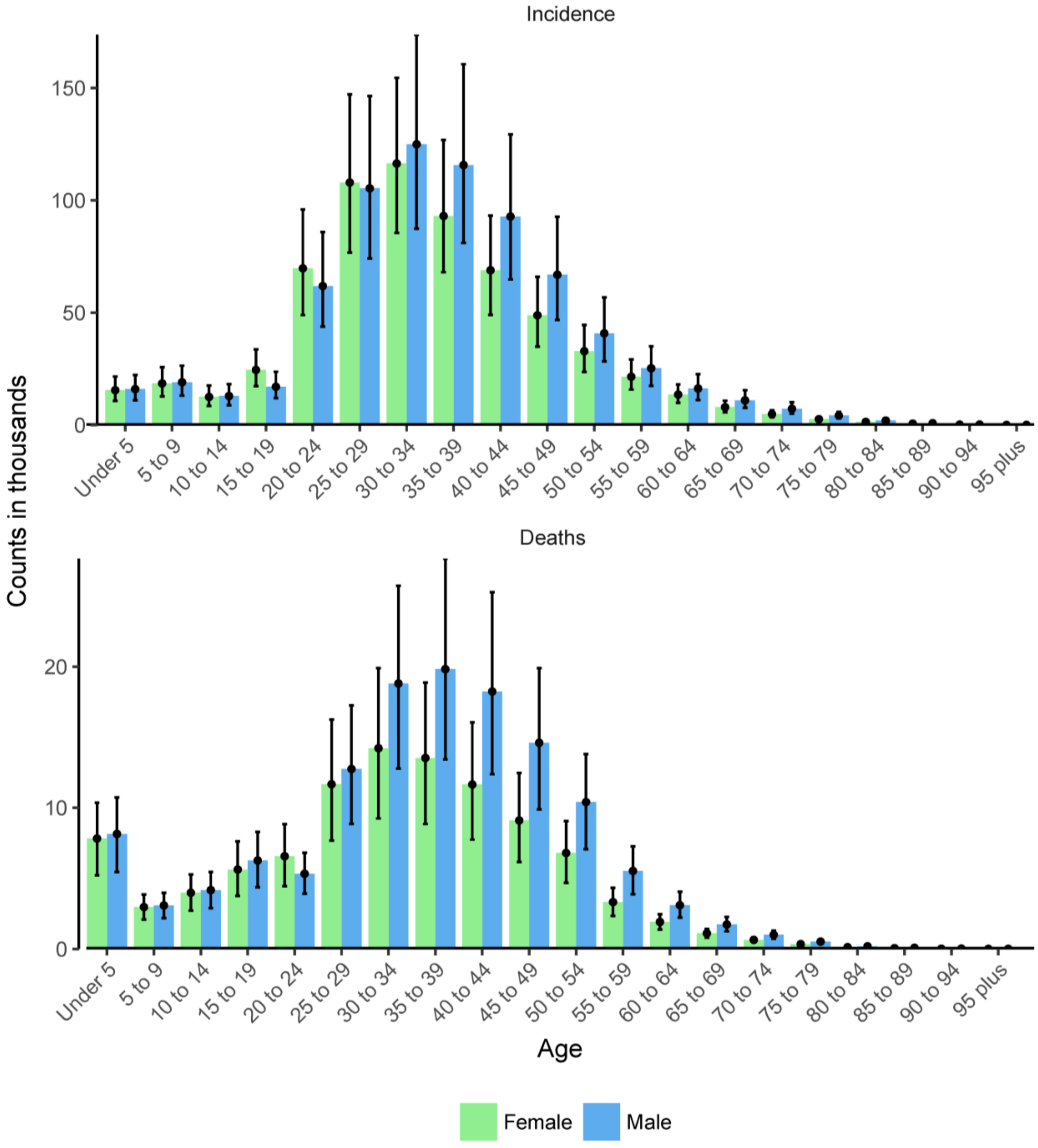

eFigure 10. Age-standardized Incidence and deaths rates, per 100,000, for HIV-positive Tuberculosis in 2016

Incidence

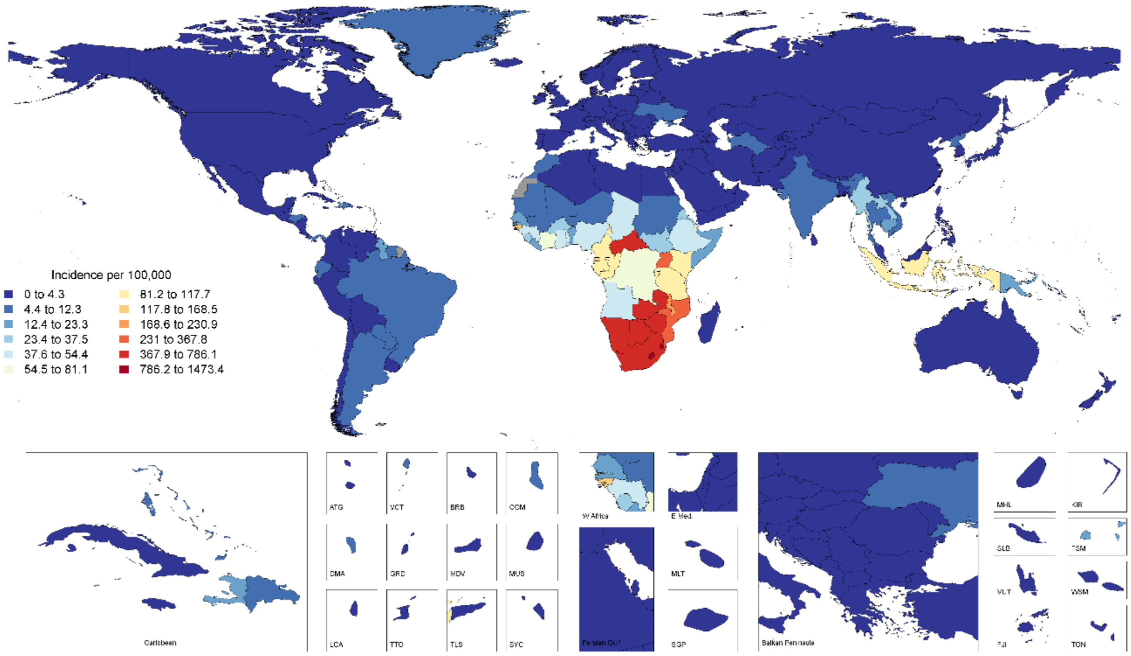

Deaths

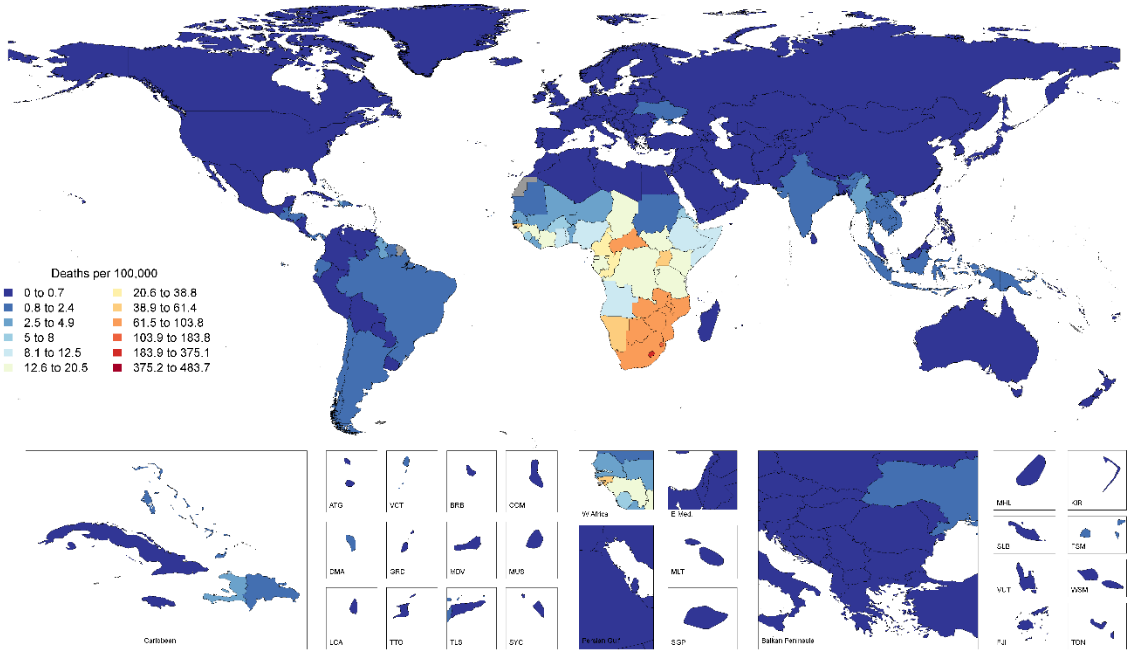

ATG=Antigua and Barbuda. BRB=Barbados. COM=Comoros. DMA=Dominica. E Med=Eastern Mediterranean. FJI=Fiji. FSM=Federated States of Micronesia. GRD=Grenada. KIR=Kiribati. LCA=Saint Lucia. MDV=Maldives. MHL=Marshall Islands. MLT=Malta. MUS=Mauritius. SGP=Singapore. SLB=Solomon Islands. SYC=Seychelles. TLS=Timor-Leste.

eTable 6. Tuberculosis, drug-susceptible tuberculosis, multidrug-resistant tuberculosis, and extensively drug-resistant tuberculosis incident cases in HIV-negative individuals, and annualized rates of change of age-standardized rates for 195 countries and territories

	Number of incident cases (all ages, both sexes) with 95% uncertainty intervals (2016)				Annualized rate of change of age-standardized rate (%) with 95% uncertainty intervals							
					1990-2006				2006-2016			
	**Drug-susceptible TB**	**MDR TB**	**XDR TB**	**All HIV-negative TB**	**Drug-susceptible TB**	**MDR TB**	**XDR TB**	**All HIV-negative TB**	**Drug-susceptible TB**	**MDR TB**	**XDR TB**	**All HIV-negative TB**
Global	8,705,207 (7,754,638-9,817,655)	295,637 (261,369-335,586)	18,452 (16,087-21,187)	9,019,296 (8,051,800 to 10,156,811)	-1.8 (-2.0 to -1.5)	11.5 (10.8 to 12.3)	43.9 (43.1 to 44.8)	-1.6 (-1.8 to -1.3)	-1.3 (-1.5 to -1.1)	-2.1 (-2.9 to -1.3)	7.9 (6.6 to 9.1)	-1.3 (-1.5 to -1.2)
Afghanistan	45,310 (36,326-54,359)	3,155 (613-9,294)	111 (22-328)	48,577 (40,244 to 58,087)	-1.1 (-2.0 to -0.3)	30.7 (21.2 to 45.0)	49.2 (40.4 to 55.3)	-0.7 (-1.2 to 0.0)	-3.0 (-4.9 to -1.4)	-2.1 (-22.3 to 15.7)	6.1 (-14.1 to 23.9)	-2.9 (-4.0 to -2.1)
Albania	387 (337-441)	3 (1-9)	1 (0-2)	391 (340 to 444)	-3.1 (-3.5 to -2.8)	13.8 (2.3 to 27.5)	28.6 (18.0 to 35.9)	-3.1 (-3.4 to -2.7)	-2.5 (-3.0 to -2.0)	-3.7 (-23.0 to 15.4)	7.6 (-11.7 to 26.6)	-2.5 (-3.1 to -2.0)
Algeria	19,278 (15,643-23,310)	409 (39-1,790)	14 (1-63)	19,702 (16,146 to 23,808)	-3.8 (-4.3 to -3.3)	-0.8 (-11.7 to 9.8)	31.5 (23.3 to 37.7)	-3.7 (-4.2 to -3.3)	-2.1 (-3.0 to -1.3)	0.7 (-19.4 to 15.7)	8.9 (-11.2 to 23.9)	-2.0 (-2.8 to -1.3)
American Samoa	15 (12-18)	0 (0-1)	0 (0-0)	15 (12 to 18)	-2.3 (-3.0 to -1.9)	8.7 (-18.7 to 36.4)	28.3 (-4.4 to 40.1)	-2.2 (-2.6 to -1.9)	-1.1 (-1.9 to -0.2)	-4.3 (-42.3 to 29.1)	7.6 (-30.4 to 41.0)	-1.2 (-1.6 to -0.7)
Andorra	7 (5-10)	0 (0-0)	0 (0-0)	7 (5 to 10)	-2.4 (-3.0 to -1.8)	-2.4 (-5.7 to 0.9)	-1.5 (-5.0 to 1.8)	-2.4 (-3.0 to -1.8)	-2.0 (-2.9 to -1.2)	-2.5 (-7.0 to 2.0)	5.2 (0.7 to 9.7)	-2.0 (-2.9 to -1.2)
Angola	68,586 (58,719-79,553)	504 (275-883)	3 (2-6)	69,093 (59,150 to 80,208)	-2.1 (-2.6 to -1.7)	7.3 (3.2 to 11.4)	27.4 (23.1 to 30.7)	-2.1 (-2.5 to -1.7)	-2.4 (-2.9 to -1.9)	-2.9 (-8.6 to 3.1)	8.3 (2.6 to 14.2)	-2.4 (-2.9 to -1.9)
Antigua and Barbuda	10 (8-12)	0 (0-0)	0 (0-0)	10 (8 to 12)	-1.6 (-2.1 to -1.2)	4.0 (-10.4 to 17.2)	20.5 (6.4 to 28.5)	-1.6 (-2.1 to -1.2)	-1.5 (-2.1 to -0.8)	-4.5 (-21.6 to 13.3)	5.3 (-11.8 to 23.0)	-1.5 (-2.1 to -0.9)
Argentina	5,444 (4,375-6,706)	146 (22-499)	18 (3-63)	5,609 (4,565 to 6,876)	-4.3 (-5.2 to -3.5)	9.3 (0.4 to 23.0)	33.4 (27.6 to 37.9)	-4.2 (-5.0 to -3.4)	-0.8 (-2.0 to 0.1)	-0.7 (-18.5 to 12.3)	7.0 (-10.9 to 20.0)	-0.8 (-1.7 to -0.0)
Armenia	990 (766-1,189)	108 (24-287)	24 (5-63)	1,122 (981 to 1,282)	-0.7 (-1.4 to -0.0)	31.9 (24.0 to 44.1)	50.8 (46.8 to 53.9)	-0.0 (-0.6 to 0.5)	-4.3 (-6.9 to -2.8)	-3.8 (-20.2 to 8.9)	7.5 (-9.0 to 20.2)	-4.1 (-4.7 to -3.6)
Australia	1,129 (969-1,296)	24 (10-45)	3 (1-6)	1,156 (994 to 1,330)	-3.6 (-4.5 to -2.6)	8.1 (-2.1 to 18.7)	25.3 (17.0 to 31.4)	-3.5 (-4.4 to -2.5)	-1.2 (-1.9 to -0.5)	-0.3 (-12.5 to 13.3)	7.4 (-4.8 to 21.0)	-1.2 (-1.8 to -0.6)
Austria	596 (452-769)	17 (11-25)	2 (1-3)	615 (468 to 795)	-4.3 (-5.1 to -3.5)	6.0 (3.2 to 9.0)	29.4 (27.0 to 31.5)	-4.2 (-5.0 to -3.4)	-2.2 (-3.2 to -1.3)	0.5 (-3.5 to 3.9)	8.1 (4.2 to 11.5)	-2.1 (-3.1 to -1.2)
Azerbaijan	10,722 (8,578-12,805)	1,839 (921-3,202)	403 (202-702)	12,964 (11,243 to 14,916)	-0.1 (-2.0 to 0.8)	39.5 (30.2 to 51.7)	61.4 (53.6 to 66.4)	1.0 (0.7 to 1.4)	-3.3 (-6.0 to 0.2)	-4.2 (-15.6 to 10.2)	7.0 (-4.4 to 21.5)	-3.2 (-3.9 to -2.6)

	Number of incident cases (all ages, both sexes) with 95% uncertainty intervals (2016)				Annualized rate of change of age-standardized rate (%) with 95% uncertainty intervals							
					1990-2006				2006-2016			
	**Drug-susceptible TB**	**MDR TB**	**XDR TB**	**All HIV-negative TB**	**Drug-susceptible TB**	**MDR TB**	**XDR TB**	**All HIV-negative TB**	**Drug-susceptible TB**	**MDR TB**	**XDR TB**	**All HIV-negative TB**
Bahrain	317 (200-466)	8 (2-21)	0 (0-1)	325 (207 to 477)	-2.5 (-3.2 to -1.7)	19.9 (8.8 to 34.4)	27.2 (17.7 to 34.1)	-2.4 (-3.1 to -1.6)	-3.0 (-4.3 to -1.8)	1.2 (-15.9 to 21.5)	9.4 (-7.7 to 29.7)	-2.9 (-4.2 to -1.8)
Bangladesh	176,721 (155,824-199,510)	3,960 (3,040-4,979)	97 (75-122)	180,778 (159,471 to 204,052)	-4.6 (-4.8 to -4.3)	23.4 (21.6 to 25.2)	35.9 (34.2 to 37.4)	-4.4 (-4.7 to -4.1)	-1.6 (-2.1 to -1.1)	-1.5 (-4.0 to 1.0)	8.2 (5.7 to 10.7)	-1.6 (-2.1 to -1.2)
Barbados	30 (25-36)	0 (0-0)	0 (0-0)	30 (25 to 36)	-1.1 (-1.5 to -0.7)	0.5 (-12.1 to 13.6)	2.8 (-11.1 to 11.5)	-1.1 (-1.5 to -0.7)	-0.9 (-1.5 to -0.4)	-2.3 (-19.6 to 16.3)	7.5 (-9.8 to 26.1)	-0.9 (-1.5 to -0.4)
Belarus	2,307 (1,941-2,659)	1,175 (978-1,421)	257 (214-311)	3,740 (3,226 to 4,266)	-1.3 (-2.4 to -0.3)	45.9 (41.6 to 50.5)	57.2 (55.0 to 59.2)	1.0 (0.5 to 1.8)	-4.2 (-5.9 to -2.4)	-2.3 (-5.4 to 1.3)	9.0 (5.8 to 12.6)	-3.1 (-3.7 to -2.6)
Belgium	888 (686-1,129)	15 (10-21)	2 (1-3)	905 (700 to 1,151)	-2.5 (-3.4 to -1.7)	3.6 (0.8 to 6.5)	28.5 (26.3 to 30.4)	-2.5 (-3.3 to -1.7)	-1.5 (-2.4 to -0.7)	-2.0 (-5.8 to 1.6)	5.6 (1.8 to 9.2)	-1.5 (-2.4 to -0.7)
Belize	108 (87-133)	1 (0-2)	0 (0-0)	108 (87 to 134)	-1.2 (-1.7 to -0.7)	5.1 (-9.3 to 18.3)	28.3 (14.4 to 37.4)	-1.2 (-1.7 to -0.6)	-2.5 (-3.1 to -1.9)	-6.2 (-24.9 to 12.0)	3.6 (-15.1 to 21.8)	-2.5 (-3.1 to -2.0)
Benin	11,277 (9,232-13,675)	106 (36-247)	1 (0-2)	11,384 (9,312 to 13,837)	-1.0 (-1.7 to -0.5)	10.5 (3.5 to 17.5)	20.7 (14.3 to 25.4)	-1.0 (-1.7 to -0.4)	-1.5 (-2.2 to -0.8)	-0.1 (-11.1 to 10.5)	11.0 (0.0 to 21.6)	-1.5 (-2.2 to -0.8)
Bermuda	4 (3-5)	0 (0-0)	0 (0-0)	4 (3 to 5)	-2.1 (-2.5 to -1.7)	-1.0 (-12.8 to 12.1)	-1.4 (-14.3 to 6.8)	-2.1 (-2.5 to -1.7)	-1.1 (-1.7 to -0.5)	-2.5 (-20.1 to 15.1)	7.3 (-10.3 to 24.9)	-1.1 (-1.7 to -0.5)
Bhutan	1,101 (931-1,298)	30 (22-41)	1 (1-1)	1,131 (957 to 1,336)	-3.2 (-3.5 to -2.8)	13.6 (11.7 to 15.4)	37.9 (36.1 to 39.8)	-3.0 (-3.3 to -2.7)	-2.2 (-2.7 to -1.8)	-1.8 (-4.4 to 0.8)	7.9 (5.3 to 10.5)	-2.2 (-2.7 to -1.8)
Bolivia	9,998 (8,900-11,113)	288 (67-834)	23 (5-66)	10,309 (9,220 to 11,401)	-5.1 (-5.6 to -4.7)	7.4 (-1.6 to 16.8)	44.7 (35.3 to 51.3)	-4.9 (-5.2 to -4.6)	-3.1 (-3.7 to -2.5)	-4.4 (-16.7 to 7.3)	5.4 (-6.9 to 17.1)	-3.1 (-3.6 to -2.7)
Bosnia and Herzegovina	1,139 (999-1,281)	3 (1-7)	1 (0-2)	1,142 (1,004 to 1,283)	-3.2 (-3.5 to -3.0)	2.0 (-7.0 to 15.7)	26.5 (22.1 to 30.0)	-3.2 (-3.5 to -3.0)	-2.4 (-2.8 to -2.0)	-4.6 (-18.4 to 6.5)	6.7 (-7.1 to 17.8)	-2.4 (-2.8 to -2.0)
Botswana	13,238 (9,748-18,115)	248 (76-559)	2 (0-4)	13,488 (9,997 to 18,327)	2.5 (1.3 to 3.6)	14.7 (8.1 to 23.2)	33.4 (29.6 to 36.5)	2.6 (1.4 to 3.7)	-3.2 (-5.0 to -1.5)	-0.1 (-12.0 to 9.4)	11.0 (-0.8 to 20.6)	-3.1 (-4.9 to -1.4)
Brazil	62,663 (54,030-71,780)	957 (806-1,115)	76 (64-88)	63,696 (54,861 to 72,921)	-1.9 (-2.3 to -1.3)	23.0 (22.3 to 23.8)	32.5 (31.5 to 33.4)	-1.8 (-2.2 to -1.2)	-2.2 (-2.4 to -2.0)	-3.2 (-3.9 to -2.5)	6.6 (5.9 to 7.3)	-2.2 (-2.4 to -2.0)
Brunei	239 (202-275)	0 (0-0)	0 (0-0)	240 (202 to 275)	-3.7 (-4.0 to -3.4)	1.4 (-2.0 to 4.7)	16.0 (12.8 to 18.9)	-3.7 (-4.0 to -3.4)	-2.4 (-2.9 to -1.8)	3.9 (-1.0 to 8.9)	11.6 (6.6 to 16.6)	-2.4 (-2.9 to -1.8)
Bulgaria	1,543 (1,366-1,724)	37 (10-99)	8 (2-22)	1,588 (1,418 to 1,768)	-0.0 (-0.7 to 0.8)	23.5 (13.9 to 37.7)	39.4 (31.0 to 45.1)	0.2 (-0.5 to 0.9)	-4.5 (-5.2 to -3.6)	-5.0 (-22.4 to 13.7)	6.2 (-11.1 to 24.9)	-4.4 (-5.0 to -3.9)

	Number of incident cases (all ages, both sexes) with 95% uncertainty intervals (2016)				Annualized rate of change of age-standardized rate (%) with 95% uncertainty intervals							
					1990-2006				2006-2016			
	Drug-susceptible TB	MDR TB	XDR TB	All HIV-negative TB	Drug-susceptible TB	MDR TB	XDR TB	All HIV-negative TB	Drug-susceptible TB	MDR TB	XDR TB	All HIV-negative TB
Burkina Faso	24,191 (19,774-29,759)	383 (110-996)	2 (1-6)	24,577 (20,168 to 30,083)	-1.2 (-1.6 to -0.7)	12.6 (4.9 to 20.2)	26.9 (19.3 to 32.7)	-1.1 (-1.5 to -0.6)	-0.5 (-1.4 to 0.5)	-1.8 (-11.6 to 9.0)	9.3 (-0.5 to 20.1)	-0.5 (-1.4 to 0.5)
Burundi	34,262 (27,714-42,836)	708 (194-1,799)	5 (1-12)	34,975 (28,288 to 43,790)	-0.8 (-1.5 to -0.2)	19.8 (11.7 to 27.8)	31.6 (23.5 to 37.3)	-0.7 (-1.4 to -0.1)	-0.7 (-1.7 to 0.3)	0.8 (-10.7 to 11.9)	11.9 (0.4 to 23.0)	-0.7 (-1.6 to 0.3)
Cambodia	30,330 (26,024-35,074)	446 (77-1,530)	41 (7-139)	30,817 (26,703 to 35,595)	-2.1 (-2.7 to -1.7)	19.8 (9.7 to 34.6)	47.1 (40.8 to 52.0)	-2.0 (-2.6 to -1.6)	-5.1 (-5.8 to -4.5)	-6.4 (-25.2 to 9.0)	5.6 (-13.3 to 20.9)	-5.2 (-5.7 to -4.6)
Cameroon	30,425 (23,569-39,329)	469 (127-1,208)	3 (1-8)	30,897 (24,068 to 39,839)	0.1 (-0.8 to 1.0)	13.4 (5.9 to 21.6)	26.2 (18.1 to 31.9)	0.2 (-0.7 to 1.1)	-1.9 (-2.6 to -1.2)	-2.9 (-13.6 to 8.2)	8.2 (-2.4 to 19.4)	-1.9 (-2.6 to -1.2)
Canada	1,531 (1,378-1,697)	14 (12-17)	2 (1-2)	1,547 (1,392 to 1,714)	-1.8 (-2.2 to -1.4)	-0.0 (-1.3 to 1.4)	19.5 (18.3 to 20.7)	-1.8 (-2.2 to -1.4)	-0.0 (-0.5 to 0.4)	-0.6 (-2.4 to 1.2)	7.1 (5.3 to 8.8)	-0.0 (-0.5 to 0.4)
Cape Verde	342 (277-424)	5 (1-13)	0 (0-0)	347 (282 to 433)	-1.9 (-2.4 to -1.5)	11.5 (3.6 to 19.7)	22.3 (14.4 to 27.6)	-1.8 (-2.3 to -1.4)	-2.9 (-3.6 to -2.3)	-3.8 (-14.8 to 7.6)	7.3 (-3.6 to 18.7)	-2.9 (-3.6 to -2.3)
Central African Republic	32,508 (25,672-39,953)	180 (96-316)	1 (1-2)	32,689 (25,770 to 40,269)	-0.5 (-1.2 to 0.2)	7.4 (3.3 to 11.7)	29.4 (25.5 to 32.8)	-0.5 (-1.2 to 0.3)	-0.2 (-0.8 to 0.4)	-1.2 (-6.8 to 4.2)	10.0 (4.4 to 15.3)	-0.2 (-0.9 to 0.4)
Chad	17,683 (14,291-21,848)	286 (80-740)	2 (1-5)	17,970 (14,485 to 22,241)	-0.5 (-1.3 to 0.2)	13.3 (4.9 to 21.3)	27.9 (19.8 to 33.9)	-0.4 (-1.2 to 0.3)	-2.5 (-3.2 to -1.9)	-3.7 (-14.7 to 7.4)	7.4 (-3.6 to 18.5)	-2.5 (-3.2 to -1.9)
Chile	2,115 (1,747-2,522)	17 (8-35)	2 (1-4)	2,135 (1,762 to 2,547)	-5.5 (-6.1 to -4.8)	3.0 (-8.6 to 15.8)	25.7 (18.0 to 31.4)	-5.4 (-6.1 to -4.8)	-1.9 (-2.5 to -1.4)	-1.7 (-13.6 to 11.2)	6.0 (-5.9 to 18.9)	-1.9 (-2.5 to -1.4)
China	1,127,371 (1,062,491-1,196,312)	49,103 (43,063-56,661)	4,477 (3,926-5,166)	1,180,952 (1,113,479 to 1,251,185)	-4.2 (-4.4 to -4.0)	4.7 (3.7 to 5.6)	45.2 (44.4 to 46.0)	-3.9 (-4.1 to -3.8)	-2.2 (-2.4 to -2.0)	-4.3 (-5.5 to -3.0)	7.6 (6.4 to 9.0)	-2.3 (-2.5 to -2.1)
Colombia	9,366 (7,941-10,901)	235 (117-410)	19 (9-32)	9,619 (8,136 to 11,205)	-2.9 (-3.4 to -2.4)	15.5 (11.4 to 20.2)	33.4 (30.8 to 35.9)	-2.7 (-3.3 to -2.2)	-1.9 (-2.5 to -1.4)	-3.7 (-9.8 to 1.9)	6.1 (0.0 to 11.7)	-2.0 (-2.5 to -1.4)
Comoros	1,462 (1,190-1,799)	30 (8-75)	0 (0-0)	1,492 (1,214 to 1,830)	-0.4 (-0.9 to 0.2)	20.4 (12.5 to 28.3)	28.0 (20.2 to 33.9)	-0.3 (-0.7 to 0.2)	-0.2 (-0.9 to 0.5)	0.9 (-10.8 to 11.6)	12.0 (0.4 to 22.7)	-0.1 (-0.9 to 0.5)
Congo	15,696 (12,943-18,738)	115 (60-204)	1 (0-1)	15,812 (13,019 to 18,852)	-2.3 (-2.8 to -1.8)	7.3 (3.3 to 11.2)	27.7 (23.5 to 31.1)	-2.2 (-2.8 to -1.8)	-2.3 (-2.8 to -1.6)	-2.7 (-8.4 to 3.2)	8.5 (2.8 to 14.4)	-2.3 (-2.8 to -1.6)

	Number of incident cases (all ages, both sexes) with 95% uncertainty intervals (2016)				Annualized rate of change of age-standardized rate (%) with 95% uncertainty intervals							
					1990-2006				2006-2016			
	**Drug-susceptible TB**	**MDR TB**	**XDR TB**	**All HIV-negative TB**	**Drug-susceptible TB**	**MDR TB**	**XDR TB**	**All HIV-negative TB**	**Drug-susceptible TB**	**MDR TB**	**XDR TB**	**All HIV-negative TB**
Costa Rica	448 (379-527)	7 (3-12)	1 (0-1)	455 (385 to 534)	-4.2 (-4.7 to -3.6)	18.3 (13.6 to 23.1)	25.1 (21.3 to 28.5)	-4.1 (-4.6 to -3.5)	-2.8 (-3.4 to -2.2)	-3.5 (-9.7 to 2.7)	6.3 (0.0 to 12.5)	-2.8 (-3.3 to -2.2)
Cote d'Ivoire	31,721 (25,280-40,064)	799 (265-1,824)	5 (2-12)	32,525 (25,771 to 41,319)	-0.7 (-1.4 to -0.1)	7.4 (0.7 to 14.0)	29.8 (24.9 to 33.9)	-0.6 (-1.2 to 0.1)	-1.8 (-2.7 to -1.0)	-2.4 (-13.0 to 6.8)	8.8 (-1.8 to 18.0)	-1.8 (-2.6 to -1.1)
Croatia	531 (468-606)	1 (0-4)	0 (0-1)	533 (469 to 607)	-4.6 (-5.2 to -4.0)	-1.5 (-11.4 to 12.4)	24.3 (19.4 to 28.5)	-4.6 (-5.1 to -4.0)	-5.1 (-5.8 to -4.5)	-10.6 (-28.3 to 2.5)	0.6 (-17.0 to 13.7)	-5.1 (-5.8 to -4.5)
Cuba	713 (597-840)	8 (2-19)	1 (0-2)	721 (602 to 851)	-3.7 (-4.5 to -2.9)	-2.1 (-10.4 to 7.3)	18.1 (12.9 to 22.5)	-3.7 (-4.5 to -2.9)	-1.8 (-2.4 to -1.2)	-0.2 (-14.0 to 12.4)	9.6 (-4.2 to 22.2)	-1.8 (-2.4 to -1.2)
Cyprus	85 (64-113)	0 (0-0)	0 (0-0)	85 (64 to 113)	-1.5 (-2.0 to -1.0)	11.7 (8.4 to 14.9)	23.0 (19.6 to 26.0)	-1.4 (-2.0 to -1.0)	-2.5 (-3.4 to -1.7)	-9.2 (-13.7 to -4.4)	-1.6 (-6.0 to 3.2)	-2.6 (-3.4 to -1.7)
Czech Republic	607 (527-694)	11 (5-21)	2 (1-5)	620 (540 to 709)	-4.1 (-4.8 to -3.5)	1.6 (-6.7 to 13.5)	28.5 (25.9 to 30.9)	-4.1 (-4.7 to -3.5)	-3.5 (-4.1 to -3.0)	-4.3 (-12.6 to 3.7)	7.0 (-1.4 to 14.9)	-3.5 (-4.1 to -3.0)
Democratic Republic of the Congo	265,565 (224,701-308,415)	1,972 (1,012-3,363)	13 (7-22)	267,549 (226,986 to 310,607)	-0.4 (-0.7 to -0.1)	9.3 (4.8 to 13.2)	28.3 (24.5 to 31.7)	-0.3 (-0.7 to -0.0)	-1.4 (-2.0 to -0.9)	-1.9 (-7.6 to 3.9)	9.2 (3.5 to 15.1)	-1.4 (-2.0 to -0.9)
Denmark	334 (255-432)	2 (1-3)	0 (0-0)	336 (257 to 435)	-2.8 (-3.7 to -2.0)	4.9 (1.8 to 7.9)	16.7 (13.6 to 19.4)	-2.8 (-3.7 to -2.0)	-1.6 (-2.6 to -0.6)	3.9 (-0.6 to 8.4)	11.6 (7.0 to 16.0)	-1.6 (-2.5 to -0.6)
Djibouti	3,226 (2,683-3,839)	137 (70-245)	1 (0-2)	3,364 (2,830 to 3,992)	2.3 (1.3 to 3.0)	35.3 (27.7 to 43.7)	36.3 (29.3 to 41.5)	2.5 (1.6 to 3.2)	-0.3 (-1.0 to 0.5)	-0.0 (-9.4 to 10.7)	11.1 (1.7 to 21.9)	-0.3 (-0.9 to 0.3)
Dominica	28 (23-33)	0 (0-1)	0 (0-0)	28 (23 to 33)	-1.0 (-1.4 to -0.5)	4.9 (-9.2 to 18.2)	27.6 (13.8 to 36.4)	-0.9 (-1.4 to -0.5)	-0.7 (-1.3 to -0.1)	-4.0 (-22.7 to 13.8)	5.8 (-12.9 to 23.6)	-0.7 (-1.3 to -0.1)
Dominican Republic	4,671 (3,903-5,531)	24 (3-94)	2 (0-7)	4,697 (3,923 to 5,575)	-4.6 (-5.4 to -3.9)	1.3 (-12.2 to 13.6)	29.5 (15.5 to 37.5)	-4.5 (-5.4 to -3.9)	-1.8 (-2.4 to -1.1)	-5.1 (-22.0 to 13.3)	4.7 (-12.2 to 23.1)	-1.8 (-2.4 to -1.2)
Ecuador	4,351 (3,648-5,132)	237 (66-624)	19 (5-49)	4,607 (3,913 to 5,347)	-5.9 (-6.5 to -5.3)	13.9 (6.0 to 23.3)	39.7 (35.5 to 43.1)	-5.5 (-6.1 to -5.0)	-2.1 (-3.1 to -1.3)	-2.5 (-14.0 to 6.0)	7.2 (-4.2 to 15.8)	-2.1 (-2.7 to -1.5)
Egypt	12,969 (10,250-16,054)	629 (141-1,634)	22 (5-58)	13,621 (10,865 to 16,774)	-3.4 (-4.0 to -2.8)	16.0 (5.9 to 30.0)	28.1 (22.3 to 32.6)	-3.2 (-3.8 to -2.7)	-1.8 (-3.1 to -0.9)	2.2 (-14.4 to 15.7)	10.4 (-6.2 to 23.9)	-1.7 (-2.4 to -0.9)
El Salvador	1,294 (1,118-1,487)	17 (8-31)	1 (1-2)	1,312 (1,136 to 1,505)	-6.2 (-6.6 to -5.8)	9.7 (4.3 to 14.5)	29.7 (24.9 to 33.5)	-6.1 (-6.5 to -5.7)	-1.7 (-2.3 to -1.1)	-3.2 (-10.2 to 4.3)	6.5 (-0.4 to 14.0)	-1.8 (-2.4 to -1.1)
Equatorial Guinea	2,584 (2,097-3,143)	19 (10-33)	0 (0-0)	2,603 (2,115 to 3,164)	-5.1 (-6.0 to -4.3)	4.2 (0.1 to 8.5)	26.7 (22.7 to 30.2)	-5.0 (-6.0 to -4.2)	-2.4 (-2.9 to -1.8)	-2.7 (-8.2 to 2.8)	8.4 (2.9 to 13.9)	-2.4 (-2.9 to -1.8)

	Number of incident cases (all ages, both sexes) with 95% uncertainty intervals (2016)				Annualized rate of change of age-standardized rate (%) with 95% uncertainty intervals							
					1990-2006				2006-2016			
	**Drug-susceptible TB**	**MDR TB**	**XDR TB**	**All HIV-negative TB**	**Drug-susceptible TB**	**MDR TB**	**XDR TB**	**All HIV-negative TB**	**Drug-susceptible TB**	**MDR TB**	**XDR TB**	**All HIV-negative TB**
Eritrea	13,854 (11,172-16,982)	294 (80-741)	2 (1-5)	14,150 (11,423 to 17,330)	0.5 (-0.0 to 1.0)	21.0 (12.7 to 29.4)	30.1 (22.2 to 35.9)	0.6 (0.1 to 1.1)	1.0 (0.2 to 1.8)	2.0 (-9.0 to 12.6)	13.2 (2.2 to 23.8)	1.0 (0.3 to 1.8)
Estonia	199 (166-233)	38 (29-51)	8 (6-11)	245 (206 to 285)	-3.1 (-3.9 to -2.4)	2.7 (-0.2 to 6.0)	50.0 (48.7 to 51.3)	-2.4 (-3.2 to -1.8)	-4.8 (-5.7 to -3.9)	-4.7 (-7.7 to -2.1)	6.5 (3.6 to 9.2)	-4.6 (-5.3 to -3.8)
Ethiopia	214,637 (177,802-259,888)	4,519 (1,307-11,134)	29 (8-72)	219,186 (182,977 to 265,292)	-3.2 (-4.0 to -2.6)	19.9 (13.1 to 27.7)	30.9 (26.7 to 34.7)	-3.1 (-3.9 to -2.5)	-3.3 (-4.3 to -2.2)	-2.0 (-12.9 to 7.3)	9.2 (-1.8 to 18.4)	-3.3 (-4.2 to -2.2)
Federated States of Micronesia	125 (105-146)	1 (0-9)	0 (0-1)	126 (108 to 147)	-2.7 (-3.4 to -2.3)	7.9 (-18.1 to 34.9)	40.0 (7.8 to 52.8)	-2.6 (-2.9 to -2.3)	-1.7 (-2.6 to -0.6)	-5.3 (-42.7 to 29.3)	6.6 (-30.8 to 41.2)	-1.7 (-2.5 to -1.2)
Fiji	494 (436-558)	1 (0-4)	0 (0-0)	495 (437 to 559)	-0.7 (-1.1 to -0.4)	3.3 (-22.6 to 30.0)	20.8 (-12.9 to 32.4)	-0.7 (-1.0 to -0.4)	-0.6 (-1.1 to -0.2)	-3.1 (-39.7 to 36.5)	8.8 (-27.8 to 48.4)	-0.6 (-1.1 to -0.2)
Finland	299 (233-376)	5 (3-7)	1 (0-1)	304 (237 to 384)	-4.1 (-4.8 to -3.5)	9.1 (5.9 to 12.3)	20.2 (17.3 to 22.7)	-4.1 (-4.7 to -3.4)	-1.5 (-2.5 to -0.6)	6.8 (2.7 to 10.6)	14.4 (10.4 to 18.3)	-1.4 (-2.4 to -0.5)
France	4,584 (3,620-5,805)	67 (45-95)	8 (6-12)	4,659 (3,679 to 5,901)	-5.1 (-6.0 to -4.1)	1.4 (-1.2 to 4.1)	26.1 (24.0 to 28.1)	-5.0 (-6.0 to -4.0)	-1.5 (-2.5 to -0.6)	-1.0 (-4.4 to 2.2)	6.7 (3.3 to 9.9)	-1.5 (-2.5 to -0.5)
Gabon	4,705 (3,865-5,708)	34 (18-58)	0 (0-0)	4,739 (3,894 to 5,751)	-1.3 (-2.2 to -0.4)	8.0 (3.7 to 12.0)	26.1 (22.3 to 29.6)	-1.2 (-2.2 to -0.4)	-3.0 (-3.8 to -2.0)	-3.4 (-8.7 to 2.6)	7.8 (2.4 to 13.8)	-3.0 (-3.8 to -2.1)
Georgia	2,464 (2,163-2,818)	399 (293-531)	87 (64-116)	2,950 (2,608 to 3,334)	-0.7 (-1.4 to -0.1)	28.2 (20.3 to 40.3)	52.4 (50.0 to 54.7)	-0.2 (-0.8 to 0.4)	-3.3 (-4.1 to -2.5)	4.2 (-0.2 to 8.7)	15.5 (11.0 to 20.0)	-2.3 (-2.8 to -1.8)
Germany	4,874 (3,752-6,188)	109 (75-152)	14 (9-19)	4,997 (3,838 to 6,338)	-5.9 (-6.7 to -5.0)	0.1 (-2.5 to 2.9)	25.6 (23.8 to 27.4)	-5.8 (-6.7 to -4.9)	0.8 (-0.4 to 1.9)	3.0 (-0.2 to 6.0)	10.6 (7.4 to 13.6)	0.8 (-0.4 to 2.0)
Ghana	43,365 (34,407-55,183)	660 (198-1,726)	4 (1-11)	44,029 (34,874 to 56,001)	-2.9 (-3.7 to -2.2)	10.2 (2.4 to 18.5)	28.1 (20.3 to 33.8)	-2.9 (-3.6 to -2.2)	-2.2 (-3.1 to -1.3)	-3.4 (-14.1 to 8.2)	7.7 (-3.0 to 19.4)	-2.3 (-3.2 to -1.3)
Greece	533 (417-689)	17 (11-27)	2 (1-3)	553 (429 to 710)	-4.5 (-5.3 to -3.7)	19.4 (16.5 to 22.4)	29.1 (26.5 to 31.5)	-4.3 (-5.1 to -3.5)	-1.2 (-2.2 to -0.4)	-2.3 (-6.7 to 2.1)	5.4 (1.0 to 9.8)	-1.2 (-2.1 to -0.5)
Greenland	24 (20-27)	0 (0-0)	0 (0-0)	24 (21 to 27)	-4.8 (-5.1 to -4.4)	-2.4 (-3.8 to -1.1)	36.8 (35.4 to 38.2)	-4.8 (-5.1 to -4.4)	-2.8 (-3.4 to -2.3)	-3.0 (-5.0 to -1.1)	4.7 (2.7 to 6.6)	-2.8 (-3.4 to -2.3)
Grenada	10 (8-12)	0 (0-0)	0 (0-0)	10 (8 to 12)	-0.5 (-1.1 to -0.1)	5.5 (-7.5 to 19.2)	20.0 (5.5 to 28.8)	-0.5 (-1.0 to -0.1)	-1.4 (-2.0 to -0.7)	-5.0 (-23.7 to 12.1)	4.8 (-13.9 to 21.9)	-1.4 (-2.0 to -0.8)
Guam	104 (89-119)	1 (0-7)	0 (0-1)	105 (90 to 121)	-2.7 (-3.3 to -2.3)	11.7 (-2.9 to 40.3)	37.7 (27.2 to 45.0)	-2.6 (-3.0 to -2.2)	0.9 (0.1 to 1.8)	-7.0 (-42.9 to 17.6)	4.9 (-31.0 to 29.5)	0.8 (0.3 to 1.3)

	Number of incident cases (all ages, both sexes) with 95% uncertainty intervals (2016)				Annualized rate of change of age-standardized rate (%) with 95% uncertainty intervals							
					1990-2006				2006-2016			
	**Drug-susceptible TB**	**MDR TB**	**XDR TB**	**All HIV-negative TB**	**Drug-susceptible TB**	**MDR TB**	**XDR TB**	**All HIV-negative TB**	**Drug-susceptible TB**	**MDR TB**	**XDR TB**	**All HIV-negative TB**
Guatemala	2,798 (2,345-3,280)	73 (36-138)	6 (3-11)	2,877 (2,430 to 3,372)	-5.0 (-5.5 to -4.6)	15.5 (11.2 to 20.3)	34.0 (30.5 to 37.1)	-4.8 (-5.3 to -4.4)	-2.4 (-3.4 to -1.5)	-3.6 (-9.9 to 2.2)	6.2 (-0.1 to 12.0)	-2.4 (-3.4 to -1.5)
Guinea	18,770 (15,013-23,402)	291 (81-759)	2 (1-5)	19,063 (15,220 to 23,751)	-0.2 (-0.9 to 0.4)	13.1 (4.7 to 20.9)	27.3 (19.6 to 33.0)	-0.1 (-0.8 to 0.5)	-0.8 (-1.4 to -0.2)	-2.0 (-13.7 to 9.2)	9.2 (-2.6 to 20.3)	-0.8 (-1.4 to -0.2)
Guinea-Bissau	3,702 (2,796-4,800)	57 (15-143)	0 (0-1)	3,760 (2,866 to 4,909)	-1.2 (-2.2 to -0.3)	12.0 (3.8 to 20.2)	29.0 (21.1 to 34.9)	-1.1 (-2.1 to -0.3)	-1.2 (-1.9 to -0.5)	-2.4 (-12.8 to 8.0)	8.7 (-1.7 to 19.1)	-1.2 (-1.9 to -0.6)
Guyana	436 (362-521)	2 (0-9)	0 (0-1)	438 (365 to 524)	0.5 (-0.2 to 1.2)	6.1 (-7.3 to 19.4)	31.4 (18.0 to 39.8)	0.5 (-0.2 to 1.2)	-1.8 (-2.6 to -1.0)	-5.1 (-22.4 to 13.2)	4.7 (-12.6 to 23.0)	-1.8 (-2.6 to -1.0)
Haiti	9,061 (7,538-10,742)	48 (5-200)	4 (0-16)	9,112 (7,589 to 10,832)	-3.5 (-4.3 to -2.8)	2.2 (-10.9 to 14.6)	33.6 (20.7 to 41.7)	-3.5 (-4.3 to -2.8)	-1.8 (-2.7 to -0.7)	-4.9 (-22.9 to 13.3)	4.9 (-13.1 to 23.1)	-1.8 (-2.7 to -0.8)
Honduras	3,086 (2,655-3,573)	51 (25-89)	4 (2-7)	3,141 (2,711 to 3,634)	-2.2 (-2.5 to -1.8)	18.7 (14.4 to 23.1)	35.4 (31.9 to 38.4)	-2.0 (-2.4 to -1.7)	-2.4 (-2.8 to -1.9)	-2.9 (-8.9 to 2.9)	6.8 (0.9 to 12.7)	-2.4 (-2.8 to -1.9)
Hungary	892 (774-1,015)	23 (5-69)	5 (1-15)	920 (809 to 1,041)	-3.8 (-4.5 to -3.2)	19.3 (9.3 to 32.7)	35.3 (27.7 to 40.8)	-3.6 (-4.2 to -3.0)	-4.9 (-5.8 to -4.0)	-7.0 (-26.5 to 10.8)	4.2 (-15.3 to 22.0)	-4.9 (-5.5 to -4.4)
Iceland	29 (22-38)	0 (0-0)	0 (0-0)	29 (22 to 39)	-3.1 (-3.7 to -2.6)	-0.3 (-3.5 to 2.8)	6.2 (2.6 to 9.3)	-3.1 (-3.7 to -2.6)	-2.4 (-3.2 to -1.7)	-8.5 (-13.4 to -3.8)	-0.8 (-5.8 to 3.8)	-2.4 (-3.2 to -1.7)
India	2,862,029 (2,644,051-3,117,771)	115,087 (104,178-126,881)	2,821 (2,553-3,110)	2,979,936 (2,756,776 to 3,248,951)	-1.6 (-1.8 to -1.4)	25.6 (25.2 to 26.1)	43.0 (42.4 to 43.6)	-1.3 (-1.6 to -1.1)	-1.7 (-1.8 to -1.5)	-1.4 (-1.9 to -0.8)	8.3 (7.8 to 8.9)	-1.6 (-1.8 to -1.4)
Indonesia	619,671 (527,200-723,052)	2,967 (1,583-5,988)	271 (144-546)	622,909 (530,078 to 726,767)	-3.4 (-4.1 to -2.9)	9.6 (5.1 to 14.3)	42.7 (38.7 to 46.6)	-3.4 (-4.1 to -2.8)	-2.6 (-2.8 to -2.4)	-9.9 (-17.1 to -2.7)	2.0 (-5.2 to 9.2)	-2.6 (-2.8 to -2.4)
Iran	13,195 (11,365-15,156)	140 (46-326)	5 (2-12)	13,340 (11,564 to 15,285)	-0.1 (-0.5 to 0.4)	23.3 (10.9 to 38.6)	23.7 (11.9 to 31.6)	0.0 (-0.4 to 0.4)	-2.9 (-3.5 to -2.3)	-4.3 (-19.8 to 16.6)	3.9 (-11.6 to 24.9)	-2.9 (-3.4 to -2.5)
Iraq	15,010 (10,302-21,084)	253 (68-638)	9 (2-23)	15,272 (10,464 to 21,462)	-0.8 (-1.5 to -0.1)	24.2 (12.6 to 38.4)	32.5 (21.4 to 39.8)	-0.7 (-1.4 to -0.0)	-3.1 (-4.2 to -2.1)	-3.0 (-19.9 to 16.5)	5.2 (-11.7 to 24.7)	-3.1 (-4.2 to -2.2)
Ireland	298 (227-390)	3 (2-5)	0 (0-1)	302 (232 to 395)	-2.4 (-3.1 to -1.7)	4.8 (1.7 to 8.0)	24.1 (21.4 to 26.7)	-2.4 (-3.1 to -1.6)	-2.9 (-3.8 to -2.1)	-1.5 (-5.7 to 2.7)	6.2 (1.9 to 10.3)	-2.9 (-3.8 to -2.0)
Israel	328 (249-426)	15 (10-22)	2 (1-3)	345 (263 to 446)	-3.7 (-4.5 to -2.9)	1.4 (-1.6 to 4.4)	30.9 (28.9 to 32.9)	-3.5 (-4.3 to -2.8)	-2.4 (-3.3 to -1.4)	-2.7 (-6.1 to 0.8)	5.0 (1.5 to 8.5)	-2.3 (-3.2 to -1.4)

	Number of incident cases (all ages, both sexes) with 95% uncertainty intervals (2016)				Annualized rate of change of age-standardized rate (%) with 95% uncertainty intervals							
					1990-2006				2006-2016			
	**Drug-susceptible TB**	**MDR TB**	**XDR TB**	**All HIV-negative TB**	**Drug-susceptible TB**	**MDR TB**	**XDR TB**	**All HIV-negative TB**	**Drug-susceptible TB**	**MDR TB**	**XDR TB**	**All HIV-negative TB**
Italy	3,315 (2,735-3,960)	88 (61-122)	11 (8-15)	3,414 (2,824 to 4,092)	-4.8 (-5.6 to -3.9)	0.6 (-1.9 to 3.3)	29.8 (28.1 to 31.3)	-4.6 (-5.4 to -3.8)	-1.7 (-2.5 to -1.0)	-3.0 (-6.3 to 0.3)	4.7 (1.4 to 8.0)	-1.7 (-2.5 to -1.0)
Jamaica	227 (184-276)	4 (1-15)	0 (0-1)	231 (191 to 281)	-2.8 (-3.8 to -2.0)	22.0 (10.2 to 35.9)	24.7 (12.1 to 33.0)	-2.7 (-3.6 to -2.0)	-0.8 (-1.9 to 0.4)	-2.3 (-20.3 to 16.6)	7.5 (-10.5 to 26.4)	-0.8 (-1.8 to 0.2)
Japan	25,482 (23,079-28,085)	231 (201-268)	29 (25-34)	25,743 (23,315 to 28,385)	-5.4 (-5.7 to -5.1)	0.8 (-0.0 to 1.6)	28.6 (27.4 to 29.7)	-5.4 (-5.7 to -5.1)	-3.1 (-3.7 to -2.4)	-4.4 (-5.7 to -3.1)	3.3 (1.9 to 4.6)	-3.1 (-3.7 to -2.4)
Jordan	476 (340-637)	34 (6-108)	1 (0-4)	512 (384 to 670)	-3.0 (-3.8 to -2.3)	20.2 (10.8 to 35.8)	28.7 (23.1 to 33.1)	-2.6 (-3.4 to -2.0)	-3.6 (-5.7 to -2.2)	-1.9 (-21.3 to 12.1)	6.3 (-13.1 to 20.3)	-3.5 (-4.5 to -2.4)
Kazakhstan	11,456 (9,840-13,327)	3,047 (2,246-3,990)	668 (492-874)	15,170 (13,280 to 17,223)	-1.2 (-2.3 to -0.2)	27.2 (19.3 to 38.5)	63.4 (60.3 to 65.9)	0.6 (0.0 to 1.1)	-6.1 (-7.8 to -4.2)	-7.5 (-12.5 to -1.7)	3.8 (-1.2 to 9.5)	-6.2 (-6.7 to -5.6)
Kenya	76,504 (64,364-90,273)	864 (635-1,171)	6 (4-8)	77,373 (65,100 to 91,472)	2.7 (2.4 to 3.1)	27.6 (25.8 to 29.3)	22.7 (20.8 to 24.7)	2.8 (2.4 to 3.1)	0.3 (-0.2 to 0.9)	1.1 (-1.4 to 3.7)	12.2 (9.8 to 14.9)	0.3 (-0.2 to 0.9)
Kiribati	294 (248-338)	4 (0-29)	0 (0-3)	299 (260 to 340)	-1.4 (-2.2 to -1.1)	8.3 (-16.4 to 37.3)	45.6 (12.7 to 58.1)	-1.4 (-1.6 to -1.1)	-0.9 (-1.7 to 0.1)	-3.1 (-41.6 to 33.5)	8.9 (-29.7 to 45.4)	-1.0 (-1.4 to -0.5)
Kuwait	955 (603-1,431)	14 (6-29)	0 (0-1)	970 (611 to 1,452)	-1.3 (-2.1 to -0.6)	21.5 (11.8 to 35.3)	27.8 (20.0 to 33.5)	-1.2 (-2.0 to -0.5)	-4.5 (-5.5 to -3.5)	-4.3 (-17.7 to 9.2)	3.9 (-9.5 to 17.4)	-4.5 (-5.5 to -3.5)
Kyrgyzstan	4,487 (2,866-5,863)	1,658 (723-2,923)	363 (158-640)	6,508 (5,716 to 7,379)	-1.6 (-4.2 to -0.1)	41.9 (33.6 to 54.9)	63.0 (57.2 to 66.8)	0.6 (0.1 to 1.1)	-1.3 (-6.9 to 4.2)	-1.6 (-13.0 to 11.2)	9.7 (-1.7 to 22.5)	-1.0 (-1.5 to -0.5)
Laos	8,032 (6,600-9,745)	39 (3-169)	4 (0-15)	8,074 (6,665 to 9,805)	-3.0 (-3.6 to -2.4)	10.3 (-3.5 to 24.3)	40.7 (25.5 to 50.3)	-2.9 (-3.5 to -2.4)	-5.3 (-6.0 to -4.4)	-13.0 (-33.9 to 7.0)	-1.1 (-22.0 to 19.0)	-5.3 (-6.1 to -4.6)
Latvia	583 (505-666)	50 (37-66)	11 (8-14)	644 (556 to 732)	-0.5 (-1.1 to 0.1)	2.3 (-0.8 to 5.9)	50.6 (49.4 to 51.7)	-0.3 (-0.8 to 0.3)	-3.8 (-4.4 to -3.2)	-6.9 (-10.1 to -4.1)	4.3 (1.2 to 7.2)	-4.0 (-4.5 to -3.4)
Lebanon	823 (688-969)	19 (2-72)	1 (0-3)	843 (709 to 983)	-6.4 (-6.8 to -5.9)	11.4 (-0.4 to 26.3)	24.8 (15.2 to 31.3)	-6.3 (-6.7 to -5.8)	-3.3 (-4.2 to -2.7)	-0.7 (-20.3 to 15.1)	7.5 (-12.1 to 23.3)	-3.2 (-3.7 to -2.7)
Lesotho	18,551 (13,143-25,645)	635 (341-1,075)	4 (2-7)	19,190 (13,571 to 26,500)	1.2 (-0.1 to 2.4)	30.7 (22.5 to 40.1)	38.0 (30.8 to 43.4)	1.4 (0.0 to 2.6)	0.7 (-1.6 to 2.8)	1.4 (-9.4 to 13.4)	12.6 (1.7 to 24.5)	0.7 (-1.6 to 2.7)
Liberia	7,160 (5,914-8,599)	117 (33-275)	1 (0-2)	7,277 (6,023 to 8,781)	-1.9 (-2.4 to -1.3)	11.5 (3.7 to 19.6)	28.8 (21.1 to 34.8)	-1.8 (-2.3 to -1.3)	-2.2 (-2.9 to -1.6)	-3.3 (-14.7 to 8.0)	7.8 (-3.5 to 19.1)	-2.3 (-2.9 to -1.6)
Libya	1,817 (1,226-2,594)	33 (3-152)	1 (0-5)	1,851 (1,246 to 2,634)	-1.7 (-2.4 to -1.0)	10.6 (-3.7 to 24.8)	25.5 (9.1 to 34.7)	-1.7 (-2.4 to -0.9)	-0.3 (-1.6 to 0.8)	4.0 (-15.3 to 23.6)	12.2 (-7.1 to 31.8)	-0.3 (-1.3 to 0.8)

	Number of incident cases (all ages, both sexes) with 95% uncertainty intervals (2016)				Annualized rate of change of age-standardized rate (%) with 95% uncertainty intervals							
					1990-2006				2006-2016			
	**Drug-susceptible TB**	**MDR TB**	**XDR TB**	**All HIV-negative TB**	**Drug-susceptible TB**	**MDR TB**	**XDR TB**	**All HIV-negative TB**	**Drug-susceptible TB**	**MDR TB**	**XDR TB**	**All HIV-negative TB**
Lithuania	1,171 (1,022-1,329)	168 (135-208)	37 (30-46)	1,376 (1,203 to 1,556)	-0.7 (-1.2 to -0.1)	6.6 (3.3 to 10.4)	53.2 (52.3 to 54.2)	-0.1 (-0.6 to 0.5)	-3.6 (-4.2 to -3.0)	-3.1 (-5.0 to -1.3)	8.2 (6.3 to 10.0)	-3.3 (-3.9 to -2.8)
Luxembourg	42 (32-57)	0 (0-0)	0 (0-0)	43 (32 to 57)	-3.9 (-4.5 to -3.2)	4.1 (0.8 to 7.3)	11.8 (8.4 to 14.6)	-3.9 (-4.5 to -3.2)	-2.5 (-3.5 to -1.6)	6.5 (1.9 to 11.3)	14.1 (9.6 to 18.9)	-2.5 (-3.5 to -1.6)
Macedonia	462 (400-531)	5 (2-11)	1 (0-2)	468 (406 to 536)	-4.3 (-4.6 to -4.0)	13.7 (3.4 to 27.6)	31.1 (20.7 to 38.2)	-4.2 (-4.6 to -3.9)	-4.0 (-4.6 to -3.4)	0.8 (-14.8 to 18.5)	12.0 (-3.6 to 29.7)	-3.9 (-4.5 to -3.4)
Madagascar	32,796 (25,995-42,158)	271 (87-682)	2 (1-4)	33,069 (26,150 to 42,490)	-1.7 (-2.3 to -1.0)	18.6 (11.6 to 26.2)	20.8 (14.9 to 25.4)	-1.6 (-2.3 to -1.0)	-1.4 (-2.5 to -0.3)	-0.3 (-11.4 to 9.7)	10.9 (-0.2 to 20.9)	-1.4 (-2.5 to -0.3)
Malawi	34,769 (28,528-42,421)	308 (121-640)	2 (1-4)	35,079 (28,763 to 42,789)	2.6 (1.9 to 3.3)	25.9 (19.0 to 34.2)	23.1 (17.2 to 28.0)	2.7 (2.0 to 3.3)	-2.5 (-3.8 to -1.3)	-2.0 (-14.0 to 9.0)	9.1 (-2.8 to 20.2)	-2.5 (-3.8 to -1.3)
Malaysia	16,509 (12,936-21,202)	125 (54-249)	11 (5-23)	16,646 (13,025 to 21,362)	-2.6 (-3.3 to -2.0)	20.3 (9.5 to 32.7)	33.7 (22.6 to 40.9)	-2.6 (-3.1 to -1.9)	-2.1 (-2.9 to -1.2)	-6.2 (-21.7 to 13.3)	5.7 (-9.8 to 25.2)	-2.1 (-2.9 to -1.3)
Maldives	177 (137-230)	1 (0-4)	0 (0-0)	178 (138 to 231)	-6.7 (-7.4 to -6.2)	7.3 (-6.9 to 21.4)	33.5 (18.2 to 43.0)	-6.7 (-7.3 to -6.1)	-2.5 (-3.4 to -1.5)	-11.1 (-29.5 to 9.1)	0.8 (-17.6 to 21.0)	-2.5 (-3.4 to -1.7)
Mali	7,766 (6,281-9,435)	122 (38-314)	1 (0-2)	7,888 (6,401 to 9,570)	-4.7 (-5.3 to -4.3)	8.6 (0.4 to 16.7)	22.1 (14.4 to 27.7)	-4.6 (-5.2 to -4.2)	-2.6 (-3.3 to -1.9)	-3.6 (-14.9 to 6.9)	7.5 (-3.7 to 18.1)	-2.6 (-3.2 to -2.0)
Malta	25 (18-33)	0 (0-0)	0 (0-0)	25 (18 to 33)	-2.7 (-3.3 to -2.1)	4.6 (1.3 to 7.7)	6.1 (2.6 to 9.0)	-2.7 (-3.3 to -2.1)	-1.4 (-2.3 to -0.5)	-8.8 (-13.6 to -4.0)	-1.2 (-5.9 to 3.7)	-1.4 (-2.3 to -0.5)
Marshall Islands	136 (116-159)	2 (0-9)	0 (0-1)	139 (121 to 161)	-1.1 (-2.0 to -0.7)	20.6 (5.4 to 50.6)	47.9 (30.4 to 57.3)	-1.0 (-1.3 to -0.6)	-2.0 (-3.0 to -0.7)	-7.6 (-36.3 to 24.6)	4.3 (-24.4 to 36.6)	-2.2 (-2.6 to -1.7)
Mauritania	2,950 (2,398-3,590)	47 (13-110)	0 (0-1)	2,997 (2,430 to 3,642)	-3.4 (-3.9 to -2.9)	9.9 (2.1 to 18.3)	23.7 (16.6 to 29.2)	-3.3 (-3.8 to -2.8)	-2.3 (-2.9 to -1.6)	-3.4 (-13.9 to 7.2)	7.7 (-2.8 to 18.3)	-2.3 (-2.9 to -1.7)
Mauritius	257 (202-329)	1 (0-3)	0 (0-0)	258 (203 to 330)	-4.1 (-4.7 to -3.4)	15.1 (4.1 to 28.0)	21.5 (10.3 to 28.6)	-4.1 (-4.7 to -3.4)	-0.5 (-1.3 to 0.3)	-2.8 (-21.0 to 14.6)	9.1 (-9.1 to 26.5)	-0.5 (-1.3 to 0.3)
Mexico	22,175 (18,770-26,011)	487 (385-596)	38 (30-47)	22,700 (19,191 to 26,607)	-1.1 (-1.5 to -0.6)	26.2 (25.0 to 27.4)	31.3 (29.9 to 32.5)	-0.9 (-1.4 to -0.5)	-1.6 (-1.9 to -1.3)	-2.5 (-4.1 to -1.0)	7.3 (5.7 to 8.8)	-1.6 (-1.9 to -1.3)
Moldova	2,005 (1,670-2,361)	816 (589-1,082)	179 (129-237)	3,000 (2,616 to 3,424)	0.7 (-0.2 to 1.4)	39.0 (35.1 to 43.7)	60.2 (58.2 to 62.0)	2.5 (2.0 to 3.1)	-4.5 (-6.1 to -2.9)	-2.1 (-6.1 to 1.5)	9.2 (5.2 to 12.8)	-3.4 (-4.0 to -2.8)

	Number of incident cases (all ages, both sexes) with 95% uncertainty intervals (2016)				Annualized rate of change of age-standardized rate (%) with 95% uncertainty intervals							
					1990-2006				2006-2016			
	**Drug-susceptible TB**	**MDR TB**	**XDR TB**	**All HIV-negative TB**	**Drug-susceptible TB**	**MDR TB**	**XDR TB**	**All HIV-negative TB**	**Drug-susceptible TB**	**MDR TB**	**XDR TB**	**All HIV-negative TB**
Mongolia	4,812 (4,112-5,490)	175 (36-505)	38 (8-111)	5,025 (4,420 to 5,702)	0.7 (0.4 to 1.0)	19.5 (11.3 to 31.3)	52.1 (48.3 to 55.4)	0.9 (0.6 to 1.2)	-3.8 (-4.8 to -3.1)	-0.6 (-19.1 to 12.1)	10.6 (-7.8 to 23.4)	-3.7 (-4.1 to -3.2)
Montenegro	100 (85-117)	0 (0-0)	0 (0-0)	100 (85 to 117)	-1.3 (-1.6 to -1.0)	6.6 (-6.4 to 21.4)	19.0 (6.1 to 27.3)	-1.3 (-1.6 to -1.0)	-2.1 (-2.6 to -1.6)	-4.9 (-26.1 to 15.2)	6.4 (-14.8 to 26.4)	-2.1 (-2.6 to -1.6)
Morocco	59,979 (40,674-85,213)	783 (244-1,863)	28 (9-66)	60,789 (41,372 to 85,931)	-2.1 (-2.8 to -1.4)	22.0 (10.7 to 36.0)	38.7 (26.6 to 46.5)	-2.0 (-2.7 to -1.4)	-2.0 (-3.1 to -1.1)	-2.8 (-18.1 to 17.3)	5.4 (-9.9 to 25.5)	-2.0 (-3.1 to -1.1)
Mozambique	66,745 (51,885-85,439)	2,490 (847-5,706)	16 (5-37)	69,251 (54,305 to 88,960)	0.5 (-0.5 to 1.4)	18.5 (12.3 to 25.8)	33.1 (29.4 to 36.3)	0.7 (-0.2 to 1.6)	-1.1 (-2.3 to 0.2)	-1.1 (-12.3 to 7.9)	10.1 (-1.2 to 19.0)	-1.1 (-2.2 to 0.2)
Myanmar	88,288 (66,880-116,274)	4,257 (1,629-9,278)	388 (149-846)	92,933 (71,050 to 122,100)	-4.1 (-5.0 to -3.4)	16.4 (7.3 to 29.5)	50.2 (46.2 to 53.5)	-3.9 (-4.7 to -3.1)	-4.2 (-5.2 to -3.2)	-3.0 (-14.0 to 6.3)	8.9 (-2.1 to 18.2)	-4.1 (-5.0 to -3.2)
Namibia	12,957 (9,691-17,589)	582 (330-924)	4 (2-6)	13,543 (10,107 to 18,293)	1.2 (-0.0 to 2.3)	32.3 (24.2 to 41.1)	40.1 (32.7 to 45.4)	1.5 (0.3 to 2.5)	-3.4 (-4.9 to -1.8)	-2.8 (-12.5 to 9.5)	8.3 (-1.3 to 20.7)	-3.4 (-4.8 to -1.8)
Nepal	34,521 (30,238-38,956)	890 (680-1,134)	22 (17-28)	35,433 (31,028 to 39,985)	-3.0 (-3.3 to -2.7)	6.2 (4.3 to 8.0)	38.3 (36.7 to 39.8)	-2.9 (-3.2 to -2.6)	-4.0 (-4.4 to -3.5)	-3.3 (-5.7 to -0.9)	6.4 (4.0 to 8.8)	-3.9 (-4.4 to -3.5)
Netherlands	842 (658-1,074)	12 (8-17)	1 (1-2)	856 (669 to 1,091)	-3.2 (-4.1 to -2.4)	2.7 (-0.2 to 5.7)	21.7 (19.2 to 24.0)	-3.2 (-4.1 to -2.4)	-1.8 (-2.8 to -0.8)	2.4 (-1.6 to 6.2)	10.1 (6.0 to 13.9)	-1.7 (-2.7 to -0.7)
New Zealand	267 (228-310)	3 (1-9)	0 (0-1)	271 (230 to 313)	-3.1 (-3.8 to -2.3)	0.7 (-7.8 to 11.5)	23.3 (18.1 to 27.2)	-3.0 (-3.8 to -2.3)	-2.1 (-2.8 to -1.4)	-0.1 (-13.2 to 12.1)	7.6 (-5.5 to 19.8)	-2.1 (-2.7 to -1.4)
Nicaragua	2,078 (1,774-2,396)	23 (11-42)	2 (1-3)	2,102 (1,793 to 2,424)	-3.7 (-4.1 to -3.4)	8.2 (3.7 to 12.8)	31.9 (28.3 to 35.0)	-3.7 (-4.0 to -3.3)	-2.7 (-3.2 to -2.2)	-3.0 (-9.4 to 3.7)	6.8 (0.4 to 13.5)	-2.7 (-3.1 to -2.2)
Niger	18,713 (15,308-22,639)	305 (88-807)	2 (1-5)	19,020 (15,647 to 22,946)	-2.0 (-2.6 to -1.5)	11.6 (3.8 to 19.5)	26.2 (18.2 to 32.0)	-1.9 (-2.5 to -1.4)	-1.6 (-2.3 to -1.0)	-2.8 (-14.5 to 8.1)	8.4 (-3.4 to 19.3)	-1.6 (-2.3 to -1.0)
Nigeria	282,601 (228,683-346,770)	9,180 (3,931-18,673)	59 (25-120)	291,840 (237,243 to 356,937)	-1.7 (-2.5 to -1.0)	26.9 (20.5 to 34.4)	33.2 (28.2 to 37.4)	-1.5 (-2.3 to -0.9)	-3.8 (-4.6 to -2.9)	-4.3 (-14.6 to 5.9)	6.9 (-3.5 to 17.0)	-3.8 (-4.4 to -2.9)
North Korea	73,689 (65,357-82,742)	1,663 (969-2,555)	152 (88-233)	75,505 (67,027 to 84,573)	-0.3 (-0.8 to 0.1)	32.1 (28.1 to 35.9)	46.4 (43.0 to 49.4)	-0.2 (-0.6 to 0.2)	0.7 (0.1 to 1.2)	0.0 (-5.2 to 5.5)	11.9 (6.7 to 17.4)	0.7 (0.1 to 1.2)

	Number of incident cases (all ages, both sexes) with 95% uncertainty intervals (2016)				Annualized rate of change of age-standardized rate (%) with 95% uncertainty intervals							
					1990-2006				2006-2016			
	**Drug-susceptible TB**	**MDR TB**	**XDR TB**	**All HIV-negative TB**	**Drug-susceptible TB**	**MDR TB**	**XDR TB**	**All HIV-negative TB**	**Drug-susceptible TB**	**MDR TB**	**XDR TB**	**All HIV-negative TB**
Northern Mariana Islands	59 (47-72)	2 (0-8)	0 (0-1)	60 (49 to 74)	-3.1 (-4.0 to -2.5)	8.2 (-5.7 to 38.9)	42.4 (33.7 to 48.4)	-2.8 (-3.1 to -2.4)	0.0 (-1.3 to 1.6)	-8.8 (-36.5 to 11.1)	3.1 (-24.6 to 23.0)	-0.3 (-0.8 to 0.2)
Norway	293 (222-376)	6 (4-9)	1 (0-1)	300 (227 to 387)	-0.6 (-1.2 to 0.0)	2.2 (-0.4 to 5.3)	26.7 (24.1 to 29.0)	-0.5 (-1.2 to 0.1)	-1.1 (-2.1 to -0.2)	-0.3 (-4.4 to 3.6)	7.4 (3.2 to 11.2)	-1.0 (-2.1 to -0.1)
Oman	579 (381-847)	10 (3-23)	0 (0-1)	589 (388 to 866)	-1.8 (-2.5 to -1.3)	2.0 (-8.4 to 16.7)	19.9 (14.9 to 24.1)	-1.8 (-2.5 to -1.2)	-0.8 (-2.0 to 0.2)	3.4 (-8.7 to 15.3)	11.6 (-0.5 to 23.5)	-0.8 (-1.8 to 0.2)
Pakistan	386,429 (308,537-483,642)	14,706 (10,703-19,524)	360 (262-479)	401,496 (320,116 to 502,636)	0.1 (-0.4 to 0.7)	32.1 (30.3 to 34.0)	43.4 (41.2 to 45.3)	0.4 (-0.1 to 0.9)	-2.3 (-3.1 to -1.6)	-2.5 (-5.1 to 0.1)	7.2 (4.6 to 9.8)	-2.3 (-3.1 to -1.6)
Palestine	144 (96-211)	3 (0-12)	0 (0-0)	147 (98 to 217)	-4.0 (-4.9 to -3.2)	8.2 (-6.0 to 24.3)	14.4 (-1.1 to 24.0)	-3.9 (-4.8 to -3.2)	-3.2 (-4.4 to -2.2)	0.9 (-17.5 to 19.7)	9.1 (-9.3 to 27.9)	-3.2 (-4.2 to -2.1)
Panama	1,384 (1,175-1,621)	18 (9-33)	1 (1-3)	1,403 (1,189 to 1,638)	-2.2 (-2.8 to -1.7)	13.4 (8.6 to 18.0)	32.5 (27.8 to 36.3)	-2.1 (-2.7 to -1.6)	-1.6 (-2.2 to -1.1)	-2.8 (-9.4 to 3.9)	7.0 (0.4 to 13.7)	-1.6 (-2.2 to -1.1)
Papua New Guinea	9,214 (7,786-10,521)	399 (64-1,276)	36 (6-116)	9,649 (8,415 to 10,949)	-1.4 (-4.1 to -0.6)	23.8 (7.5 to 55.2)	50.5 (29.1 to 61.1)	-0.9 (-1.5 to -0.5)	-2.3 (-3.8 to 2.2)	-7.4 (-30.7 to 28.1)	4.5 (-18.8 to 40.0)	-2.6 (-3.1 to -2.0)
Paraguay	2,463 (2,068-2,897)	32 (24-42)	3 (2-3)	2,497 (2,097 to 2,934)	-0.6 (-1.2 to -0.1)	17.2 (15.5 to 18.8)	32.7 (31.0 to 34.3)	-0.5 (-1.1 to -0.0)	-0.9 (-1.5 to -0.2)	-2.2 (-4.5 to 0.2)	7.6 (5.3 to 10.0)	-0.9 (-1.6 to -0.2)
Peru	17,063 (15,007-19,264)	891 (694-1,136)	70 (55-90)	18,025 (15,822 to 20,350)	-6.4 (-6.7 to -6.0)	5.2 (0.5 to 11.1)	44.3 (42.6 to 46.0)	-6.1 (-6.5 to -5.7)	-4.6 (-5.3 to -3.9)	-3.8 (-7.1 to -0.5)	6.0 (2.7 to 9.3)	-4.6 (-5.2 to -3.9)
Philippines	221,067 (192,440-253,315)	6,309 (2,008-15,569)	575 (183-1,420)	227,951 (200,365 to 259,332)	-2.6 (-3.4 to -2.1)	26.5 (16.2 to 39.6)	48.4 (39.2 to 55.1)	-2.4 (-2.9 to -1.9)	1.3 (0.5 to 2.5)	-1.5 (-19.5 to 16.8)	10.4 (-7.6 to 28.7)	1.2 (0.7 to 1.8)
Poland	6,003 (5,259-6,743)	30 (17-48)	7 (4-10)	6,039 (5,294 to 6,792)	-4.0 (-4.7 to -3.4)	-0.5 (-8.1 to 10.0)	26.7 (23.9 to 29.2)	-4.0 (-4.6 to -3.3)	-2.6 (-3.1 to -2.2)	-4.7 (-11.0 to 1.7)	6.6 (0.2 to 13.0)	-2.6 (-3.1 to -2.2)
Portugal	1,716 (1,295-2,273)	19 (13-28)	2 (2-4)	1,738 (1,312 to 2,300)	-3.3 (-4.2 to -2.3)	-1.3 (-3.8 to 1.6)	31.3 (29.1 to 33.3)	-3.2 (-4.2 to -2.3)	-2.8 (-3.6 to -1.7)	-3.6 (-7.3 to -0.0)	4.0 (0.3 to 7.7)	-2.8 (-3.6 to -1.7)
Puerto Rico	124 (100-150)	1 (0-2)	0 (0-0)	124 (101 to 152)	-6.0 (-6.6 to -5.5)	10.1 (0.5 to 22.0)	16.4 (7.2 to 22.9)	-6.0 (-6.5 to -5.4)	-2.1 (-2.9 to -1.3)	-9.1 (-26.0 to 6.8)	0.7 (-16.2 to 16.6)	-2.2 (-3.0 to -1.4)
Qatar	862 (541-1,300)	12 (3-34)	0 (0-1)	875 (549 to 1,318)	-4.6 (-5.4 to -3.9)	4.4 (-5.8 to 18.5)	25.3 (18.6 to 30.4)	-4.6 (-5.4 to -3.8)	-1.4 (-2.6 to -0.3)	2.9 (-13.5 to 17.0)	11.1 (-5.3 to 25.2)	-1.4 (-2.6 to -0.3)
Romania	12,307 (10,882-13,768)	313 (137-617)	69 (30-135)	12,689 (11,253 to 14,195)	1.1 (0.5 to 1.8)	8.6 (-0.1 to 18.5)	46.3 (40.8 to 50.7)	1.3 (0.6 to 1.9)	-3.6 (-4.2 to -3.0)	-4.3 (-14.8 to 6.3)	6.9 (-3.5 to 17.5)	-3.6 (-4.1 to -3.0)

	Number of incident cases (all ages, both sexes) with 95% uncertainty intervals (2016)				Annualized rate of change of age-standardized rate (%) with 95% uncertainty intervals							
					1990-2006				2006-2016			
	**Drug-susceptible TB**	**MDR TB**	**XDR TB**	**All HIV-negative TB**	**Drug-susceptible TB**	**MDR TB**	**XDR TB**	**All HIV-negative TB**	**Drug-susceptible TB**	**MDR TB**	**XDR TB**	**All HIV-negative TB**
Russia	77,428 (65,749-90,061)	12,895 (8,846-17,596)	2,825 (1,938-3,855)	93,148 (80,624 to 106,585)	1.6 (1.0 to 2.2)	7.5 (4.3 to 11.3)	54.1 (52.8 to 55.2)	2.1 (1.6 to 2.7)	-3.0 (-4.0 to -2.2)	-0.0 (-3.9 to 3.3)	11.2 (7.3 to 14.5)	-2.4 (-3.1 to -1.8)
Rwanda	23,792 (19,091-30,053)	412 (210-724)	3 (1-5)	24,206 (19,389 to 30,593)	-3.3 (-3.8 to -2.7)	24.9 (17.5 to 33.1)	28.7 (21.4 to 34.2)	-3.2 (-3.7 to -2.7)	-1.7 (-2.8 to -0.5)	-2.2 (-11.3 to 9.4)	9.0 (-0.2 to 20.5)	-1.7 (-2.9 to -0.5)
Saint Lucia	49 (41-59)	0 (0-1)	0 (0-0)	49 (41 to 59)	-1.0 (-1.4 to -0.6)	5.0 (-7.5 to 18.2)	26.8 (13.2 to 35.4)	-0.9 (-1.3 to -0.5)	-2.4 (-3.2 to -1.8)	-5.9 (-23.9 to 12.8)	3.9 (-14.1 to 22.6)	-2.5 (-3.1 to -1.9)
Saint Vincent and the Grenadines	29 (23-35)	0 (0-1)	0 (0-0)	29 (23 to 35)	-0.7 (-1.3 to -0.2)	5.6 (-6.6 to 17.7)	25.8 (12.9 to 34.4)	-0.7 (-1.3 to -0.2)	-1.1 (-1.7 to -0.5)	-4.8 (-23.0 to 11.3)	5.0 (-13.2 to 21.0)	-1.1 (-1.7 to -0.5)
Samoa	54 (46-64)	0 (0-1)	0 (0-0)	54 (46 to 64)	-2.5 (-2.8 to -2.2)	0.9 (-27.1 to 28.6)	17.9 (-15.9 to 30.2)	-2.5 (-2.8 to -2.2)	-1.7 (-2.2 to -1.2)	-1.7 (-39.1 to 34.8)	10.2 (-27.2 to 46.7)	-1.7 (-2.2 to -1.2)
Sao Tome and Principe	148 (120-183)	2 (1-6)	0 (0-0)	150 (122 to 185)	-1.1 (-1.6 to -0.6)	12.4 (4.4 to 20.3)	24.3 (16.7 to 30.0)	-1.0 (-1.5 to -0.5)	-2.4 (-3.1 to -1.6)	-3.3 (-14.2 to 8.5)	7.8 (-3.1 to 19.7)	-2.4 (-3.1 to -1.6)
Saudi Arabia	11,250 (10,092-12,553)	306 (117-724)	11 (4-26)	11,567 (10,502 to 12,872)	-0.5 (-0.7 to -0.2)	25.6 (19.1 to 31.9)	32.1 (26.7 to 37.4)	-0.3 (-0.5 to -0.1)	-0.8 (-1.2 to -0.4)	0.2 (-8.6 to 9.4)	8.4 (-0.4 to 17.6)	-0.7 (-1.0 to -0.5)
Senegal	20,421 (16,739-24,918)	184 (80-374)	1 (1-2)	20,607 (16,872 to 25,179)	-1.6 (-2.2 to -1.2)	20.0 (12.2 to 27.5)	23.9 (17.0 to 29.3)	-1.6 (-2.1 to -1.1)	-2.1 (-2.7 to -1.5)	-2.6 (-12.5 to 8.0)	8.5 (-1.3 to 19.2)	-2.1 (-2.7 to -1.5)
Serbia	1,757 (1,539-1,988)	12 (4-25)	3 (1-5)	1,771 (1,554 to 2,001)	-1.7 (-2.0 to -1.4)	8.4 (-0.8 to 23.3)	27.5 (23.4 to 31.2)	-1.7 (-2.0 to -1.4)	-3.1 (-3.7 to -2.6)	0.3 (-12.2 to 11.6)	11.6 (-0.9 to 22.8)	-3.1 (-3.6 to -2.6)
Seychelles	30 (23-39)	0 (0-0)	0 (0-0)	30 (23 to 39)	-2.2 (-2.8 to -1.7)	1.6 (-12.1 to 15.7)	11.2 (-4.1 to 20.5)	-2.2 (-2.8 to -1.7)	-1.8 (-2.8 to -1.0)	-3.1 (-24.8 to 16.7)	8.8 (-12.9 to 28.6)	-1.8 (-2.8 to -1.0)
Sierra Leone	12,000 (9,884-14,601)	148 (41-376)	1 (0-2)	12,149 (9,946 to 14,823)	1.3 (0.8 to 1.8)	9.8 (1.5 to 17.7)	28.0 (21.3 to 33.2)	1.4 (0.9 to 1.9)	-2.9 (-3.5 to -2.4)	-3.1 (-14.5 to 7.9)	8.1 (-3.4 to 19.0)	-2.9 (-3.5 to -2.4)
Singapore	1,522 (1,323-1,738)	9 (6-13)	1 (1-2)	1,532 (1,333 to 1,751)	-5.4 (-5.9 to -4.8)	-1.6 (-4.9 to 1.5)	26.0 (23.4 to 28.0)	-5.4 (-5.9 to -4.7)	0.2 (-0.4 to 0.8)	5.9 (1.6 to 10.4)	13.6 (9.3 to 18.0)	0.3 (-0.3 to 0.8)
Slovakia	441 (378-510)	3 (1-10)	1 (0-2)	445 (382 to 513)	-3.6 (-4.0 to -3.3)	1.0 (-8.3 to 14.1)	26.7 (22.6 to 30.3)	-3.6 (-3.9 to -3.3)	-3.9 (-4.5 to -3.3)	-7.1 (-25.3 to 6.2)	4.2 (-14.0 to 17.5)	-3.9 (-4.4 to -3.4)
Slovenia	167 (143-192)	0 (0-0)	0 (0-0)	167 (144 to 192)	-4.9 (-5.4 to -4.4)	-4.0 (-14.9 to 8.9)	19.5 (12.4 to 24.6)	-4.9 (-5.4 to -4.4)	-3.1 (-3.6 to -2.6)	-17.2 (-36.5 to -2.9)	-5.9 (-25.2 to 8.4)	-3.1 (-3.6 to -2.6)

	Number of incident cases (all ages, both sexes) with 95% uncertainty intervals (2016)				Annualized rate of change of age-standardized rate (%) with 95% uncertainty intervals							
					1990-2006				2006-2016			
	**Drug-susceptible TB**	**MDR TB**	**XDR TB**	**All HIV-negative TB**	**Drug-susceptible TB**	**MDR TB**	**XDR TB**	**All HIV-negative TB**	**Drug-susceptible TB**	**MDR TB**	**XDR TB**	**All HIV-negative TB**
Solomon Islands	461 (401-528)	5 (0-40)	0 (0-4)	467 (411 to 529)	-2.0 (-2.8 to -1.7)	10.6 (-15.0 to 38.7)	39.1 (6.7 to 51.9)	-1.9 (-2.2 to -1.7)	-2.1 (-2.9 to -1.0)	-5.8 (-39.8 to 30.3)	6.1 (-27.9 to 42.2)	-2.1 (-2.5 to -1.7)
Somalia	28,415 (23,565-34,013)	1,651 (728-3,057)	11 (5-20)	30,077 (25,187 to 36,127)	2.2 (1.6 to 2.7)	36.3 (29.5 to 43.9)	38.1 (33.0 to 42.6)	2.5 (2.0 to 3.0)	-0.1 (-1.0 to 0.8)	0.2 (-10.6 to 11.0)	11.3 (0.6 to 22.2)	-0.1 (-0.6 to 0.5)
South Africa	243,731 (188,739-310,091)	5,256 (2,866-9,247)	34 (18-60)	249,020 (192,775 to 316,296)	0.3 (-1.0 to 1.6)	12.0 (7.8 to 16.4)	33.6 (29.7 to 37.1)	0.4 (-0.9 to 1.7)	-0.4 (-1.1 to 0.3)	-1.4 (-6.6 to 3.8)	9.8 (4.5 to 14.9)	-0.4 (-1.2 to 0.3)
South Korea	42,122 (37,531-47,408)	632 (394-980)	79 (50-123)	42,833 (38,121 to 48,287)	-7.2 (-7.6 to -6.9)	2.7 (-0.5 to 5.7)	43.9 (41.5 to 45.9)	-7.1 (-7.5 to -6.8)	-3.9 (-4.4 to -3.5)	-6.2 (-10.5 to -1.8)	1.4 (-2.9 to 5.9)	-4.0 (-4.4 to -3.5)
South Sudan	20,785 (17,006-25,694)	421 (130-1,068)	3 (1-7)	21,209 (17,430 to 26,188)	1.8 (1.2 to 2.4)	22.1 (13.6 to 30.3)	27.0 (18.8 to 33.3)	2.0 (1.4 to 2.5)	0.8 (0.1 to 1.5)	1.7 (-9.5 to 12.7)	12.9 (1.6 to 23.9)	0.8 (0.1 to 1.5)
Spain	3,813 (2,864-5,034)	37 (21-60)	5 (3-8)	3,854 (2,897 to 5,091)	-3.6 (-4.6 to -2.6)	-1.4 (-4.6 to 1.9)	26.4 (23.3 to 29.1)	-3.6 (-4.6 to -2.6)	-4.4 (-5.4 to -3.4)	-4.2 (-8.7 to 0.4)	3.5 (-1.1 to 8.1)	-4.4 (-5.4 to -3.4)
Sri Lanka	8,599 (7,074-10,290)	24 (2-86)	2 (0-8)	8,625 (7,090 to 10,332)	-2.6 (-3.0 to -2.2)	10.3 (-1.8 to 24.5)	24.8 (13.7 to 32.3)	-2.6 (-3.0 to -2.2)	-3.8 (-4.4 to -3.1)	-5.5 (-25.7 to 12.3)	6.4 (-13.8 to 24.2)	-3.8 (-4.5 to -3.1)
Sudan	40,034 (33,506-47,765)	738 (65-3,268)	26 (2-115)	40,798 (34,293 to 48,536)	-2.5 (-3.0 to -2.0)	10.3 (-4.0 to 24.1)	35.4 (19.7 to 44.3)	-2.4 (-2.9 to -2.0)	-1.9 (-2.6 to -1.2)	1.8 (-18.4 to 21.1)	10.0 (-10.2 to 29.3)	-1.8 (-2.4 to -1.2)
Suriname	129 (105-157)	1 (0-3)	0 (0-0)	130 (106 to 158)	-0.4 (-1.4 to 0.4)	5.1 (-7.4 to 17.3)	25.6 (12.5 to 33.9)	-0.4 (-1.4 to 0.4)	-2.1 (-2.8 to -1.2)	-5.4 (-23.6 to 12.4)	4.4 (-13.8 to 22.2)	-2.1 (-2.8 to -1.2)
Swaziland	8,555 (6,025-11,800)	664 (240-1,460)	4 (2-9)	9,223 (6,551 to 12,522)	1.0 (-0.2 to 2.5)	20.7 (13.9 to 28.0)	41.5 (37.0 to 45.5)	1.4 (0.1 to 2.8)	-1.5 (-4.2 to 0.7)	1.0 (-11.9 to 11.9)	12.2 (-0.8 to 23.1)	-1.4 (-4.0 to 0.8)
Sweden	626 (514-753)	17 (11-24)	2 (1-3)	645 (530 to 774)	-0.0 (-0.4 to 0.4)	6.1 (3.4 to 8.7)	26.3 (23.8 to 28.7)	0.1 (-0.4 to 0.5)	0.8 (0.1 to 1.4)	4.4 (1.0 to 7.5)	12.0 (8.6 to 15.2)	0.9 (0.2 to 1.5)
Switzerland	487 (376-624)	12 (7-17)	1 (1-2)	500 (386 to 645)	-4.3 (-5.1 to -3.5)	1.4 (-1.7 to 4.4)	25.4 (23.0 to 27.7)	-4.2 (-5.1 to -3.4)	-0.6 (-1.6 to 0.4)	3.5 (-0.4 to 7.5)	11.2 (7.2 to 15.2)	-0.5 (-1.5 to 0.6)
Syria	3,377 (2,155-4,971)	254 (27-942)	9 (1-33)	3,641 (2,476 to 5,312)	-3.6 (-4.5 to -2.8)	19.6 (9.0 to 34.7)	34.4 (27.4 to 39.6)	-3.2 (-3.9 to -2.5)	-1.2 (-3.5 to 0.1)	-0.3 (-19.6 to 14.1)	7.9 (-11.4 to 22.3)	-1.2 (-2.1 to -0.2)
Taiwan	6,509 (5,897-7,156)	98 (55-158)	9 (5-14)	6,616 (5,983 to 7,272)	-4.2 (-4.5 to -3.9)	23.7 (20.0 to 27.3)	31.0 (27.4 to 34.1)	-4.0 (-4.4 to -3.8)	-2.7 (-3.1 to -2.2)	-4.2 (-9.4 to 1.0)	7.7 (2.5 to 12.9)	-2.7 (-3.1 to -2.3)

	Number of incident cases (all ages, both sexes) with 95% uncertainty intervals (2016)				Annualized rate of change of age-standardized rate (%) with 95% uncertainty intervals							
					1990-2006				2006-2016			
	**Drug-susceptible TB**	**MDR TB**	**XDR TB**	**All HIV-negative TB**	**Drug-susceptible TB**	**MDR TB**	**XDR TB**	**All HIV-negative TB**	**Drug-susceptible TB**	**MDR TB**	**XDR TB**	**All HIV-negative TB**
Tajikistan	5,619 (4,817-6,471)	651 (379-1,021)	143 (83-224)	6,414 (5,590 to 7,286)	1.4 (-0.1 to 2.2)	39.8 (30.4 to 51.8)	58.0 (49.7 to 63.3)	2.2 (1.9 to 2.6)	-5.1 (-6.7 to -2.3)	-6.4 (-17.9 to 9.0)	4.9 (-6.6 to 20.3)	-5.1 (-5.6 to -4.6)
Tanzania	86,939 (70,917-104,658)	1,147 (345-2,684)	7 (2-17)	88,093 (72,546 to 106,481)	1.5 (0.9 to 2.1)	24.6 (18.3 to 32.6)	23.7 (18.9 to 27.7)	1.6 (1.0 to 2.2)	0.1 (-0.7 to 1.2)	1.2 (-10.0 to 11.0)	12.4 (1.1 to 22.2)	0.1 (-0.7 to 1.2)
Thailand	51,423 (39,841-66,483)	1,370 (430-3,352)	125 (39-305)	52,917 (41,061 to 67,725)	-4.2 (-5.1 to -3.4)	1.9 (-6.6 to 13.1)	37.6 (33.3 to 40.9)	-4.2 (-5.1 to -3.3)	-3.4 (-4.3 to -2.5)	2.0 (-11.1 to 13.0)	13.9 (0.8 to 24.9)	-3.2 (-4.0 to -2.4)
The Bahamas	133 (107-162)	2 (0-5)	0 (0-0)	134 (109 to 164)	-2.5 (-3.0 to -2.0)	14.7 (3.5 to 27.7)	26.9 (15.7 to 34.8)	-2.4 (-2.9 to -2.0)	-0.8 (-1.4 to -0.1)	4.2 (-14.1 to 21.2)	13.9 (-4.4 to 31.0)	-0.7 (-1.3 to -0.1)
The Gambia	2,126 (1,776-2,495)	33 (9-83)	0 (0-1)	2,160 (1,810 to 2,538)	-0.9 (-1.6 to -0.4)	12.7 (4.6 to 20.2)	26.4 (18.2 to 32.2)	-0.8 (-1.5 to -0.4)	-2.0 (-2.5 to -1.5)	-3.2 (-14.0 to 7.4)	7.9 (-2.8 to 18.5)	-2.0 (-2.5 to -1.5)
Timor-Leste	983 (796-1,239)	5 (0-22)	0 (0-2)	989 (798 to 1,250)	-2.9 (-3.4 to -2.4)	10.6 (-3.1 to 25.8)	38.1 (23.9 to 46.5)	-2.8 (-3.3 to -2.3)	-3.0 (-3.8 to -2.2)	-10.6 (-31.1 to 7.5)	1.4 (-19.2 to 19.5)	-3.1 (-3.9 to -2.3)
Togo	10,388 (8,344-12,831)	164 (44-425)	1 (0-3)	10,553 (8,450 to 13,050)	-0.7 (-1.6 to 0.0)	12.6 (4.9 to 20.8)	27.3 (19.3 to 33.1)	-0.6 (-1.6 to 0.2)	-1.5 (-2.2 to -0.6)	-2.5 (-13.9 to 8.5)	8.7 (-2.8 to 19.6)	-1.5 (-2.2 to -0.7)
Tonga	23 (19-27)	0 (0-2)	0 (0-0)	23 (20 to 28)	-1.4 (-2.2 to -1.0)	9.6 (-15.4 to 37.9)	29.8 (-3.3 to 42.8)	-1.3 (-1.6 to -1.0)	-1.1 (-1.9 to -0.0)	-4.4 (-42.6 to 31.5)	7.5 (-30.7 to 43.4)	-1.1 (-1.6 to -0.7)
Trinidad and Tobago	176 (143-214)	1 (0-4)	0 (0-0)	177 (144 to 215)	-2.5 (-3.1 to -2.0)	3.1 (-10.6 to 17.5)	21.1 (6.2 to 29.5)	-2.5 (-3.1 to -1.9)	-1.1 (-1.8 to -0.5)	-4.3 (-22.8 to 14.2)	5.5 (-13.0 to 24.0)	-1.2 (-1.8 to -0.6)
Tunisia	3,376 (2,309-4,684)	25 (7-61)	1 (0-2)	3,402 (2,338 to 4,709)	-2.8 (-3.5 to -2.1)	18.2 (5.7 to 32.3)	24.3 (11.4 to 31.8)	-2.7 (-3.5 to -2.0)	-2.0 (-3.0 to -1.0)	-3.8 (-19.6 to 16.0)	4.4 (-11.4 to 24.2)	-2.0 (-3.0 to -1.0)
Turkey	19,330 (16,434-22,359)	558 (270-1,049)	20 (10-37)	19,908 (17,004 to 22,968)	-6.6 (-7.2 to -6.2)	21.9 (9.8 to 36.8)	31.8 (21.1 to 39.0)	-6.4 (-6.8 to -6.1)	-3.8 (-4.5 to -2.9)	-4.1 (-18.7 to 14.2)	4.1 (-10.5 to 22.4)	-3.8 (-4.3 to -3.3)
Turkmenistan	7,490 (6,070-8,940)	1,244 (625-2,076)	272 (137-455)	9,006 (7,758 to 10,356)	1.4 (-0.5 to 2.3)	40.9 (32.1 to 52.9)	62.2 (54.8 to 67.6)	2.4 (2.1 to 2.9)	-3.0 (-5.5 to 0.5)	-3.4 (-15.5 to 11.0)	7.9 (-4.2 to 22.2)	-2.8 (-3.4 to -2.3)
Uganda	103,904 (83,860-130,298)	1,967 (859-4,168)	13 (6-27)	105,884 (85,446 to 132,360)	2.1 (1.5 to 2.6)	29.9 (22.8 to 37.2)	29.5 (23.7 to 34.1)	2.2 (1.6 to 2.7)	-0.7 (-1.7 to 0.5)	-0.3 (-11.2 to 10.7)	10.8 (-0.0 to 21.8)	-0.7 (-1.7 to 0.5)
Ukraine	19,267 (15,848-22,894)	5,527 (3,779-7,896)	1,211 (828-1,730)	26,005 (22,285 to 30,099)	0.7 (-0.7 to 1.7)	39.9 (35.6 to 44.6)	57.7 (54.1 to 60.6)	2.3 (1.6 to 3.0)	-3.1 (-4.8 to -1.2)	-2.9 (-7.9 to 2.6)	8.4 (3.3 to 13.8)	-2.8 (-3.4 to -2.1)

	Number of incident cases (all ages, both sexes) with 95% uncertainty intervals (2016)				Annualized rate of change of age-standardized rate (%) with 95% uncertainty intervals							
					1990-2006				2006-2016			
	**Drug-susceptible TB**	**MDR TB**	**XDR TB**	**All HIV-negative TB**	**Drug-susceptible TB**	**MDR TB**	**XDR TB**	**All HIV-negative TB**	**Drug-susceptible TB**	**MDR TB**	**XDR TB**	**All HIV-negative TB**
United Arab Emirates	1,419 (1,190-1,667)	27 (2-125)	1 (0-4)	1,447 (1,238 to 1,710)	-3.0 (-3.5 to -2.6)	9.8 (-5.2 to 24.4)	22.0 (6.5 to 31.6)	-3.0 (-3.3 to -2.6)	-1.5 (-2.4 to -0.8)	1.7 (-17.1 to 20.8)	9.9 (-8.9 to 29.0)	-1.4 (-2.1 to -0.9)
United Kingdom	6,885 (5,605-8,396)	70 (56-86)	9 (7-11)	6,964 (5,668 to 8,491)	-0.7 (-1.0 to -0.5)	1.8 (1.4 to 2.3)	26.8 (25.5 to 28.1)	-0.7 (-1.0 to -0.5)	-2.2 (-2.5 to -1.9)	-1.2 (-1.8 to -0.6)	6.5 (5.9 to 7.1)	-2.2 (-2.5 to -1.9)
United States	10,620 (9,605-11,711)	124 (110-139)	16 (14-17)	10,760 (9,736 to 11,860)	-2.6 (-3.5 to -1.7)	-3.7 (-4.7 to -2.8)	20.5 (19.7 to 21.1)	-2.6 (-3.5 to -1.7)	-2.4 (-2.9 to -2.0)	-2.4 (-3.0 to -1.7)	5.3 (4.6 to 6.0)	-2.4 (-2.9 to -2.0)
Uruguay	592 (506-685)	1 (0-4)	0 (0-0)	594 (507 to 689)	-3.6 (-4.2 to -3.0)	-1.1 (-12.8 to 12.3)	12.0 (1.9 to 19.3)	-3.6 (-4.2 to -3.0)	1.3 (0.6 to 2.1)	12.2 (-2.5 to 29.4)	19.9 (5.2 to 37.0)	1.3 (0.6 to 2.1)
Uzbekistan	13,646 (9,441-17,598)	4,588 (1,880-8,033)	1,005 (412-1,760)	19,239 (16,916 to 21,679)	-1.9 (-4.5 to -0.5)	40.6 (32.3 to 52.6)	61.0 (55.7 to 65.0)	0.0 (-0.5 to 0.6)	-4.1 (-8.7 to 1.1)	-4.3 (-17.4 to 7.6)	6.9 (-6.1 to 18.9)	-3.8 (-4.3 to -3.4)
Vanuatu	204 (178-234)	0 (0-2)	0 (0-0)	204 (178 to 234)	-2.0 (-2.3 to -1.7)	3.8 (-25.7 to 27.1)	25.4 (-7.8 to 35.4)	-2.0 (-2.3 to -1.7)	-2.0 (-2.4 to -1.5)	-3.4 (-39.2 to 33.1)	8.5 (-27.3 to 45.0)	-2.0 (-2.4 to -1.5)
Venezuela	6,179 (5,256-7,191)	81 (39-150)	6 (3-12)	6,266 (5,326 to 7,324)	-2.7 (-3.2 to -2.2)	13.0 (8.0 to 17.7)	29.2 (24.9 to 32.9)	-2.6 (-3.2 to -2.1)	-1.4 (-1.9 to -0.9)	-2.7 (-9.6 to 3.5)	7.1 (0.2 to 13.3)	-1.4 (-1.9 to -0.9)
Vietnam	114,718 (96,196-136,141)	4,260 (1,288-9,731)	388 (117-887)	119,366 (100,647 to 141,193)	-2.6 (-3.1 to -2.2)	11.0 (2.6 to 22.4)	45.2 (41.0 to 48.5)	-2.5 (-2.9 to -2.1)	-3.2 (-4.0 to -2.5)	-1.0 (-14.9 to 9.9)	10.9 (-2.9 to 21.9)	-3.1 (-3.7 to -2.5)
Virgin Islands, U.S.	13 (11-16)	0 (0-0)	0 (0-0)	13 (11 to 16)	-1.6 (-2.1 to -1.1)	4.3 (-9.2 to 17.9)	21.3 (7.4 to 30.2)	-1.6 (-2.1 to -1.1)	-0.8 (-1.4 to -0.2)	-4.6 (-22.4 to 14.4)	5.2 (-12.6 to 24.2)	-0.8 (-1.5 to -0.2)
Yemen	14,107 (9,704-20,238)	302 (68-860)	11 (2-30)	14,420 (10,084 to 20,490)	-2.4 (-3.1 to -1.8)	17.0 (7.2 to 32.1)	35.7 (30.0 to 40.1)	-2.3 (-3.0 to -1.6)	-1.6 (-2.7 to -0.6)	-3.3 (-19.3 to 10.5)	4.9 (-11.0 to 18.7)	-1.7 (-2.7 to -0.7)
Zambia	67,052 (53,226-82,833)	936 (337-2,194)	6 (2-14)	67,994 (54,212 to 84,451)	4.7 (4.0 to 5.3)	20.1 (13.1 to 27.5)	30.1 (25.3 to 34.1)	4.7 (4.1 to 5.3)	-1.7 (-3.0 to -0.4)	-1.1 (-11.6 to 9.0)	10.0 (-0.4 to 20.1)	-1.7 (-3.1 to -0.4)
Zimbabwe	81,293 (59,617-110,081)	2,347 (512-6,572)	15 (3-42)	83,655 (62,081 to 113,164)	3.4 (2.4 to 4.5)	18.6 (8.9 to 28.1)	34.4 (24.6 to 40.9)	3.5 (2.5 to 4.6)	-3.1 (-5.5 to -1.1)	0.5 (-11.9 to 13.4)	11.7 (-0.7 to 24.5)	-3.0 (-5.5 to -1.0)

eTable 7. Tuberculosis, drug-susceptible tuberculosis, multidrug-resistant tuberculosis, and extensively drug-resistant tuberculosis deaths in HIV-negative individuals, and annualized rates of change of age-standardized rates for 195 countries and territories

	Number of deaths (all ages, both sexes) with 95% uncertainty intervals (2016)				Annualized rate of change of age-standardized rate (%) with 95% uncertainty intervals							
					1990-2006				2006-2016			
	**Drug-susceptible TB**	**MDR TB**	**XDR TB**	**All HIV-negative TB**	**Drug-susceptible TB**	**MDR TB**	**XDR TB**	**All HIV-negative TB**	**Drug-susceptible TB**	**MDR TB**	**XDR TB**	**All HIV-negative TB**
Global	1,105,898 (1,055,638 to 1,158,544)	96,238 (79,994 to 113,348)	10,920 (8,896 to 13,162)	1,213,057 (1,161,548 to 1,265,425)	-3.7 (-4.3 to -3.4)	11.9 (10.9 to 12.7)	43.9 (42.7 to 45.1)	-3.2 (-3.7 to -2.9)	-4.4 (-4.9 to -4.1)	-5.5 (-6.5 to -4.5)	3.1 (1.8 to 4.5)	-4.5 (-5.0 to -4.1)
Afghanistan	11,529 (4,345-16,450)	2,203 (392-5,933)	205 (37-559)	13,937 (5,302 to 18,534)	-2.3 (-4.1 to -1.0)	29.3 (19.9 to 43.8)	58.7 (48.6 to 64.3)	-1.1 (-2.1 to -0.2)	-5.2 (-9.5 to -1.3)	-5.1 (-22.5 to 10.3)	2.9 (-14.5 to 18.4)	-5.1 (-6.3 to -3.7)
Albania	12 (10-14)	0 (0-1)	0 (0-0)	12 (10 to 14)	-7.4 (-8.3 to -6.6)	8.8 (-2.7 to 22.6)	20.6 (10.2 to 27.5)	-7.3 (-8.1 to -6.5)	-6.0 (-7.7 to -4.1)	-8.7 (-27.0 to 8.9)	2.4 (-15.9 to 20.0)	-6.0 (-7.6 to -4.3)
Algeria	1,141 (506-1,725)	66 (6-294)	6 (1-26)	1,214 (550 to 1,815)	-7.0 (-8.2 to -5.2)	-4.1 (-15.4 to 6.5)	30.5 (21.7 to 36.8)	-6.9 (-8.0 to -5.2)	-4.7 (-6.7 to -2.9)	-2.9 (-21.1 to 10.3)	5.2 (-13.1 to 18.3)	-4.5 (-6.1 to -3.1)
American Samoa	1 (0-1)	0 (0-0)	0 (0-0)	1 (0 to 1)	-6.6 (-8.3 to -5.3)	2.8 (-24.5 to 31.1)	22.1 (-9.7 to 34.1)	-6.4 (-7.6 to -5.2)	-0.8 (-3.8 to 1.7)	-3.4 (-39.0 to 27.5)	8.3 (-27.3 to 39.2)	-0.8 (-3.0 to 1.1)
Andorra	0 (0-1)	0 (0-0)	0 (0-0)	0 (0 to 1)	-5.4 (-7.0 to -3.5)	-6.0 (-9.6 to -2.1)	-12.3 (-16.7 to -7.7)	-5.4 (-7.0 to -3.5)	-1.3 (-3.3 to 0.7)	-2.6 (-7.5 to 2.0)	4.8 (-0.0 to 9.5)	-1.3 (-3.3 to 0.7)
Angola	10,940 (8,217-14,880)	246 (124-455)	4 (2-8)	11,191 (8,426 to 15,150)	-3.7 (-5.6 to -1.8)	5.6 (1.1 to 10.0)	34.1 (29.1 to 37.6)	-3.6 (-5.5 to -1.7)	-5.0 (-7.0 to -3.0)	-5.4 (-10.8 to 0.4)	5.8 (0.4 to 11.6)	-5.0 (-7.0 to -3.0)
Antigua and Barbuda	0 (0-1)	0 (0-0)	0 (0-0)	0 (0 to 1)	-2.5 (-3.5 to -1.5)	3.3 (-11.0 to 16.2)	17.1 (3.0 to 25.3)	-2.4 (-3.2 to -1.5)	-4.2 (-5.9 to -2.5)	-8.6 (-25.3 to 8.0)	1.0 (-15.6 to 17.7)	-4.3 (-5.9 to -2.7)
Argentina	608 (492-693)	40 (6-131)	13 (2-42)	661 (597 to 728)	-6.4 (-7.2 to -5.8)	6.8 (-2.5 to 20.4)	33.9 (28.4 to 38.1)	-5.9 (-6.5 to -5.4)	-3.4 (-5.4 to -1.9)	-5.0 (-21.3 to 6.2)	2.5 (-13.9 to 13.7)	-3.4 (-4.4 to -2.3)
Armenia	60 (33-83)	14 (4-31)	8 (2-17)	83 (70 to 100)	0.8 (-0.4 to 2.0)	32.5 (24.1 to 45.5)	47.9 (45.3 to 50.0)	2.6 (1.6 to 3.5)	-8.6 (-14.8 to -4.9)	-10.1 (-23.4 to -0.5)	1.0 (-12.3 to 10.6)	-8.3 (-10.3 to -6.0)
Australia	68 (59-77)	4 (2-7)	1 (1-2)	73 (65 to 82)	-4.8 (-5.7 to -4.0)	6.5 (-3.3 to 16.8)	19.5 (11.0 to 25.6)	-4.4 (-5.0 to -3.8)	-3.4 (-4.7 to -1.9)	-3.5 (-14.9 to 10.0)	3.9 (-7.4 to 17.4)	-3.3 (-4.3 to -2.1)
Austria	46 (40-53)	4 (2-5)	1 (1-2)	51 (45 to 58)	-9.3 (-9.9 to -8.7)	0.5 (-2.2 to 3.5)	22.9 (21.0 to 24.7)	-8.9 (-9.5 to -8.3)	-2.8 (-4.0 to -1.3)	-1.0 (-4.6 to 2.6)	6.5 (2.9 to 10.0)	-2.5 (-3.8 to -1.1)
Azerbaijan	386 (238-579)	153 (85-259)	89 (49-146)	629 (479 to 920)	-2.5 (-6.4 to -0.2)	35.9 (27.3 to 48.4)	55.8 (49.3 to 59.4)	0.3 (-0.8 to 1.4)	-7.9 (-14.2 to -0.1)	-9.8 (-19.2 to 2.4)	1.3 (-8.1 to 13.5)	-7.5 (-10.5 to -4.2)
Bahrain	10 (7-12)	1 (0-2)	0 (0-0)	10 (8 to 13)	-5.4 (-6.8 to -4.0)	17.3 (6.3 to 31.9)	26.5 (17.3 to 32.7)	-5.1 (-6.5 to -3.7)	-6.4 (-9.1 to -3.7)	-4.0 (-20.7 to 14.1)	4.0 (-12.6 to 22.1)	-6.3 (-8.6 to -3.8)
Bangladesh	17,551 (15,135-20,270)	1,175 (849-1,599)	76 (54-105)	18,803 (16,191 to 21,683)	-7.1 (-8.1 to -6.0)	20.9 (18.9 to 22.8)	36.6 (34.4 to 38.5)	-6.7 (-7.7 to -5.6)	-1.0 (-2.6 to 0.5)	-1.3 (-4.0 to 1.4)	8.2 (5.6 to 11.0)	-1.0 (-2.6 to 0.5)

	Number of deaths (all ages, both sexes) with 95% uncertainty intervals (2016)				Annualized rate of change of age-standardized rate (%) with 95% uncertainty intervals							
					1990-2006				2006-2016			
	**Drug-susceptible TB**	**MDR TB**	**XDR TB**	**All HIV-negative TB**	**Drug-susceptible TB**	**MDR TB**	**XDR TB**	**All HIV-negative TB**	**Drug-susceptible TB**	**MDR TB**	**XDR TB**	**All HIV-negative TB**
Barbados	2 (2-3)	0 (0-0)	0 (0-0)	2 (2 to 3)	-1.9 (-2.7 to -1.1)	-0.5 (-14.2 to 13.0)	-0.8 (-15.2 to 8.1)	-1.9 (-2.7 to -1.1)	-2.0 (-3.2 to -0.7)	-4.4 (-20.4 to 13.0)	5.3 (-10.8 to 22.6)	-2.0 (-3.2 to -0.7)
Belarus	151 (110-202)	184 (141-237)	106 (81-141)	442 (348 to 564)	-1.2 (-3.4 to 0.5)	45.1 (41.0 to 49.8)	54.8 (53.2 to 56.1)	4.1 (3.2 to 5.0)	-8.9 (-12.6 to -4.8)	-8.3 (-11.5 to -4.7)	2.8 (-0.4 to 6.4)	-6.8 (-9.5 to -4.1)
Belgium	87 (76-100)	4 (3-5)	1 (1-2)	92 (81 to 105)	-4.1 (-4.8 to -3.4)	1.6 (-1.2 to 4.4)	24.3 (22.2 to 26.0)	-3.9 (-4.5 to -3.2)	-3.8 (-5.2 to -2.4)	-5.5 (-9.1 to -2.2)	2.0 (-1.7 to 5.3)	-3.8 (-5.2 to -2.5)
Belize	16 (13-19)	0 (0-1)	0 (0-0)	16 (14 to 19)	0.3 (-0.7 to 1.2)	6.6 (-7.8 to 20.0)	33.2 (19.3 to 41.7)	0.4 (-0.5 to 1.3)	-3.8 (-5.8 to -1.9)	-8.8 (-26.5 to 7.5)	0.8 (-16.9 to 17.2)	-3.9 (-5.8 to -2.0)
Benin	2,512 (1,930-3,168)	72 (25-177)	1 (0-3)	2,586 (1,994 to 3,256)	-1.3 (-2.4 to -0.3)	10.3 (3.3 to 17.8)	28.6 (22.2 to 33.2)	-1.2 (-2.3 to -0.1)	-2.4 (-3.7 to -1.1)	-1.4 (-11.6 to 8.6)	9.8 (-0.5 to 19.8)	-2.4 (-3.7 to -1.1)
Bermuda	0 (0-0)	0 (0-0)	0 (0-0)	0 (0 to 0)	-4.6 (-5.5 to -3.7)	-3.6 (-16.2 to 10.1)	-8.1 (-20.6 to 0.2)	-4.6 (-5.5 to -3.7)	-7.0 (-8.7 to -5.3)	-9.2 (-25.8 to 6.9)	0.4 (-16.2 to 16.5)	-7.0 (-8.7 to -5.3)
Bhutan	49 (29-79)	4 (2-7)	0 (0-0)	53 (32 to 86)	-6.8 (-8.5 to -5.1)	10.3 (7.8 to 12.9)	36.6 (32.8 to 39.9)	-6.3 (-7.9 to -4.7)	-5.4 (-7.4 to -3.6)	-6.0 (-8.7 to -3.2)	3.6 (0.8 to 6.3)	-5.5 (-7.4 to -3.7)
Bolivia	1,029 (737-1,367)	80 (20-217)	17 (4-46)	1,125 (837 to 1,479)	-8.8 (-10.2 to -7.5)	3.5 (-5.2 to 13.2)	46.2 (37.3 to 52.3)	-8.2 (-9.3 to -7.0)	-5.1 (-7.3 to -2.9)	-7.5 (-18.8 to 3.0)	2.1 (-9.2 to 12.7)	-5.2 (-7.1 to -3.4)
Bosnia and Herzegovina	144 (120-169)	1 (0-2)	0 (0-1)	145 (121 to 170)	-6.6 (-7.8 to -5.4)	-1.9 (-10.9 to 11.9)	26.4 (22.1 to 30.0)	-6.6 (-7.8 to -5.4)	-5.1 (-7.0 to -3.3)	-9.1 (-22.8 to 1.8)	2.1 (-11.7 to 12.9)	-5.1 (-7.1 to -3.3)
Botswana	800 (374-1,234)	42 (11-99)	1 (0-2)	844 (390 to 1,291)	2.9 (-0.7 to 5.5)	15.4 (7.8 to 24.5)	34.5 (29.8 to 38.4)	3.1 (-0.4 to 5.8)	-6.0 (-12.0 to -0.4)	-4.2 (-17.2 to 7.1)	6.9 (-6.0 to 18.3)	-5.9 (-11.9 to -0.2)
Brazil	5,071 (4,822-5,427)	215 (181-254)	45 (37-54)	5,331 (5,068 to 5,697)	-4.4 (-4.8 to -4.1)	20.2 (19.6 to 20.8)	31.7 (30.4 to 32.9)	-4.1 (-4.4 to -3.8)	-4.4 (-4.9 to -3.9)	-6.2 (-7.0 to -5.4)	3.4 (2.6 to 4.3)	-4.4 (-4.9 to -3.9)
Brunei	16 (13-19)	0 (0-0)	0 (0-0)	16 (13 to 19)	-4.8 (-5.5 to -4.0)	-0.2 (-3.6 to 3.5)	14.8 (11.2 to 17.9)	-4.8 (-5.5 to -3.9)	-2.0 (-3.6 to -0.4)	3.5 (-1.4 to 8.3)	10.9 (6.1 to 15.8)	-2.0 (-3.6 to -0.4)
Bulgaria	109 (86-135)	6 (2-15)	4 (1-9)	119 (96 to 144)	-1.6 (-2.5 to -0.9)	21.2 (11.4 to 35.9)	33.4 (25.2 to 38.8)	-1.1 (-1.7 to -0.5)	-5.6 (-8.4 to -2.9)	-7.8 (-24.6 to 9.3)	3.4 (-13.4 to 20.5)	-5.5 (-7.6 to -3.4)
Burkina Faso	5,865 (5,132-6,608)	279 (82-686)	5 (1-12)	6,148 (5,444 to 6,847)	-2.4 (-3.5 to -1.3)	11.6 (4.0 to 19.2)	35.7 (27.7 to 41.2)	-2.1 (-3.1 to -1.0)	-1.4 (-3.2 to 0.2)	-3.5 (-12.2 to 6.6)	7.7 (-1.1 to 17.8)	-1.5 (-3.2 to 0.0)
Burundi	10,476 (7,950-13,266)	647 (173-1,583)	11 (3-27)	11,135 (8,522 to 13,780)	-0.1 (-2.1 to 3.1)	20.2 (11.6 to 28.9)	41.0 (32.5 to 46.4)	0.2 (-1.8 to 3.5)	-1.8 (-3.4 to -0.2)	-0.3 (-11.0 to 9.5)	10.8 (0.2 to 20.7)	-1.7 (-3.1 to -0.2)
Cambodia	2,386 (1,944-2,970)	93 (16-295)	22 (4-72)	2,501 (2,102 to 3,113)	-4.5 (-5.6 to -3.1)	17.0 (6.8 to 32.3)	48.5 (43.0 to 52.7)	-4.2 (-5.2 to -2.8)	-8.6 (-10.6 to -6.5)	-10.5 (-27.8 to 3.0)	1.2 (-16.1 to 14.7)	-8.6 (-10.3 to -6.7)

	Number of deaths (all ages, both sexes) with 95% uncertainty intervals (2016)				Annualized rate of change of age-standardized rate (%) with 95% uncertainty intervals							
					1990-2006				2006-2016			
	Drug-susceptible TB	MDR TB	XDR TB	All HIV-negative TB	Drug-susceptible TB	MDR TB	XDR TB	All HIV-negative TB	Drug-susceptible TB	MDR TB	XDR TB	All HIV-negative TB
Cameroon	5,344 (3,209-7,375)	246 (64-656)	4 (1-11)	5,594 (3,381 to 7,753)	0.5 (-0.9 to 1.7)	14.0 (6.5 to 22.2)	33.2 (25.2 to 38.8)	0.8 (-0.5 to 2.0)	-2.8 (-4.5 to -1.2)	-4.6 (-14.7 to 5.7)	6.6 (-3.6 to 16.8)	-2.9 (-4.5 to -1.3)
Canada	120 (107-136)	3 (2-4)	1 (1-1)	124 (110 to 141)	-5.1 (-5.7 to -4.6)	-4.2 (-5.5 to -2.7)	15.8 (14.1 to 17.3)	-5.0 (-5.6 to -4.5)	-3.5 (-4.6 to -2.4)	-4.5 (-6.5 to -2.6)	2.9 (0.9 to 4.9)	-3.5 (-4.6 to -2.3)
Cape Verde	35 (27-47)	2 (0-4)	0 (0-0)	37 (28 to 49)	-3.9 (-5.7 to -2.3)	9.9 (1.6 to 18.2)	26.0 (18.0 to 31.7)	-3.6 (-5.3 to -2.1)	-6.4 (-8.8 to -4.3)	-8.1 (-18.2 to 2.4)	3.1 (-7.1 to 13.5)	-6.5 (-8.7 to -4.4)
Central African Republic	12,771 (9,728-16,352)	215 (114-385)	4 (2-7)	12,989 (9,874 to 16,629)	-0.1 (-1.6 to 1.1)	7.5 (3.2 to 12.4)	38.9 (34.9 to 42.3)	-0.1 (-1.5 to 1.2)	-0.3 (-2.1 to 1.4)	-1.1 (-6.8 to 4.3)	10.0 (4.3 to 15.4)	-0.3 (-2.1 to 1.4)
Chad	4,135 (3,167-5,264)	202 (57-499)	3 (1-9)	4,340 (3,352 to 5,519)	-0.2 (-2.1 to 1.3)	13.9 (5.2 to 21.8)	36.6 (28.7 to 42.5)	0.1 (-1.7 to 1.6)	-3.6 (-5.2 to -2.1)	-5.6 (-15.8 to 4.9)	5.6 (-4.7 to 16.1)	-3.7 (-5.1 to -2.2)
Chile	456 (355-580)	10 (4-20)	3 (1-6)	469 (366 to 596)	-6.0 (-6.8 to -5.2)	2.1 (-9.2 to 14.5)	30.4 (22.3 to 36.0)	-5.9 (-6.6 to -5.1)	-4.9 (-7.4 to -2.2)	-5.7 (-17.4 to 7.8)	1.7 (-10.0 to 15.2)	-4.9 (-7.4 to -2.3)
China	35,009 (32,796-38,700)	4,119 (3,324-5,073)	992 (792-1,246)	40,120 (37,987 to 44,436)	-8.7 (-9.2 to -8.3)	-0.3 (-1.3 to 0.7)	40.5 (39.0 to 41.9)	-8.0 (-8.4 to -7.6)	-8.7 (-9.3 to -7.7)	-11.5 (-12.8 to -10.1)	0.3 (-1.1 to 1.6)	-8.8 (-9.5 to -7.9)
Colombia	773 (673-901)	53 (27-91)	11 (6-19)	837 (736 to 971)	-4.9 (-5.5 to -4.4)	13.1 (9.0 to 17.9)	34.3 (31.7 to 36.7)	-4.4 (-4.9 to -3.9)	-6.2 (-7.5 to -4.7)	-8.4 (-14.2 to -3.2)	1.2 (-4.6 to 6.4)	-6.2 (-7.6 to -4.9)
Comoros	249 (184-315)	15 (4-37)	0 (0-1)	265 (198 to 332)	0.6 (-1.3 to 3.1)	21.2 (12.7 to 29.9)	33.7 (25.4 to 39.3)	0.9 (-0.9 to 3.4)	-1.1 (-2.7 to 0.4)	-0.1 (-10.2 to 9.6)	11.1 (1.0 to 20.8)	-1.0 (-2.4 to 0.3)
Congo (Brazzaville)	2,055 (1,520-2,640)	46 (23-84)	1 (0-1)	2,101 (1,559 to 2,701)	-4.3 (-5.7 to -2.9)	5.0 (0.9 to 9.3)	32.3 (28.1 to 35.8)	-4.2 (-5.6 to -2.8)	-4.2 (-6.1 to -2.4)	-4.6 (-10.2 to 0.7)	6.6 (0.9 to 11.9)	-4.2 (-6.2 to -2.4)
Costa Rica	46 (41-52)	2 (1-3)	0 (0-1)	48 (43 to 54)	-7.5 (-8.1 to -6.8)	14.6 (9.8 to 19.4)	25.7 (21.8 to 29.0)	-7.2 (-7.8 to -6.5)	-5.4 (-6.6 to -4.2)	-6.6 (-12.4 to -0.9)	3.0 (-2.8 to 8.7)	-5.4 (-6.6 to -4.3)
Cote d'Ivoire	6,289 (4,202-8,344)	475 (164-1,064)	8 (3-18)	6,772 (4,447 to 8,976)	-0.5 (-1.8 to 0.6)	7.3 (1.3 to 13.8)	37.1 (31.9 to 41.0)	-0.2 (-1.4 to 0.9)	-3.4 (-5.0 to -1.7)	-3.9 (-13.2 to 4.3)	7.3 (-2.1 to 15.5)	-3.4 (-4.8 to -1.9)
Croatia	65 (55-77)	0 (0-1)	0 (0-1)	65 (55 to 77)	-7.9 (-8.7 to -7.0)	-5.7 (-15.7 to 8.4)	22.7 (17.4 to 27.0)	-7.9 (-8.7 to -7.0)	-7.8 (-9.6 to -6.0)	-14.5 (-32.1 to -1.8)	-3.3 (-21.0 to 9.3)	-7.9 (-9.6 to -6.1)
Cuba	39 (35-44)	1 (0-3)	0 (0-1)	41 (37 to 45)	-5.5 (-6.2 to -4.8)	-3.9 (-12.1 to 5.3)	12.6 (7.6 to 16.9)	-5.5 (-6.1 to -4.8)	-3.3 (-4.6 to -2.0)	-2.9 (-16.3 to 9.1)	6.7 (-6.7 to 18.7)	-3.3 (-4.4 to -2.0)
Cyprus	4 (4-5)	0 (0-0)	0 (0-0)	4 (4 to 5)	-6.1 (-6.9 to -5.3)	5.1 (1.8 to 8.4)	15.5 (12.3 to 18.2)	-6.0 (-6.8 to -5.2)	-5.2 (-6.4 to -3.6)	-10.4 (-14.5 to -6.1)	-3.0 (-7.1 to 1.3)	-5.2 (-6.5 to -3.7)
Czech Republic	48 (42-56)	2 (1-4)	1 (1-2)	51 (45 to 59)	-7.4 (-8.1 to -6.7)	-2.0 (-10.1 to 9.7)	24.4 (21.7 to 26.8)	-7.2 (-7.8 to -6.5)	-5.4 (-6.8 to -3.8)	-8.0 (-16.5 to -0.2)	3.1 (-5.3 to 10.9)	-5.4 (-6.6 to -3.9)

	Number of deaths (all ages, both sexes) with 95% uncertainty intervals (2016)				Annualized rate of change of age-standardized rate (%) with 95% uncertainty intervals							
					1990-2006				2006-2016			
	**Drug-susceptible TB**	**MDR TB**	**XDR TB**	**All HIV-negative TB**	**Drug-susceptible TB**	**MDR TB**	**XDR TB**	**All HIV-negative TB**	**Drug-susceptible TB**	**MDR TB**	**XDR TB**	**All HIV-negative TB**
Democratic Republic of the Congo	68,569 (50,595-89,117)	1,551 (762-2,692)	26 (13-46)	70,146 (51,990 to 90,730)	0.7 (-0.6 to 1.9)	10.2 (5.5 to 14.3)	36.7 (32.1 to 40.6)	0.8 (-0.5 to 2.0)	-2.9 (-4.0 to -1.7)	-3.3 (-8.4 to 2.4)	7.8 (2.7 to 13.5)	-2.9 (-4.0 to -1.7)
Denmark	26 (22-31)	0 (0-1)	0 (0-0)	27 (23 to 32)	-3.4 (-4.0 to -2.6)	4.1 (1.1 to 7.2)	13.0 (10.0 to 15.5)	-3.3 (-4.0 to -2.5)	-5.6 (-7.5 to -3.8)	-1.5 (-6.1 to 2.8)	5.9 (1.3 to 10.2)	-5.5 (-7.4 to -3.7)
Djibouti	396 (280-513)	51 (25-91)	1 (0-2)	448 (313 to 573)	4.2 (2.3 to 5.8)	37.2 (29.5 to 45.3)	40.4 (33.7 to 45.1)	4.9 (3.2 to 6.6)	-2.5 (-4.4 to -0.2)	-2.2 (-10.5 to 7.8)	8.9 (0.7 to 18.9)	-2.4 (-4.0 to -0.7)
Dominica	3 (3-4)	0 (0-0)	0 (0-0)	3 (3 to 4)	-2.0 (-2.9 to -1.2)	4.0 (-10.8 to 17.8)	28.2 (14.4 to 36.6)	-1.9 (-2.7 to -1.1)	-2.0 (-4.2 to 0.0)	-6.6 (-23.9 to 10.8)	3.1 (-14.3 to 20.5)	-2.0 (-4.1 to -0.1)
Dominican Republic	372 (308-438)	5 (1-19)	1 (0-4)	379 (316 to 442)	-7.5 (-8.5 to -6.5)	-1.5 (-14.8 to 11.1)	29.5 (15.4 to 37.4)	-7.4 (-8.4 to -6.4)	-4.1 (-5.9 to -2.3)	-8.8 (-25.2 to 8.5)	0.8 (-15.6 to 18.1)	-4.2 (-5.9 to -2.4)
Ecuador	514 (386-614)	74 (23-166)	16 (5-35)	604 (541 to 685)	-8.6 (-9.6 to -7.7)	11.0 (3.1 to 20.4)	43.3 (39.4 to 46.4)	-7.6 (-8.1 to -7.0)	-6.8 (-9.2 to -5.1)	-8.4 (-18.5 to -1.1)	1.3 (-8.8 to 8.5)	-6.9 (-8.0 to -5.6)
Egypt	810 (585-1,043)	112 (29-268)	10 (3-25)	932 (697 to 1,173)	-6.5 (-8.1 to -4.9)	13.0 (3.1 to 26.8)	27.0 (21.1 to 31.2)	-5.9 (-7.5 to -4.1)	-4.3 (-7.4 to -1.8)	-1.2 (-16.4 to 11.4)	6.9 (-8.4 to 19.5)	-3.9 (-5.8 to -2.1)
El Salvador	76 (66-88)	3 (1-5)	1 (0-1)	79 (69 to 91)	-11.6 (-12.4 to -10.2)	3.6 (-1.7 to 8.7)	26.8 (21.9 to 30.6)	-11.3 (-12.1 to -10.0)	-4.2 (-5.6 to -2.9)	-5.9 (-12.8 to 1.0)	3.7 (-3.1 to 10.6)	-4.3 (-5.6 to -2.9)
Equatorial Guinea	188 (105-295)	4 (2-8)	0 (0-0)	192 (108 to 304)	-11.5 (-14.2 to -9.0)	-2.3 (-6.7 to 2.6)	28.7 (23.7 to 32.7)	-11.4 (-14.1 to -8.9)	-5.8 (-9.1 to -2.6)	-6.1 (-12.0 to -0.2)	5.1 (-0.9 to 10.9)	-5.8 (-9.1 to -2.6)
Eritrea	3,249 (2,052-4,125)	209 (58-514)	4 (1-9)	3,461 (2,199 to 4,320)	0.9 (-0.4 to 2.2)	21.3 (12.8 to 29.8)	37.9 (30.2 to 43.8)	1.3 (-0.0 to 2.5)	0.6 (-1.6 to 2.2)	1.5 (-9.0 to 11.7)	12.7 (2.2 to 22.8)	0.6 (-1.4 to 2.1)
Estonia	17 (13-22)	8 (6-10)	5 (3-6)	29 (23 to 37)	-1.9 (-3.0 to -0.8)	3.2 (0.8 to 6.1)	48.7 (47.5 to 49.8)	-0.2 (-1.0 to 0.5)	-10.0 (-12.8 to -7.3)	-11.3 (-14.4 to -8.2)	-0.2 (-3.3 to 3.0)	-9.4 (-11.8 to -7.1)
Ethiopia	45,944 (37,330-55,237)	2,916 (865-6,806)	50 (14-116)	48,910 (40,310 to 58,195)	-5.0 (-6.3 to -2.8)	18.0 (10.9 to 26.2)	39.5 (35.5 to 43.3)	-4.7 (-6.0 to -2.4)	-6.3 (-8.2 to -4.4)	-5.1 (-15.2 to 3.3)	6.0 (-4.1 to 14.4)	-6.2 (-7.9 to -4.4)
Federated States of Micronesia	6 (5-8)	0 (0-1)	0 (0-0)	6 (5 to 8)	-6.3 (-8.2 to -4.7)	3.0 (-22.4 to 31.4)	36.6 (3.3 to 48.8)	-6.2 (-7.7 to -4.7)	-3.1 (-5.7 to -0.1)	-6.6 (-41.6 to 26.7)	5.1 (-29.9 to 38.5)	-3.2 (-5.4 to -1.1)
Fiji	38 (29-48)	0 (0-1)	0 (0-0)	38 (29 to 48)	-1.9 (-4.1 to -0.1)	1.3 (-25.1 to 29.5)	20.1 (-14.0 to 31.8)	-1.9 (-4.1 to -0.2)	-2.4 (-5.4 to 0.1)	-5.4 (-39.5 to 31.9)	6.3 (-27.8 to 43.6)	-2.4 (-5.3 to 0.1)
Finland	51 (44-60)	2 (1-3)	1 (0-1)	54 (46 to 63)	-6.7 (-7.4 to -6.0)	6.1 (3.0 to 9.1)	20.6 (17.9 to 22.9)	-6.6 (-7.2 to -5.9)	-6.3 (-7.8 to -4.7)	1.0 (-2.9 to 4.9)	8.4 (4.5 to 12.4)	-6.0 (-7.4 to -4.4)
France	998 (854-1,162)	38 (26-54)	13 (9-18)	1,048 (899 to 1,219)	-5.7 (-6.2 to -5.1)	0.2 (-2.3 to 2.7)	26.9 (25.2 to 28.4)	-5.5 (-6.0 to -4.9)	-5.4 (-6.7 to -4.1)	-6.1 (-9.5 to -2.9)	1.4 (-2.1 to 4.6)	-5.4 (-6.7 to -4.0)
Gabon	480 (362-627)	10 (5-18)	0 (0-0)	490 (370 to 643)	-3.3 (-4.8 to -2.0)	5.8 (1.4 to 10.0)	28.9 (24.6 to 32.4)	-3.2 (-4.7 to -1.9)	-5.7 (-7.8 to -3.2)	-6.1 (-11.7 to -0.5)	5.0 (-0.5 to 10.7)	-5.7 (-7.8 to -3.2)

	Number of deaths (all ages, both sexes) with 95% uncertainty intervals (2016)				Annualized rate of change of age-standardized rate (%) with 95% uncertainty intervals							
					1990-2006				2006-2016			
	**Drug-susceptible TB**	**MDR TB**	**XDR TB**	**All HIV-negative TB**	**Drug-susceptible TB**	**MDR TB**	**XDR TB**	**All HIV-negative TB**	**Drug-susceptible TB**	**MDR TB**	**XDR TB**	**All HIV-negative TB**
Georgia	113 (83-147)	43 (30-60)	25 (17-35)	181 (141 to 231)	-4.0 (-5.1 to -2.8)	23.6 (15.8 to 36.0)	45.0 (42.8 to 47.2)	-2.7 (-3.8 to -1.7)	-4.4 (-7.7 to -1.6)	2.6 (-1.7 to 7.1)	13.8 (9.4 to 18.2)	-1.7 (-4.6 to 0.9)
Germany	416 (366-472)	25 (17-33)	8 (6-11)	449 (398 to 509)	-9.4 (-10.1 to -8.7)	-4.1 (-6.6 to -1.4)	21.3 (19.7 to 22.8)	-9.2 (-9.9 to -8.5)	-4.5 (-5.8 to -3.2)	-3.0 (-6.0 to -0.2)	4.4 (1.5 to 7.2)	-4.3 (-5.6 to -3.0)
Ghana	5,916 (4,952-7,094)	272 (83-700)	5 (1-12)	6,193 (5,195 to 7,326)	-4.6 (-6.1 to -3.1)	8.9 (0.9 to 17.4)	34.1 (26.7 to 39.3)	-4.3 (-5.6 to -2.8)	-4.7 (-6.3 to -3.2)	-6.6 (-16.8 to 4.2)	4.6 (-5.6 to 15.3)	-4.8 (-6.3 to -3.4)
Greece	144 (126-162)	12 (8-19)	4 (3-6)	160 (142 to 181)	-6.5 (-7.1 to -5.9)	16.7 (14.0 to 19.7)	28.5 (26.0 to 30.5)	-5.8 (-6.3 to -5.2)	-0.1 (-1.4 to 1.1)	-2.0 (-6.1 to 1.7)	5.4 (1.4 to 9.2)	-0.2 (-1.4 to 1.0)
Greenland	2 (2-3)	0 (0-0)	0 (0-0)	2 (2 to 3)	-6.5 (-7.6 to -5.1)	-4.8 (-6.5 to -2.9)	38.7 (36.6 to 40.4)	-6.4 (-7.5 to -5.0)	-4.8 (-6.9 to -2.6)	-5.6 (-8.3 to -2.8)	1.9 (-0.8 to 4.6)	-4.8 (-6.8 to -2.5)
Grenada	1 (1-1)	0 (0-0)	0 (0-0)	1 (1 to 1)	-0.7 (-1.7 to 0.3)	5.4 (-8.4 to 19.4)	20.0 (5.7 to 29.0)	-0.6 (-1.5 to 0.4)	-3.0 (-4.8 to -1.2)	-7.9 (-25.3 to 7.8)	1.7 (-15.7 to 17.5)	-3.1 (-4.9 to -1.4)
Guam	5 (4-6)	0 (0-1)	0 (0-0)	5 (4 to 6)	-6.2 (-7.7 to -5.0)	8.0 (-7.0 to 37.2)	31.0 (21.5 to 37.3)	-5.8 (-6.9 to -4.7)	2.4 (-0.5 to 5.3)	-6.7 (-40.3 to 15.2)	5.1 (-28.6 to 26.9)	2.0 (-0.2 to 4.0)
Guatemala	323 (247-410)	23 (11-42)	5 (2-9)	351 (270 to 445)	-11.1 (-12.4 to -9.9)	8.9 (4.5 to 13.8)	36.4 (32.8 to 39.5)	-10.6 (-11.8 to -9.4)	-5.3 (-8.2 to -2.6)	-7.0 (-13.1 to -1.2)	2.6 (-3.4 to 8.4)	-5.3 (-8.2 to -2.7)
Guinea	5,021 (3,743-6,167)	235 (65-595)	4 (1-10)	5,260 (3,943 to 6,437)	-0.2 (-1.4 to 1.0)	13.4 (5.1 to 21.4)	36.1 (29.0 to 41.5)	0.1 (-1.0 to 1.2)	-1.3 (-2.9 to 0.4)	-3.2 (-13.9 to 6.8)	8.0 (-2.8 to 18.0)	-1.4 (-2.8 to 0.2)
Guinea-Bissau	1,132 (694-1,447)	52 (14-126)	1 (0-2)	1,185 (726 to 1,500)	-0.3 (-1.8 to 1.0)	13.2 (5.3 to 22.0)	38.9 (30.9 to 44.3)	0.0 (-1.4 to 1.2)	-2.3 (-3.8 to -0.6)	-4.3 (-14.0 to 5.7)	6.8 (-2.9 to 16.9)	-2.4 (-3.8 to -0.8)
Guyana	69 (58-81)	1 (0-4)	0 (0-1)	70 (59 to 82)	0.8 (-0.1 to 1.6)	6.3 (-6.6 to 20.1)	35.8 (22.5 to 44.1)	0.9 (0.1 to 1.7)	-4.2 (-5.7 to -2.5)	-9.0 (-25.7 to 8.0)	0.6 (-16.0 to 17.6)	-4.3 (-5.8 to -2.7)
Haiti	1,300 (901-1,720)	18 (2-76)	4 (0-16)	1,322 (920 to 1,741)	-5.4 (-6.7 to -3.7)	0.2 (-12.8 to 13.2)	38.2 (25.3 to 46.3)	-5.3 (-6.6 to -3.7)	-3.5 (-5.2 to -1.5)	-8.0 (-25.4 to 9.1)	1.7 (-15.8 to 18.7)	-3.5 (-5.2 to -1.7)
Honduras	447 (334-584)	20 (10-37)	4 (2-8)	472 (355 to 610)	-4.5 (-6.3 to -2.9)	15.9 (11.2 to 20.7)	39.4 (35.5 to 42.7)	-4.2 (-5.9 to -2.6)	-3.9 (-6.2 to -1.7)	-5.0 (-10.7 to 0.6)	4.7 (-1.0 to 10.3)	-3.9 (-6.2 to -1.7)
Hungary	70 (55-84)	4 (1-12)	2 (1-7)	76 (66 to 89)	-10.2 (-11.3 to -9.3)	12.2 (2.5 to 26.1)	31.1 (24.3 to 36.1)	-9.6 (-10.3 to -8.9)	-7.3 (-10.1 to -4.8)	-11.3 (-29.2 to 5.0)	-0.2 (-18.1 to 16.1)	-7.4 (-9.0 to -5.7)
Iceland	1 (1-2)	0 (0-0)	0 (0-0)	1 (1 to 2)	-5.5 (-6.3 to -4.7)	-3.0 (-6.3 to 0.3)	0.7 (-2.7 to 3.4)	-5.5 (-6.3 to -4.7)	-6.4 (-7.7 to -5.2)	-13.7 (-18.2 to -9.5)	-6.2 (-10.7 to -2.0)	-6.5 (-7.7 to -5.2)
India	386,165 (357,891-413,895)	46,287 (38,082-55,290)	2,996 (2,432-3,676)	435,448 (404,177 to 464,480)	-4.8 (-5.4 to -4.4)	22.3 (21.6 to 23.0)	47.4 (46.1 to 48.8)	-4.1 (-4.6 to -3.7)	-5.1 (-5.9 to -4.4)	-5.1 (-6.1 to -4.2)	4.5 (3.4 to 5.4)	-5.0 (-5.9 to -4.4)

	Number of deaths (all ages, both sexes) with 95% uncertainty intervals (2016)				Annualized rate of change of age-standardized rate (%) with 95% uncertainty intervals							
					1990-2006				2006-2016			
	**Drug-susceptible TB**	**MDR TB**	**XDR TB**	**All HIV-negative TB**	**Drug-susceptible TB**	**MDR TB**	**XDR TB**	**All HIV-negative TB**	**Drug-susceptible TB**	**MDR TB**	**XDR TB**	**All HIV-negative TB**
Indonesia	88,468 (82,843-94,260)	1,143 (592-2,313)	275 (143-566)	89,887 (84,340 to 95,552)	-4.1 (-4.8 to -3.4)	8.3 (3.3 to 13.9)	46.8 (42.4 to 51.0)	-3.9 (-4.6 to -3.2)	-5.2 (-5.9 to -4.4)	-12.8 (-19.7 to -5.9)	-1.1 (-7.9 to 5.8)	-5.3 (-6.0 to -4.6)
Iran	1,005 (833-1,220)	31 (10-69)	3 (1-7)	1,039 (865 to 1,257)	-3.1 (-5.0 to -1.3)	20.3 (7.8 to 35.7)	24.7 (12.6 to 32.4)	-2.8 (-4.7 to -1.2)	-5.6 (-8.1 to -3.0)	-7.4 (-23.0 to 12.5)	0.6 (-15.0 to 20.5)	-5.6 (-8.1 to -3.2)
Iraq	1,282 (977-1,634)	62 (18-147)	6 (2-14)	1,350 (1,041 to 1,708)	-2.0 (-3.5 to -0.6)	22.9 (11.4 to 37.4)	34.1 (23.5 to 40.9)	-1.7 (-3.2 to -0.3)	-4.9 (-7.2 to -1.9)	-5.4 (-21.6 to 14.1)	2.7 (-13.6 to 22.1)	-4.9 (-7.1 to -1.9)
Ireland	29 (24-34)	1 (1-1)	0 (0-0)	30 (25 to 36)	-5.5 (-6.2 to -4.8)	1.7 (-1.6 to 4.9)	22.5 (20.0 to 25.1)	-5.4 (-6.0 to -4.7)	-4.9 (-6.7 to -3.1)	-5.5 (-9.6 to -1.1)	2.0 (-2.2 to 6.3)	-4.9 (-6.7 to -3.1)
Israel	25 (20-31)	3 (2-4)	1 (1-1)	28 (23 to 35)	-5.7 (-6.7 to -4.7)	-1.0 (-3.7 to 2.0)	29.3 (27.5 to 31.1)	-5.1 (-6.1 to -4.2)	-7.0 (-9.2 to -4.8)	-8.4 (-11.8 to -4.8)	-1.0 (-4.4 to 2.7)	-7.0 (-9.1 to -4.8)
Italy	396 (350-448)	28 (19-38)	9 (6-13)	432 (384 to 489)	-6.6 (-7.2 to -6.0)	-1.8 (-4.1 to 0.9)	24.9 (23.2 to 26.4)	-6.3 (-6.8 to -5.7)	-2.9 (-4.1 to -1.7)	-5.2 (-8.5 to -2.0)	2.3 (-1.0 to 5.4)	-3.0 (-4.2 to -1.8)
Jamaica	15 (11-18)	1 (0-2)	0 (0-0)	15 (13 to 19)	-5.4 (-6.8 to -4.3)	19.0 (7.1 to 33.2)	21.2 (9.5 to 28.7)	-5.0 (-6.1 to -4.0)	-1.4 (-4.1 to 1.1)	-4.0 (-20.8 to 12.1)	5.7 (-11.1 to 21.7)	-1.5 (-3.8 to 0.8)
Japan	3,823 (3,550-4,082)	91 (72-113)	30 (24-38)	3,944 (3,665 to 4,214)	-5.6 (-5.7 to -5.4)	0.1 (-0.7 to 0.8)	26.5 (25.0 to 27.9)	-5.4 (-5.6 to -5.3)	-4.3 (-4.9 to -3.7)	-6.4 (-7.5 to -5.3)	1.1 (-0.1 to 2.2)	-4.3 (-5.0 to -3.8)
Jordan	19 (11-26)	4 (1-10)	0 (0-1)	23 (18 to 29)	-5.8 (-7.9 to -4.1)	17.3 (8.4 to 32.8)	28.2 (23.3 to 32.1)	-4.7 (-6.6 to -3.2)	-7.3 (-12.3 to -3.7)	-6.9 (-22.9 to 5.3)	1.1 (-14.9 to 13.3)	-7.1 (-9.4 to -4.6)
Kazakhstan	547 (401-721)	344 (250-453)	199 (144-265)	1,090 (883 to 1,327)	-0.9 (-3.5 to 1.3)	27.1 (19.4 to 38.8)	61.7 (59.3 to 63.7)	3.5 (2.3 to 4.7)	-13.5 (-17.7 to -8.9)	-16.7 (-21.0 to -12.0)	-5.6 (-9.9 to -0.8)	-13.7 (-16.2 to -11.3)
Kenya	9,621 (7,500-11,994)	320 (225-438)	5 (4-8)	9,947 (7,758 to 12,425)	5.1 (3.7 to 6.2)	29.9 (27.7 to 31.9)	27.9 (25.5 to 30.1)	5.3 (3.9 to 6.4)	-1.1 (-2.0 to -0.3)	-0.7 (-2.9 to 1.7)	10.5 (8.3 to 12.9)	-1.1 (-2.0 to -0.2)
Kiribati	42 (32-51)	1 (0-10)	0 (0-2)	44 (36 to 52)	-3.2 (-5.0 to -2.0)	5.6 (-19.8 to 34.8)	48.2 (16.7 to 60.8)	-3.0 (-4.0 to -2.0)	-2.4 (-4.8 to 0.3)	-4.8 (-40.0 to 30.2)	7.0 (-28.2 to 41.9)	-2.4 (-3.8 to -1.2)
Kuwait	17 (13-23)	1 (0-1)	0 (0-0)	18 (13 to 24)	-3.6 (-4.9 to -2.2)	19.4 (9.4 to 33.5)	27.0 (20.0 to 32.7)	-3.3 (-4.6 to -2.0)	-9.0 (-12.1 to -6.1)	-9.6 (-21.6 to 3.5)	-1.6 (-13.6 to 11.5)	-9.0 (-12.1 to -6.2)
Kyrgyzstan	212 (101-340)	174 (99-252)	101 (57-144)	486 (425 to 556)	-0.8 (-5.0 to 2.0)	41.5 (33.8 to 55.0)	60.8 (56.9 to 63.2)	4.0 (3.3 to 4.7)	-7.7 (-17.7 to 2.2)	-9.4 (-17.3 to -1.1)	1.7 (-6.2 to 10.1)	-7.1 (-8.5 to -5.6)
Laos	1,491 (1,219-1,886)	19 (2-85)	5 (0-20)	1,515 (1,245 to 1,920)	-4.2 (-5.2 to -3.2)	8.4 (-5.1 to 23.0)	47.7 (32.3 to 57.1)	-4.0 (-4.9 to -3.1)	-8.4 (-10.0 to -5.8)	-16.6 (-36.2 to 2.1)	-4.9 (-24.4 to 13.8)	-8.5 (-10.1 to -6.0)
Latvia	46 (37-57)	9 (7-13)	5 (4-7)	60 (50 to 74)	-0.4 (-1.4 to 0.6)	1.8 (-0.7 to 4.8)	48.1 (46.8 to 49.3)	0.4 (-0.4 to 1.2)	-8.2 (-10.6 to -5.8)	-12.9 (-16.2 to -9.7)	-1.7 (-5.1 to 1.5)	-8.7 (-10.8 to -6.4)

	Number of deaths (all ages, both sexes) with 95% uncertainty intervals (2016)				Annualized rate of change of age-standardized rate (%) with 95% uncertainty intervals							
					1990-2006				2006-2016			
	Drug-susceptible TB	MDR TB	XDR TB	All HIV-negative TB	Drug-susceptible TB	MDR TB	XDR TB	All HIV-negative TB	Drug-susceptible TB	MDR TB	XDR TB	All HIV-negative TB
Lebanon	44 (31-58)	3 (0-10)	0 (0-1)	47 (35 to 61)	-11.2 (-12.9 to -8.6)	6.3 (-5.6 to 22.4)	22.5 (13.2 to 29.1)	-10.9 (-12.4 to -8.3)	-5.9 (-8.5 to -3.4)	-4.4 (-22.7 to 9.8)	3.6 (-14.7 to 17.9)	-5.8 (-7.8 to -3.5)
Lesotho	2,996 (2,269-3,896)	293 (164-483)	5 (3-8)	3,294 (2,504 to 4,280)	5.0 (1.2 to 7.0)	34.2 (25.8 to 43.5)	45.9 (39.6 to 51.0)	5.5 (1.8 to 7.6)	-1.9 (-4.5 to 0.8)	-1.6 (-11.3 to 9.5)	9.6 (-0.2 to 20.6)	-1.8 (-4.2 to 0.5)
Liberia	1,281 (1,002-1,621)	63 (19-148)	1 (0-3)	1,345 (1,059 to 1,685)	-2.3 (-4.1 to -0.5)	11.4 (3.2 to 19.7)	35.6 (27.8 to 41.2)	-2.0 (-3.8 to -0.2)	-3.6 (-5.0 to -2.0)	-5.3 (-15.7 to 5.2)	5.8 (-4.5 to 16.4)	-3.7 (-4.9 to -2.2)
Libya	120 (55-159)	6 (0-24)	1 (0-2)	126 (57 to 165)	-4.9 (-6.2 to -3.8)	7.9 (-6.7 to 22.3)	26.0 (10.2 to 34.5)	-4.7 (-5.8 to -3.6)	-1.8 (-4.1 to -0.1)	1.3 (-16.4 to 19.3)	9.3 (-8.4 to 27.4)	-1.6 (-3.4 to -0.2)
Lithuania	122 (103-146)	42 (33-52)	24 (19-30)	188 (164 to 219)	0.9 (-0.0 to 1.8)	7.5 (4.4 to 11.2)	52.6 (51.4 to 53.7)	2.5 (1.6 to 3.2)	-7.5 (-9.4 to -5.7)	-8.4 (-10.6 to -6.1)	2.7 (0.5 to 5.0)	-6.9 (-8.7 to -5.1)
Luxembourg	2 (2-2)	0 (0-0)	0 (0-0)	2 (2 to 2)	-6.6 (-7.4 to -5.8)	-1.1 (-4.3 to 2.1)	1.9 (-1.3 to 4.7)	-6.6 (-7.4 to -5.8)	-4.2 (-5.8 to -2.8)	6.9 (2.5 to 11.6)	14.4 (9.9 to 19.0)	-4.2 (-5.7 to -2.8)
Macedonia	33 (29-37)	1 (0-2)	0 (0-1)	34 (31 to 38)	-5.8 (-6.6 to -5.0)	11.3 (0.5 to 25.7)	28.2 (17.6 to 35.5)	-5.7 (-6.5 to -4.9)	-7.5 (-8.8 to -6.2)	-4.0 (-18.6 to 13.2)	7.2 (-7.5 to 24.4)	-7.3 (-8.5 to -6.2)
Madagascar	3,523 (2,720-4,454)	89 (27-219)	2 (0-4)	3,613 (2,791 to 4,556)	-3.2 (-4.5 to -1.8)	16.9 (9.5 to 24.7)	22.1 (16.8 to 26.3)	-3.1 (-4.4 to -1.7)	-1.9 (-3.9 to 0.1)	-0.7 (-11.2 to 8.3)	10.5 (-0.1 to 19.4)	-1.9 (-3.8 to 0.1)
Malawi	6,282 (4,490-8,412)	162 (60-337)	3 (1-6)	6,447 (4,597 to 8,658)	4.0 (2.3 to 5.5)	27.1 (20.3 to 35.4)	31.4 (25.2 to 35.8)	4.1 (2.5 to 5.6)	-3.4 (-6.3 to -0.5)	-3.3 (-14.4 to 7.8)	7.9 (-3.2 to 18.9)	-3.4 (-6.2 to -0.6)
Malaysia	1,262 (1,107-1,430)	26 (11-52)	6 (3-13)	1,295 (1,140 to 1,462)	-5.6 (-6.5 to -4.7)	17.1 (6.2 to 30.2)	34.6 (23.8 to 41.8)	-5.3 (-6.2 to -4.5)	-4.4 (-5.8 to -2.9)	-9.3 (-24.0 to 9.6)	2.4 (-12.2 to 21.4)	-4.5 (-5.8 to -3.1)
Maldives	8 (6-10)	0 (0-0)	0 (0-0)	8 (6 to 10)	-13.0 (-14.7 to -8.6)	0.5 (-13.8 to 15.3)	33.3 (18.1 to 42.8)	-12.8 (-14.5 to -8.5)	-7.8 (-10.6 to -4.9)	-16.9 (-33.5 to 2.1)	-5.2 (-21.8 to 13.9)	-8.0 (-10.7 to -5.3)
Mali	1,833 (1,357-2,595)	87 (26-217)	1 (0-4)	1,922 (1,446 to 2,693)	-6.8 (-8.5 to -4.5)	6.8 (-1.4 to 15.4)	30.8 (23.4 to 36.5)	-6.5 (-8.1 to -4.2)	-3.4 (-5.5 to -1.2)	-5.2 (-15.5 to 4.6)	6.0 (-4.3 to 15.8)	-3.5 (-5.5 to -1.5)
Malta	1 (1-1)	0 (0-0)	0 (0-0)	1 (1 to 1)	-6.0 (-6.9 to -5.1)	5.5 (2.2 to 8.7)	3.1 (-0.1 to 5.9)	-6.0 (-6.9 to -5.1)	-4.5 (-6.5 to -2.4)	-20.6 (-25.5 to -16.2)	-13.1 (-18.0 to -8.7)	-4.5 (-6.5 to -2.4)
Marshall Islands	4 (3-5)	0 (0-1)	0 (0-0)	4 (3 to 5)	-3.3 (-5.8 to -1.8)	17.7 (1.7 to 48.1)	43.3 (26.7 to 51.8)	-2.8 (-4.1 to -1.5)	-5.2 (-8.1 to -1.4)	-11.0 (-38.1 to 19.2)	0.7 (-26.3 to 31.0)	-5.5 (-7.1 to -3.6)
Mauritania	314 (213-486)	15 (4-39)	0 (0-1)	330 (224 to 509)	-4.9 (-6.5 to -3.2)	8.6 (0.8 to 17.0)	28.1 (20.6 to 33.6)	-4.6 (-6.2 to -2.8)	-4.4 (-6.7 to -2.4)	-6.3 (-16.2 to 3.1)	4.9 (-5.0 to 14.2)	-4.5 (-6.8 to -2.5)
Mauritius	13 (11-16)	0 (0-0)	0 (0-0)	13 (11 to 16)	-7.3 (-7.9 to -6.6)	11.5 (-0.0 to 24.8)	16.7 (5.5 to 23.4)	-7.2 (-7.8 to -6.5)	-2.3 (-4.1 to -0.4)	-5.3 (-22.2 to 11.3)	6.5 (-10.5 to 23.1)	-2.3 (-4.1 to -0.6)

	Number of deaths (all ages, both sexes) with 95% uncertainty intervals (2016)				Annualized rate of change of age-standardized rate (%) with 95% uncertainty intervals							
					1990-2006				2006-2016			
	**Drug-susceptible TB**	**MDR TB**	**XDR TB**	**All HIV-negative TB**	**Drug-susceptible TB**	**MDR TB**	**XDR TB**	**All HIV-negative TB**	**Drug-susceptible TB**	**MDR TB**	**XDR TB**	**All HIV-negative TB**
Mexico	2,291 (2,163-2,429)	139 (110-172)	29 (23-37)	2,458 (2,332 to 2,594)	-8.9 (-9.1 to -8.6)	18.0 (16.7 to 19.1)	32.7 (31.1 to 34.2)	-8.4 (-8.6 to -8.2)	-4.3 (-5.0 to -3.8)	-5.8 (-7.4 to -4.3)	3.8 (2.2 to 5.3)	-4.4 (-5.0 to -3.8)
Moldova	101 (71-142)	96 (71-128)	56 (41-75)	253 (206 to 325)	1.1 (-0.6 to 2.6)	38.4 (34.5 to 43.2)	57.0 (55.2 to 58.4)	5.4 (4.4 to 6.4)	-10.4 (-14.1 to -6.6)	-9.0 (-12.3 to -5.6)	2.1 (-1.2 to 5.5)	-8.1 (-10.5 to -5.5)
Mongolia	273 (199-359)	23 (5-58)	13 (3-34)	308 (249 to 395)	-0.9 (-2.2 to 0.3)	17.5 (9.0 to 29.4)	49.6 (46.1 to 52.5)	-0.4 (-1.6 to 0.8)	-7.0 (-10.4 to -3.8)	-6.0 (-21.6 to 4.7)	5.2 (-10.5 to 15.8)	-6.6 (-8.9 to -3.9)
Montenegro	4 (3-5)	0 (0-0)	0 (0-0)	4 (3 to 5)	-2.1 (-3.2 to -0.9)	5.3 (-8.0 to 20.5)	12.1 (-0.7 to 20.1)	-2.0 (-3.2 to -0.9)	-4.8 (-6.4 to -2.7)	-9.2 (-29.6 to 9.7)	1.9 (-18.5 to 20.8)	-4.8 (-6.3 to -2.7)
Morocco	11,218 (8,197-21,557)	426 (137-1,094)	40 (13-99)	11,684 (8,668 to 22,474)	-5.5 (-6.6 to -4.4)	18.4 (7.2 to 33.7)	44.7 (31.7 to 52.4)	-5.3 (-6.2 to -4.1)	-4.5 (-6.0 to -2.9)	-5.8 (-20.2 to 13.7)	2.2 (-12.2 to 21.8)	-4.5 (-5.8 to -3.2)
Mozambique	15,223 (11,471-19,962)	1,652 (587-3,544)	28 (10-60)	16,903 (13,089 to 21,808)	1.3 (-0.3 to 2.9)	19.3 (13.3 to 26.5)	42.1 (38.2 to 45.3)	2.0 (0.4 to 3.5)	-1.9 (-4.6 to 0.6)	-2.4 (-12.3 to 5.6)	8.7 (-1.2 to 16.8)	-2.0 (-4.4 to 0.4)
Myanmar	12,423 (10,069-15,463)	1,596 (672-3,058)	384 (167-728)	14,403 (12,365 to 17,579)	-6.3 (-7.5 to -4.8)	13.9 (4.8 to 27.5)	55.7 (52.3 to 58.6)	-5.5 (-6.7 to -4.1)	-9.0 (-11.3 to -6.8)	-8.8 (-18.6 to -0.4)	2.9 (-6.9 to 11.4)	-8.8 (-10.4 to -7.0)
Namibia	791 (524-1,327)	102 (54-179)	2 (1-3)	895 (592 to 1,514)	0.6 (-1.6 to 2.4)	31.5 (23.6 to 40.9)	42.0 (35.3 to 47.0)	1.3 (-0.7 to 3.1)	-7.3 (-10.5 to -3.8)	-7.2 (-16.5 to 4.4)	4.0 (-5.3 to 15.6)	-7.2 (-10.4 to -3.8)
Nepal	4,009 (2,860-6,786)	308 (187-548)	20 (12-36)	4,337 (3,100 to 7,317)	-5.2 (-6.7 to -3.7)	4.0 (1.6 to 6.4)	41.2 (38.0 to 45.2)	-4.9 (-6.3 to -3.4)	-5.6 (-7.2 to -4.0)	-5.5 (-8.2 to -2.9)	4.1 (1.3 to 6.7)	-5.6 (-7.2 to -4.0)
Netherlands	82 (73-92)	3 (2-4)	1 (1-1)	86 (77 to 97)	-4.5 (-5.2 to -3.9)	0.5 (-2.2 to 3.5)	18.8 (16.8 to 20.8)	-4.4 (-5.1 to -3.8)	-6.3 (-7.4 to -5.2)	-2.8 (-6.4 to 1.0)	4.6 (1.1 to 8.4)	-6.1 (-7.2 to -5.0)
New Zealand	15 (13-18)	0 (0-1)	0 (0-0)	16 (14 to 18)	-5.9 (-6.5 to -5.2)	-3.2 (-11.7 to 7.6)	16.8 (11.7 to 20.8)	-5.8 (-6.4 to -5.1)	-4.5 (-5.9 to -3.0)	-2.7 (-14.8 to 8.2)	4.8 (-7.3 to 15.6)	-4.4 (-5.7 to -3.0)
Nicaragua	151 (125-180)	5 (2-8)	1 (0-2)	156 (130 to 186)	-6.2 (-6.9 to -5.4)	5.4 (0.8 to 10.0)	31.2 (27.2 to 34.7)	-6.0 (-6.7 to -5.2)	-5.0 (-6.9 to -3.1)	-5.8 (-12.1 to 0.5)	3.8 (-2.4 to 10.1)	-5.0 (-6.7 to -3.1)
Niger	5,401 (4,034-6,998)	267 (77-671)	5 (1-12)	5,673 (4,256 to 7,262)	-2.1 (-3.5 to -0.2)	11.9 (4.1 to 20.0)	35.2 (27.2 to 41.3)	-1.7 (-3.0 to 0.1)	-2.0 (-3.9 to -0.3)	-3.9 (-14.1 to 5.8)	7.3 (-2.9 to 17.0)	-2.1 (-3.9 to -0.6)
Nigeria	31,272 (23,625-42,670)	3,078 (1,298-6,071)	52 (22-103)	34,403 (26,533 to 46,550)	-3.6 (-5.2 to -2.2)	25.3 (18.7 to 33.1)	38.7 (33.9 to 42.7)	-2.9 (-4.3 to -1.6)	-6.7 (-9.0 to -4.3)	-7.7 (-17.7 to 2.1)	3.4 (-6.5 to 13.3)	-6.8 (-8.8 to -4.7)
North Korea	2,314 (1,953-2,768)	143 (79-225)	34 (19-54)	2,490 (2,109 to 2,969)	-0.9 (-2.3 to 0.4)	31.2 (27.0 to 35.1)	40.5 (36.7 to 43.7)	-0.5 (-1.8 to 0.9)	-2.9 (-4.5 to -1.3)	-4.3 (-9.1 to 0.6)	7.4 (2.6 to 12.3)	-2.9 (-4.4 to -1.4)

	Number of deaths (all ages, both sexes) with 95% uncertainty intervals (2016)				Annualized rate of change of age-standardized rate (%) with 95% uncertainty intervals							
					1990-2006				2006-2016			
	**Drug-susceptible TB**	**MDR TB**	**XDR TB**	**All HIV-negative TB**	**Drug-susceptible TB**	**MDR TB**	**XDR TB**	**All HIV-negative TB**	**Drug-susceptible TB**	**MDR TB**	**XDR TB**	**All HIV-negative TB**
Northern Mariana Islands	1 (1-1)	0 (0-0)	0 (0-0)	1 (1 to 1)	-8.7 (-11.2 to -6.1)	3.6 (-10.0 to 34.0)	35.0 (27.5 to 39.8)	-7.7 (-9.5 to -5.6)	3.2 (-0.2 to 6.4)	-8.0 (-33.6 to 8.9)	3.7 (-21.9 to 20.7)	2.0 (0.1 to 3.7)
Norway	34 (28-40)	2 (1-3)	1 (0-1)	36 (31 to 43)	-3.6 (-4.3 to -2.8)	-1.3 (-3.9 to 1.7)	25.0 (22.9 to 26.9)	-3.4 (-4.1 to -2.7)	-4.8 (-6.6 to -3.2)	-5.0 (-9.1 to -1.2)	2.5 (-1.7 to 6.3)	-4.7 (-6.5 to -3.0)
Oman	23 (19-28)	1 (0-3)	0 (0-0)	24 (19 to 29)	-5.7 (-8.0 to -4.2)	0.3 (-10.3 to 15.1)	21.2 (16.5 to 25.3)	-5.5 (-7.7 to -4.1)	-4.0 (-6.4 to -2.2)	-3.6 (-15.4 to 7.4)	4.5 (-7.4 to 15.4)	-4.0 (-6.3 to -2.3)
Pakistan	44,042 (35,157-55,982)	4,994 (3,469-6,884)	323 (223-449)	49,359 (39,663 to 62,151)	-1.8 (-3.8 to -0.4)	30.2 (27.8 to 32.5)	46.9 (44.8 to 48.9)	-1.0 (-3.1 to 0.3)	-4.5 (-6.0 to -3.1)	-5.1 (-7.9 to -2.4)	4.4 (1.7 to 7.2)	-4.5 (-6.0 to -3.1)
Palestine	2 (2-3)	0 (0-0)	0 (0-0)	3 (2 to 3)	-9.2 (-10.9 to -7.9)	3.3 (-10.7 to 19.3)	8.6 (-6.2 to 17.2)	-9.0 (-10.3 to -7.8)	-5.2 (-7.4 to -3.4)	-2.7 (-19.3 to 14.7)	5.3 (-11.3 to 22.8)	-5.1 (-6.6 to -3.7)
Panama	183 (157-214)	7 (3-12)	1 (1-2)	191 (165 to 224)	-4.3 (-5.1 to -3.5)	10.7 (5.8 to 15.3)	34.1 (29.1 to 38.0)	-4.0 (-4.8 to -3.2)	-3.4 (-5.1 to -1.6)	-4.7 (-11.1 to 1.4)	4.9 (-1.4 to 11.0)	-3.4 (-5.0 to -1.7)
Papua New Guinea	360 (253-464)	40 (8-109)	10 (2-26)	409 (331 to 501)	-3.2 (-8.2 to -1.2)	21.0 (4.9 to 53.0)	47.1 (26.8 to 56.0)	-2.2 (-3.5 to -0.9)	-4.4 (-8.4 to 4.4)	-8.8 (-31.1 to 23.7)	3.0 (-19.3 to 35.4)	-4.8 (-6.4 to -3.2)
Paraguay	244 (207-283)	9 (6-12)	2 (1-2)	254 (216 to 296)	-2.5 (-3.3 to -1.7)	15.0 (13.1 to 16.8)	33.5 (31.6 to 35.2)	-2.3 (-3.0 to -1.5)	-2.2 (-4.0 to -0.6)	-4.2 (-6.8 to -1.6)	5.5 (2.8 to 8.1)	-2.3 (-4.0 to -0.6)
Peru	1,257 (993-1,612)	181 (129-251)	38 (27-53)	1,476 (1,175 to 1,900)	-10.9 (-12.0 to -9.3)	0.7 (-3.9 to 6.7)	43.3 (41.4 to 45.2)	-10.1 (-11.2 to -8.5)	-6.0 (-8.5 to -3.7)	-6.3 (-10.0 to -2.5)	3.3 (-0.4 to 7.1)	-5.9 (-8.3 to -3.6)
Philippines	25,664 (21,214-30,341)	1,962 (599-4,636)	473 (145-1,134)	28,098 (24,130 to 32,488)	-3.8 (-5.3 to -2.9)	25.0 (14.6 to 38.2)	53.3 (43.8 to 59.6)	-3.1 (-3.9 to -2.5)	-4.0 (-6.3 to -1.3)	-7.6 (-24.5 to 9.7)	4.2 (-12.8 to 21.5)	-4.2 (-5.8 to -2.6)
Poland	580 (499-688)	7 (4-11)	4 (2-7)	591 (509 to 701)	-7.1 (-7.7 to -6.5)	-4.2 (-11.8 to 6.3)	25.7 (22.7 to 28.4)	-7.1 (-7.7 to -6.4)	-6.2 (-7.8 to -4.4)	-9.7 (-16.4 to -3.1)	1.4 (-5.3 to 8.0)	-6.2 (-7.8 to -4.5)
Portugal	214 (190-246)	6 (4-9)	2 (1-3)	222 (198 to 256)	-5.5 (-6.1 to -4.9)	-4.0 (-6.4 to -1.2)	28.5 (26.8 to 30.0)	-5.4 (-6.0 to -4.8)	-4.4 (-5.7 to -3.0)	-6.3 (-9.9 to -2.7)	1.2 (-2.4 to 4.8)	-4.4 (-5.7 to -3.0)
Puerto Rico	23 (19-27)	0 (0-1)	0 (0-0)	23 (20 to 27)	-8.8 (-9.5 to -8.1)	8.4 (-1.4 to 20.6)	19.2 (10.9 to 25.1)	-8.5 (-9.1 to -7.9)	-3.2 (-4.9 to -1.4)	-13.1 (-30.4 to 1.4)	-3.5 (-20.7 to 11.0)	-3.5 (-5.0 to -1.7)
Qatar	8 (5-11)	0 (0-1)	0 (0-0)	8 (6 to 11)	-9.7 (-11.7 to -7.8)	-0.6 (-11.2 to 14.1)	17.3 (10.6 to 22.3)	-9.5 (-11.5 to -7.6)	-2.1 (-6.5 to 2.0)	1.6 (-14.9 to 15.8)	9.6 (-6.9 to 23.8)	-1.9 (-6.4 to 2.1)
Romania	917 (772-1,069)	55 (24-110)	32 (14-65)	1,004 (875 to 1,164)	0.2 (-0.8 to 1.0)	7.0 (-1.7 to 16.8)	42.4 (37.1 to 46.8)	0.6 (-0.2 to 1.3)	-5.8 (-7.6 to -4.0)	-8.0 (-18.1 to 2.3)	3.1 (-7.0 to 13.4)	-5.7 (-7.3 to -4.2)
Russia	8,079 (5,386-11,778)	3,196 (1,950-4,759)	1,848 (1,131-2,743)	13,123 (9,081 to 18,405)	3.8 (2.4 to 5.1)	9.0 (6.0 to 12.8)	54.9 (53.3 to 56.3)	5.1 (3.9 to 6.3)	-8.2 (-12.2 to -4.3)	-6.7 (-11.8 to -2.4)	4.4 (-0.6 to 8.7)	-6.7 (-10.5 to -3.1)

	Number of deaths (all ages, both sexes) with 95% uncertainty intervals (2016)				Annualized rate of change of age-standardized rate (%) with 95% uncertainty intervals							
					1990-2006				2006-2016			
	**Drug-susceptible TB**	**MDR TB**	**XDR TB**	**All HIV-negative TB**	**Drug-susceptible TB**	**MDR TB**	**XDR TB**	**All HIV-negative TB**	**Drug-susceptible TB**	**MDR TB**	**XDR TB**	**All HIV-negative TB**
Rwanda	4,505 (3,668-5,539)	233 (123-393)	4 (2-7)	4,741 (3,883 to 5,828)	-6.9 (-8.4 to -4.3)	21.2 (13.6 to 29.6)	36.7 (29.9 to 41.7)	-6.5 (-8.0 to -3.9)	-4.5 (-6.2 to -2.8)	-5.2 (-13.6 to 5.5)	6.0 (-2.4 to 16.7)	-4.6 (-6.2 to -3.1)
Saint Lucia	5 (4-5)	0 (0-0)	0 (0-0)	5 (5 to 6)	-2.6 (-3.4 to -1.8)	3.5 (-9.2 to 17.2)	27.1 (13.9 to 35.5)	-2.5 (-3.1 to -1.8)	-4.4 (-5.5 to -3.2)	-9.1 (-26.8 to 7.7)	0.5 (-17.1 to 17.3)	-4.5 (-5.5 to -3.4)
Saint Vincent and the Grenadines	3 (3-3)	0 (0-0)	0 (0-0)	3 (3 to 3)	-1.8 (-2.7 to -1.0)	4.5 (-7.8 to 16.7)	26.3 (13.3 to 34.6)	-1.7 (-2.5 to -1.0)	-2.0 (-3.3 to -0.6)	-6.8 (-23.8 to 8.5)	2.9 (-14.2 to 18.1)	-2.0 (-3.3 to -0.7)
Samoa	5 (4-10)	0 (0-0)	0 (0-0)	5 (4 to 10)	-6.0 (-7.4 to -4.6)	-2.8 (-31.1 to 26.3)	19.3 (-15.1 to 31.4)	-5.9 (-7.3 to -4.7)	-3.2 (-4.9 to -1.4)	-4.4 (-40.2 to 30.0)	7.3 (-28.4 to 41.8)	-3.2 (-4.8 to -1.5)
Sao Tome and Principe	14 (11-18)	1 (0-2)	0 (0-0)	15 (12 to 19)	-1.5 (-3.2 to -0.1)	12.4 (4.3 to 20.5)	28.1 (20.4 to 33.7)	-1.1 (-2.9 to 0.1)	-4.9 (-7.1 to -2.4)	-6.5 (-17.5 to 4.2)	4.6 (-6.3 to 15.4)	-4.9 (-7.1 to -2.6)
Saudi Arabia	778 (670-878)	58 (24-130)	5 (2-12)	842 (747 to 933)	-4.5 (-6.5 to -3.3)	21.2 (15.1 to 27.5)	36.3 (31.0 to 40.9)	-4.1 (-6.1 to -2.9)	-5.8 (-7.7 to -4.4)	-5.4 (-12.8 to 2.4)	2.6 (-4.8 to 10.4)	-5.7 (-7.5 to -4.5)
Senegal	4,782 (4,100-5,610)	131 (56-245)	2 (1-4)	4,915 (4,221 to 5,745)	-2.3 (-3.2 to -1.3)	19.5 (11.8 to 27.5)	31.8 (25.0 to 36.7)	-2.1 (-3.0 to -1.2)	-3.1 (-4.3 to -1.8)	-3.9 (-13.8 to 6.6)	7.3 (-2.7 to 17.8)	-3.1 (-4.3 to -1.9)
Serbia	132 (119-150)	2 (1-5)	1 (0-3)	135 (123 to 152)	-2.7 (-3.7 to -1.6)	5.8 (-3.7 to 21.1)	23.4 (19.6 to 27.0)	-2.6 (-3.7 to -1.5)	-6.9 (-8.1 to -5.6)	-3.4 (-15.9 to 7.5)	7.8 (-4.7 to 18.6)	-6.8 (-8.0 to -5.5)
Seychelles	1 (1-2)	0 (0-0)	0 (0-0)	1 (1 to 2)	-6.1 (-7.0 to -5.1)	-2.6 (-16.5 to 12.0)	7.5 (-7.6 to 16.4)	-6.0 (-7.0 to -5.1)	-4.4 (-6.2 to -2.7)	-6.4 (-26.7 to 12.4)	5.3 (-15.0 to 24.2)	-4.4 (-6.2 to -2.7)
Sierra Leone	2,203 (1,867-2,560)	82 (23-200)	1 (0-3)	2,287 (1,942 to 2,659)	1.6 (0.3 to 2.8)	10.1 (1.9 to 18.0)	35.2 (28.3 to 40.5)	1.8 (0.5 to 2.9)	-4.8 (-6.3 to -3.2)	-5.2 (-15.9 to 4.7)	5.9 (-4.8 to 15.8)	-4.8 (-6.2 to -3.3)
Singapore	53 (43-65)	1 (1-1)	0 (0-0)	54 (44 to 66)	-8.1 (-9.1 to -6.9)	-4.8 (-8.2 to -1.5)	21.4 (18.7 to 23.7)	-8.0 (-9.1 to -6.9)	-6.7 (-9.2 to -4.3)	-1.8 (-6.7 to 2.9)	5.6 (0.8 to 10.4)	-6.6 (-9.1 to -4.2)
Slovakia	29 (25-37)	0 (0-2)	0 (0-1)	30 (26 to 37)	-6.0 (-6.7 to -5.2)	-1.5 (-10.7 to 11.5)	23.8 (19.4 to 27.1)	-5.9 (-6.6 to -5.1)	-7.4 (-9.2 to -5.1)	-12.9 (-30.8 to -0.5)	-1.8 (-19.6 to 10.6)	-7.4 (-9.2 to -5.3)
Slovenia	14 (12-17)	0 (0-0)	0 (0-0)	14 (12 to 17)	-7.3 (-8.2 to -6.5)	-5.8 (-16.6 to 7.0)	17.3 (10.4 to 22.4)	-7.3 (-8.1 to -6.4)	-6.6 (-8.5 to -4.5)	-24.2 (-42.8 to -10.9)	-13.0 (-31.6 to 0.2)	-6.7 (-8.6 to -4.7)
Solomon Islands	76 (56-96)	2 (0-15)	0 (0-4)	79 (62 to 98)	-3.6 (-5.5 to -2.2)	7.2 (-18.5 to 36.0)	43.7 (12.8 to 56.4)	-3.4 (-4.6 to -2.1)	-4.8 (-7.0 to -2.2)	-7.5 (-39.5 to 26.1)	4.2 (-27.7 to 37.8)	-4.8 (-6.4 to -3.0)
Somalia	8,620 (6,403-11,399)	1,516 (680-2,759)	26 (12-47)	10,161 (7,817 to 13,226)	4.2 (2.5 to 5.9)	38.3 (31.5 to 46.2)	46.7 (41.4 to 50.9)	5.2 (3.8 to 6.7)	-0.6 (-2.7 to 1.7)	-0.3 (-9.9 to 9.2)	10.8 (1.2 to 20.4)	-0.5 (-1.8 to 0.7)
South Africa	21,002 (18,977-23,385)	1,303 (777-2,120)	22 (13-36)	22,326 (20,329 to 24,780)	1.5 (-0.8 to 2.7)	13.0 (8.5 to 17.4)	36.6 (33.2 to 39.9)	1.9 (-0.4 to 3.0)	-5.9 (-7.1 to -4.4)	-7.2 (-12.2 to -2.5)	4.0 (-1.0 to 8.7)	-5.9 (-7.1 to -4.6)

	Number of deaths (all ages, both sexes) with 95% uncertainty intervals (2016)				Annualized rate of change of age-standardized rate (%) with 95% uncertainty intervals							
					1990-2006				2006-2016			
	**Drug-susceptible TB**	**MDR TB**	**XDR TB**	**All HIV-negative TB**	**Drug-susceptible TB**	**MDR TB**	**XDR TB**	**All HIV-negative TB**	**Drug-susceptible TB**	**MDR TB**	**XDR TB**	**All HIV-negative TB**
South Korea	3,200 (2,435-4,099)	125 (71-205)	42 (24-68)	3,367 (2,560 to 4,246)	-8.5 (-9.5 to -7.2)	0.9 (-2.5 to 4.2)	40.6 (38.0 to 42.8)	-8.2 (-9.1 to -6.9)	-5.0 (-7.9 to -2.5)	-8.2 (-13.1 to -3.5)	-0.8 (-5.6 to 3.9)	-5.1 (-7.9 to -2.6)
South Sudan	7,204 (5,284-9,842)	440 (139-1,071)	7 (2-18)	7,651 (5,687 to 10,418)	4.4 (2.7 to 6.1)	24.5 (16.2 to 32.9)	35.9 (27.5 to 41.7)	4.7 (3.1 to 6.5)	0.9 (-1.0 to 2.9)	1.9 (-8.6 to 12.6)	13.0 (2.5 to 23.7)	1.0 (-0.9 to 2.9)
Spain	385 (335-437)	10 (6-15)	3 (2-5)	398 (347 to 451)	-6.9 (-7.4 to -6.4)	-5.2 (-8.3 to -1.9)	21.4 (18.6 to 23.9)	-6.8 (-7.3 to -6.3)	-5.1 (-6.5 to -3.8)	-6.1 (-10.5 to -2.2)	1.4 (-3.0 to 5.3)	-5.1 (-6.5 to -3.8)
Sri Lanka	666 (515-855)	5 (1-19)	1 (0-4)	673 (518 to 863)	-5.4 (-6.4 to -4.5)	7.1 (-5.4 to 21.7)	24.6 (13.4 to 32.0)	-5.4 (-6.3 to -4.5)	-8.0 (-10.6 to -5.5)	-10.5 (-29.8 to 6.3)	1.3 (-18.1 to 18.1)	-8.0 (-10.6 to -5.4)
Sudan	4,556 (2,379-7,303)	231 (21-1,001)	21 (2-93)	4,808 (2,603 to 7,475)	-5.4 (-6.6 to -4.4)	7.6 (-6.7 to 21.7)	41.4 (25.9 to 50.5)	-5.2 (-6.0 to -4.2)	-5.3 (-7.2 to -3.7)	-3.3 (-21.1 to 14.2)	4.7 (-13.1 to 22.2)	-5.2 (-6.4 to -4.0)
Suriname	10 (9-12)	0 (0-1)	0 (0-0)	10 (9 to 12)	-0.9 (-1.7 to -0.1)	4.7 (-7.9 to 17.3)	25.9 (12.5 to 34.4)	-0.8 (-1.6 to -0.0)	-5.0 (-6.6 to -3.4)	-9.6 (-27.0 to 6.8)	0.0 (-17.4 to 16.4)	-5.1 (-6.6 to -3.5)
Swaziland	687 (413-1,016)	148 (54-300)	3 (1-5)	837 (544 to 1,192)	4.0 (-0.1 to 6.8)	23.6 (16.4 to 31.1)	47.0 (42.8 to 50.3)	5.0 (1.0 to 7.8)	-6.2 (-10.5 to -1.7)	-4.9 (-15.6 to 4.5)	6.2 (-4.4 to 15.7)	-6.0 (-9.6 to -1.8)
Sweden	71 (62-82)	5 (3-7)	2 (1-2)	78 (68 to 89)	-4.7 (-5.4 to -4.2)	0.7 (-2.2 to 3.4)	24.4 (21.6 to 26.9)	-4.5 (-5.1 to -3.9)	-5.7 (-7.1 to -4.4)	-2.8 (-6.3 to 0.6)	4.7 (1.2 to 8.0)	-5.4 (-6.8 to -4.0)
Switzerland	35 (27-44)	2 (1-3)	1 (0-1)	38 (29 to 48)	-7.1 (-7.8 to -6.3)	-2.2 (-5.2 to 0.7)	19.8 (17.7 to 22.0)	-6.9 (-7.6 to -6.2)	-4.6 (-7.2 to -2.1)	-1.1 (-5.4 to 3.0)	6.4 (2.1 to 10.5)	-4.3 (-6.9 to -1.8)
Syria	45 (26-58)	9 (1-27)	1 (0-2)	55 (46 to 64)	-8.4 (-10.4 to -6.5)	14.8 (4.6 to 30.1)	24.8 (18.4 to 29.2)	-7.2 (-8.5 to -5.5)	-3.9 (-9.0 to -1.1)	-4.4 (-21.1 to 6.7)	3.6 (-13.1 to 14.7)	-3.9 (-5.6 to -2.5)
Taiwan	869 (745-995)	36 (20-58)	9 (5-14)	913 (787 to 1,044)	-8.0 (-8.9 to -6.8)	19.4 (15.6 to 23.2)	31.9 (28.2 to 35.1)	-7.7 (-8.6 to -6.5)	-4.7 (-6.3 to -3.2)	-6.9 (-12.1 to -2.0)	4.8 (-0.4 to 9.7)	-4.8 (-6.3 to -3.3)
Tajikistan	291 (221-412)	79 (49-122)	46 (29-71)	417 (337 to 557)	1.2 (-2.2 to 3.3)	38.5 (29.4 to 50.6)	54.8 (47.2 to 59.4)	3.3 (2.2 to 4.5)	-8.0 (-12.3 to -2.0)	-10.1 (-19.3 to 3.3)	1.0 (-8.1 to 14.4)	-7.8 (-9.9 to -5.4)
Tanzania	15,122 (12,107-19,203)	593 (185-1,434)	10 (3-24)	15,726 (12,675 to 19,851)	2.5 (0.9 to 3.8)	25.4 (18.9 to 33.5)	30.6 (25.9 to 34.4)	2.7 (1.2 to 4.0)	-1.6 (-3.5 to 0.2)	-0.7 (-11.4 to 8.5)	10.5 (-0.3 to 19.7)	-1.6 (-3.5 to 0.1)
Thailand	5,941 (4,910-7,130)	431 (136-999)	104 (32-242)	6,476 (5,604 to 7,620)	-9.1 (-10.3 to -7.8)	-3.2 (-11.6 to 7.7)	39.3 (35.2 to 42.7)	-8.9 (-10.1 to -7.6)	-6.7 (-8.9 to -4.9)	-2.2 (-14.8 to 8.1)	9.5 (-3.1 to 19.8)	-6.3 (-7.8 to -4.9)
The Bahamas	9 (8-11)	0 (0-1)	0 (0-0)	10 (8 to 12)	-4.0 (-5.0 to -2.9)	13.2 (1.6 to 26.7)	25.0 (13.4 to 32.6)	-3.9 (-4.7 to -2.8)	-1.7 (-3.6 to 0.0)	1.5 (-15.7 to 17.1)	11.1 (-6.0 to 26.7)	-1.6 (-3.2 to 0.1)
The Gambia	438 (323-635)	21 (6-52)	0 (0-1)	459 (338 to 672)	-1.1 (-2.3 to -0.1)	12.8 (4.8 to 20.5)	34.9 (26.2 to 40.5)	-0.8 (-1.9 to 0.2)	-3.0 (-4.4 to -1.6)	-5.0 (-15.1 to 5.0)	6.2 (-3.9 to 16.1)	-3.1 (-4.4 to -1.8)

	Number of deaths (all ages, both sexes) with 95% uncertainty intervals (2016)				Annualized rate of change of age-standardized rate (%) with 95% uncertainty intervals							
					1990-2006				2006-2016			
	**Drug-susceptible TB**	**MDR TB**	**XDR TB**	**All HIV-negative TB**	**Drug-susceptible TB**	**MDR TB**	**XDR TB**	**All HIV-negative TB**	**Drug-susceptible TB**	**MDR TB**	**XDR TB**	**All HIV-negative TB**
Timor-Leste	148 (105-206)	2 (0-9)	0 (0-2)	151 (107 to 211)	-5.8 (-7.6 to -3.9)	6.9 (-6.9 to 22.0)	43.8 (29.8 to 52.4)	-5.7 (-7.3 to -3.8)	-6.8 (-8.6 to -4.5)	-14.7 (-34.2 to 2.1)	-2.9 (-22.5 to 13.9)	-6.9 (-8.8 to -4.8)
Togo	2,034 (1,571-2,472)	97 (26-232)	2 (0-4)	2,132 (1,664 to 2,562)	-0.6 (-1.9 to 0.7)	13.0 (5.1 to 21.3)	35.0 (27.2 to 40.6)	-0.3 (-1.6 to 0.9)	-2.9 (-4.4 to -1.1)	-4.6 (-15.6 to 5.5)	6.6 (-4.4 to 16.7)	-2.9 (-4.4 to -1.2)
Tonga	1 (1-2)	0 (0-0)	0 (0-0)	1 (1 to 2)	-3.2 (-5.7 to -1.4)	6.1 (-18.5 to 35.2)	26.4 (-5.6 to 38.6)	-3.0 (-4.7 to -1.4)	-3.3 (-6.0 to -0.3)	-6.4 (-42.0 to 27.6)	5.3 (-30.2 to 39.3)	-3.4 (-5.5 to -1.3)
Trinidad and Tobago	21 (18-24)	0 (0-1)	0 (0-0)	21 (18 to 25)	-3.2 (-4.0 to -2.4)	2.4 (-11.4 to 16.8)	22.5 (8.0 to 30.7)	-3.1 (-3.8 to -2.4)	-3.5 (-5.0 to -2.1)	-7.9 (-25.3 to 9.0)	1.7 (-15.7 to 18.6)	-3.6 (-5.0 to -2.2)
Tunisia	293 (215-397)	6 (2-15)	1 (0-1)	300 (220 to 404)	-7.3 (-8.6 to -5.5)	13.6 (1.0 to 28.0)	25.0 (11.6 to 32.3)	-7.1 (-8.4 to -5.4)	-4.6 (-6.4 to -2.7)	-6.8 (-22.4 to 12.6)	1.2 (-14.4 to 20.7)	-4.7 (-6.5 to -2.8)
Turkey	734 (604-890)	62 (29-112)	6 (3-10)	802 (667 to 954)	-11.4 (-13.0 to -8.5)	16.9 (5.0 to 32.3)	26.8 (16.4 to 33.7)	-10.8 (-12.1 to -8.1)	-6.2 (-8.7 to -3.1)	-6.9 (-21.0 to 11.5)	1.1 (-12.9 to 19.5)	-6.2 (-8.4 to -4.2)
Turkmenistan	303 (218-393)	117 (67-178)	68 (38-103)	488 (435 to 563)	-1.5 (-5.4 to 0.4)	36.9 (28.0 to 49.2)	57.6 (51.6 to 61.8)	1.2 (0.6 to 1.7)	-6.7 (-12.0 to 0.5)	-8.1 (-17.6 to 3.5)	3.0 (-6.4 to 14.7)	-6.2 (-7.5 to -4.7)
Uganda	22,750 (17,805-28,122)	1,252 (569-2,424)	21 (10-41)	24,023 (18,882 to 29,559)	3.1 (1.7 to 4.5)	30.9 (23.8 to 38.5)	39.7 (34.5 to 44.2)	3.4 (2.0 to 4.8)	-3.1 (-4.7 to -1.6)	-3.1 (-13.5 to 7.2)	8.0 (-2.4 to 18.4)	-3.1 (-4.7 to -1.6)
Ukraine	1,650 (1,089-2,361)	1,125 (728-1,608)	651 (422-936)	3,425 (2,503 to 4,594)	1.2 (-1.1 to 3.0)	39.7 (35.5 to 44.2)	56.8 (54.4 to 58.6)	5.3 (4.3 to 6.1)	-7.8 (-12.5 to -3.4)	-9.0 (-13.7 to -4.5)	2.1 (-2.5 to 6.7)	-7.0 (-10.2 to -3.9)
United Arab Emirates	68 (48-92)	4 (0-16)	0 (0-1)	72 (53 to 97)	-6.2 (-8.2 to -4.3)	7.0 (-7.9 to 22.1)	23.4 (8.5 to 32.5)	-6.0 (-7.8 to -4.1)	-2.0 (-4.7 to 0.2)	-0.4 (-17.6 to 17.0)	7.6 (-9.6 to 25.0)	-1.9 (-4.1 to 0.1)
United Kingdom	379 (357-404)	10 (8-12)	3 (3-4)	393 (370 to 418)	-3.9 (-4.0 to -3.7)	-1.7 (-2.3 to -1.2)	18.6 (17.2 to 20.0)	-3.8 (-3.9 to -3.6)	-3.2 (-3.8 to -2.6)	-3.1 (-4.0 to -2.2)	4.3 (3.4 to 5.2)	-3.1 (-3.7 to -2.6)
United States	919 (878-960)	28 (23-34)	9 (8-11)	956 (913 to 1,000)	-7.2 (-7.4 to -6.9)	-9.0 (-9.4 to -8.7)	16.1 (14.8 to 17.3)	-7.2 (-7.4 to -7.0)	-2.3 (-2.8 to -1.9)	-3.0 (-3.6 to -2.5)	4.4 (3.9 to 5.0)	-2.3 (-2.7 to -1.9)
Uruguay	52 (48-57)	0 (0-1)	0 (0-0)	53 (48 to 58)	-6.2 (-6.8 to -5.6)	-3.5 (-15.2 to 9.7)	11.2 (0.7 to 18.8)	-6.2 (-6.8 to -5.6)	-2.5 (-3.4 to -1.5)	6.3 (-8.1 to 23.4)	13.7 (-0.7 to 30.9)	-2.4 (-3.4 to -1.4)
Uzbekistan	1,130 (576-1,789)	848 (433-1,248)	490 (250-720)	2,468 (2,111 to 2,868)	-2.0 (-6.4 to 0.6)	39.3 (31.1 to 51.6)	59.8 (56.3 to 62.3)	2.5 (1.6 to 3.3)	-6.9 (-15.5 to 2.8)	-8.5 (-17.1 to -0.8)	2.6 (-6.0 to 10.3)	-6.2 (-8.1 to -4.5)
Vanuatu	34 (27-42)	0 (0-1)	0 (0-0)	34 (27 to 42)	-3.1 (-4.8 to -1.7)	1.2 (-27.9 to 26.4)	29.8 (-2.1 to 41.2)	-3.0 (-4.8 to -1.7)	-4.3 (-6.0 to -2.6)	-5.5 (-41.9 to 29.3)	6.3 (-30.2 to 41.0)	-4.3 (-5.8 to -2.7)
Venezuela	698 (579-844)	25 (12-46)	5 (2-10)	729 (603 to 883)	-6.0 (-6.7 to -5.2)	9.1 (4.0 to 13.7)	30.3 (26.0 to 33.8)	-5.7 (-6.5 to -4.9)	-2.7 (-4.7 to -0.6)	-4.1 (-10.7 to 2.1)	5.5 (-1.1 to 11.7)	-2.7 (-4.7 to -0.6)

	Number of deaths (all ages, both sexes) with 95% uncertainty intervals (2016)				Annualized rate of change of age-standardized rate (%) with 95% uncertainty intervals							
					1990-2006				2006-2016			
	**Drug-susceptible TB**	**MDR TB**	**XDR TB**	**All HIV-negative TB**	**Drug-susceptible TB**	**MDR TB**	**XDR TB**	**All HIV-negative TB**	**Drug-susceptible TB**	**MDR TB**	**XDR TB**	**All HIV-negative TB**
Vietnam	16,612 (13,181-20,148)	1,660 (516-3,687)	400 (125-891)	18,672 (15,880 to 22,214)	-6.3 (-7.9 to -4.8)	6.7 (-1.7 to 17.8)	48.9 (44.9 to 52.2)	-5.9 (-7.4 to -4.3)	-6.7 (-9.3 to -4.5)	-5.1 (-18.3 to 5.3)	6.7 (-6.5 to 17.0)	-6.4 (-8.2 to -4.6)
Virgin Islands, U.S.	1 (1-1)	0 (0-0)	0 (0-0)	1 (1 to 1)	-3.8 (-4.9 to -2.5)	2.2 (-11.5 to 15.8)	16.7 (2.9 to 25.4)	-3.7 (-4.7 to -2.5)	-1.5 (-3.2 to 0.0)	-6.4 (-23.3 to 11.1)	3.2 (-13.7 to 20.7)	-1.6 (-3.2 to -0.2)
Yemen	1,699 (718-2,520)	106 (21-307)	10 (2-29)	1,815 (764 to 2,707)	-6.0 (-7.2 to -4.8)	13.5 (3.3 to 28.5)	41.7 (34.5 to 46.3)	-5.5 (-6.6 to -4.3)	-3.9 (-5.7 to -2.3)	-6.3 (-22.1 to 6.2)	1.8 (-14.1 to 14.3)	-4.1 (-5.4 to -2.6)
Zambia	11,634 (7,897-15,237)	468 (168-1,112)	8 (3-19)	12,110 (8,258 to 15,693)	9.8 (7.1 to 11.9)	25.0 (18.0 to 33.1)	39.6 (34.7 to 43.4)	10.0 (7.2 to 12.1)	-5.1 (-7.7 to -2.6)	-4.9 (-14.9 to 4.4)	6.2 (-3.8 to 15.6)	-5.1 (-7.6 to -2.6)
Zimbabwe	9,010 (6,255-11,365)	716 (171-1,812)	12 (3-31)	9,738 (7,125 to 12,209)	7.6 (4.2 to 11.0)	22.8 (13.0 to 32.6)	41.2 (31.3 to 47.0)	8.0 (4.5 to 11.2)	-6.0 (-9.1 to -2.5)	-3.4 (-14.6 to 8.6)	7.8 (-3.5 to 19.8)	-5.9 (-8.5 to -2.4)

eTable 8. Tuberculosis, drug-susceptible tuberculosis, multidrug-resistant tuberculosis, and extensively drug-resistant tuberculosis incident cases in HIV-positive individuals, and annualized rates of change of age-standardized rates for 195 countries and territories

	Number of incident cases (all ages, both sexes) with 95% uncertainty intervals (2016)				Annualized rate of change of age-standardized rate (%) with 95% uncertainty intervals							
					1990-2006				2006-2016			
	**Drug-susceptible HIV-TB**	**MDR HIV-TB**	**XDR HIV-TB**	**All HIV-positive TB**	**Drug-susceptible HIV-TB**	**MDR HIV-TB**	**XDR HIV-TB**	**All HIV-positive TB**	**Drug-susceptible HIV-TB**	**MDR HIV-TB**	**XDR HIV-TB**	**All HIV-positive TB**
Global	1,364,692 (981,274-1,846,658)	35,815 (23,524-51,741)	1,303 (793-2,019)	1,401,810 (1,005,231 to 1,894,784)	7.9 (7.3 to 8.6)	23.3 (21.6 to 25.4)	27.7 (24.5 to 30.6)	8.1 (7.5 to 8.8)	-4.0 (-4.5 to -3.7)	-4.6 (-6.6 to -2.7)	7.2 (5.4 to 8.7)	-4.0 (-4.5 to -3.7)
Afghanistan	44 (25-77)	4 (1-13)	0 (0-0)	48 (27 to 83)	1.1 (0.0 to 2.1)	32.8 (23.6 to 47.5)	5.4 (-4.2 to 12.2)	1.6 (0.9 to 2.5)	-1.9 (-4.3 to 0.1)	-1.0 (-21.5 to 16.6)	7.2 (-13.3 to 24.8)	-1.8 (-3.2 to -0.8)
Albania	7 (4-13)	0 (0-0)	0 (0-0)	7 (4 to 13)	-0.9 (-1.4 to -0.4)	16.1 (4.8 to 29.5)	6.1 (-6.0 to 13.7)	-0.8 (-1.3 to -0.3)	-4.1 (-4.7 to -3.4)	-5.2 (-24.4 to 14.1)	6.1 (-13.1 to 25.3)	-4.1 (-4.7 to -3.5)
Algeria	136 (72-245)	4 (0-16)	0 (0-1)	140 (74 to 249)	1.3 (0.3 to 2.3)	4.2 (-6.6 to 14.9)	4.4 (-5.2 to 11.4)	1.3 (0.3 to 2.3)	-6.5 (-7.8 to -5.3)	-3.7 (-23.9 to 10.8)	4.5 (-15.7 to 19.0)	-6.4 (-7.6 to -5.3)
American Samoa	1 (0-1)	0 (0-0)	0 (0-0)	1 (0 to 1)	3.2 (2.1 to 4.3)	13.8 (-13.2 to 41.9)	7.3 (-24.9 to 19.2)	3.3 (2.4 to 4.3)	2.5 (1.4 to 3.6)	-0.7 (-38.5 to 32.7)	11.2 (-26.6 to 44.6)	2.4 (1.6 to 3.2)
Andorra	1 (0-1)	0 (0-0)	0 (0-0)	1 (0 to 1)	-6.4 (-7.1 to -5.7)	-6.3 (-9.9 to -3.0)	-15.0 (-19.5 to -11.0)	-6.4 (-7.1 to -5.7)	-3.0 (-3.8 to -2.2)	-3.5 (-8.0 to 1.2)	4.2 (-0.4 to 8.8)	-3.0 (-3.8 to -2.2)
Angola	8,782 (5,186-13,922)	78 (35-161)	1 (0-1)	8,861 (5,241 to 14,055)	5.0 (4.1 to 5.9)	14.4 (10.3 to 18.6)	15.9 (10.7 to 20.1)	5.0 (4.1 to 6.0)	-3.1 (-3.7 to -2.5)	-3.6 (-9.4 to 2.3)	7.6 (1.8 to 13.4)	-3.1 (-3.8 to -2.5)
Antigua and Barbuda	2 (1-2)	0 (0-0)	0 (0-0)	2 (1 to 2)	2.0 (1.3 to 2.8)	7.8 (-7.1 to 20.7)	11.0 (-3.8 to 19.8)	2.0 (1.3 to 2.8)	-3.1 (-3.9 to -2.3)	-6.0 (-23.4 to 11.6)	3.8 (-13.7 to 21.4)	-3.1 (-3.9 to -2.3)
Argentina	3,836 (2,826-4,952)	121 (18-391)	15 (2-49)	3,972 (2,928 to 5,117)	-1.7 (-2.8 to -0.6)	11.9 (2.8 to 25.8)	32.2 (26.1 to 36.8)	-1.5 (-2.6 to -0.4)	-1.1 (-2.3 to 0.0)	-0.8 (-18.7 to 12.3)	6.8 (-11.0 to 19.9)	-1.1 (-1.9 to -0.0)
Armenia	45 (23-76)	6 (1-17)	1 (0-4)	52 (30 to 86)	4.4 (2.4 to 58.8)	37.0 (29.1 to 48.8)	28.2 (22.3 to 32.7)	5.2 (3.4 to 59.7)	3.2 (-0.5 to 5.6)	3.6 (-12.7 to 17.0)	14.9 (-1.5 to 28.3)	3.4 (2.0 to 5.2)
Australia	32 (18-56)	1 (0-2)	0 (0-0)	33 (18 to 57)	-9.3 (-10.6 to -8.1)	2.6 (-7.7 to 13.1)	5.7 (-3.5 to 12.3)	-9.2 (-10.4 to -8.0)	-4.2 (-5.1 to -3.3)	-3.1 (-15.3 to 10.5)	4.5 (-7.6 to 18.1)	-4.1 (-5.0 to -3.3)
Austria	27 (14-47)	1 (0-2)	0 (0-0)	28 (15 to 48)	-5.5 (-6.7 to 52.7)	4.9 (1.7 to 30.4)	12.8 (8.6 to 16.5)	-5.4 (-6.5 to 52.9)	-6.0 (-7.1 to -5.0)	-3.3 (-7.3 to 0.1)	4.4 (0.4 to 7.8)	-5.9 (-7.0 to -4.9)
Azerbaijan	91 (48-158)	20 (8-41)	4 (2-9)	116 (65 to 199)	5.7 (3.0 to 7.3)	45.5 (36.0 to 58.1)	37.7 (29.1 to 43.2)	7.2 (6.3 to 8.1)	-10.8 (-14.4 to -6.3)	-11.7 (-23.6 to 3.1)	-0.4 (-12.3 to 14.4)	-10.7 (-12.0 to -9.4)
Bahrain	10 (5-19)	0 (0-1)	0 (0-0)	11 (5 to 19)	0.7 (-0.3 to 1.8)	23.0 (12.0 to 37.6)	6.3 (-4.1 to 13.7)	0.8 (-0.2 to 1.9)	-3.9 (-5.5 to -2.5)	0.3 (-16.5 to 20.4)	8.5 (-8.3 to 28.6)	-3.8 (-5.4 to -2.4)

	Number of incident cases (all ages, both sexes) with 95% uncertainty intervals (2016)				Annualized rate of change of age-standardized rate (%) with 95% uncertainty intervals							
					1990-2006				2006-2016			
	**Drug-susceptible HIV-TB**	**MDR HIV-TB**	**XDR HIV-TB**	**All HIV-positive TB**	**Drug-susceptible HIV-TB**	**MDR HIV-TB**	**XDR HIV-TB**	**All HIV-positive TB**	**Drug-susceptible HIV-TB**	**MDR HIV-TB**	**XDR HIV-TB**	**All HIV-positive TB**
Bangladesh	2,103 (1,135-3,689)	58 (30-104)	1 (1-3)	2,162 (1,168 to 3,794)	56.2 (52.1 to 59.9)	33.7 (29.3 to 37.6)	4.5 (0.0 to 8.4)	56.4 (52.3 to 60.1)	4.4 (3.5 to 5.6)	4.5 (1.9 to 7.3)	14.2 (11.6 to 17.0)	4.5 (3.5 to 5.6)
Barbados	4 (3-7)	0 (0-0)	0 (0-0)	4 (3 to 7)	1.5 (0.8 to 2.1)	3.2 (-9.6 to 16.2)	-7.1 (-21.7 to 1.7)	1.5 (0.8 to 2.1)	-3.3 (-4.1 to -2.6)	-4.7 (-22.2 to 13.7)	5.1 (-12.4 to 23.5)	-3.3 (-4.1 to -2.6)
Belarus	73 (41-123)	52 (30-87)	11 (7-19)	136 (80 to 222)	2.6 (0.4 to 4.1)	50.3 (46.0 to 55.1)	37.6 (32.7 to 41.4)	5.6 (4.6 to 6.4)	-3.6 (-6.1 to -0.4)	-1.3 (-4.6 to 3.3)	10.0 (6.6 to 14.6)	-2.1 (-2.8 to -0.8)
Belgium	52 (28-87)	1 (1-2)	0 (0-0)	53 (29 to 89)	-5.3 (-6.4 to -4.4)	0.8 (-2.1 to 3.8)	12.7 (8.4 to 16.3)	-5.2 (-6.3 to -4.3)	-4.0 (-4.9 to -2.9)	-4.5 (-8.4 to -0.9)	3.2 (-0.7 to 6.8)	-4.0 (-4.9 to -2.9)
Belize	27 (17-41)	0 (0-1)	0 (0-0)	27 (17 to 41)	3.1 (2.1 to 4.1)	9.4 (-5.3 to 22.7)	21.1 (5.9 to 29.8)	3.2 (2.1 to 4.1)	-3.3 (-4.0 to -2.3)	-7.1 (-25.6 to 11.6)	2.7 (-15.8 to 21.4)	-3.3 (-4.0 to -2.4)
Benin	1,373 (821-2,188)	16 (5-41)	0 (0-0)	1,389 (829 to 2,207)	6.0 (5.0 to 7.0)	17.5 (10.7 to 24.4)	11.7 (4.5 to 17.2)	6.1 (5.1 to 7.1)	-6.7 (-7.7 to -5.6)	-5.2 (-15.9 to 5.7)	6.0 (-4.8 to 16.9)	-6.6 (-7.7 to -5.6)
Bermuda	1 (0-1)	0 (0-0)	0 (0-0)	1 (0 to 1)	-1.0 (-1.5 to -0.5)	0.2 (-11.8 to 13.4)	-9.4 (-23.0 to -0.7)	-1.0 (-1.5 to -0.5)	-3.3 (-3.9 to -2.6)	-4.6 (-22.2 to 13.0)	5.2 (-12.4 to 22.8)	-3.3 (-3.9 to -2.6)
Bhutan	8 (4-13)	0 (0-0)	0 (0-0)	8 (5 to 13)	2.0 (1.1 to 2.9)	18.8 (16.8 to 20.8)	6.1 (2.4 to 9.8)	2.2 (1.3 to 3.1)	-0.1 (-0.9 to 0.6)	0.3 (-2.4 to 2.9)	10.0 (7.3 to 12.6)	-0.1 (-0.8 to 0.7)
Bolivia	282 (169-463)	10 (2-29)	1 (0-2)	292 (174 to 479)	-2.3 (-3.0 to -1.6)	10.2 (1.3 to 19.7)	25.5 (15.6 to 32.5)	-2.0 (-2.6 to -1.4)	-6.0 (-6.9 to -5.2)	-7.3 (-19.8 to 4.4)	2.5 (-10.0 to 14.2)	-6.1 (-6.8 to -5.4)
Bosnia and Herzegovina	8 (4-14)	0 (0-0)	0 (0-0)	8 (4 to 14)	-1.6 (-2.0 to -1.3)	3.5 (-5.3 to 17.4)	-2.5 (-8.7 to 2.6)	-1.6 (-2.0 to -1.3)	-2.7 (-3.2 to -2.1)	-4.9 (-18.7 to 6.2)	6.3 (-7.4 to 17.5)	-2.7 (-3.2 to -2.1)
Botswana	11,103 (7,980-14,712)	240 (71-561)	2 (0-4)	11,344 (8,141 to 15,105)	8.9 (7.3 to 10.8)	21.1 (14.4 to 29.5)	38.1 (34.2 to 41.2)	9.0 (7.4 to 10.8)	-9.1 (-10.6 to -7.8)	-6.1 (-17.8 to 4.0)	5.0 (-6.7 to 15.2)	-9.0 (-10.5 to -7.8)
Brazil	13,193 (8,425-19,637)	244 (152-377)	19 (12-30)	13,457 (8,591 to 20,036)	0.3 (-0.3 to 0.9)	25.2 (24.4 to 26.1)	23.4 (20.5 to 26.1)	0.4 (-0.1 to 1.0)	-1.9 (-2.2 to -1.7)	-2.9 (-3.6 to -2.1)	6.9 (6.1 to 7.7)	-1.9 (-2.2 to -1.7)
Brunei	2 (1-4)	0 (0-0)	0 (0-0)	2 (1 to 4)	-0.9 (-1.4 to -0.4)	4.2 (0.8 to 7.6)	-13.7 (-18.5 to -9.3)	-0.9 (-1.4 to -0.4)	-1.8 (-2.4 to -1.1)	4.5 (-0.6 to 9.5)	12.2 (7.1 to 17.2)	-1.8 (-2.4 to -1.1)
Bulgaria	4 (2-7)	0 (0-0)	0 (0-0)	4 (2 to 7)	4.0 (2.8 to 43.0)	26.9 (17.0 to 41.3)	5.1 (-4.3 to 12.0)	4.2 (3.1 to 43.2)	-6.5 (-7.5 to -5.5)	-7.0 (-24.8 to 11.9)	4.2 (-13.5 to 23.2)	-6.5 (-7.2 to -5.8)
Burkina Faso	4,002 (2,415-6,291)	77 (20-222)	0 (0-1)	4,080 (2,472 to 6,411)	-1.0 (-1.6 to -0.5)	12.8 (5.2 to 20.4)	21.7 (13.0 to 28.0)	-0.9 (-1.4 to -0.4)	-7.8 (-9.2 to -6.5)	-9.1 (-19.0 to 1.7)	2.1 (-7.8 to 12.8)	-7.8 (-9.2 to -6.6)
Burundi	6,790 (4,169-10,635)	168 (41-465)	1 (0-3)	6,960 (4,261 to 10,883)	3.3 (2.4 to 4.1)	23.9 (15.6 to 31.9)	27.3 (18.6 to 33.3)	3.4 (2.6 to 4.2)	-7.3 (-8.7 to -6.0)	-5.7 (-17.5 to 5.3)	5.5 (-6.4 to 16.4)	-7.3 (-8.6 to -5.9)

	Number of incident cases (all ages, both sexes) with 95% uncertainty intervals (2016)				Annualized rate of change of age-standardized rate (%) with 95% uncertainty intervals							
					1990-2006				2006-2016			
	**Drug-susceptible HIV-TB**	**MDR HIV-TB**	**XDR HIV-TB**	**All HIV-positive TB**	**Drug-susceptible HIV-TB**	**MDR HIV-TB**	**XDR HIV-TB**	**All HIV-positive TB**	**Drug-susceptible HIV-TB**	**MDR HIV-TB**	**XDR HIV-TB**	**All HIV-positive TB**
Cambodia	2,061 (1,232-3,263)	37 (6-137)	3 (1-12)	2,101 (1,255 to 3,318)	22.7 (19.5 to 26.0)	44.7 (34.3 to 59.3)	35.3 (28.3 to 40.7)	22.8 (19.6 to 26.1)	-11.9 (-13.2 to -10.7)	-13.0 (-31.8 to 2.6)	-1.1 (-19.9 to 14.6)	-11.9 (-13.2 to -10.7)
Cameroon	16,602 (11,155-23,026)	302 (81-792)	2 (1-5)	16,906 (11,349 to 23,418)	7.4 (6.2 to 8.6)	20.6 (13.1 to 28.9)	24.8 (17.1 to 30.7)	7.5 (6.3 to 8.7)	-3.3 (-4.1 to -2.4)	-4.2 (-15.1 to 6.7)	7.0 (-4.0 to 17.8)	-3.3 (-4.1 to -2.5)
Canada	166 (100-263)	2 (1-3)	0 (0-0)	168 (101 to 266)	-5.6 (-6.4 to -4.8)	-3.6 (-5.1 to -2.0)	8.3 (4.9 to 11.0)	-5.6 (-6.3 to -4.8)	-3.4 (-4.1 to -2.8)	-3.8 (-5.7 to -2.1)	3.8 (2.0 to 5.6)	-3.4 (-4.1 to -2.8)
Cape Verde	32 (19-51)	1 (0-2)	0 (0-0)	32 (19 to 52)	0.8 (0.2 to 1.6)	14.2 (6.3 to 22.4)	9.3 (0.9 to 15.0)	1.0 (0.3 to 1.6)	-4.6 (-5.5 to -3.8)	-5.6 (-16.5 to 5.5)	5.6 (-5.4 to 16.7)	-4.7 (-5.5 to -3.9)
Central African Republic	15,430 (10,037-22,229)	102 (49-187)	1 (0-1)	15,532 (10,077 to 22,397)	4.9 (4.0 to 5.7)	12.7 (8.6 to 17.2)	27.5 (22.9 to 31.1)	4.9 (4.1 to 5.8)	-3.5 (-4.3 to -2.7)	-4.3 (-9.8 to 1.3)	6.8 (1.3 to 12.4)	-3.5 (-4.3 to -2.7)
Chad	4,154 (2,520-6,485)	81 (19-218)	1 (0-1)	4,235 (2,549 to 6,571)	6.7 (5.8 to 7.7)	20.4 (12.0 to 28.2)	21.3 (12.4 to 27.9)	6.8 (5.9 to 7.8)	-4.6 (-5.4 to -3.7)	-5.7 (-16.6 to 5.4)	5.4 (-5.4 to 16.5)	-4.6 (-5.4 to -3.8)
Chile	775 (513-1,112)	8 (3-16)	1 (0-2)	784 (520 to 1,125)	-2.3 (-3.1 to -1.4)	6.1 (-5.4 to 18.8)	20.1 (12.0 to 26.1)	-2.3 (-3.1 to -1.4)	-1.5 (-2.1 to -0.8)	-1.2 (-13.0 to 11.6)	6.5 (-5.4 to 19.3)	-1.5 (-2.1 to -0.9)
China	41,440 (25,289-65,010)	2,221 (1,287-3,653)	203 (117-333)	43,864 (26,753 to 68,788)	7.0 (5.4 to 8.8)	15.9 (14.0 to 18.1)	22.9 (19.4 to 26.2)	7.3 (5.8 to 9.1)	2.0 (1.4 to 2.6)	-0.1 (-1.9 to 1.7)	11.8 (10.0 to 13.6)	1.9 (1.4 to 2.5)
Colombia	1,103 (666-1,741)	34 (14-71)	3 (1-6)	1,140 (693 to 1,787)	2.6 (1.7 to 3.6)	21.0 (16.9 to 25.6)	21.2 (16.7 to 25.1)	2.9 (1.9 to 3.9)	-2.5 (-3.2 to -1.9)	-4.2 (-10.3 to 1.3)	5.6 (-0.5 to 11.1)	-2.6 (-3.2 to -2.0)
Comoros	37 (21-61)	1 (0-3)	0 (0-0)	37 (22 to 62)	13.4 (11.4 to 15.7)	34.3 (25.8 to 42.9)	5.2 (-4.1 to 11.7)	13.6 (11.5 to 15.8)	0.7 (-0.2 to 2.0)	1.8 (-9.9 to 12.7)	12.9 (1.2 to 23.8)	0.8 (-0.2 to 2.0)
Congo (Brazzaville)	4,352 (2,705-6,586)	39 (17-78)	0 (0-1)	4,391 (2,729 to 6,646)	0.2 (-0.2 to 0.7)	9.7 (5.7 to 13.7)	22.5 (17.6 to 26.5)	0.3 (-0.2 to 0.7)	-5.0 (-5.6 to -4.3)	-5.3 (-11.1 to 0.6)	5.8 (0.1 to 11.7)	-5.0 (-5.6 to -4.3)
Costa Rica	42 (26-68)	1 (0-2)	0 (0-0)	43 (26 to 69)	-0.4 (-1.2 to 0.3)	22.1 (17.2 to 26.9)	11.4 (5.9 to 15.9)	-0.3 (-1.1 to 0.4)	-2.9 (-3.5 to -2.2)	-3.7 (-9.6 to 2.7)	6.1 (0.2 to 12.4)	-2.9 (-3.6 to -2.2)
Cote d'Ivoire	10,087 (6,261-15,350)	303 (88-713)	2 (1-5)	10,392 (6,487 to 15,823)	3.4 (2.6 to 4.1)	11.4 (4.9 to 18.1)	26.2 (20.7 to 30.4)	3.5 (2.7 to 4.3)	-5.5 (-6.6 to -4.6)	-6.1 (-16.7 to 3.1)	5.1 (-5.5 to 14.3)	-5.6 (-6.5 to -4.7)
Croatia	5 (3-8)	0 (0-0)	0 (0-0)	5 (3 to 8)	-3.9 (-4.6 to -3.3)	-0.9 (-10.9 to 13.5)	-3.5 (-10.5 to 1.6)	-3.9 (-4.6 to -3.3)	-5.6 (-6.3 to -4.9)	-11.2 (-28.9 to 2.3)	0.1 (-17.6 to 13.6)	-5.6 (-6.3 to -4.9)
Cuba	49 (29-80)	1 (0-2)	0 (0-0)	49 (30 to 81)	-1.4 (-2.7 to -0.4)	0.2 (-8.6 to 9.7)	-1.1 (-8.0 to 3.9)	-1.4 (-2.6 to -0.3)	3.8 (2.5 to 5.5)	5.4 (-8.3 to 18.4)	15.2 (1.5 to 28.2)	3.8 (2.5 to 5.5)

	Number of incident cases (all ages, both sexes) with 95% uncertainty intervals (2016)				Annualized rate of change of age-standardized rate (%) with 95% uncertainty intervals							
					1990-2006				2006-2016			
	Drug-susceptible HIV-TB	MDR HIV-TB	XDR HIV-TB	All HIV-positive TB	Drug-susceptible HIV-TB	MDR HIV-TB	XDR HIV-TB	All HIV-positive TB	Drug-susceptibl e HIV-TB	MDR HIV-TB	XDR HIV-TB	All HIV-positive TB
Cyprus	4 (2-8)	0 (0-0)	0 (0-0)	4 (2 to 8)	1.7 (1.0 to 2.4)	14.9 (11.7 to 18.1)	5.3 (0.4 to 9.4)	1.7 (1.1 to 2.4)	-2.3 (-3.1 to -1.4)	-9.0 (-13.5 to -4.2)	-1.4 (-5.8 to 3.4)	-2.4 (-3.2 to -1.4)
Czech Republic	20 (11-34)	0 (0-1)	0 (0-0)	20 (11 to 35)	-3.3 (-4.2 to -2.6)	2.5 (-5.9 to 14.3)	6.1 (0.5 to 10.4)	-3.2 (-4.1 to -2.5)	0.5 (-0.4 to 2.0)	-0.3 (-8.7 to 7.9)	11.0 (2.5 to 19.1)	0.5 (-0.4 to 2.0)
Democratic Republic of the Congo	33,757 (20,096-53,627)	304 (134-604)	2 (1-4)	34,063 (20,289 to 54,130)	0.9 (0.5 to 1.3)	10.5 (6.2 to 14.4)	19.1 (13.9 to 23.4)	0.9 (0.5 to 1.3)	-6.0 (-6.9 to -5.2)	-6.5 (-12.2 to -0.6)	4.7 (-1.0 to 10.5)	-6.0 (-6.9 to -5.2)
Denmark	13 (7-22)	0 (0-0)	0 (0-0)	13 (7 to 22)	-6.2 (-7.3 to -5.4)	1.5 (-1.5 to 4.5)	-1.2 (-5.7 to 2.8)	-6.2 (-7.2 to -5.4)	-5.3 (-6.4 to -4.3)	0.2 (-4.4 to 4.6)	7.9 (3.2 to 12.3)	-5.3 (-6.4 to -4.3)
Djibouti	617 (371-966)	32 (14-68)	0 (0-0)	649 (393 to 1,019)	16.2 (14.4 to 18.0)	49.2 (41.7 to 57.8)	28.2 (20.1 to 34.0)	16.5 (14.7 to 18.3)	-1.8 (-2.7 to -0.7)	-1.5 (-11.0 to 9.8)	9.6 (0.2 to 21.0)	-1.8 (-2.5 to -0.9)
Dominica	4 (2-6)	0 (0-0)	0 (0-0)	4 (2 to 6)	2.9 (2.2 to 3.6)	9.0 (-5.7 to 22.1)	17.3 (2.2 to 26.1)	3.0 (2.2 to 3.6)	-1.7 (-2.4 to -1.0)	-4.9 (-23.7 to 13.0)	4.9 (-14.0 to 22.8)	-1.7 (-2.4 to -1.1)
Dominican Republic	977 (606-1,492)	6 (1-23)	0 (0-2)	983 (610 to 1,499)	5.0 (3.8 to 6.4)	10.8 (-2.5 to 23.4)	23.0 (8.1 to 31.3)	5.1 (3.9 to 6.4)	-4.8 (-5.6 to -4.1)	-8.2 (-25.2 to 10.5)	1.6 (-15.4 to 20.3)	-4.9 (-5.5 to -4.2)
Ecuador	932 (596-1,430)	62 (16-167)	5 (1-13)	999 (641 to 1,510)	0.8 (-0.2 to 2.1)	20.6 (13.0 to 30.2)	29.8 (24.5 to 33.9)	1.2 (0.3 to 2.4)	0.0 (-1.1 to 1.0)	-0.4 (-11.8 to 8.2)	9.4 (-2.0 to 18.0)	0.0 (-0.6 to 0.8)
Egypt	103 (53-186)	6 (1-18)	0 (0-1)	110 (56 to 197)	0.0 (-0.8 to 0.9)	19.6 (9.3 to 33.4)	-0.2 (-7.1 to 5.1)	0.3 (-0.5 to 1.1)	-3.1 (-4.5 to -2.0)	1.1 (-15.6 to 14.7)	9.3 (-7.4 to 22.9)	-2.8 (-3.7 to -2.0)
El Salvador	112 (67-181)	2 (1-4)	0 (0-0)	114 (69 to 184)	-0.6 (-1.4 to 0.4)	15.3 (10.2 to 20.2)	19.2 (13.4 to 23.8)	-0.4 (-1.3 to 0.5)	-7.2 (-8.3 to -6.2)	-8.7 (-15.8 to -1.2)	1.1 (-6.0 to 8.6)	-7.2 (-8.3 to -6.2)
Equatorial Guinea	886 (551-1,328)	8 (4-14)	0 (0-0)	894 (554 to 1,339)	6.3 (5.1 to 7.6)	15.5 (11.6 to 20.1)	21.7 (16.8 to 25.6)	6.4 (5.2 to 7.6)	-3.2 (-3.9 to -2.5)	-3.6 (-9.1 to 2.0)	7.5 (2.1 to 13.1)	-3.2 (-3.9 to -2.5)
Eritrea	1,053 (614-1,687)	27 (7-76)	0 (0-0)	1,081 (643 to 1,752)	6.2 (5.3 to 7.2)	26.7 (18.2 to 35.2)	17.8 (9.2 to 24.1)	6.4 (5.5 to 7.3)	-3.2 (-4.3 to -2.0)	-2.1 (-13.1 to 8.5)	9.0 (-2.0 to 19.7)	-3.2 (-4.3 to -2.1)
Estonia	20 (12-32)	5 (3-8)	1 (1-2)	27 (16 to 42)	10.6 (8.6 to 12.8)	16.4 (13.2 to 20.6)	37.6 (33.8 to 40.8)	11.4 (9.5 to 13.7)	-4.3 (-5.5 to -2.9)	-4.1 (-7.0 to -1.2)	7.2 (4.2 to 10.1)	-4.0 (-4.7 to -2.8)
Ethiopia	27,409 (16,585-43,954)	695 (178-1,777)	4 (1-11)	28,108 (17,103 to 44,817)	3.4 (2.6 to 4.3)	26.5 (19.7 to 34.1)	24.8 (19.6 to 29.3)	3.6 (2.8 to 4.5)	-10.9 (-12.4 to -9.6)	-9.5 (-20.7 to -0.1)	1.7 (-9.5 to 11.0)	-10.9 (-12.3 to -9.6)
Federated States of Micronesia	12 (7-19)	0 (0-1)	0 (0-0)	12 (7 to 19)	2.1 (1.0 to 3.2)	12.6 (-13.4 to 39.9)	19.6 (-14.0 to 32.4)	2.2 (1.3 to 3.2)	12.7 (11.0 to 14.5)	9.0 (-28.6 to 43.8)	20.9 (-16.7 to 55.7)	12.7 (11.1 to 14.2)
Fiji	10 (6-17)	0 (0-0)	0 (0-0)	10 (6 to 17)	6.2 (5.2 to 7.3)	10.2 (-15.8 to 37.2)	-1.6 (-35.8 to 10.4)	6.2 (5.2 to 7.3)	-2.2 (-2.9 to -1.7)	-4.5 (-41.5 to 34.8)	7.4 (-29.6 to 46.7)	-2.2 (-2.9 to -1.7)

	Number of incident cases (all ages, both sexes) with 95% uncertainty intervals (2016)				Annualized rate of change of age-standardized rate (%) with 95% uncertainty intervals							
					1990-2006				2006-2016			
	**Drug-susceptible HIV-TB**	**MDR HIV-TB**	**XDR HIV-TB**	**All HIV-positive TB**	**Drug-susceptible HIV-TB**	**MDR HIV-TB**	**XDR HIV-TB**	**All HIV-positive TB**	**Drug-susceptible HIV-TB**	**MDR HIV-TB**	**XDR HIV-TB**	**All HIV-positive TB**
Finland	6 (3-11)	0 (0-0)	0 (0-0)	7 (3 to 12)	-5.2 (-5.9 to -4.5)	8.1 (4.9 to 11.2)	-0.6 (-5.2 to 3.6)	-5.1 (-5.9 to -4.4)	-5.4 (-6.4 to -4.5)	2.9 (-1.1 to 6.8)	10.6 (6.5 to 14.5)	-5.3 (-6.3 to -4.4)
France	375 (209-627)	7 (3-12)	1 (0-1)	382 (213 to 638)	-10.3 (-11.6 to -9.2)	-3.8 (-6.5 to -1.0)	13.9 (9.9 to 17.0)	-10.2 (-11.5 to -9.1)	-6.4 (-7.5 to -5.2)	-5.8 (-9.1 to -2.5)	1.9 (-1.4 to 5.2)	-6.4 (-7.5 to -5.2)
Gabon	1,677 (1,066-2,437)	14 (7-27)	0 (0-0)	1,691 (1,073 to 2,459)	6.7 (5.7 to 7.8)	15.9 (11.7 to 20.0)	23.7 (19.2 to 27.6)	6.7 (5.7 to 7.9)	-7.3 (-8.1 to -6.5)	-7.7 (-12.9 to -1.9)	3.4 (-1.8 to 9.3)	-7.3 (-8.1 to -6.5)
Georgia	85 (50-144)	18 (9-31)	4 (2-7)	107 (62 to 178)	2.5 (1.8 to 3.3)	31.5 (23.7 to 43.7)	26.7 (22.1 to 30.9)	3.1 (2.4 to 3.9)	6.1 (4.4 to 8.0)	13.8 (9.3 to 18.7)	25.1 (20.5 to 29.9)	7.3 (5.8 to 9.0)
Germany	247 (132-421)	7 (3-12)	1 (0-2)	254 (136 to 434)	-10.1 (-11.3 to -9.0)	-4.1 (-6.8 to -1.2)	9.3 (5.4 to 12.9)	-10.0 (-11.2 to -8.9)	-2.7 (-3.8 to -1.6)	-0.4 (-3.6 to 2.5)	7.2 (4.1 to 10.2)	-2.6 (-3.7 to -1.5)
Ghana	11,767 (7,302-18,198)	213 (55-568)	1 (0-4)	11,982 (7,376 to 18,519)	2.2 (1.4 to 3.1)	15.1 (7.6 to 23.4)	23.0 (14.6 to 28.7)	2.3 (1.5 to 3.2)	-6.2 (-7.3 to -5.2)	-7.3 (-18.0 to 4.2)	3.8 (-6.8 to 15.3)	-6.2 (-7.3 to -5.2)
Greece	29 (15-50)	1 (1-2)	0 (0-0)	30 (16 to 52)	-7.6 (-8.6 to -6.8)	16.3 (13.4 to 19.3)	12.0 (7.2 to 16.2)	-7.4 (-8.4 to -6.5)	-0.9 (-1.8 to -0.0)	-1.9 (-6.4 to 2.5)	5.8 (1.2 to 10.2)	-0.9 (-1.8 to -0.1)
Greenland	3 (2-4)	0 (0-0)	0 (0-0)	3 (2 to 4)	-6.4 (-7.1 to -5.8)	-4.1 (-5.6 to -2.7)	25.4 (22.2 to 28.3)	-6.4 (-7.1 to -5.8)	-5.7 (-6.6 to -4.9)	-5.9 (-8.0 to -4.0)	1.8 (-0.4 to 3.7)	-5.7 (-6.6 to -4.9)
Grenada	1 (1-2)	0 (0-0)	0 (0-0)	1 (1 to 2)	3.5 (2.7 to 4.3)	9.6 (-3.6 to 23.4)	10.0 (-4.7 to 19.4)	3.5 (2.8 to 4.3)	-3.7 (-4.5 to -3.0)	-7.3 (-26.2 to 9.8)	2.5 (-16.4 to 19.6)	-3.8 (-4.5 to -3.0)
Guam	4 (3-7)	0 (0-0)	0 (0-0)	4 (3 to 7)	3.0 (2.0 to 4.0)	17.5 (2.7 to 46.5)	16.9 (5.0 to 24.5)	3.2 (2.3 to 4.1)	4.7 (3.6 to 6.2)	-3.2 (-39.1 to 21.4)	8.7 (-27.2 to 33.3)	4.6 (3.8 to 5.5)
Guatemala	569 (356-869)	18 (8-35)	1 (1-3)	588 (369 to 895)	-2.9 (-3.5 to -2.3)	17.5 (13.4 to 22.4)	27.0 (22.5 to 30.8)	-2.7 (-3.3 to -2.1)	-5.3 (-6.5 to -4.3)	-6.5 (-13.1 to -0.6)	3.3 (-3.3 to 9.2)	-5.4 (-6.5 to -4.3)
Guinea	5,155 (3,168-7,891)	96 (25-253)	1 (0-2)	5,251 (3,216 to 8,023)	8.7 (7.6 to 9.9)	21.8 (13.7 to 29.8)	20.2 (12.3 to 25.9)	8.8 (7.7 to 10.0)	-1.0 (-1.8 to -0.3)	-2.1 (-13.8 to 9.0)	9.0 (-2.7 to 20.1)	-1.1 (-1.8 to -0.3)
Guinea-Bissau	2,405 (1,648-3,324)	43 (12-111)	0 (0-1)	2,448 (1,676 to 3,378)	9.1 (7.7 to 10.6)	22.1 (14.1 to 30.5)	26.7 (18.7 to 32.6)	9.2 (7.8 to 10.6)	-0.8 (-1.7 to 0.0)	-2.0 (-12.6 to 8.2)	9.1 (-1.4 to 19.3)	-0.8 (-1.6 to -0.0)
Guyana	93 (60-136)	1 (0-2)	0 (0-0)	94 (60 to 137)	6.8 (5.8 to 7.8)	12.3 (-1.0 to 26.2)	25.4 (11.7 to 34.3)	6.8 (5.9 to 7.8)	-5.3 (-6.3 to -4.3)	-8.7 (-25.9 to 9.7)	1.1 (-16.1 to 19.5)	-5.3 (-6.3 to -4.3)
Haiti	1,588 (955-2,483)	10 (1-43)	1 (0-3)	1,599 (968 to 2,487)	2.8 (2.0 to 3.6)	8.3 (-4.4 to 20.9)	29.0 (15.5 to 37.7)	2.8 (2.0 to 3.6)	-8.7 (-9.8 to -7.7)	-11.8 (-29.7 to 6.2)	-2.0 (-20.0 to 16.0)	-8.7 (-9.8 to -7.8)
Honduras	333 (201-530)	7 (3-13)	1 (0-1)	340 (205 to 539)	0.4 (-0.1 to 1.0)	21.3 (16.9 to 25.8)	23.4 (18.5 to 27.4)	0.6 (0.0 to 1.1)	-3.8 (-4.4 to -3.1)	-4.4 (-10.2 to 1.4)	5.4 (-0.4 to 11.2)	-3.8 (-4.4 to -3.1)

	Number of incident cases (all ages, both sexes) with 95% uncertainty intervals (2016)				Annualized rate of change of age-standardized rate (%) with 95% uncertainty intervals							
					1990-2006				2006-2016			
	**Drug-susceptible HIV-TB**	**MDR HIV-TB**	**XDR HIV-TB**	**All HIV-positive TB**	**Drug-susceptible HIV-TB**	**MDR HIV-TB**	**XDR HIV-TB**	**All HIV-positive TB**	**Drug-susceptible HIV-TB**	**MDR HIV-TB**	**XDR HIV-TB**	**All HIV-positive TB**
Hungary	15 (9-26)	0 (0-2)	0 (0-0)	16 (9 to 27)	-6.7 (-7.6 to -6.0)	16.6 (6.3 to 30.2)	13.7 (5.0 to 20.0)	-6.5 (-7.2 to -5.8)	-9.6 (-10.9 to -8.5)	-11.8 (-31.9 to 6.3)	-0.6 (-20.6 to 17.6)	-9.7 (-10.6 to -8.9)
Iceland	1 (1-2)	0 (0-0)	0 (0-0)	1 (1 to 2)	-5.8 (-6.6 to -5.1)	-3.0 (-6.2 to 0.6)	-11.4 (-16.2 to -7.2)	-5.8 (-6.6 to -5.1)	-5.2 (-6.1 to -4.3)	-11.3 (-16.2 to -6.6)	-3.6 (-8.5 to 1.1)	-5.2 (-6.1 to -4.3)
India	89,967 (54,381-142,767)	4,441 (2,613-7,311)	109 (64-179)	94,517 (57,094 to 150,466)	8.3 (7.0 to 9.8)	35.5 (34.1 to 37.1)	25.7 (22.5 to 28.7)	8.6 (7.3 to 10.1)	-7.3 (-8.3 to -6.5)	-6.9 (-8.1 to -5.8)	2.8 (1.6 to 3.9)	-7.3 (-8.2 to -6.4)
Indonesia	253,317 (184,694-337,120)	1,479 (768-2,779)	135 (70-253)	254,932 (185,650 to 339,199)	85.5 (83.1 to 87.7)	58.0 (53.6 to 62.6)	35.6 (31.2 to 40.1)	85.6 (83.2 to 87.8)	0.5 (-0.3 to 1.4)	-6.9 (-13.4 to -0.6)	5.0 (-1.5 to 11.4)	0.4 (-0.3 to 1.3)
Iran	1,976 (1,163-3,185)	25 (7-63)	1 (0-2)	2,001 (1,178 to 3,225)	7.6 (6.4 to 9.2)	30.8 (18.3 to 46.4)	13.3 (0.7 to 21.2)	7.7 (6.4 to 9.3)	-5.6 (-6.4 to -4.8)	-6.9 (-22.5 to 14.0)	1.3 (-14.3 to 22.2)	-5.6 (-6.4 to -4.9)
Iraq	5 (2-9)	0 (0-0)	0 (0-0)	5 (2 to 9)	4.0 (2.8 to 5.2)	29.1 (17.1 to 43.6)	-18.1 (-30.8 to -10.2)	4.1 (3.0 to 5.3)	-1.8 (-3.1 to -0.3)	-1.9 (-18.7 to 18.1)	6.3 (-10.5 to 26.3)	-1.8 (-3.0 to -0.4)
Ireland	29 (15-49)	0 (0-1)	0 (0-0)	29 (16 to 50)	-5.5 (-6.4 to -4.7)	1.8 (-1.3 to 5.2)	10.2 (5.4 to 13.9)	-5.5 (-6.3 to -4.6)	-3.3 (-4.2 to -2.3)	-1.8 (-6.1 to 2.3)	5.8 (1.6 to 10.0)	-3.2 (-4.2 to -2.3)
Israel	15 (8-26)	1 (0-2)	0 (0-0)	16 (9 to 27)	-5.6 (-6.5 to -4.8)	-0.5 (-3.6 to 2.6)	13.7 (9.7 to 17.3)	-5.4 (-6.3 to -4.6)	-3.4 (-4.3 to -2.4)	-3.7 (-7.1 to -0.2)	4.0 (0.5 to 7.5)	-3.4 (-4.3 to -2.4)
Italy	102 (57-170)	3 (2-6)	0 (0-1)	105 (60 to 176)	-8.3 (-9.2 to -7.4)	-2.8 (-5.4 to -0.2)	10.2 (6.4 to 13.5)	-8.1 (-9.0 to -7.2)	-5.7 (-6.6 to -4.9)	-7.0 (-10.5 to -3.7)	0.7 (-2.8 to 3.9)	-5.8 (-6.6 to -5.0)
Jamaica	53 (33-81)	1 (0-4)	0 (0-0)	54 (34 to 82)	5.4 (4.1 to 6.8)	30.1 (18.3 to 44.3)	21.2 (8.4 to 29.2)	5.5 (4.4 to 6.9)	-7.6 (-9.1 to -6.3)	-9.1 (-27.1 to 9.9)	0.6 (-17.3 to 19.7)	-7.6 (-9.0 to -6.5)
Japan	204 (110-363)	2 (1-4)	0 (0-1)	207 (111 to 367)	-3.2 (-3.7 to -2.8)	3.0 (2.0 to 3.9)	2.1 (-2.0 to 5.9)	-3.2 (-3.6 to -2.7)	-2.9 (-3.7 to -2.1)	-4.2 (-5.8 to -2.8)	3.5 (1.9 to 4.9)	-2.9 (-3.8 to -2.1)
Jordan	3 (1-5)	0 (0-1)	0 (0-0)	3 (1 to 6)	2.0 (0.9 to 3.2)	25.3 (15.8 to 40.6)	-2.7 (-10.1 to 2.8)	2.5 (1.4 to 3.7)	-3.6 (-6.3 to -2.1)	-2.1 (-21.0 to 12.2)	6.1 (-12.8 to 20.4)	-3.5 (-4.7 to -2.3)
Kazakhstan	123 (71-206)	43 (23-75)	9 (5-16)	176 (103 to 293)	3.0 (1.3 to 4.4)	31.9 (24.0 to 43.3)	38.3 (33.6 to 42.4)	5.3 (4.4 to 6.1)	-9.0 (-11.3 to -6.3)	-10.4 (-15.5 to -4.6)	0.8 (-4.2 to 6.6)	-9.1 (-9.9 to -8.3)
Kenya	32,808 (23,297-43,765)	431 (276-631)	3 (2-4)	33,241 (23,564 to 44,378)	6.3 (5.4 to 7.2)	31.0 (28.4 to 33.7)	22.5 (20.0 to 24.5)	6.3 (5.4 to 7.3)	-4.7 (-5.7 to -3.7)	-3.9 (-6.9 to -0.7)	7.3 (4.2 to 10.5)	-4.7 (-5.7 to -3.7)
Kiribati	2 (1-4)	0 (0-0)	0 (0-0)	2 (1 to 4)	1.9 (0.9 to 2.7)	11.7 (-12.7 to 41.0)	17.5 (-15.8 to 30.7)	2.1 (1.5 to 2.7)	-4.5 (-5.7 to -3.1)	-6.2 (-45.3 to 29.9)	5.7 (-33.4 to 41.8)	-4.6 (-5.3 to -3.9)

	Number of incident cases (all ages, both sexes) with 95% uncertainty intervals (2016)				Annualized rate of change of age-standardized rate (%) with 95% uncertainty intervals							
					1990-2006				2006-2016			
	**Drug-susceptible HIV-TB**	**MDR HIV-TB**	**XDR HIV-TB**	**All HIV-positive TB**	**Drug-susceptible HIV-TB**	**MDR HIV-TB**	**XDR HIV-TB**	**All HIV-positive TB**	**Drug-susceptible HIV-TB**	**MDR HIV-TB**	**XDR HIV-TB**	**All HIV-positive TB**
Kuwait	3 (1-6)	0 (0-0)	0 (0-0)	3 (1 to 6)	-1.8 (-2.8 to -1.0)	21.1 (11.2 to 34.8)	-3.4 (-12.6 to 2.9)	-1.7 (-2.6 to -0.9)	-10.2 (-11.7 to -8.8)	-10.2 (-23.7 to 3.6)	-2.0 (-15.6 to 11.8)	-10.2 (-11.8 to -8.8)
Kyrgyzstan	93 (38-170)	47 (17-95)	10 (4-21)	149 (88 to 247)	4.3 (0.0 to 62.0)	48.2 (39.8 to 61.3)	40.5 (33.5 to 45.2)	7.1 (5.6 to 64.3)	-1.2 (-9.4 to 6.5)	-1.4 (-13.1 to 11.5)	9.8 (-1.8 to 22.8)	-0.8 (-1.5 to -0.2)
Laos	1,774 (1,113-2,698)	10 (1-45)	1 (0-4)	1,786 (1,123 to 2,708)	80.0 (77.0 to 82.8)	52.7 (37.1 to 62.1)	30.3 (14.7 to 39.7)	80.1 (77.1 to 82.9)	-2.1 (-3.0 to -1.0)	-9.8 (-30.5 to 10.3)	2.1 (-18.6 to 22.2)	-2.1 (-3.0 to -1.2)
Latvia	57 (35-91)	6 (3-10)	1 (1-2)	65 (40 to 101)	3.7 (2.7 to 63.5)	6.6 (3.4 to 51.4)	35.9 (32.3 to 39.0)	4.1 (3.2 to 64.3)	-0.6 (-1.5 to 0.4)	-3.8 (-6.9 to -0.9)	7.5 (4.4 to 10.4)	-0.9 (-1.6 to -0.0)
Lebanon	20 (11-34)	1 (0-2)	0 (0-0)	21 (12 to 35)	-7.1 (-7.6 to -6.6)	10.8 (-1.1 to 25.7)	4.4 (-5.9 to 11.5)	-6.9 (-7.4 to -6.5)	-5.9 (-6.9 to -5.1)	-3.3 (-22.8 to 12.3)	4.9 (-14.6 to 20.5)	-5.8 (-6.5 to -5.2)
Lesotho	26,865 (20,277-34,608)	1,073 (558-1,799)	7 (4-12)	27,945 (20,962 to 35,839)	13.0 (11.0 to 15.2)	42.4 (34.5 to 52.4)	45.2 (38.7 to 50.3)	13.3 (11.3 to 15.4)	-2.3 (-3.4 to -1.1)	-1.6 (-11.9 to 10.3)	9.6 (-0.7 to 21.5)	-2.2 (-3.2 to -1.1)
Liberia	509 (294-842)	10 (2-29)	0 (0-0)	519 (301 to 850)	6.2 (5.0 to 7.4)	19.6 (11.7 to 28.0)	15.5 (6.8 to 22.3)	6.3 (5.2 to 7.5)	-6.0 (-6.9 to -5.0)	-7.0 (-18.4 to 4.2)	4.1 (-7.2 to 15.3)	-6.0 (-6.9 to -5.1)
Libya	189 (98-329)	4 (0-20)	0 (0-1)	193 (99 to 333)	3.2 (2.0 to 4.5)	15.6 (1.2 to 30.0)	10.3 (-6.5 to 19.0)	3.3 (2.1 to 4.6)	2.1 (0.6 to 3.8)	6.9 (-13.0 to 26.5)	15.1 (-4.8 to 34.6)	2.2 (0.9 to 3.8)
Lithuania	19 (11-32)	4 (2-6)	1 (0-1)	24 (14 to 39)	4.6 (3.4 to 53.4)	12.0 (8.5 to 41.5)	29.1 (25.0 to 32.7)	5.3 (4.2 to 54.3)	-3.0 (-3.8 to -1.8)	-2.4 (-4.4 to -0.2)	8.9 (6.9 to 11.1)	-2.7 (-3.4 to -1.5)
Luxembourg	2 (1-3)	0 (0-0)	0 (0-0)	2 (1 to 3)	-7.0 (-7.8 to -6.3)	1.0 (-2.3 to 4.2)	-5.0 (-10.0 to -0.7)	-7.0 (-7.8 to -6.3)	-5.7 (-6.8 to -4.8)	3.2 (-1.4 to 7.9)	10.9 (6.3 to 15.6)	-5.7 (-6.8 to -4.8)
Macedonia	3 (2-6)	0 (0-0)	0 (0-0)	3 (2 to 6)	-1.0 (-1.5 to -0.4)	16.9 (7.0 to 30.8)	0.6 (-11.2 to 8.2)	-1.0 (-1.5 to -0.4)	-3.3 (-4.0 to -2.6)	1.4 (-14.4 to 19.1)	12.6 (-3.2 to 30.4)	-3.3 (-3.9 to -2.6)
Madagascar	161 (90-275)	2 (0-4)	0 (0-0)	162 (91 to 278)	9.2 (7.5 to 10.8)	29.5 (22.5 to 37.6)	-10.2 (-17.1 to -5.1)	9.2 (7.6 to 10.9)	-2.3 (-3.5 to -0.9)	-1.1 (-12.1 to 8.7)	10.0 (-1.0 to 19.9)	-2.3 (-3.5 to -0.9)
Malawi	26,991 (21,255-32,958)	277 (99-589)	2 (1-4)	27,270 (21,509 to 33,445)	5.2 (4.5 to 6.2)	28.5 (21.6 to 36.8)	27.2 (21.5 to 31.9)	5.3 (4.6 to 6.2)	-5.9 (-6.8 to -5.0)	-5.4 (-17.3 to 5.7)	5.8 (-6.2 to 16.8)	-5.9 (-6.8 to -5.0)
Malaysia	1,229 (705-2,045)	11 (4-24)	1 (0-2)	1,241 (713 to 2,065)	12.5 (10.5 to 14.8)	35.5 (24.9 to 48.2)	20.0 (7.6 to 28.0)	12.6 (10.6 to 14.9)	-5.5 (-6.5 to -4.3)	-9.6 (-25.0 to 10.8)	2.3 (-13.1 to 22.7)	-5.5 (-6.6 to -4.4)
Maldives	1 (1-3)	0 (0-0)	0 (0-0)	1 (1 to 3)	-3.4 (-4.3 to -2.4)	10.6 (-3.4 to 25.0)	3.4 (-12.7 to 12.9)	-3.3 (-4.2 to -2.4)	-2.1 (-3.2 to -1.0)	-10.9 (-29.3 to 9.3)	1.0 (-17.4 to 21.3)	-2.2 (-3.3 to -1.1)
Mali	1,307 (786-2,091)	25 (7-68)	0 (0-0)	1,333 (800 to 2,111)	0.9 (0.1 to 1.8)	14.3 (6.2 to 22.3)	12.4 (4.5 to 18.5)	1.1 (0.3 to 1.9)	-4.2 (-5.0 to -3.4)	-5.2 (-16.4 to 5.2)	6.0 (-5.2 to 16.3)	-4.2 (-4.9 to -3.5)

	Number of incident cases (all ages, both sexes) with 95% uncertainty intervals (2016)				Annualized rate of change of age-standardized rate (%) with 95% uncertainty intervals							
					1990-2006				2006-2016			
	Drug-susceptible HIV-TB	MDR HIV-TB	XDR HIV-TB	All HIV-positive TB	Drug-susceptible HIV-TB	MDR HIV-TB	XDR HIV-TB	All HIV-positive TB	Drug-susceptible HIV-TB	MDR HIV-TB	XDR HIV-TB	All HIV-positive TB
Malta	1 (1-2)	0 (0-0)	0 (0-0)	1 (1 to 2)	-5.3 (-6.1 to -4.5)	2.0 (-1.3 to 5.1)	-11.9 (-17.2 to -7.7)	-5.3 (-6.1 to -4.5)	-4.0 (-5.0 to -3.0)	-11.4 (-16.3 to -6.4)	-3.8 (-8.7 to 1.2)	-4.0 (-5.0 to -3.0)
Marshall Islands	2 (1-3)	0 (0-0)	0 (0-0)	2 (1 to 3)	3.4 (2.2 to 4.4)	25.1 (9.6 to 55.2)	21.3 (3.8 to 30.8)	3.7 (2.9 to 4.6)	0.3 (-1.0 to 2.1)	-5.2 (-33.8 to 26.8)	6.7 (-21.9 to 38.8)	0.2 (-0.5 to 0.8)
Mauritania	337 (204-534)	7 (2-17)	0 (0-0)	344 (208 to 545)	0.4 (-0.3 to 1.1)	13.5 (5.8 to 21.8)	12.1 (4.2 to 18.2)	0.5 (-0.2 to 1.2)	-4.6 (-5.4 to -3.8)	-5.7 (-16.1 to 4.7)	5.5 (-5.0 to 15.9)	-4.6 (-5.3 to -3.9)
Mauritius	21 (13-35)	0 (0-0)	0 (0-0)	22 (13 to 35)	-2.5 (-3.3 to -1.5)	16.7 (5.8 to 29.6)	3.3 (-8.5 to 10.7)	-2.4 (-3.3 to -1.5)	5.4 (4.2 to 6.6)	2.9 (-15.3 to 20.3)	14.8 (-3.4 to 32.3)	5.4 (4.2 to 6.6)
Mexico	2,660 (1,649-4,207)	71 (42-117)	6 (3-9)	2,737 (1,695 to 4,327)	2.7 (2.1 to 3.4)	30.0 (28.6 to 31.3)	19.2 (16.0 to 22.3)	2.9 (2.3 to 3.6)	-2.2 (-2.5 to -1.9)	-3.1 (-4.8 to -1.4)	6.7 (5.0 to 8.4)	-2.2 (-2.5 to -1.9)
Moldova	57 (32-94)	32 (18-56)	7 (4-12)	96 (56 to 156)	4.9 (3.7 to 6.1)	43.7 (40.0 to 48.4)	40.9 (36.9 to 44.3)	7.3 (6.5 to 8.2)	-6.6 (-8.9 to -4.4)	-3.7 (-7.7 to -0.0)	7.6 (3.5 to 11.2)	-5.1 (-5.8 to -4.4)
Mongolia	8 (4-15)	0 (0-1)	0 (0-0)	9 (5 to 16)	21.3 (18.8 to 24.7)	40.1 (31.8 to 53.1)	8.0 (1.9 to 12.8)	21.5 (19.0 to 24.9)	4.5 (2.9 to 6.2)	7.9 (-10.8 to 20.4)	19.2 (0.4 to 31.6)	4.7 (3.4 to 6.1)
Montenegro	1 (1-2)	0 (0-0)	0 (0-0)	1 (1 to 2)	2.2 (1.7 to 2.7)	10.1 (-3.1 to 24.8)	-7.4 (-21.8 to 1.0)	2.2 (1.7 to 2.7)	-2.3 (-2.8 to -1.6)	-5.1 (-26.3 to 15.3)	6.2 (-15.0 to 26.6)	-2.3 (-2.8 to -1.6)
Morocco	1,603 (820-2,913)	26 (7-67)	1 (0-2)	1,630 (832 to 2,959)	4.4 (3.1 to 5.7)	28.3 (17.0 to 42.7)	18.3 (5.6 to 26.2)	4.5 (3.2 to 5.9)	-4.2 (-5.7 to -3.0)	-5.0 (-20.5 to 14.8)	3.2 (-12.3 to 23.0)	-4.2 (-5.7 to -3.0)
Mozambique	48,501 (35,375-63,293)	2,112 (693-4,882)	14 (4-31)	50,627 (36,832 to 65,873)	7.3 (5.8 to 9.0)	25.2 (18.9 to 32.7)	34.8 (30.8 to 38.0)	7.6 (6.0 to 9.2)	-2.3 (-3.3 to -1.3)	-2.2 (-13.2 to 6.8)	8.9 (-2.0 to 18.0)	-2.3 (-3.2 to -1.3)
Myanmar	15,060 (8,917-23,881)	882 (289-2,119)	80 (26-193)	16,023 (9,555 to 25,639)	14.2 (11.8 to 16.7)	34.7 (25.6 to 47.8)	42.5 (37.6 to 46.5)	14.5 (12.2 to 17.0)	-8.5 (-9.8 to -7.3)	-7.1 (-18.0 to 1.7)	4.8 (-6.0 to 13.6)	-8.4 (-9.5 to -7.3)
Namibia	8,751 (6,127-11,830)	456 (241-722)	3 (2-5)	9,210 (6,413 to 12,457)	11.1 (9.3 to 13.0)	42.1 (34.3 to 51.4)	42.9 (35.8 to 48.3)	11.4 (9.6 to 13.2)	-7.9 (-9.2 to -6.6)	-7.4 (-17.0 to 4.7)	3.7 (-5.9 to 15.9)	-7.9 (-9.2 to -6.7)
Nepal	584 (344-970)	19 (10-32)	0 (0-1)	603 (355 to 1,000)	65.6 (62.4 to 68.7)	43.6 (39.9 to 46.9)	14.4 (10.7 to 17.7)	65.8 (62.6 to 68.9)	-4.9 (-5.4 to -4.3)	-4.2 (-6.6 to -1.7)	5.5 (3.1 to 8.0)	-4.8 (-5.4 to -4.3)
Netherlands	71 (39-122)	1 (1-2)	0 (0-0)	72 (40 to 124)	-8.9 (-10.1 to -7.8)	-2.8 (-5.8 to 0.2)	8.7 (4.4 to 12.2)	-8.8 (-10.0 to -7.7)	-5.8 (-6.9 to -4.7)	-1.5 (-5.5 to 2.4)	6.2 (2.1 to 10.0)	-5.7 (-6.9 to -4.6)
New Zealand	7 (4-11)	0 (0-0)	0 (0-0)	7 (4 to 12)	-9.3 (-10.6 to -8.2)	-5.5 (-14.2 to 5.7)	3.0 (-3.5 to 7.8)	-9.3 (-10.5 to -8.2)	-5.3 (-6.1 to -4.6)	-3.2 (-16.1 to 8.9)	4.5 (-8.4 to 16.5)	-5.2 (-6.0 to -4.5)
Nicaragua	63 (37-106)	1 (0-2)	0 (0-0)	64 (38 to 108)	1.0 (0.1 to 1.9)	12.9 (8.3 to 17.5)	10.7 (5.3 to 14.8)	1.0 (0.1 to 1.9)	-3.2 (-3.8 to -2.5)	-3.5 (-9.8 to 3.2)	6.3 (0.0 to 13.0)	-3.2 (-3.8 to -2.5)

	Number of incident cases (all ages, both sexes) with 95% uncertainty intervals (2016)				Annualized rate of change of age-standardized rate (%) with 95% uncertainty intervals							
					1990-2006				2006-2016			
	Drug-susceptible HIV-TB	MDR HIV-TB	XDR HIV-TB	All HIV-positive TB	Drug-susceptible HIV-TB	MDR HIV-TB	XDR HIV-TB	All HIV-positive TB	Drug-susceptibl e HIV-TB	MDR HIV-TB	XDR HIV-TB	All HIV-positive TB
Niger	1,632 (969-2,645)	32 (8-95)	0 (0-1)	1,664 (984 to 2,673)	7.4 (6.1 to 8.7)	20.9 (12.8 to 29.0)	14.8 (6.2 to 21.2)	7.5 (6.2 to 8.8)	-6.8 (-7.9 to -5.8)	-7.9 (-19.4 to 3.3)	3.3 (-8.2 to 14.4)	-6.9 (-7.8 to -5.9)
Nigeria	60,216 (35,769-93,946)	2,367 (879-5,374)	15 (6-35)	62,598 (37,436 to 96,918)	5.3 (4.4 to 6.2)	33.8 (27.4 to 41.3)	26.7 (21.0 to 31.3)	5.5 (4.6 to 6.5)	-6.9 (-7.8 to -6.0)	-7.3 (-17.8 to 2.6)	3.8 (-6.6 to 13.7)	-6.9 (-7.7 to -6.1)
North Korea	2,459 (1,459-4,025)	69 (32-132)	6 (3-12)	2,534 (1,512 to 4,116)	10.5 (8.9 to 12.5)	42.9 (38.9 to 47.0)	24.6 (19.4 to 28.8)	10.7 (9.1 to 12.6)	3.8 (3.1 to 4.5)	3.1 (-2.1 to 8.5)	15.0 (9.8 to 20.5)	3.8 (3.1 to 4.5)
Northern Mariana Islands	2 (1-4)	0 (0-0)	0 (0-0)	3 (2 to 4)	2.5 (1.1 to 3.7)	14.0 (0.1 to 44.4)	20.6 (10.9 to 27.6)	2.9 (1.9 to 3.9)	2.7 (1.0 to 4.5)	-6.4 (-34.1 to 14.0)	5.5 (-22.2 to 25.9)	2.2 (1.4 to 3.1)
Norway	10 (5-17)	0 (0-1)	0 (0-0)	10 (5 to 18)	-3.4 (-4.1 to -2.8)	-0.6 (-3.2 to 2.5)	8.7 (4.2 to 12.6)	-3.3 (-4.1 to -2.7)	-5.2 (-6.3 to -4.1)	-4.4 (-8.7 to -0.5)	3.3 (-1.0 to 7.2)	-5.1 (-6.3 to -4.1)
Oman	18 (9-33)	0 (0-1)	0 (0-0)	18 (9 to 33)	1.8 (0.8 to 2.6)	5.6 (-5.0 to 20.1)	-3.1 (-9.7 to 2.0)	1.8 (0.9 to 2.6)	1.4 (-0.6 to 3.0)	5.7 (-6.6 to 17.9)	13.9 (1.6 to 26.1)	1.5 (-0.5 to 3.1)
Pakistan	2,221 (1,229-3,839)	105 (56-185)	3 (1-5)	2,328 (1,296 to 3,999)	1.7 (1.1 to 2.4)	33.7 (31.8 to 35.6)	8.1 (3.8 to 12.0)	2.0 (1.4 to 2.7)	3.7 (2.4 to 5.1)	3.5 (0.7 to 6.3)	13.2 (10.4 to 16.0)	3.7 (2.4 to 5.0)
Palestine	2 (1-3)	0 (0-0)	0 (0-0)	2 (1 to 3)	1.8 (0.6 to 3.2)	14.0 (-0.3 to 30.2)	-14.0 (-30.6 to -4.6)	1.9 (0.7 to 3.3)	-2.6 (-4.0 to -1.2)	1.4 (-16.8 to 20.7)	9.6 (-8.6 to 28.9)	-2.5 (-3.7 to -1.2)
Panama	215 (133-328)	3 (1-7)	0 (0-1)	219 (136 to 335)	5.6 (4.6 to 6.8)	21.2 (16.4 to 25.9)	22.6 (16.7 to 27.0)	5.7 (4.7 to 6.9)	-2.7 (-3.4 to -2.1)	-3.9 (-10.4 to 2.8)	5.9 (-0.6 to 12.6)	-2.7 (-3.3 to -2.1)
Papua New Guinea	923 (558-1,479)	50 (7-160)	5 (1-15)	978 (602 to 1,563)	11.6 (8.0 to 13.8)	36.9 (21.0 to 68.6)	39.8 (17.9 to 50.3)	12.2 (10.5 to 14.1)	-5.5 (-7.5 to 0.6)	-10.4 (-34.0 to 24.6)	1.5 (-22.1 to 36.5)	-5.8 (-6.7 to -5.0)
Paraguay	364 (226-575)	6 (3-10)	0 (0-1)	370 (230 to 586)	5.3 (4.4 to 6.3)	23.1 (21.3 to 24.9)	24.6 (21.4 to 27.6)	5.4 (4.5 to 6.4)	-5.3 (-6.3 to -4.4)	-6.6 (-9.1 to -4.2)	3.2 (0.7 to 5.6)	-5.3 (-6.4 to -4.4)
Peru	920 (554-1,516)	59 (33-102)	5 (3-8)	984 (589 to 1,623)	-5.5 (-6.0 to -5.0)	6.1 (1.4 to 12.1)	31.2 (27.4 to 34.4)	-5.2 (-5.7 to -4.7)	-10.7 (-12.0 to -9.5)	-9.9 (-13.4 to -6.4)	-0.1 (-3.6 to 3.4)	-10.6 (-11.9 to -9.5)
Philippines	420 (247-692)	15 (4-40)	1 (0-4)	436 (255 to 716)	2.1 (0.9 to 3.2)	31.4 (20.9 to 44.3)	10.4 (0.1 to 17.1)	2.4 (1.4 to 3.4)	0.0 (-1.0 to 1.5)	-2.8 (-21.2 to 15.5)	9.1 (-9.3 to 27.5)	-0.1 (-0.8 to 0.6)
Poland	90 (50-156)	1 (0-1)	0 (0-0)	91 (50 to 157)	-0.9 (-1.7 to -0.1)	2.6 (-5.0 to 13.2)	2.7 (-2.2 to 6.7)	-0.9 (-1.7 to -0.1)	-3.6 (-4.2 to -2.9)	-5.7 (-12.1 to 1.1)	5.6 (-0.8 to 12.4)	-3.6 (-4.3 to -2.9)
Portugal	277 (163-443)	4 (2-6)	0 (0-1)	281 (165 to 451)	1.9 (0.9 to 3.2)	3.8 (1.3 to 6.8)	23.3 (19.7 to 26.4)	2.0 (0.9 to 3.2)	-7.9 (-9.0 to -6.7)	-8.7 (-12.5 to -5.2)	-1.0 (-4.8 to 2.5)	-7.9 (-9.0 to -6.7)
Puerto Rico	27 (17-41)	0 (0-1)	0 (0-0)	27 (17 to 41)	-8.8 (-9.6 to -8.0)	7.5 (-2.3 to 19.5)	10.0 (0.7 to 16.4)	-8.7 (-9.4 to -7.9)	-5.6 (-6.5 to -4.7)	-12.3 (-29.1 to 3.5)	-2.5 (-19.3 to 13.3)	-5.7 (-6.6 to -4.8)

	Number of incident cases (all ages, both sexes) with 95% uncertainty intervals (2016)				Annualized rate of change of age-standardized rate (%) with 95% uncertainty intervals							
					1990-2006				2006-2016			
	Drug-susceptible HIV-TB	MDR HIV-TB	XDR HIV-TB	All HIV-positive TB	Drug-susceptible HIV-TB	MDR HIV-TB	XDR HIV-TB	All HIV-positive TB	Drug-susceptible HIV-TB	MDR HIV-TB	XDR HIV-TB	All HIV-positive TB
Qatar	2 (1-3)	0 (0-0)	0 (0-0)	2 (1 to 3)	-4.8 (-5.8 to -3.8)	4.3 (-6.1 to 18.3)	-9.5 (-17.6 to -3.3)	-4.7 (-5.7 to -3.7)	-7.4 (-9.0 to -5.8)	-3.2 (-20.1 to 11.1)	5.0 (-11.9 to 19.3)	-7.3 (-8.9 to -5.7)
Romania	249 (141-417)	8 (3-18)	2 (1-4)	258 (148 to 436)	4.0 (3.3 to 4.9)	11.5 (2.9 to 21.6)	23.3 (16.5 to 28.4)	4.2 (3.5 to 5.0)	-2.5 (-3.2 to -1.8)	-3.2 (-13.7 to 7.5)	8.0 (-2.4 to 18.7)	-2.5 (-3.1 to -1.9)
Russia	3,870 (2,288-6,298)	826 (427-1,490)	181 (94-326)	4,876 (2,905 to 7,859)	6.9 (5.8 to 7.8)	12.8 (9.4 to 16.7)	35.8 (31.8 to 39.3)	7.5 (6.5 to 8.3)	-1.4 (-2.8 to -0.0)	1.8 (-2.1 to 5.3)	13.0 (9.1 to 16.5)	-0.6 (-1.5 to 0.4)
Rwanda	6,005 (3,749-9,048)	122 (55-237)	1 (0-2)	6,128 (3,821 to 9,270)	-1.3 (-2.0 to -0.6)	26.9 (19.4 to 35.2)	27.3 (19.8 to 32.5)	-1.2 (-1.9 to -0.5)	-9.2 (-10.9 to -7.5)	-9.5 (-18.6 to 1.8)	1.6 (-7.5 to 12.9)	-9.2 (-10.8 to -7.6)
Saint Lucia	5 (3-8)	0 (0-0)	0 (0-0)	5 (3 to 8)	2.5 (1.7 to 3.2)	8.6 (-4.2 to 21.8)	14.7 (0.0 to 23.6)	2.5 (1.8 to 3.2)	-3.9 (-4.7 to -3.1)	-7.4 (-25.4 to 11.2)	2.4 (-15.5 to 21.0)	-4.0 (-4.7 to -3.1)
Saint Vincent and the Grenadines	7 (4-10)	0 (0-0)	0 (0-0)	7 (4 to 10)	3.9 (3.1 to 4.7)	10.0 (-2.1 to 22.0)	18.6 (5.0 to 27.2)	3.9 (3.1 to 4.7)	-2.4 (-3.1 to -1.6)	-6.0 (-24.1 to 10.0)	3.8 (-14.3 to 19.8)	-2.4 (-3.1 to -1.7)
Samoa	2 (1-3)	0 (0-0)	0 (0-0)	2 (1 to 3)	3.0 (2.1 to 3.9)	6.1 (-21.5 to 34.4)	-2.9 (-37.9 to 10.2)	3.0 (2.1 to 3.9)	1.4 (0.7 to 2.1)	1.2 (-36.3 to 38.0)	13.1 (-24.4 to 49.9)	1.4 (0.8 to 2.1)
Sao Tome and Principe	10 (6-16)	0 (0-1)	0 (0-0)	10 (6 to 16)	4.3 (3.4 to 5.2)	17.8 (9.9 to 25.5)	12.5 (4.1 to 18.9)	4.4 (3.6 to 5.4)	-8.6 (-9.8 to -7.4)	-9.5 (-20.6 to 2.7)	1.7 (-9.4 to 13.8)	-8.6 (-9.8 to -7.4)
Saudi Arabia	348 (205-578)	12 (4-33)	0 (0-1)	360 (215 to 594)	5.2 (4.5 to 6.1)	31.3 (24.6 to 38.2)	8.6 (1.7 to 14.7)	5.4 (4.7 to 6.3)	1.8 (1.1 to 2.3)	2.8 (-6.3 to 12.2)	11.0 (1.9 to 20.4)	1.8 (1.4 to 2.2)
Senegal	1,831 (1,094-2,929)	20 (8-45)	0 (0-0)	1,851 (1,113 to 2,960)	5.7 (4.6 to 6.8)	27.4 (19.7 to 34.9)	11.5 (3.7 to 17.1)	5.8 (4.7 to 6.9)	-4.7 (-5.5 to -3.9)	-5.2 (-15.2 to 5.6)	6.0 (-4.1 to 16.8)	-4.7 (-5.5 to -3.8)
Serbia	117 (64-205)	1 (0-3)	0 (0-1)	119 (64 to 207)	-2.0 (-4.7 to 64.5)	8.2 (-1.5 to 32.9)	13.6 (7.6 to 18.5)	-2.0 (-4.7 to 64.5)	-5.9 (-6.6 to -5.1)	-2.5 (-15.1 to 9.2)	8.8 (-3.9 to 20.5)	-5.9 (-6.5 to -5.1)
Seychelles	2 (1-4)	0 (0-0)	0 (0-0)	2 (1 to 4)	9.4 (7.7 to 11.4)	13.2 (-0.1 to 27.7)	-6.2 (-21.8 to 3.0)	9.4 (7.7 to 11.4)	1.8 (0.7 to 2.9)	0.3 (-20.9 to 20.5)	12.3 (-9.0 to 32.4)	1.8 (0.7 to 2.9)
Sierra Leone	1,266 (738-2,055)	19 (5-52)	0 (0-0)	1,285 (745 to 2,087)	13.9 (12.3 to 15.6)	22.3 (13.9 to 30.4)	15.1 (7.6 to 20.8)	14.0 (12.4 to 15.7)	-3.5 (-4.2 to -2.8)	-3.6 (-15.0 to 7.4)	7.5 (-3.9 to 18.6)	-3.5 (-4.2 to -2.8)
Singapore	51 (29-84)	0 (0-1)	0 (0-0)	51 (30 to 85)	0.1 (-0.9 to 1.1)	4.0 (0.5 to 7.4)	7.1 (2.7 to 10.8)	0.1 (-0.8 to 1.2)	-2.0 (-2.8 to -1.3)	3.7 (-0.8 to 8.2)	11.3 (6.9 to 15.9)	-2.0 (-2.8 to -1.2)
Slovakia	1 (0-2)	0 (0-0)	0 (0-0)	1 (0 to 2)	2.7 (1.6 to 33.4)	7.0 (-2.3 to 20.0)	-10.5 (-17.6 to -5.3)	2.7 (1.6 to 33.5)	-4.3 (-5.0 to -3.3)	-7.5 (-25.6 to 6.1)	3.7 (-14.4 to 17.3)	-4.4 (-5.0 to -3.4)
Slovenia	2 (1-3)	0 (0-0)	0 (0-0)	2 (1 to 3)	-7.0 (-7.9 to 43.1)	-6.0 (-17.1 to 8.9)	-7.1 (-15.6 to -1.2)	-7.0 (-7.9 to 43.1)	-5.9 (-6.7 to -5.2)	-20.1 (-39.4 to -5.8)	-8.8 (-28.1 to 5.4)	-6.0 (-6.8 to -5.3)

	Number of incident cases (all ages, both sexes) with 95% uncertainty intervals (2016)				Annualized rate of change of age-standardized rate (%) with 95% uncertainty intervals							
					1990-2006				2006-2016			
	**Drug-susceptible HIV-TB**	**MDR HIV-TB**	**XDR HIV-TB**	**All HIV-positive TB**	**Drug-susceptible HIV-TB**	**MDR HIV-TB**	**XDR HIV-TB**	**All HIV-positive TB**	**Drug-susceptible HIV-TB**	**MDR HIV-TB**	**XDR HIV-TB**	**All HIV-positive TB**
Solomon Islands	16 (10-27)	0 (0-2)	0 (0-0)	17 (10 to 27)	2.7 (1.6 to 3.7)	15.2 (-10.4 to 43.8)	17.4 (-15.1 to 30.7)	2.8 (2.0 to 3.7)	0.4 (-0.6 to 1.8)	-3.3 (-37.6 to 33.1)	8.7 (-25.6 to 45.0)	0.3 (-0.2 to 0.9)
Somalia	1,497 (863-2,413)	108 (38-240)	1 (0-2)	1,606 (928 to 2,608)	8.3 (7.1 to 9.3)	42.5 (35.5 to 50.1)	21.8 (15.2 to 26.8)	8.7 (7.7 to 9.6)	-1.4 (-2.5 to -0.2)	-1.1 (-11.9 to 9.8)	10.1 (-0.8 to 20.9)	-1.4 (-2.1 to -0.7)
South Africa	241,274 (186,594-303,738)	6,065 (3,352-10,829)	39 (22-70)	247,378 (191,531 to 311,090)	18.0 (15.9 to 20.5)	29.4 (24.4 to 34.9)	37.2 (33.5 to 40.9)	18.2 (16.0 to 20.6)	-4.0 (-4.6 to -3.3)	-4.9 (-10.6 to 0.4)	6.2 (0.5 to 11.6)	-4.0 (-4.6 to -3.3)
South Korea	648 (349-1,140)	12 (5-24)	2 (1-3)	662 (357 to 1,161)	-5.8 (-7.3 to 61.3)	4.1 (0.1 to 38.4)	18.7 (14.0 to 22.6)	-5.7 (-7.2 to 61.5)	-3.7 (-4.3 to -3.1)	-6.1 (-10.4 to -1.6)	1.6 (-2.8 to 6.1)	-3.8 (-4.4 to -3.2)
South Sudan	2,957 (1,731-4,846)	73 (19-210)	0 (0-1)	3,030 (1,766 to 4,973)	6.6 (5.8 to 7.4)	26.8 (18.2 to 34.9)	17.1 (7.9 to 23.4)	6.7 (5.9 to 7.5)	-1.4 (-2.3 to -0.5)	-0.4 (-11.8 to 10.8)	10.7 (-0.6 to 21.9)	-1.4 (-2.2 to -0.6)
Spain	348 (196-581)	4 (2-8)	1 (0-1)	352 (198 to 588)	-6.6 (-7.7 to -5.5)	-4.3 (-7.6 to -1.0)	14.4 (9.6 to 18.3)	-6.6 (-7.7 to -5.5)	-9.7 (-10.9 to -8.5)	-9.3 (-14.1 to -4.6)	-1.7 (-6.4 to 3.0)	-9.7 (-11.0 to -8.5)
Sri Lanka	43 (23-76)	0 (0-1)	0 (0-0)	43 (23 to 76)	2.6 (1.8 to 3.5)	15.4 (3.1 to 29.5)	-5.4 (-18.1 to 2.4)	2.6 (1.8 to 3.5)	-6.4 (-7.3 to -5.6)	-8.1 (-28.4 to 9.8)	3.8 (-16.5 to 21.7)	-6.4 (-7.3 to -5.6)
Sudan	2,693 (1,586-4,356)	61 (4-281)	2 (0-10)	2,757 (1,657 to 4,418)	1.5 (0.7 to 2.3)	14.3 (-0.3 to 28.4)	20.4 (4.0 to 29.5)	1.6 (0.9 to 2.3)	-3.3 (-4.2 to -2.3)	0.6 (-19.7 to 19.7)	8.8 (-11.5 to 27.9)	-3.2 (-3.9 to -2.4)
Suriname	39 (25-58)	0 (0-1)	0 (0-0)	39 (25 to 58)	8.4 (7.1 to 9.8)	14.0 (1.4 to 26.2)	22.4 (8.4 to 31.1)	8.4 (7.2 to 9.9)	-7.2 (-8.2 to -6.2)	-10.2 (-28.7 to 7.4)	-0.4 (-18.9 to 17.2)	-7.2 (-8.2 to -6.3)
Swaziland	10,266 (7,675-13,338)	926 (339-2,004)	6 (2-13)	11,198 (8,476 to 14,470)	14.5 (12.3 to 17.0)	34.2 (27.4 to 41.8)	49.2 (45.3 to 53.0)	14.9 (12.7 to 17.4)	-6.6 (-8.1 to -5.3)	-4.2 (-16.6 to 6.7)	6.9 (-5.4 to 17.8)	-6.4 (-7.5 to -5.4)
Sweden	20 (11-35)	1 (0-1)	0 (0-0)	21 (11 to 36)	-4.1 (-4.9 to -3.6)	2.1 (-0.5 to 4.4)	7.5 (2.9 to 11.2)	-4.0 (-4.8 to -3.5)	-2.8 (-3.6 to -2.0)	0.9 (-2.4 to 4.0)	8.5 (5.3 to 11.6)	-2.6 (-3.4 to -1.9)
Switzerland	24 (13-42)	1 (0-1)	0 (0-0)	25 (14 to 43)	-9.0 (-10.0 to -8.0)	-3.3 (-6.4 to -0.1)	9.3 (4.8 to 13.0)	-8.9 (-9.9 to -8.0)	-4.9 (-6.0 to -3.8)	-0.8 (-4.8 to 3.2)	6.9 (2.9 to 10.9)	-4.8 (-5.9 to -3.6)
Syria	21 (9-38)	2 (0-8)	0 (0-0)	22 (10 to 44)	-1.9 (-3.0 to -0.8)	21.4 (10.5 to 36.4)	2.9 (-5.5 to 9.2)	-1.4 (-2.3 to -0.4)	-0.6 (-3.4 to 1.1)	0.3 (-19.0 to 14.5)	8.5 (-10.8 to 22.7)	-0.5 (-1.8 to 0.7)
Taiwan	119 (70-198)	2 (1-4)	0 (0-0)	122 (71 to 202)	6.9 (5.4 to 8.6)	34.4 (29.8 to 38.6)	4.8 (-0.6 to 9.4)	7.0 (5.5 to 8.7)	1.9 (1.3 to 2.6)	0.3 (-4.7 to 5.5)	12.2 (7.2 to 17.5)	1.9 (1.3 to 2.6)
Tajikistan	30 (17-52)	4 (2-9)	1 (0-2)	36 (20 to 60)	12.0 (9.4 to 14.0)	50.7 (41.4 to 63.0)	32.2 (23.0 to 38.7)	13.0 (11.5 to 14.7)	-13.1 (-15.6 to -9.5)	-14.4 (-25.9 to 1.3)	-3.2 (-14.7 to 12.6)	-13.1 (-14.6 to -11.8)

	Number of incident cases (all ages, both sexes) with 95% uncertainty intervals (2016)				Annualized rate of change of age-standardized rate (%) with 95% uncertainty intervals							
					1990-2006				2006-2016			
	Drug-susceptible HIV-TB	MDR HIV-TB	XDR HIV-TB	All HIV-positive TB	Drug-susceptible HIV-TB	MDR HIV-TB	XDR HIV-TB	All HIV-positive TB	Drug-susceptible HIV-TB	MDR HIV-TB	XDR HIV-TB	All HIV-positive TB
Tanzania	34,132 (22,705-47,260)	527 (157-1,315)	3 (1-8)	34,662 (23,115 to 47,753)	5.4 (4.3 to 6.5)	28.5 (22.0 to 36.4)	23.4 (18.5 to 27.6)	5.5 (4.4 to 6.6)	-5.4 (-6.5 to -4.2)	-4.2 (-15.4 to 6.0)	7.0 (-4.2 to 17.1)	-5.3 (-6.5 to -4.2)
Thailand	8,018 (4,818-12,817)	261 (71-679)	24 (6-62)	8,302 (5,017 to 13,398)	10.9 (9.0 to 13.2)	17.0 (8.7 to 28.5)	29.4 (24.1 to 33.7)	11.0 (9.1 to 13.3)	-7.1 (-8.4 to -5.9)	-1.7 (-14.6 to 9.2)	10.2 (-2.7 to 21.1)	-7.0 (-8.2 to -5.9)
The Bahamas	40 (25-60)	1 (0-2)	0 (0-0)	41 (26 to 61)	0.2 (-0.4 to 0.8)	17.3 (6.0 to 30.5)	22.1 (9.5 to 30.2)	0.3 (-0.3 to 0.8)	-3.6 (-4.3 to -2.8)	1.4 (-17.1 to 18.2)	11.2 (-7.3 to 28.0)	-3.5 (-4.1 to -2.8)
The Gambia	395 (238-615)	7 (2-20)	0 (0-0)	403 (243 to 626)	10.4 (9.0 to 11.8)	23.9 (15.8 to 31.6)	17.1 (8.2 to 23.0)	10.5 (9.2 to 11.9)	-2.3 (-2.9 to -1.7)	-3.4 (-14.2 to 7.3)	7.7 (-3.1 to 18.4)	-2.3 (-2.9 to -1.8)
Timor-Leste	12 (7-20)	0 (0-0)	0 (0-0)	12 (7 to 20)	9.9 (8.0 to 11.7)	23.3 (9.7 to 38.0)	12.1 (-2.9 to 21.2)	9.9 (8.0 to 11.8)	-4.2 (-5.2 to -3.1)	-11.7 (-32.7 to 6.4)	0.2 (-20.7 to 18.4)	-4.2 (-5.2 to -3.3)
Togo	2,218 (1,331-3,522)	42 (10-111)	0 (0-1)	2,260 (1,354 to 3,615)	9.1 (8.0 to 10.4)	22.3 (14.8 to 30.7)	21.3 (12.8 to 27.5)	9.3 (8.1 to 10.5)	-5.7 (-6.7 to -4.8)	-6.6 (-18.3 to 4.0)	4.5 (-7.1 to 15.2)	-5.7 (-6.7 to -4.9)
Tonga	1 (1-1)	0 (0-0)	0 (0-0)	1 (1 to 1)	3.3 (2.2 to 4.3)	14.4 (-10.6 to 42.6)	9.1 (-24.2 to 20.8)	3.4 (2.6 to 4.3)	1.8 (0.8 to 3.3)	-2.0 (-39.8 to 34.4)	9.9 (-27.9 to 46.3)	1.8 (1.1 to 2.4)
Trinidad and Tobago	50 (32-74)	0 (0-1)	0 (0-0)	51 (32 to 75)	0.5 (-0.5 to 1.2)	6.1 (-7.6 to 20.4)	15.2 (-0.8 to 23.8)	0.5 (-0.4 to 1.3)	-3.0 (-3.8 to -1.6)	-6.2 (-25.0 to 13.2)	3.6 (-15.2 to 23.0)	-3.0 (-3.8 to -1.6)
Tunisia	88 (45-159)	1 (0-2)	0 (0-0)	89 (46 to 161)	3.5 (2.2 to 5.1)	24.4 (11.9 to 38.7)	0.1 (-13.9 to 7.8)	3.6 (2.3 to 5.2)	2.1 (0.7 to 3.6)	0.2 (-15.6 to 20.7)	8.4 (-7.4 to 28.9)	2.1 (0.8 to 3.5)
Turkey	134 (71-233)	5 (2-11)	0 (0-0)	139 (73 to 240)	5.4 (2.6 to 49.3)	31.0 (18.5 to 47.7)	0.9 (-11.4 to 8.8)	5.7 (3.0 to 49.6)	-2.2 (-3.1 to -0.8)	-2.5 (-17.3 to 17.2)	5.7 (-9.1 to 25.4)	-2.2 (-2.9 to -1.3)
Turkmenistan	277 (161-462)	58 (24-120)	13 (5-26)	348 (203 to 573)	2.9 (0.3 to 4.1)	42.6 (33.8 to 54.7)	44.4 (36.3 to 50.4)	4.3 (3.8 to 4.8)	-5.2 (-8.4 to -0.4)	-5.7 (-18.1 to 9.1)	5.6 (-6.8 to 20.3)	-5.0 (-5.8 to -4.4)
Uganda	69,067 (51,096-89,616)	1,514 (632-3,102)	10 (4-20)	70,591 (52,273 to 91,642)	2.4 (1.7 to 3.0)	30.3 (23.6 to 37.6)	32.5 (27.0 to 37.0)	2.5 (1.9 to 3.1)	-4.6 (-5.6 to -3.7)	-4.3 (-14.8 to 6.7)	6.8 (-3.7 to 17.9)	-4.6 (-5.5 to -3.7)
Ukraine	2,388 (1,395-3,795)	896 (484-1,509)	196 (106-331)	3,480 (2,121 to 5,373)	8.3 (6.3 to 9.9)	47.8 (43.4 to 52.7)	47.3 (42.4 to 51.2)	10.3 (9.2 to 11.5)	-4.6 (-6.9 to -2.0)	-4.2 (-9.3 to 1.6)	7.1 (2.0 to 12.9)	-4.1 (-4.7 to -3.3)
United Arab Emirates	28 (17-44)	1 (0-3)	0 (0-0)	29 (18 to 45)	2.9 (2.0 to 3.9)	15.8 (0.7 to 30.6)	-4.6 (-20.9 to 5.1)	3.0 (2.1 to 4.0)	11.8 (7.6 to 16.3)	13.9 (-4.9 to 34.3)	22.1 (3.3 to 42.5)	11.9 (7.7 to 16.3)
United Kingdom	19 (10-33)	0 (0-0)	0 (0-0)	19 (10 to 34)	-2.4 (-2.7 to -2.1)	0.2 (-0.4 to 0.7)	-7.7 (-11.6 to -4.2)	-2.4 (-2.7 to -2.0)	-4.8 (-5.2 to -4.4)	-3.8 (-4.5 to -3.1)	3.9 (3.2 to 4.5)	-4.8 (-5.2 to -4.4)
United States	933 (568-1,451)	13 (8-21)	2 (1-3)	948 (577 to 1,473)	-6.2 (-7.3 to -5.2)	-7.2 (-8.3 to -6.1)	8.5 (5.4 to 11.2)	-6.2 (-7.3 to -5.2)	-6.4 (-7.2 to -5.7)	-6.3 (-7.2 to -5.4)	1.4 (0.4 to 2.3)	-6.4 (-7.2 to -5.7)

	Number of incident cases (all ages, both sexes) with 95% uncertainty intervals (2016)				Annualized rate of change of age-standardized rate (%) with 95% uncertainty intervals							
					1990-2006				2006-2016			
	Drug-susceptible HIV-TB	MDR HIV-TB	XDR HIV-TB	All HIV-positive TB	Drug-susceptible HIV-TB	MDR HIV-TB	XDR HIV-TB	All HIV-positive TB	Drug-susceptibl e HIV-TB	MDR HIV-TB	XDR HIV-TB	All HIV-positive TB
Uruguay	105 (66-161)	0 (0-1)	0 (0-0)	105 (66 to 161)	1.1 (0.3 to 2.0)	3.6 (-8.2 to 17.0)	2.9 (-8.4 to 10.1)	1.1 (0.3 to 2.0)	0.7 (0.0 to 1.4)	11.5 (-3.1 to 28.8)	19.2 (4.5 to 36.4)	0.7 (0.1 to 1.4)
Uzbekistan	202 (87-366)	91 (30-191)	20 (7-42)	313 (184 to 516)	-0.2 (-4.0 to 1.6)	42.7 (34.5 to 54.9)	36.0 (29.7 to 40.7)	2.3 (1.6 to 3.1)	-3.9 (-11.0 to 3.5)	-4.1 (-17.2 to 7.9)	7.2 (-5.9 to 19.1)	-3.5 (-4.2 to -3.0)
Vanuatu	7 (4-12)	0 (0-0)	0 (0-0)	7 (5 to 12)	3.5 (2.7 to 4.3)	9.3 (-19.9 to 32.9)	4.0 (-30.1 to 14.0)	3.5 (2.7 to 4.3)	0.2 (-0.5 to 0.8)	-0.7 (-37.1 to 35.5)	11.3 (-25.2 to 47.4)	0.2 (-0.5 to 0.8)
Venezuela	873 (539-1,350)	14 (5-28)	1 (0-2)	889 (551 to 1,373)	-0.0 (-0.7 to 0.8)	15.7 (10.9 to 20.6)	17.0 (11.5 to 21.5)	0.1 (-0.6 to 0.9)	-0.2 (-0.7 to 0.4)	-1.5 (-8.3 to 4.8)	8.3 (1.5 to 14.6)	-0.2 (-0.7 to 0.4)
Vietnam	10,873 (6,540-17,122)	499 (131-1,311)	45 (12-120)	11,417 (6,870 to 18,265)	11.3 (9.2 to 13.4)	24.8 (16.4 to 36.5)	31.5 (26.1 to 35.9)	11.5 (9.5 to 13.6)	-4.1 (-5.1 to -3.3)	-1.8 (-15.7 to 9.2)	10.1 (-3.8 to 21.1)	-4.0 (-4.7 to -3.3)
Virgin Islands, U.S.	3 (2-5)	0 (0-0)	0 (0-0)	3 (2 to 5)	4.2 (3.4 to 5.1)	10.0 (-3.2 to 23.6)	13.1 (-1.4 to 21.9)	4.2 (3.4 to 5.1)	1.0 (0.4 to 1.7)	-2.5 (-20.4 to 16.4)	7.3 (-10.6 to 26.2)	1.0 (0.4 to 1.6)
Yemen	377 (190-687)	10 (2-31)	0 (0-1)	387 (194 to 694)	-0.7 (-1.5 to 0.1)	18.7 (9.0 to 33.9)	14.4 (7.1 to 19.6)	-0.5 (-1.3 to 0.3)	-2.7 (-4.1 to -1.5)	-4.2 (-20.4 to 9.1)	4.0 (-12.2 to 17.3)	-2.8 (-4.0 to -1.6)
Zambia	55,496 (43,293-68,748)	900 (301-2,204)	6 (2-14)	56,402 (44,051 to 69,821)	7.9 (7.2 to 8.6)	23.4 (16.6 to 30.8)	35.4 (30.9 to 39.4)	8.0 (7.3 to 8.7)	-6.3 (-7.3 to -5.3)	-5.8 (-16.0 to 4.5)	5.3 (-4.8 to 15.7)	-6.3 (-7.3 to -5.3)
Zimbabwe	80,433 (60,455-102,985)	2,664 (627-7,687)	17 (4-50)	83,114 (62,589 to 105,903)	6.2 (5.4 to 7.2)	21.4 (11.5 to 31.0)	41.6 (31.9 to 47.7)	6.4 (5.5 to 7.3)	-9.2 (-10.6 to -7.9)	-5.7 (-18.2 to 6.7)	5.4 (-7.1 to 17.9)	-9.1 (-10.4 to -7.8)

eTable 9. Tuberculosis, drug-susceptible tuberculosis, multidrug-resistant tuberculosis, and extensively drug-resistant tuberculosis deaths in HIV-positive individuals, and annualized rates of change of age-standardized rates for 195 countries and territories

	Number of deaths (all ages, both sexes) with 95% uncertainty intervals (2016)				Annualized rate of change of age-standardized rate (%) with 95% uncertainty intervals							
					1990-2006				2006-2016			
	**Drug-susceptible HIV-TB**	**MDR HIV-TB**	**XDR HIV-TB**	**All HIV-positive TB**	**Drug-susceptible HIV-TB**	**MDR HIV-TB**	**XDR HIV-TB**	**All HIV-positive TB**	**Drug-susceptible HIV-TB**	**MDR HIV-TB**	**XDR HIV-TB**	**All HIV-positive TB**
Global	215,740 (148,684 to 288,554)	18,375 (11,208 to 27,747)	1,151 (689 to 1,802)	235,267 (162,511 to 312,333)	8.7 (7.8 to 9.6)	25.5 (23.8 to 27.1)	30.0 (27.0 to 32.6)	9.2 (8.3 to 10.1)	-8.9 (-9.5 to -8.4)	-9.1 (-11.2 to -7.2)	2.3 (0.9 to 3.7)	-8.9 (-9.5 to -8.4)
Afghanistan	11 (2-25)	3 (0-9)	0 (0-1)	14 (2 to 30)	0.8 (-3.8 to 11.8)	32.4 (22.7 to 51.2)	12.5 (1.9 to 19.1)	2.2 (-2.1 to 13.2)	-5.8 (-20.4 to 0.9)	-5.5 (-27.0 to 9.9)	2.5 (-19.0 to 17.9)	-5.6 (-19.7 to -0.6)
Albania	0 (0-1)	0 (0-0)	0 (0-0)	0 (0 to 1)	-0.9 (-4.3 to 2.6)	15.3 (2.4 to 29.9)	0.4 (-11.1 to 7.9)	-0.7 (-4.0 to 2.9)	-4.9 (-6.3 to -3.2)	-7.6 (-26.0 to 10.0)	3.6 (-14.9 to 21.1)	-4.8 (-6.1 to -3.4)
Algeria	12 (4-22)	1 (0-4)	0 (0-0)	13 (5 to 24)	2.8 (-1.0 to 6.7)	5.6 (-5.7 to 16.2)	3.6 (-6.4 to 10.5)	3.0 (-0.7 to 6.9)	-8.7 (-11.3 to -7.0)	-7.0 (-24.9 to 6.3)	1.1 (-16.8 to 14.3)	-8.6 (-10.1 to -7.2)
American Samoa	0 (0-0)	0 (0-0)	0 (0-0)	0 (0 to 0)	0.6 (-2.1 to 12.7)	9.5 (-17.2 to 40.6)	0.5 (-31.8 to 12.4)	0.8 (-1.4 to 13.0)	3.0 (0.6 to 5.4)	0.7 (-34.7 to 31.8)	12.4 (-23.0 to 43.6)	3.0 (1.4 to 4.1)
Andorra	0 (0-0)	0 (0-0)	0 (0-0)	0 (0 to 0)	-7.9 (-9.3 to -6.3)	-8.4 (-12.3 to -4.8)	-19.6 (-25.3 to -14.4)	-7.9 (-9.3 to -6.3)	-0.9 (-2.4 to 0.4)	-2.3 (-6.9 to 2.5)	5.2 (0.5 to 9.9)	-0.9 (-2.4 to 0.4)
Angola	1,960 (1,167-3,136)	54 (22-111)	1 (0-2)	2,015 (1,208 to 3,232)	9.8 (3.6 to 17.8)	19.0 (11.5 to 28.3)	22.4 (17.4 to 26.3)	9.9 (3.7 to 18.0)	-7.6 (-9.1 to -5.6)	-7.9 (-13.5 to -2.1)	3.2 (-2.3 to 9.1)	-7.6 (-9.2 to -5.5)
Antigua and Barbuda	0 (0-0)	0 (0-0)	0 (0-0)	0 (0 to 0)	1.6 (0.6 to 2.4)	7.4 (-7.5 to 20.5)	10.7 (-4.0 to 19.1)	1.7 (0.9 to 2.4)	-6.9 (-7.9 to -5.9)	-11.4 (-27.5 to 5.4)	-1.8 (-17.9 to 15.0)	-7.0 (-7.9 to -6.1)
Argentina	633 (453-723)	49 (8-159)	16 (3-53)	699 (529 to 756)	4.3 (3.3 to 4.8)	17.3 (8.2 to 30.8)	34.4 (28.9 to 38.7)	4.8 (4.3 to 5.1)	-1.9 (-4.5 to -0.4)	-3.2 (-19.7 to 8.0)	4.2 (-12.2 to 15.4)	-1.9 (-3.7 to -1.1)
Armenia	4 (2-7)	1 (0-3)	1 (0-2)	6 (4 to 10)	10.7 (9.1 to 11.9)	42.4 (34.2 to 55.5)	28.5 (25.6 to 31.0)	12.8 (11.7 to 13.8)	-0.5 (-8.4 to 5.2)	-2.1 (-15.7 to 7.2)	9.1 (-4.6 to 18.4)	-0.1 (-4.3 to 4.2)
Australia	3 (2-5)	0 (0-0)	0 (0-0)	4 (2 to 6)	-8.2 (-9.3 to -7.3)	3.3 (-6.6 to 13.4)	5.7 (-3.6 to 12.3)	-7.8 (-8.6 to -7.0)	-6.4 (-8.0 to -4.8)	-6.6 (-17.7 to 6.7)	0.9 (-10.2 to 14.1)	-6.3 (-7.5 to -5.2)
Austria	4 (2-6)	0 (0-1)	0 (0-0)	4 (2 to 7)	-10.9 (-11.7 to -9.8)	-1.0 (-3.9 to 2.2)	11.1 (6.6 to 15.0)	-10.5 (-11.2 to -9.3)	-2.5 (-3.6 to -1.3)	-0.6 (-4.0 to 2.8)	6.9 (3.5 to 10.2)	-2.1 (-3.2 to -1.0)
Azerbaijan	5 (2-9)	3 (1-5)	1 (1-3)	9 (5 to 14)	5.1 (-0.7 to 15.1)	43.6 (34.4 to 61.4)	34.1 (27.1 to 38.5)	8.5 (5.9 to 18.4)	-15.1 (-22.3 to -5.9)	-17.0 (-25.9 to -5.3)	-5.9 (-14.7 to 5.8)	-14.7 (-16.8 to -11.6)
Bahrain	1 (0-1)	0 (0-0)	0 (0-0)	1 (0 to 1)	-3.0 (-4.1 to -2.0)	19.7 (9.0 to 34.5)	3.6 (-6.3 to 10.6)	-2.6 (-3.4 to -1.7)	-6.5 (-8.7 to -4.7)	-3.7 (-19.8 to 14.3)	4.4 (-11.8 to 22.3)	-6.3 (-7.2 to -5.2)
Bangladesh	105 (3-384)	9 (0-34)	1 (0-2)	114 (3 to 414)	35.5 (20.5 to 43.2)	20.1 (5.2 to 28.1)	-3.0 (-18.0 to 5.1)	36.0 (21.1 to 43.7)	7.7 (-11.8 to 14.6)	7.4 (-12.1 to 14.6)	17.0 (-2.5 to 24.1)	7.7 (-11.8 to 14.6)

	Number of deaths (all ages, both sexes) with 95% uncertainty intervals (2016)				Annualized rate of change of age-standardized rate (%) with 95% uncertainty intervals							
					1990-2006				2006-2016			
	**Drug-susceptible HIV-TB**	**MDR HIV-TB**	**XDR HIV-TB**	**All HIV-positive TB**	**Drug-susceptible HIV-TB**	**MDR HIV-TB**	**XDR HIV-TB**	**All HIV-positive TB**	**Drug-susceptible HIV-TB**	**MDR HIV-TB**	**XDR HIV-TB**	**All HIV-positive TB**
Barbados	1 (0-1)	0 (0-0)	0 (0-0)	1 (0 to 1)	1.4 (0.8 to 2.1)	3.0 (-10.5 to 16.3)	-6.3 (-21.4 to 3.0)	1.4 (0.8 to 2.1)	-3.6 (-4.5 to -2.7)	-6.0 (-22.1 to 11.2)	3.7 (-12.5 to 20.9)	-3.6 (-4.5 to -2.7)
Belarus	7 (4-13)	13 (8-21)	7 (4-12)	28 (16 to 45)	1.8 (-1.4 to 4.1)	48.6 (44.6 to 53.5)	38.3 (34.6 to 41.6)	8.3 (7.4 to 9.1)	-8.0 (-12.0 to -3.6)	-7.0 (-9.1 to -4.2)	4.2 (2.0 to 6.9)	-5.3 (-6.7 to -3.5)
Belgium	9 (5-14)	0 (0-1)	0 (0-0)	9 (5 to 15)	-6.1 (-6.9 to -5.3)	-0.4 (-3.1 to 2.3)	14.4 (10.1 to 18.1)	-5.8 (-6.5 to -5.0)	-3.9 (-4.9 to -2.9)	-5.4 (-9.2 to -2.2)	2.0 (-1.8 to 5.2)	-3.9 (-4.8 to -2.9)
Belize	6 (4-10)	0 (0-0)	0 (0-0)	6 (4 to 11)	5.8 (4.6 to 6.8)	12.0 (-2.3 to 25.4)	26.7 (11.8 to 35.9)	5.9 (4.9 to 6.9)	-5.6 (-6.6 to -4.4)	-10.6 (-27.8 to 6.0)	-1.0 (-18.2 to 15.6)	-5.7 (-6.6 to -4.6)
Benin	387 (221-643)	14 (4-37)	0 (0-1)	401 (230 to 668)	8.5 (4.9 to 17.1)	19.9 (11.6 to 30.7)	19.3 (11.5 to 24.7)	8.6 (5.0 to 17.2)	-11.3 (-12.8 to -9.7)	-10.0 (-20.3 to -0.0)	1.1 (-9.1 to 11.1)	-11.2 (-12.7 to -9.7)
Bermuda	0 (0-0)	0 (0-0)	0 (0-0)	0 (0 to 0)	-2.2 (-2.9 to -1.5)	-1.2 (-13.8 to 12.5)	-13.1 (-26.6 to -4.5)	-2.2 (-2.9 to -1.5)	-9.2 (-10.0 to -8.2)	-11.2 (-27.8 to 5.3)	-1.6 (-18.2 to 14.9)	-9.2 (-10.1 to -8.2)
Bhutan	0 (0-1)	0 (0-0)	0 (0-0)	1 (0 to 1)	0.3 (-3.0 to 12.2)	17.3 (13.4 to 29.4)	4.7 (-0.8 to 9.6)	0.9 (-2.5 to 12.9)	-2.7 (-4.1 to -1.2)	-3.2 (-5.9 to -0.5)	6.3 (3.7 to 9.1)	-2.7 (-4.1 to -1.2)
Bolivia	38 (14-67)	4 (1-11)	1 (0-2)	42 (15 to 73)	-0.5 (-6.2 to 12.8)	11.8 (1.0 to 28.5)	28.1 (18.2 to 34.9)	0.2 (-5.4 to 13.6)	-9.3 (-16.5 to -6.5)	-11.5 (-24.5 to -0.6)	-1.9 (-14.8 to 9.0)	-9.4 (-16.6 to -7.1)
Bosnia and Herzegovina	1 (1-2)	0 (0-0)	0 (0-0)	1 (1 to 2)	0.7 (-1.5 to 3.2)	5.4 (-3.7 to 19.5)	-1.0 (-5.8 to 3.0)	0.7 (-1.5 to 3.3)	-2.7 (-4.9 to -1.0)	-6.7 (-20.5 to 4.3)	4.5 (-9.4 to 15.5)	-2.7 (-4.9 to -1.0)
Botswana	1,206 (680-1,894)	79 (22-189)	1 (0-3)	1,286 (719 to 2,001)	13.5 (11.2 to 16.7)	26.1 (19.1 to 35.2)	42.0 (37.2 to 45.7)	13.8 (11.6 to 17.0)	-15.0 (-17.1 to -12.9)	-13.1 (-24.3 to -4.5)	-1.9 (-13.1 to 6.7)	-14.8 (-16.8 to -12.9)
Brazil	1,805 (1,078-2,906)	93 (52-161)	19 (11-34)	1,917 (1,147 to 3,081)	-1.6 (-2.1 to -0.7)	23.0 (22.1 to 24.1)	24.9 (21.2 to 28.2)	-1.2 (-1.7 to -0.3)	-3.1 (-3.5 to -2.7)	-4.8 (-5.6 to -4.0)	4.8 (4.0 to 5.7)	-3.2 (-3.5 to -2.7)
Brunei	0 (0-0)	0 (0-0)	0 (0-0)	0 (0 to 0)	-2.0 (-2.7 to -1.3)	2.6 (-0.8 to 6.1)	-14.5 (-20.1 to -9.9)	-2.0 (-2.7 to -1.3)	-1.5 (-2.3 to -0.7)	4.0 (-0.6 to 8.7)	11.4 (6.8 to 16.2)	-1.5 (-2.3 to -0.6)
Bulgaria	0 (0-1)	0 (0-0)	0 (0-0)	1 (0 to 1)	3.8 (2.6 to 4.8)	26.6 (17.1 to 41.2)	5.1 (-4.1 to 11.3)	4.4 (3.6 to 5.2)	-7.7 (-10.0 to -5.4)	-10.0 (-25.9 to 7.0)	1.1 (-14.7 to 18.2)	-7.7 (-8.9 to -6.1)
Burkina Faso	1,036 (719-1,370)	61 (17-157)	1 (0-3)	1,098 (767 to 1,453)	-1.5 (-3.4 to 1.8)	12.5 (4.7 to 20.7)	29.3 (20.5 to 35.3)	-1.1 (-3.0 to 2.2)	-13.5 (-16.0 to -10.8)	-15.3 (-24.2 to -5.3)	-4.2 (-13.0 to 5.9)	-13.6 (-16.1 to -10.9)
Burundi	1,192 (916-1,554)	90 (24-223)	2 (0-4)	1,283 (1,006 to 1,667)	7.2 (2.5 to 13.8)	27.5 (18.6 to 38.5)	33.4 (25.1 to 38.8)	7.6 (3.0 to 14.2)	-14.9 (-17.8 to -11.5)	-13.1 (-23.8 to -2.6)	-2.0 (-12.7 to 8.6)	-14.7 (-17.4 to -11.6)

	Number of deaths (all ages, both sexes) with 95% uncertainty intervals (2016)				Annualized rate of change of age-standardized rate (%) with 95% uncertainty intervals							
					1990-2006				2006-2016			
	**Drug-susceptible HIV-TB**	**MDR HIV-TB**	**XDR HIV-TB**	**All HIV-positive TB**	**Drug-susceptible HIV-TB**	**MDR HIV-TB**	**XDR HIV-TB**	**All HIV-positive TB**	**Drug-susceptible HIV-TB**	**MDR HIV-TB**	**XDR HIV-TB**	**All HIV-positive TB**
Cambodia	212 (121-338)	10 (1-34)	2 (0-8)	225 (132 to 362)	36.1 (28.7 to 46.0)	57.6 (45.7 to 77.1)	36.4 (29.7 to 41.7)	36.4 (29.0 to 46.3)	-16.3 (-18.8 to -14.0)	-18.3 (-35.4 to -4.6)	-6.6 (-23.7 to 7.2)	-16.3 (-18.3 to -13.9)
Cameroon	3,843 (1,795-6,851)	215 (45-583)	4 (1-10)	4,062 (1,917 to 7,259)	8.6 (6.4 to 13.3)	22.1 (13.9 to 31.5)	31.2 (21.9 to 37.7)	9.0 (6.8 to 13.6)	-5.9 (-7.2 to -4.7)	-7.6 (-17.6 to 2.3)	3.6 (-6.5 to 13.4)	-6.0 (-7.0 to -5.0)
Canada	22 (13-36)	1 (0-1)	0 (0-0)	23 (13 to 38)	-7.8 (-8.5 to -7.1)	-6.6 (-8.1 to -5.1)	9.5 (5.4 to 13.1)	-7.7 (-8.5 to -7.1)	-5.4 (-6.6 to -4.3)	-6.2 (-8.2 to -4.2)	1.2 (-0.8 to 3.2)	-5.4 (-6.6 to -4.3)
Cape Verde	5 (3-9)	0 (0-1)	0 (0-0)	5 (3 to 9)	-1.4 (-3.3 to 0.3)	12.4 (4.4 to 20.6)	13.8 (5.1 to 20.1)	-1.1 (-2.9 to 0.6)	-8.5 (-10.6 to -6.9)	-10.2 (-20.2 to 0.4)	1.0 (-9.0 to 11.6)	-8.6 (-10.5 to -7.1)
Central African Republic	3,284 (2,644-4,053)	66 (33-118)	1 (1-2)	3,352 (2,692 to 4,145)	9.5 (6.9 to 12.5)	17.1 (12.3 to 22.8)	33.4 (29.0 to 37.1)	9.6 (7.0 to 12.6)	-7.1 (-8.3 to -5.7)	-7.8 (-13.1 to -2.5)	3.3 (-1.9 to 8.6)	-7.1 (-8.3 to -5.7)
Chad	1,381 (736-2,438)	82 (18-222)	1 (0-4)	1,464 (795 to 2,579)	8.2 (5.3 to 12.5)	22.2 (13.7 to 31.2)	30.1 (20.7 to 36.5)	8.6 (5.8 to 12.9)	-8.4 (-9.8 to -7.0)	-10.4 (-20.2 to 0.3)	0.8 (-9.0 to 11.5)	-8.6 (-9.7 to -7.2)
Chile	201 (173-215)	5 (2-10)	2 (1-3)	208 (181 to 222)	4.7 (4.2 to 5.1)	12.6 (1.1 to 25.0)	24.0 (15.5 to 29.8)	4.8 (4.4 to 5.2)	-0.8 (-2.3 to 0.1)	-1.4 (-13.6 to 12.0)	6.0 (-6.1 to 19.5)	-0.8 (-2.3 to 0.0)
China	2,129 (1,267-3,397)	310 (168-538)	75 (40-131)	2,514 (1,511 to 4,025)	10.2 (7.3 to 13.6)	18.7 (15.4 to 22.5)	20.6 (16.8 to 23.9)	11.1 (8.2 to 14.5)	-3.5 (-4.4 to -2.5)	-6.3 (-8.1 to -4.6)	5.4 (3.6 to 7.2)	-3.7 (-4.6 to -2.8)
Colombia	157 (93-252)	13 (5-28)	3 (1-6)	173 (103 to 286)	0.7 (-0.3 to 2.0)	18.6 (14.5 to 23.4)	24.0 (19.5 to 27.9)	1.4 (0.4 to 2.6)	-5.8 (-6.9 to -4.7)	-8.0 (-13.7 to -2.9)	1.6 (-4.1 to 6.7)	-5.9 (-6.7 to -4.8)
Comoros	5 (0-10)	0 (0-1)	0 (0-0)	5 (0 to 11)	24.6 (15.6 to 45.3)	45.3 (33.3 to 68.4)	7.3 (-8.5 to 14.6)	25.0 (15.8 to 45.6)	-1.7 (-15.2 to 5.9)	-0.7 (-16.6 to 10.9)	10.5 (-5.4 to 22.1)	-1.6 (-14.9 to 6.0)
Congo (Brazzaville)	820 (436-1,349)	23 (9-47)	0 (0-1)	843 (448 to 1,379)	0.1 (-3.0 to 3.8)	9.5 (4.3 to 15.0)	27.2 (21.4 to 31.8)	0.2 (-2.8 to 4.0)	-7.8 (-8.9 to -6.8)	-8.1 (-13.4 to -2.9)	3.0 (-2.3 to 8.2)	-7.8 (-8.9 to -6.8)
Costa Rica	7 (4-12)	0 (0-1)	0 (0-0)	8 (5 to 13)	-2.6 (-3.4 to -1.9)	19.4 (14.7 to 24.3)	14.5 (9.1 to 18.8)	-2.3 (-3.0 to -1.6)	-4.7 (-5.5 to -3.8)	-5.8 (-11.4 to -0.4)	3.8 (-1.8 to 9.2)	-4.7 (-5.5 to -3.9)
Cote d'Ivoire	2,396 (1,163-4,328)	221 (58-584)	4 (1-10)	2,621 (1,308 to 4,609)	3.4 (2.1 to 4.8)	11.2 (4.9 to 17.6)	32.6 (25.6 to 38.0)	3.8 (2.6 to 5.1)	-9.2 (-10.8 to -7.9)	-9.6 (-18.8 to -1.7)	1.5 (-7.7 to 9.5)	-9.3 (-10.5 to -8.1)
Croatia	1 (1-2)	0 (0-0)	0 (0-0)	1 (1 to 2)	-0.7 (-4.0 to 2.3)	1.4 (-9.7 to 16.1)	1.4 (-5.7 to 6.6)	-0.7 (-3.9 to 2.3)	-9.2 (-10.4 to -7.8)	-15.9 (-33.0 to -3.0)	-4.7 (-21.8 to 8.1)	-9.2 (-10.5 to -7.9)
Cuba	5 (3-7)	0 (0-0)	0 (0-0)	5 (3 to 8)	-2.6 (-3.3 to -1.9)	-1.1 (-9.3 to 8.1)	-3.0 (-9.6 to 2.0)	-2.6 (-3.1 to -2.0)	4.5 (3.2 to 5.8)	5.0 (-8.5 to 17.1)	14.7 (1.1 to 26.7)	4.5 (3.5 to 5.7)
Cyprus	0 (0-1)	0 (0-0)	0 (0-0)	0 (0 to 1)	-0.6 (-1.8 to 1.2)	10.6 (7.0 to 14.1)	2.2 (-2.7 to 6.7)	-0.5 (-1.7 to 1.3)	-4.0 (-5.2 to -2.5)	-9.2 (-13.4 to -4.8)	-1.8 (-6.0 to 2.7)	-4.0 (-5.2 to -2.6)

	Number of deaths (all ages, both sexes) with 95% uncertainty intervals (2016)				Annualized rate of change of age-standardized rate (%) with 95% uncertainty intervals							
					1990-2006				2006-2016			
	**Drug-susceptible HIV-TB**	**MDR HIV-TB**	**XDR HIV-TB**	**All HIV-positive TB**	**Drug-susceptible HIV-TB**	**MDR HIV-TB**	**XDR HIV-TB**	**All HIV-positive TB**	**Drug-susceptible HIV-TB**	**MDR HIV-TB**	**XDR HIV-TB**	**All HIV-positive TB**
Czech Republic	3 (1-5)	0 (0-0)	0 (0-0)	3 (2 to 5)	-4.1 (-6.9 to -1.3)	1.3 (-7.6 to 13.7)	5.5 (0.3 to 9.5)	-3.8 (-6.5 to -1.0)	1.5 (-0.1 to 3.2)	-1.2 (-9.7 to 6.6)	9.9 (1.5 to 17.7)	1.5 (0.2 to 3.0)
Democratic Republic of the Congo	9,880 (7,091-12,424)	275 (124-506)	5 (2-9)	10,160 (7,298 to 12,809)	2.7 (1.1 to 4.8)	12.2 (7.5 to 16.6)	26.5 (21.7 to 30.4)	2.9 (1.2 to 4.9)	-9.7 (-11.0 to -8.3)	-10.0 (-15.3 to -4.3)	1.2 (-4.2 to 6.8)	-9.7 (-11.0 to -8.4)
Denmark	2 (1-3)	0 (0-0)	0 (0-0)	2 (1 to 3)	-7.2 (-8.0 to -6.5)	0.3 (-2.7 to 3.3)	-0.2 (-5.1 to 3.9)	-7.2 (-7.9 to -6.5)	-6.0 (-7.2 to -4.9)	-1.9 (-6.2 to 2.2)	5.5 (1.3 to 9.6)	-5.9 (-7.1 to -4.8)
Djibouti	97 (51-163)	15 (6-32)	0 (0-1)	113 (60 to 190)	19.4 (15.8 to 27.9)	52.4 (43.9 to 62.8)	31.7 (23.6 to 37.5)	20.3 (16.7 to 28.7)	-4.7 (-6.5 to -2.8)	-4.3 (-12.8 to 5.6)	6.8 (-1.6 to 16.8)	-4.6 (-5.9 to -3.6)
Dominica	1 (0-1)	0 (0-0)	0 (0-0)	1 (0 to 1)	3.0 (2.1 to 3.9)	9.1 (-5.5 to 22.8)	20.9 (5.8 to 29.6)	3.1 (2.3 to 3.9)	-3.1 (-4.6 to -1.8)	-7.8 (-25.0 to 9.3)	1.9 (-15.4 to 18.9)	-3.1 (-4.5 to -2.0)
Dominican Republic	133 (77-215)	2 (0-9)	0 (0-2)	136 (79 to 218)	3.8 (2.1 to 6.2)	9.7 (-3.7 to 22.6)	25.4 (10.7 to 33.6)	3.9 (2.2 to 6.3)	-7.0 (-8.1 to -5.8)	-11.5 (-27.5 to 6.0)	-1.9 (-17.9 to 15.6)	-7.0 (-8.0 to -6.1)
Ecuador	187 (101-304)	33 (8-86)	7 (2-18)	227 (132 to 367)	9.6 (6.8 to 11.2)	29.1 (21.2 to 38.3)	35.6 (30.8 to 39.3)	10.8 (8.0 to 12.0)	-4.2 (-7.5 to -0.9)	-5.8 (-15.7 to 1.7)	3.9 (-6.0 to 11.4)	-4.3 (-5.8 to -1.2)
Egypt	11 (5-19)	2 (0-5)	0 (0-0)	13 (7 to 21)	-1.0 (-4.0 to 4.1)	18.6 (8.0 to 33.2)	0.5 (-7.0 to 5.7)	-0.3 (-3.2 to 4.9)	-5.6 (-8.9 to -3.4)	-2.5 (-17.3 to 9.6)	5.5 (-9.3 to 17.6)	-5.1 (-6.3 to -4.0)
El Salvador	11 (6-18)	0 (0-1)	0 (0-0)	12 (7 to 19)	-3.5 (-5.6 to -0.7)	11.6 (5.9 to 17.0)	18.2 (12.0 to 23.0)	-3.2 (-5.2 to -0.4)	-8.7 (-9.9 to -7.5)	-10.2 (-16.6 to -3.4)	-0.6 (-6.9 to 6.2)	-8.7 (-9.8 to -7.6)
Equatorial Guinea	102 (52-176)	3 (1-6)	0 (0-0)	105 (53 to 180)	9.7 (2.6 to 16.6)	18.9 (10.9 to 26.9)	24.1 (17.8 to 28.7)	9.9 (2.7 to 16.6)	-7.1 (-8.1 to -6.0)	-7.4 (-12.5 to -2.3)	3.8 (-1.4 to 8.8)	-7.1 (-8.1 to -6.0)
Eritrea	288 (153-433)	23 (5-59)	0 (0-1)	311 (164 to 465)	7.4 (4.2 to 14.6)	27.7 (18.3 to 38.3)	24.3 (15.2 to 30.8)	7.8 (4.7 to 15.0)	-6.7 (-10.2 to -4.5)	-5.7 (-16.3 to 4.5)	5.4 (-5.1 to 15.7)	-6.6 (-9.7 to -4.7)
Estonia	3 (2-5)	2 (1-3)	1 (1-2)	6 (3 to 9)	20.5 (17.1 to 23.2)	25.6 (21.7 to 29.1)	41.3 (37.8 to 44.0)	22.5 (19.3 to 24.9)	-10.3 (-13.0 to -7.5)	-11.4 (-14.0 to -8.8)	-0.3 (-2.9 to 2.3)	-9.5 (-11.2 to -7.4)
Ethiopia	6,691 (4,384-8,992)	523 (142-1,292)	9 (2-22)	7,224 (4,813 to 9,530)	9.9 (5.8 to 13.4)	32.9 (25.2 to 42.0)	32.7 (27.3 to 36.7)	10.3 (6.2 to 13.8)	-18.1 (-19.9 to -16.3)	-16.7 (-26.8 to -8.6)	-5.5 (-15.7 to 2.6)	-18.0 (-19.5 to -16.3)
Federated States of Micronesia	1 (0-1)	0 (0-0)	0 (0-0)	1 (0 to 1)	4.8 (-0.5 to 22.6)	12.8 (-11.2 to 47.9)	13.8 (-18.9 to 25.8)	5.0 (-0.1 to 22.7)	12.0 (7.9 to 22.0)	8.2 (-26.1 to 41.9)	19.9 (-14.4 to 53.7)	11.9 (8.2 to 21.1)
Fiji	1 (1-2)	0 (0-0)	0 (0-0)	1 (1 to 2)	7.0 (4.9 to 11.8)	10.2 (-15.3 to 38.4)	-1.4 (-35.9 to 10.9)	7.0 (4.9 to 11.8)	-3.2 (-4.5 to -1.9)	-5.9 (-40.5 to 30.9)	5.7 (-28.8 to 42.7)	-3.2 (-4.4 to -2.1)
Finland	2 (1-3)	0 (0-0)	0 (0-0)	2 (1 to 3)	-2.6 (-5.4 to -0.3)	10.2 (5.9 to 14.3)	6.5 (1.6 to 10.4)	-2.4 (-5.2 to -0.1)	-9.3 (-10.8 to -8.0)	-2.0 (-5.9 to 1.8)	5.5 (1.6 to 9.2)	-8.9 (-10.3 to -7.6)

	Number of deaths (all ages, both sexes) with 95% uncertainty intervals (2016)				Annualized rate of change of age-standardized rate (%) with 95% uncertainty intervals							
					1990-2006				2006-2016			
	Drug-susceptible HIV-TB	MDR HIV-TB	XDR HIV-TB	All HIV-positive TB	Drug-susceptible HIV-TB	MDR HIV-TB	XDR HIV-TB	All HIV-positive TB	Drug-susceptible HIV-TB	MDR HIV-TB	XDR HIV-TB	All HIV-positive TB
France	140 (81-193)	6 (3-11)	2 (1-4)	148 (86 to 205)	-9.0 (-9.9 to -8.1)	-2.9 (-5.6 to -0.3)	21.5 (17.7 to 24.7)	-8.7 (-9.7 to -7.8)	-7.7 (-9.0 to -6.3)	-8.1 (-11.4 to -5.1)	-0.7 (-4.0 to 2.3)	-7.7 (-8.9 to -6.2)
Gabon	232 (129-375)	6 (2-12)	0 (0-0)	239 (134 to 385)	9.9 (5.1 to 14.6)	18.9 (12.8 to 25.1)	26.7 (20.7 to 31.1)	10.0 (5.3 to 14.8)	-10.1 (-11.3 to -8.4)	-10.3 (-15.4 to -4.9)	0.9 (-4.3 to 6.2)	-10.1 (-11.3 to -8.4)
Georgia	6 (3-9)	3 (2-5)	2 (1-3)	11 (6 to 16)	5.0 (3.2 to 6.2)	32.7 (24.9 to 45.4)	20.5 (17.5 to 23.0)	6.6 (4.8 to 7.6)	8.6 (4.5 to 12.7)	15.8 (11.1 to 20.5)	26.9 (22.2 to 31.7)	11.8 (8.6 to 15.4)
Germany	37 (21-60)	3 (1-5)	1 (0-2)	40 (23 to 66)	-12.7 (-13.5 to -11.9)	-7.4 (-9.8 to -4.7)	11.0 (6.8 to 14.7)	-12.5 (-13.2 to -11.6)	-4.8 (-5.8 to -3.8)	-3.2 (-6.3 to -0.5)	4.2 (1.2 to 7.0)	-4.5 (-5.5 to -3.6)
Ghana	2,011 (1,142-3,288)	112 (29-324)	2 (0-6)	2,125 (1,221 to 3,503)	3.2 (-0.5 to 8.8)	16.5 (7.5 to 26.6)	28.2 (19.7 to 34.6)	3.6 (-0.1 to 9.1)	-10.4 (-11.8 to -9.0)	-12.1 (-22.4 to -1.4)	-1.0 (-11.2 to 9.7)	-10.5 (-11.8 to -9.3)
Greece	8 (7-9)	1 (1-1)	0 (0-0)	9 (9 to 10)	-7.7 (-9.2 to -7.1)	15.6 (12.9 to 18.8)	14.4 (11.3 to 17.0)	-6.9 (-8.3 to -6.3)	2.8 (1.8 to 4.5)	1.0 (-3.0 to 4.8)	8.5 (4.5 to 12.2)	2.8 (1.9 to 4.4)
Greenland	0 (0-0)	0 (0-0)	0 (0-0)	0 (0 to 1)	-4.3 (-6.4 to -1.6)	-2.6 (-5.2 to 0.6)	25.9 (22.0 to 29.2)	-4.3 (-6.4 to -1.6)	-6.9 (-8.5 to -5.2)	-7.7 (-10.1 to -5.4)	-0.3 (-2.6 to 2.0)	-6.9 (-8.5 to -5.2)
Grenada	0 (0-0)	0 (0-0)	0 (0-0)	0 (0 to 0)	3.6 (2.6 to 4.5)	9.7 (-3.8 to 23.7)	12.6 (-2.8 to 22.1)	3.8 (3.0 to 4.6)	-5.3 (-6.3 to -4.2)	-10.2 (-27.6 to 5.5)	-0.5 (-18.0 to 15.2)	-5.4 (-6.2 to -4.5)
Guam	0 (0-0)	0 (0-0)	0 (0-0)	0 (0 to 1)	3.3 (0.1 to 17.3)	17.8 (2.7 to 50.0)	11.2 (0.5 to 18.0)	3.8 (0.9 to 17.9)	8.0 (2.5 to 11.2)	-1.1 (-34.9 to 20.5)	10.7 (-23.1 to 32.3)	7.5 (2.7 to 9.3)
Guatemala	108 (64-172)	10 (4-20)	2 (1-4)	120 (71 to 190)	-1.0 (-4.2 to 1.9)	19.0 (13.7 to 24.8)	31.2 (26.2 to 35.5)	-0.4 (-3.5 to 2.6)	-8.1 (-9.3 to -7.2)	-9.8 (-15.3 to -4.5)	-0.2 (-5.7 to 5.1)	-8.2 (-9.1 to -7.3)
Guinea	1,674 (1,010-2,379)	96 (23-265)	2 (0-4)	1,771 (1,070 to 2,509)	13.8 (8.5 to 18.3)	27.3 (17.7 to 36.8)	28.0 (20.3 to 33.8)	14.2 (8.8 to 18.7)	-3.2 (-4.7 to -1.7)	-5.0 (-15.6 to 4.8)	6.2 (-4.4 to 16.0)	-3.3 (-4.6 to -2.0)
Guinea-Bissau	705 (464-900)	39 (10-93)	1 (0-2)	744 (492 to 943)	18.0 (11.9 to 24.8)	31.2 (22.5 to 42.8)	33.7 (25.5 to 39.1)	18.3 (12.4 to 25.1)	-2.5 (-4.7 to 1.2)	-4.4 (-14.7 to 5.5)	6.7 (-3.5 to 16.7)	-2.6 (-4.6 to 0.9)
Guyana	25 (15-41)	0 (0-2)	0 (0-0)	26 (15 to 42)	7.6 (6.4 to 8.6)	13.1 (-0.1 to 26.8)	31.5 (17.4 to 40.3)	7.7 (6.7 to 8.6)	-7.0 (-8.2 to -5.6)	-11.5 (-27.7 to 5.6)	-1.9 (-18.1 to 15.2)	-7.1 (-8.2 to -5.8)
Haiti	345 (183-569)	6 (1-23)	1 (0-5)	353 (190 to 581)	0.8 (-0.8 to 2.8)	6.2 (-6.8 to 18.9)	34.6 (20.7 to 42.9)	0.9 (-0.7 to 2.8)	-12.3 (-13.8 to -10.7)	-16.6 (-33.8 to -0.1)	-7.0 (-24.2 to 9.6)	-12.4 (-13.8 to -10.9)
Honduras	74 (42-117)	4 (2-8)	1 (0-2)	79 (45 to 124)	-0.7 (-2.9 to 2.4)	19.7 (14.8 to 24.9)	27.2 (22.0 to 31.4)	-0.3 (-2.5 to 2.8)	-5.0 (-6.3 to -3.7)	-6.0 (-11.5 to -0.6)	3.6 (-1.9 to 9.0)	-5.0 (-6.2 to -3.8)
Hungary	2 (1-3)	0 (0-0)	0 (0-0)	2 (1 to 4)	-12.4 (-13.7 to -11.6)	10.1 (0.2 to 24.1)	14.0 (6.1 to 19.9)	-11.7 (-12.3 to -11.0)	-10.3 (-13.5 to -7.7)	-14.6 (-32.0 to 1.4)	-3.4 (-20.9 to 12.5)	-10.5 (-11.8 to -9.1)

	Number of deaths (all ages, both sexes) with 95% uncertainty intervals (2016)				Annualized rate of change of age-standardized rate (%) with 95% uncertainty intervals							
					1990-2006				2006-2016			
	Drug-susceptible HIV-TB	MDR HIV-TB	XDR HIV-TB	All HIV-positive TB	Drug-susceptible HIV-TB	MDR HIV-TB	XDR HIV-TB	All HIV-positive TB	Drug-susceptible HIV-TB	MDR HIV-TB	XDR HIV-TB	All HIV-positive TB
Iceland	0 (0-0)	0 (0-0)	0 (0-0)	0 (0 to 0)	-7.2 (-7.9 to -6.5)	-4.7 (-8.0 to -1.3)	-12.3 (-17.4 to -7.6)	-7.2 (-7.9 to -6.5)	-7.5 (-8.5 to -6.5)	-14.7 (-19.2 to -10.4)	-7.2 (-11.8 to -2.9)	-7.5 (-8.5 to -6.5)
India	16,637 (10,548-23,358)	2,462 (1,421-3,919)	159 (92-254)	19,258 (12,155 to 27,088)	12.8 (10.3 to 14.6)	39.9 (37.3 to 41.9)	30.8 (27.5 to 33.5)	13.7 (11.1 to 15.5)	-11.2 (-12.2 to -10.2)	-11.1 (-12.3 to -10.0)	-1.5 (-2.7 to -0.4)	-11.1 (-12.2 to -10.2)
Indonesia	3,267 (2,822-3,910)	51 (25-105)	12 (6-25)	3,330 (2,877 to 3,980)	54.6 (54.2 to 55.2)	33.4 (29.5 to 37.4)	17.2 (13.2 to 21.3)	54.8 (54.5 to 55.4)	6.5 (5.1 to 7.9)	-1.3 (-7.7 to 5.1)	10.5 (4.1 to 16.9)	6.3 (5.0 to 7.7)
Iran	92 (50-205)	3 (1-10)	0 (0-1)	95 (52 to 213)	13.4 (9.9 to 22.7)	36.4 (23.3 to 54.2)	8.1 (-3.9 to 15.6)	13.7 (10.4 to 23.0)	-6.7 (-13.2 to 1.7)	-8.5 (-25.8 to 12.8)	-0.5 (-17.7 to 20.8)	-6.8 (-13.1 to 1.8)
Iraq	1 (0-1)	0 (0-0)	0 (0-0)	1 (0 to 1)	2.4 (0.9 to 3.6)	27.4 (15.7 to 42.1)	-15.8 (-27.8 to -8.3)	2.8 (1.6 to 3.9)	-4.3 (-6.0 to -1.4)	-4.9 (-20.4 to 14.2)	3.2 (-12.3 to 22.3)	-4.3 (-5.7 to -1.5)
Ireland	4 (3-4)	0 (0-0)	0 (0-0)	4 (3 to 4)	-12.2 (-12.7 to -11.7)	-4.7 (-7.8 to -1.4)	1.7 (-1.5 to 4.4)	-12.0 (-12.5 to -11.5)	11.3 (8.3 to 12.4)	10.7 (6.5 to 14.8)	18.2 (13.9 to 22.3)	11.3 (8.4 to 12.4)
Israel	2 (1-3)	0 (0-1)	0 (0-0)	2 (1 to 4)	-6.5 (-7.2 to -5.7)	-1.7 (-4.4 to 1.1)	15.9 (11.5 to 19.7)	-5.8 (-6.5 to -5.2)	-6.7 (-8.1 to -5.6)	-8.1 (-11.2 to -5.0)	-0.7 (-3.8 to 2.4)	-6.7 (-7.8 to -5.6)
Italy	22 (13-37)	2 (1-4)	1 (0-1)	24 (14 to 41)	-8.3 (-9.0 to -7.6)	-3.4 (-5.7 to -0.7)	12.5 (8.4 to 16.1)	-7.9 (-8.5 to -7.2)	-4.0 (-5.2 to -2.9)	-6.2 (-9.4 to -3.1)	1.3 (-2.0 to 4.4)	-4.1 (-5.2 to -3.0)
Jamaica	6 (3-9)	0 (0-1)	0 (0-0)	6 (3 to 9)	3.0 (1.5 to 4.3)	27.1 (15.1 to 41.9)	20.4 (7.7 to 27.9)	3.4 (2.1 to 4.6)	-8.4 (-10.4 to -6.5)	-10.7 (-27.4 to 5.9)	-1.0 (-17.8 to 15.5)	-8.5 (-9.8 to -7.2)
Japan	52 (31-63)	2 (1-2)	1 (0-1)	54 (33 to 65)	0.2 (-1.0 to 1.3)	5.9 (4.3 to 7.2)	5.7 (1.8 to 8.7)	0.4 (-0.8 to 1.5)	-3.0 (-3.7 to -2.2)	-5.1 (-6.3 to -3.8)	2.4 (1.2 to 3.7)	-3.0 (-3.7 to -2.3)
Jordan	0 (0-0)	0 (0-0)	0 (0-0)	0 (0 to 0)	-1.4 (-3.3 to 0.2)	21.8 (12.8 to 37.0)	-3.8 (-9.8 to 1.1)	-0.1 (-1.5 to 1.2)	-7.0 (-12.4 to -3.8)	-6.8 (-23.0 to 4.7)	1.2 (-15.0 to 12.7)	-6.9 (-7.9 to -5.7)
Kazakhstan	9 (5-16)	8 (4-13)	5 (2-8)	22 (13 to 35)	3.1 (-0.5 to 5.7)	31.5 (23.8 to 43.0)	39.0 (34.9 to 42.5)	8.4 (7.5 to 9.3)	-15.6 (-20.1 to -10.2)	-18.8 (-22.4 to -14.9)	-7.7 (-11.3 to -3.8)	-15.8 (-17.0 to -14.5)
Kenya	6,713 (4,031-9,865)	272 (145-452)	5 (2-8)	6,989 (4,200 to 10,269)	9.6 (8.3 to 10.6)	34.2 (31.8 to 36.7)	29.1 (24.6 to 32.5)	9.8 (8.5 to 10.8)	-10.5 (-11.7 to -9.0)	-9.9 (-12.4 to -6.7)	1.3 (-1.3 to 4.4)	-10.5 (-11.6 to -9.0)
Kiribati	0 (0-0)	0 (0-0)	0 (0-0)	0 (0 to 0)	5.5 (1.4 to 10.2)	14.2 (-11.4 to 43.8)	14.4 (-16.8 to 26.7)	5.7 (2.0 to 10.3)	-6.0 (-9.6 to -1.7)	-8.4 (-44.4 to 25.7)	3.3 (-32.6 to 37.4)	-6.1 (-8.4 to -2.3)
Kuwait	0 (0-0)	0 (0-0)	0 (0-0)	0 (0 to 0)	-3.0 (-3.9 to -2.3)	20.0 (10.2 to 34.0)	-8.7 (-17.1 to -2.9)	-2.6 (-3.5 to -2.0)	-13.2 (-14.8 to -11.6)	-13.8 (-26.1 to -0.8)	-5.8 (-18.1 to 7.2)	-13.2 (-14.5 to -11.9)

	Number of deaths (all ages, both sexes) with 95% uncertainty intervals (2016)				Annualized rate of change of age-standardized rate (%) with 95% uncertainty intervals							
					1990-2006				2006-2016			
	**Drug-susceptible HIV-TB**	**MDR HIV-TB**	**XDR HIV-TB**	**All HIV-positive TB**	**Drug-susceptible HIV-TB**	**MDR HIV-TB**	**XDR HIV-TB**	**All HIV-positive TB**	**Drug-susceptible HIV-TB**	**MDR HIV-TB**	**XDR HIV-TB**	**All HIV-positive TB**
Kyrgyzstan	7 (2-14)	8 (4-14)	4 (2-8)	19 (11 to 30)	4.3 (-1.9 to 7.8)	47.1 (39.2 to 60.6)	40.1 (34.9 to 43.8)	10.1 (9.1 to 11.1)	-7.7 (-21.2 to 4.4)	-9.3 (-16.8 to -1.5)	1.8 (-5.6 to 9.6)	-6.9 (-7.9 to -5.8)
Laos	106 (2-536)	2 (0-13)	0 (0-3)	108 (2 to 542)	61.5 (32.7 to 72.5)	40.6 (6.6 to 53.1)	24.4 (-9.8 to 37.0)	61.7 (33.0 to 72.8)	-0.1 (-10.9 to 8.3)	-8.6 (-32.0 to 11.3)	3.1 (-20.3 to 23.0)	-0.3 (-11.0 to 7.8)
Latvia	7 (4-12)	2 (1-3)	1 (1-2)	10 (6 to 17)	4.1 (2.8 to 5.6)	6.3 (3.6 to 9.4)	37.4 (33.6 to 40.7)	5.0 (4.1 to 6.2)	-3.6 (-5.4 to -1.7)	-8.4 (-11.2 to -5.6)	2.7 (-0.1 to 5.5)	-4.2 (-5.6 to -2.5)
Lebanon	2 (1-3)	0 (0-1)	0 (0-0)	2 (1 to 4)	-10.0 (-15.3 to -1.6)	7.7 (-4.6 to 25.3)	2.9 (-11.0 to 10.1)	-9.6 (-14.8 to -1.3)	-6.6 (-10.1 to 1.5)	-5.1 (-23.0 to 10.4)	2.9 (-15.0 to 18.4)	-6.4 (-9.1 to 1.3)
Lesotho	3,992 (3,152-5,184)	486 (256-853)	8 (4-15)	4,486 (3,613 to 5,874)	23.7 (20.6 to 27.7)	53.0 (44.9 to 63.5)	49.5 (42.9 to 54.5)	24.4 (21.1 to 28.3)	-7.0 (-8.9 to -4.3)	-6.4 (-15.9 to 4.8)	4.8 (-4.8 to 16.0)	-7.0 (-8.4 to -4.7)
Liberia	128 (71-216)	8 (2-20)	0 (0-0)	136 (75 to 228)	6.6 (4.1 to 10.2)	20.1 (11.8 to 28.5)	22.3 (13.5 to 28.1)	6.9 (4.6 to 10.7)	-9.2 (-10.6 to -7.5)	-10.9 (-21.0 to -0.6)	0.2 (-9.8 to 10.6)	-9.3 (-10.6 to -7.9)
Libya	12 (2-26)	1 (0-4)	0 (0-0)	13 (2 to 28)	6.3 (0.5 to 15.5)	18.5 (3.4 to 37.2)	7.2 (-12.7 to 16.6)	6.5 (0.8 to 15.8)	1.2 (-6.4 to 6.8)	4.2 (-14.7 to 22.2)	12.2 (-6.7 to 30.2)	1.4 (-6.0 to 6.8)
Lithuania	3 (2-5)	1 (1-3)	1 (0-1)	6 (3 to 9)	5.7 (4.5 to 6.9)	12.4 (9.3 to 16.2)	32.1 (28.5 to 35.5)	7.6 (6.6 to 8.5)	-6.0 (-7.5 to -4.4)	-6.8 (-8.5 to -4.9)	4.3 (2.6 to 6.2)	-5.2 (-6.4 to -3.8)
Luxembourg	0 (0-0)	0 (0-0)	0 (0-0)	0 (0 to 0)	-9.1 (-9.9 to -8.2)	-3.5 (-6.8 to -0.3)	-9.8 (-15.0 to -5.3)	-9.0 (-9.9 to -8.2)	-6.2 (-7.3 to -5.0)	5.0 (0.6 to 9.8)	12.5 (8.1 to 17.2)	-6.1 (-7.2 to -4.9)
Macedonia	0 (0-1)	0 (0-0)	0 (0-0)	0 (0 to 1)	3.1 (-0.7 to 6.0)	20.3 (9.0 to 35.8)	0.9 (-10.9 to 8.4)	3.3 (-0.5 to 6.1)	-5.6 (-7.0 to -3.4)	-2.1 (-16.7 to 15.1)	9.0 (-5.6 to 26.2)	-5.3 (-6.7 to -3.1)
Madagascar	25 (15-41)	1 (0-2)	0 (0-0)	26 (15 to 42)	10.3 (6.6 to 27.3)	30.4 (22.0 to 49.4)	-7.4 (-14.3 to -2.1)	10.5 (6.8 to 27.5)	-3.7 (-4.9 to -2.5)	-2.5 (-13.1 to 6.3)	8.7 (-2.0 to 17.4)	-3.6 (-4.7 to -2.5)
Malawi	8,718 (5,017-12,579)	278 (86-615)	5 (1-11)	9,001 (5,192 to 13,004)	6.7 (5.1 to 8.6)	29.8 (23.0 to 38.3)	36.4 (29.7 to 41.6)	6.9 (5.3 to 8.9)	-11.7 (-13.1 to -10.3)	-11.3 (-22.5 to -0.9)	-0.1 (-11.3 to 10.3)	-11.7 (-13.0 to -10.3)
Malaysia	160 (93-253)	4 (1-9)	1 (0-2)	165 (96 to 259)	17.7 (12.4 to 22.9)	40.1 (28.3 to 54.4)	21.3 (9.8 to 28.9)	17.9 (12.7 to 23.1)	-6.9 (-8.3 to -5.3)	-11.7 (-26.7 to 7.0)	0.0 (-15.0 to 18.7)	-7.1 (-8.3 to -5.8)
Maldives	0 (0-0)	0 (0-0)	0 (0-0)	0 (0 to 0)	2.8 (-1.5 to 6.4)	16.3 (1.5 to 30.3)	3.6 (-12.7 to 13.5)	3.0 (-1.2 to 6.5)	-6.4 (-8.1 to -4.3)	-15.4 (-32.5 to 3.1)	-3.7 (-20.8 to 14.8)	-6.6 (-8.1 to -4.9)
Mali	423 (230-711)	25 (7-69)	0 (0-1)	448 (250 to 754)	1.2 (-3.0 to 9.0)	14.8 (5.2 to 25.9)	20.6 (12.1 to 26.6)	1.6 (-2.6 to 9.6)	-7.0 (-8.5 to -5.5)	-8.6 (-19.0 to 1.1)	2.5 (-7.8 to 12.3)	-7.1 (-8.3 to -5.8)
Malta	0 (0-0)	0 (0-0)	0 (0-0)	0 (0 to 0)	-6.0 (-6.7 to -5.4)	5.6 (2.3 to 8.7)	-9.7 (-14.7 to -5.5)	-6.0 (-6.7 to -5.4)	-4.2 (-5.3 to -3.1)	-20.3 (-24.6 to -15.6)	-12.8 (-17.2 to -8.1)	-4.3 (-5.3 to -3.1)

	Number of deaths (all ages, both sexes) with 95% uncertainty intervals (2016)				Annualized rate of change of age-standardized rate (%) with 95% uncertainty intervals							
					1990-2006				2006-2016			
	Drug-susceptible HIV-TB	MDR HIV-TB	XDR HIV-TB	All HIV-positive TB	Drug-susceptible HIV-TB	MDR HIV-TB	XDR HIV-TB	All HIV-positive TB	Drug-susceptible HIV-TB	MDR HIV-TB	XDR HIV-TB	All HIV-positive TB
Marshall Islands	0 (0-0)	0 (0-0)	0 (0-0)	0 (0 to 0)	5.1 (0.2 to 19.5)	25.8 (10.0 to 58.6)	15.6 (-1.7 to 24.1)	5.7 (2.1 to 20.1)	-0.8 (-4.6 to 6.5)	-6.1 (-33.9 to 24.3)	5.6 (-22.2 to 36.1)	-1.1 (-3.2 to 4.7)
Mauritania	39 (8-76)	2 (0-7)	0 (0-0)	42 (9 to 79)	1.3 (-2.6 to 13.8)	14.7 (5.5 to 30.8)	14.8 (4.8 to 21.3)	1.6 (-2.2 to 14.1)	-8.5 (-16.0 to -5.8)	-10.3 (-21.2 to -0.6)	0.9 (-10.1 to 10.6)	-8.6 (-16.2 to -6.1)
Mauritius	2 (1-3)	0 (0-0)	0 (0-0)	2 (1 to 3)	-3.2 (-4.0 to -2.5)	15.4 (4.1 to 28.8)	1.2 (-10.4 to 8.9)	-3.1 (-3.8 to -2.4)	4.6 (3.4 to 5.9)	1.5 (-15.7 to 17.9)	13.2 (-4.0 to 29.7)	4.5 (3.4 to 5.8)
Mexico	467 (278-747)	35 (19-61)	7 (4-13)	509 (305 to 819)	-2.9 (-4.5 to -1.2)	24.0 (21.8 to 26.1)	23.1 (19.1 to 26.7)	-2.3 (-3.9 to -0.6)	-4.9 (-5.4 to -4.3)	-6.3 (-8.1 to -4.6)	3.3 (1.6 to 5.0)	-4.9 (-5.4 to -4.3)
Moldova	4 (2-8)	6 (3-10)	3 (2-6)	13 (8 to 22)	5.4 (3.0 to 7.4)	43.1 (39.2 to 48.0)	40.8 (37.1 to 44.1)	10.6 (9.8 to 11.5)	-12.7 (-16.9 to -8.7)	-10.9 (-13.6 to -8.0)	0.2 (-2.4 to 3.1)	-9.8 (-11.2 to -7.7)
Mongolia	0 (0-1)	0 (0-0)	0 (0-0)	1 (0 to 1)	26.6 (9.3 to 33.4)	12.0 (-6.4 to 19.2)	1.5 (-16.8 to 8.6)	27.3 (10.0 to 34.0)	5.9 (-2.5 to 20.6)	7.0 (-9.9 to 24.4)	18.2 (1.2 to 35.5)	6.4 (-0.7 to 20.6)
Montenegro	0 (0-0)	0 (0-0)	0 (0-0)	0 (0 to 0)	2.1 (1.1 to 3.2)	9.4 (-3.8 to 24.6)	-10.6 (-25.0 to -2.1)	2.2 (1.1 to 3.3)	-4.5 (-5.8 to -2.7)	-8.7 (-29.3 to 10.5)	2.5 (-18.2 to 21.7)	-4.5 (-5.8 to -2.7)
Morocco	110 (5-453)	5 (0-25)	0 (0-2)	115 (6 to 485)	9.8 (-5.8 to 16.2)	34.2 (14.0 to 50.8)	16.3 (-11.6 to 26.9)	10.2 (-5.3 to 16.4)	-6.0 (-16.1 to 2.5)	-8.1 (-24.0 to 15.2)	-0.2 (-16.0 to 23.2)	-6.1 (-15.8 to 2.3)
Mozambique	16,839 (9,706-23,923)	2,259 (741-5,001)	39 (13-86)	19,137 (11,287 to 26,282)	12.6 (9.0 to 17.2)	30.5 (23.5 to 39.5)	43.6 (39.5 to 47.0)	13.4 (9.8 to 17.9)	-7.5 (-9.4 to -5.2)	-7.8 (-18.0 to -0.0)	3.4 (-6.8 to 11.1)	-7.5 (-9.0 to -5.4)
Myanmar	2,061 (1,458-2,877)	319 (124-623)	77 (30-151)	2,457 (1,794 to 3,370)	29.8 (20.5 to 40.5)	50.1 (38.3 to 69.5)	44.2 (39.6 to 47.6)	30.8 (21.6 to 41.3)	-10.4 (-14.2 to -6.2)	-10.2 (-20.6 to -1.2)	1.5 (-8.8 to 10.6)	-10.2 (-13.4 to -5.9)
Namibia	916 (513-1,450)	146 (68-256)	2 (1-4)	1,065 (599 to 1,660)	15.6 (11.9 to 19.9)	46.5 (38.1 to 57.0)	46.0 (38.3 to 51.0)	16.5 (13.0 to 20.8)	-14.0 (-16.3 to -11.1)	-13.6 (-22.8 to -2.4)	-2.5 (-11.6 to 8.8)	-13.9 (-15.8 to -11.5)
Nepal	82 (17-166)	8 (2-17)	1 (0-1)	90 (19 to 188)	56.0 (49.2 to 59.9)	41.2 (34.3 to 45.6)	18.1 (11.0 to 22.5)	56.6 (49.8 to 60.4)	-9.0 (-18.7 to -6.1)	-8.8 (-18.8 to -4.9)	0.7 (-9.2 to 4.7)	-9.0 (-18.6 to -6.0)
Netherlands	12 (6-19)	1 (0-1)	0 (0-0)	12 (7 to 21)	-10.5 (-11.5 to -9.4)	-5.2 (-7.9 to -2.0)	9.8 (5.5 to 13.0)	-10.4 (-11.4 to -9.3)	-5.1 (-6.4 to -2.9)	-1.5 (-5.3 to 2.3)	6.0 (2.2 to 9.7)	-4.9 (-6.1 to -2.7)
New Zealand	1 (0-1)	0 (0-0)	0 (0-0)	1 (0 to 1)	-10.2 (-11.1 to -9.3)	-7.4 (-15.8 to 3.0)	1.9 (-4.6 to 7.0)	-10.1 (-11.1 to -9.3)	-7.0 (-8.5 to -5.8)	-5.3 (-17.2 to 5.8)	2.1 (-9.7 to 13.2)	-6.9 (-8.1 to -5.8)
Nicaragua	8 (5-13)	0 (0-1)	0 (0-0)	8 (5 to 13)	0.7 (-1.2 to 3.8)	12.2 (7.2 to 17.5)	12.4 (7.0 to 17.1)	1.0 (-1.0 to 4.1)	-6.4 (-7.6 to -5.4)	-7.2 (-13.0 to -1.4)	2.4 (-3.4 to 8.2)	-6.4 (-7.5 to -5.4)
Niger	665 (382-1,074)	40 (10-116)	1 (0-2)	706 (410 to 1,143)	11.0 (6.9 to 16.2)	24.9 (16.0 to 34.6)	24.0 (15.4 to 30.2)	11.4 (7.4 to 16.6)	-9.2 (-10.7 to -7.3)	-11.0 (-20.8 to -1.3)	0.2 (-9.6 to 9.9)	-9.3 (-10.7 to -7.7)

	Number of deaths (all ages, both sexes) with 95% uncertainty intervals (2016)				Annualized rate of change of age-standardized rate (%) with 95% uncertainty intervals							
					1990-2006				2006-2016			
	**Drug-susceptible HIV-TB**	**MDR HIV-TB**	**XDR HIV-TB**	**All HIV-positive TB**	**Drug-susceptible HIV-TB**	**MDR HIV-TB**	**XDR HIV-TB**	**All HIV-positive TB**	**Drug-susceptible HIV-TB**	**MDR HIV-TB**	**XDR HIV-TB**	**All HIV-positive TB**
Nigeria	11,409 (6,252-21,240)	1,359 (482-2,972)	23 (8-51)	12,791 (7,036 to 23,316)	6.2 (2.4 to 14.3)	34.9 (27.0 to 46.9)	33.2 (26.6 to 38.3)	7.0 (3.2 to 14.9)	-12.4 (-14.3 to -10.5)	-13.6 (-23.0 to -4.3)	-2.4 (-11.8 to 6.8)	-12.6 (-13.9 to -11.2)
North Korea	82 (12-161)	6 (1-14)	1 (0-4)	89 (14 to 174)	14.8 (7.5 to 42.8)	46.2 (36.5 to 72.0)	17.1 (4.9 to 23.3)	15.4 (8.2 to 43.2)	2.1 (-1.5 to 10.3)	0.6 (-5.4 to 10.5)	12.4 (6.4 to 22.2)	2.1 (-1.5 to 10.3)
Northern Mariana Islands	0 (0-0)	0 (0-0)	0 (0-0)	0 (0 to 0)	-1.7 (-5.3 to 11.4)	10.6 (-2.0 to 43.0)	10.4 (2.6 to 16.0)	-0.5 (-3.0 to 12.8)	6.7 (2.3 to 10.6)	-4.5 (-29.6 to 12.0)	7.2 (-17.8 to 23.8)	5.4 (3.2 to 6.7)
Norway	2 (1-3)	0 (0-0)	0 (0-0)	2 (1 to 4)	-5.9 (-6.8 to -5.1)	-3.6 (-6.2 to -0.6)	12.8 (8.2 to 16.6)	-5.7 (-6.6 to -5.0)	-7.5 (-9.0 to -6.2)	-7.6 (-11.6 to -4.1)	-0.2 (-4.2 to 3.4)	-7.4 (-8.9 to -6.1)
Oman	1 (1-2)	0 (0-0)	0 (0-0)	1 (1 to 2)	-3.2 (-5.5 to -1.7)	2.8 (-7.6 to 17.6)	-3.0 (-9.3 to 2.1)	-3.0 (-5.2 to -1.5)	1.3 (-0.9 to 2.9)	1.7 (-9.9 to 12.9)	9.8 (-1.8 to 20.9)	1.4 (-0.6 to 2.8)
Pakistan	316 (24-641)	45 (3-101)	3 (0-7)	363 (28 to 753)	2.0 (-4.7 to 17.3)	34.0 (26.9 to 49.5)	9.2 (-2.7 to 15.0)	2.9 (-3.8 to 18.2)	4.3 (-9.5 to 14.1)	3.7 (-10.4 to 13.5)	13.2 (-0.8 to 23.0)	4.3 (-9.7 to 14.2)
Palestine	0 (0-0)	0 (0-0)	0 (0-0)	0 (0 to 0)	-4.4 (-6.2 to -2.7)	8.1 (-6.2 to 24.5)	-21.4 (-37.4 to -12.5)	-4.1 (-5.5 to -2.6)	-4.5 (-6.6 to -2.7)	-2.0 (-18.9 to 15.2)	6.0 (-10.9 to 23.2)	-4.3 (-5.5 to -3.2)
Panama	47 (28-75)	2 (1-5)	0 (0-1)	50 (30 to 79)	4.3 (2.5 to 6.6)	19.2 (13.7 to 24.5)	26.8 (20.3 to 31.6)	4.6 (2.9 to 6.9)	-4.2 (-5.0 to -3.4)	-5.5 (-11.8 to 0.6)	4.1 (-2.2 to 10.2)	-4.2 (-5.0 to -3.5)
Papua New Guinea	58 (29-99)	8 (1-23)	2 (0-6)	68 (40 to 109)	15.0 (8.1 to 24.5)	39.4 (23.4 to 71.8)	36.2 (15.7 to 45.5)	16.2 (11.6 to 25.8)	-9.5 (-14.2 to 1.6)	-13.9 (-35.3 to 18.5)	-2.1 (-23.6 to 30.3)	-10.0 (-11.2 to -8.7)
Paraguay	34 (21-60)	1 (1-3)	0 (0-1)	36 (23 to 64)	9.1 (6.0 to 17.3)	26.6 (22.8 to 34.5)	25.4 (22.6 to 27.8)	9.5 (6.3 to 17.7)	-9.8 (-14.3 to -4.9)	-11.6 (-16.8 to -6.2)	-2.0 (-7.2 to 3.4)	-9.8 (-14.3 to -5.0)
Peru	102 (67-143)	18 (11-29)	4 (2-6)	124 (82 to 175)	0.5 (-2.3 to 3.5)	12.2 (6.8 to 18.5)	31.7 (28.3 to 34.7)	1.5 (-1.3 to 4.4)	-12.5 (-14.4 to -10.7)	-12.7 (-16.0 to -9.4)	-3.1 (-6.4 to 0.2)	-12.3 (-14.2 to -10.5)
Philippines	81 (47-129)	8 (2-20)	2 (0-5)	90 (53 to 143)	2.7 (0.7 to 3.8)	31.3 (21.3 to 44.7)	16.2 (6.3 to 22.9)	3.5 (2.6 to 4.3)	-5.4 (-7.7 to -2.4)	-8.8 (-25.2 to 7.8)	3.0 (-13.5 to 19.6)	-5.6 (-6.4 to -4.8)
Poland	15 (8-25)	0 (0-0)	0 (0-0)	15 (8 to 25)	-2.9 (-4.2 to -0.5)	0.1 (-7.7 to 10.4)	5.5 (0.2 to 10.1)	-2.9 (-4.2 to -0.4)	-5.5 (-6.6 to -4.3)	-9.1 (-15.5 to -2.7)	2.1 (-4.3 to 8.4)	-5.6 (-6.6 to -4.3)
Portugal	61 (35-100)	2 (1-4)	1 (0-1)	63 (37 to 105)	1.7 (0.2 to 4.1)	3.0 (0.1 to 6.4)	25.9 (22.0 to 29.5)	1.8 (0.2 to 4.3)	-6.8 (-8.0 to -5.5)	-8.4 (-12.0 to -4.9)	-0.9 (-4.6 to 2.6)	-6.8 (-8.0 to -5.5)
Puerto Rico	8 (5-14)	0 (0-1)	0 (0-0)	8 (5 to 14)	-10.7 (-11.5 to -10.0)	6.6 (-3.0 to 18.9)	17.0 (7.9 to 23.4)	-10.4 (-11.0 to -9.8)	-5.9 (-7.3 to -4.2)	-15.7 (-32.4 to -1.1)	-6.0 (-22.8 to 8.5)	-6.2 (-7.5 to -4.7)
Qatar	0 (0-0)	0 (0-0)	0 (0-0)	0 (0 to 0)	-10.1 (-11.0 to -9.2)	-1.1 (-11.2 to 13.0)	-20.6 (-28.4 to -14.9)	-9.9 (-10.8 to -9.1)	-9.4 (-11.3 to -7.4)	-5.7 (-21.6 to 7.7)	2.3 (-13.6 to 15.8)	-9.2 (-10.9 to -7.3)

	Number of deaths (all ages, both sexes) with 95% uncertainty intervals (2016)				Annualized rate of change of age-standardized rate (%) with 95% uncertainty intervals							
					1990-2006				2006-2016			
	Drug-susceptible HIV-TB	MDR HIV-TB	XDR HIV-TB	All HIV-positive TB	Drug-susceptible HIV-TB	MDR HIV-TB	XDR HIV-TB	All HIV-positive TB	Drug-susceptible HIV-TB	MDR HIV-TB	XDR HIV-TB	All HIV-positive TB
Romania	31 (17-47)	2 (1-5)	1 (0-3)	34 (20 to 53)	3.5 (1.6 to 4.7)	10.3 (1.9 to 20.1)	24.2 (17.7 to 29.3)	4.0 (2.2 to 5.0)	-5.0 (-6.9 to -2.6)	-7.2 (-17.1 to 2.9)	3.9 (-6.0 to 14.0)	-4.9 (-6.3 to -2.9)
Russia	653 (352-1,137)	329 (174-559)	190 (99-324)	1,173 (691 to 1,935)	9.1 (7.9 to 10.2)	14.3 (11.5 to 17.8)	40.4 (36.7 to 43.8)	10.6 (9.6 to 11.5)	-6.6 (-9.1 to -4.4)	-5.0 (-8.3 to -2.4)	6.1 (2.9 to 8.8)	-4.8 (-6.0 to -3.3)
Rwanda	1,204 (855-1,496)	76 (37-139)	1 (1-2)	1,282 (907 to 1,590)	0.6 (-3.2 to 4.7)	28.7 (20.8 to 38.2)	33.7 (26.3 to 38.9)	1.0 (-2.8 to 5.1)	-15.6 (-18.2 to -13.2)	-15.9 (-24.6 to -5.0)	-4.7 (-13.4 to 6.2)	-15.6 (-18.0 to -13.4)
Saint Lucia	1 (0-1)	0 (0-0)	0 (0-0)	1 (1 to 1)	1.6 (0.7 to 2.5)	7.8 (-5.0 to 21.2)	17.9 (4.1 to 26.8)	1.7 (0.9 to 2.5)	-5.8 (-6.8 to -4.7)	-10.5 (-28.1 to 6.3)	-0.9 (-18.5 to 15.9)	-5.9 (-6.8 to -5.1)
Saint Vincent and the Grenadines	1 (1-2)	0 (0-0)	0 (0-0)	1 (1 to 2)	3.6 (2.5 to 4.6)	9.8 (-2.4 to 22.1)	21.7 (7.8 to 30.3)	3.7 (2.8 to 4.6)	-3.6 (-4.6 to -2.6)	-8.3 (-25.1 to 7.2)	1.3 (-15.5 to 16.8)	-3.7 (-4.6 to -2.8)
Samoa	0 (0-0)	0 (0-0)	0 (0-0)	0 (0 to 0)	3.7 (0.0 to 19.4)	7.3 (-21.9 to 37.8)	-2.5 (-36.5 to 10.0)	3.7 (0.0 to 19.4)	1.0 (-1.2 to 5.3)	-0.2 (-35.5 to 34.1)	11.5 (-23.8 to 45.8)	1.0 (-1.1 to 5.4)
Sao Tome and Principe	1 (1-2)	0 (0-0)	0 (0-0)	1 (1 to 2)	6.5 (2.4 to 21.4)	20.3 (11.3 to 36.4)	16.6 (6.9 to 23.2)	6.9 (2.9 to 21.9)	-14.6 (-17.9 to -11.6)	-16.2 (-27.1 to -5.4)	-5.0 (-16.0 to 5.7)	-14.7 (-18.0 to -11.8)
Saudi Arabia	32 (19-53)	3 (1-7)	0 (0-1)	35 (21 to 58)	4.5 (1.4 to 7.9)	30.0 (22.5 to 37.7)	9.2 (2.5 to 14.7)	5.0 (2.1 to 8.3)	-2.3 (-4.6 to -0.2)	-1.8 (-10.1 to 6.1)	6.2 (-2.0 to 14.1)	-2.2 (-4.2 to -0.2)
Senegal	483 (290-737)	16 (6-37)	0 (0-1)	499 (302 to 759)	9.8 (5.3 to 14.6)	31.5 (22.7 to 40.9)	18.4 (10.6 to 24.2)	10.1 (5.4 to 14.7)	-7.8 (-9.0 to -6.5)	-8.5 (-18.2 to 1.9)	2.7 (-7.0 to 13.1)	-7.8 (-9.0 to -6.6)
Serbia	4 (2-8)	0 (0-0)	0 (0-0)	4 (2 to 8)	-4.7 (-12.0 to 44.1)	3.4 (-8.4 to 18.6)	2.1 (-4.4 to 7.6)	-4.6 (-12.0 to 44.2)	-3.4 (-8.1 to -0.2)	0.3 (-12.7 to 11.9)	11.5 (-1.5 to 23.0)	-3.2 (-7.8 to -0.1)
Seychelles	0 (0-0)	0 (0-0)	0 (0-0)	0 (0 to 0)	6.7 (4.8 to 8.6)	10.0 (-4.2 to 25.2)	-7.8 (-23.4 to 1.2)	6.7 (4.8 to 8.6)	-0.3 (-1.5 to 0.7)	-2.2 (-22.9 to 15.8)	9.5 (-11.2 to 27.6)	-0.3 (-1.5 to 0.7)
Sierra Leone	330 (192-537)	15 (4-40)	0 (0-1)	345 (202 to 566)	19.5 (13.3 to 40.8)	28.0 (17.4 to 51.1)	22.7 (14.7 to 28.4)	19.7 (13.6 to 41.1)	-7.9 (-9.2 to -6.5)	-8.4 (-18.8 to 1.7)	2.8 (-7.6 to 12.9)	-7.9 (-9.1 to -6.6)
Singapore	3 (2-5)	0 (0-0)	0 (0-0)	3 (2 to 5)	-0.6 (-2.1 to 1.9)	2.6 (-1.1 to 6.6)	5.3 (0.5 to 9.3)	-0.6 (-2.1 to 1.9)	-6.7 (-7.7 to -5.5)	-1.8 (-5.9 to 2.3)	5.6 (1.5 to 9.7)	-6.5 (-7.6 to -5.4)
Slovakia	0 (0-0)	0 (0-0)	0 (0-0)	0 (0 to 0)	1.7 (-0.6 to 28.7)	5.5 (-4.0 to 37.5)	-10.1 (-16.7 to -5.3)	1.8 (-0.4 to 28.9)	-6.6 (-7.8 to -4.6)	-12.1 (-29.6 to -0.1)	-1.0 (-18.5 to 11.0)	-6.7 (-7.8 to -4.7)
Slovenia	0 (0-0)	0 (0-0)	0 (0-0)	0 (0 to 0)	-10.0 (-11.0 to -8.5)	-8.4 (-19.3 to 4.4)	-6.1 (-14.3 to 0.2)	-10.0 (-11.0 to -8.6)	-3.6 (-4.9 to -2.4)	-21.3 (-40.1 to -8.2)	-10.1 (-29.0 to 3.0)	-3.8 (-5.1 to -2.5)
Solomon Islands	2 (0-5)	0 (0-1)	0 (0-0)	2 (0 to 5)	6.2 (-0.8 to 23.4)	17.2 (-9.9 to 51.5)	17.0 (-18.1 to 29.7)	6.4 (-0.0 to 23.8)	0.2 (-5.2 to 13.4)	-2.3 (-35.5 to 33.1)	9.4 (-23.8 to 44.8)	0.1 (-4.6 to 12.4)

	Number of deaths (all ages, both sexes) with 95% uncertainty intervals (2016)				Annualized rate of change of age-standardized rate (%) with 95% uncertainty intervals							
					1990-2006				2006-2016			
	Drug-susceptible HIV-TB	MDR HIV-TB	XDR HIV-TB	All HIV-positive TB	Drug-susceptible HIV-TB	MDR HIV-TB	XDR HIV-TB	All HIV-positive TB	Drug-susceptible HIV-TB	MDR HIV-TB	XDR HIV-TB	All HIV-positive TB
Somalia	570 (325-883)	125 (46-257)	2 (1-4)	697 (420 to 1,049)	10.4 (7.8 to 15.3)	44.4 (37.4 to 53.8)	30.4 (24.5 to 35.1)	11.6 (9.3 to 16.4)	-3.3 (-5.6 to -0.7)	-3.0 (-12.6 to 6.5)	8.2 (-1.4 to 17.7)	-3.2 (-4.6 to -1.5)
South Africa	41,615 (25,610-58,710)	3,148 (1,470-5,870)	54 (25-100)	44,817 (27,517 to 62,866)	29.2 (25.8 to 32.1)	40.5 (34.7 to 46.1)	43.3 (38.7 to 46.9)	29.6 (26.2 to 32.5)	-9.5 (-10.7 to -8.4)	-10.8 (-15.9 to -6.0)	0.4 (-4.7 to 5.1)	-9.6 (-10.6 to -8.5)
South Korea	38 (23-76)	2 (1-4)	1 (0-1)	40 (25 to 81)	-2.1 (-12.5 to 46.7)	7.2 (-4.5 to 32.6)	10.8 (0.9 to 19.2)	-1.7 (-12.0 to 47.2)	0.7 (-9.0 to 10.8)	-2.6 (-13.2 to 9.7)	4.9 (-5.8 to 17.1)	0.5 (-9.1 to 10.7)
South Sudan	1,624 (862-2,755)	120 (33-306)	2 (1-5)	1,745 (959 to 2,908)	8.0 (6.4 to 9.8)	27.9 (19.9 to 36.8)	27.0 (18.0 to 33.2)	8.4 (6.9 to 10.2)	-2.1 (-4.0 to -0.4)	-1.1 (-11.6 to 9.0)	10.0 (-0.4 to 20.2)	-2.1 (-3.9 to -0.6)
Spain	62 (36-101)	2 (1-4)	1 (0-1)	65 (38 to 105)	-8.4 (-9.0 to -7.9)	-6.6 (-9.7 to -3.3)	15.5 (10.9 to 19.7)	-8.3 (-8.9 to -7.8)	-7.7 (-9.2 to -6.3)	-8.4 (-12.8 to -4.5)	-1.0 (-5.4 to 3.0)	-7.7 (-9.1 to -6.3)
Sri Lanka	5 (3-9)	0 (0-0)	0 (0-0)	5 (3 to 9)	1.8 (-0.1 to 7.0)	14.3 (1.4 to 29.8)	-3.7 (-15.8 to 4.5)	1.9 (0.0 to 7.0)	-9.3 (-10.7 to -7.9)	-12.0 (-31.1 to 4.7)	-0.2 (-19.3 to 16.4)	-9.4 (-10.6 to -7.9)
Sudan	512 (207-978)	32 (3-136)	3 (0-12)	547 (233 to 1,024)	3.7 (-1.6 to 9.1)	16.8 (2.3 to 32.0)	26.5 (10.5 to 35.3)	4.0 (-1.5 to 9.3)	-7.0 (-9.5 to -4.7)	-5.0 (-23.4 to 12.0)	3.0 (-15.4 to 20.0)	-6.9 (-8.3 to -4.8)
Suriname	5 (3-8)	0 (0-0)	0 (0-0)	5 (3 to 8)	8.6 (7.0 to 10.6)	14.0 (1.4 to 26.5)	24.6 (10.6 to 33.1)	8.7 (7.1 to 10.6)	-10.0 (-11.6 to -8.2)	-14.3 (-31.6 to 2.0)	-4.7 (-22.0 to 11.7)	-10.1 (-11.6 to -8.4)
Swaziland	1,353 (928-1,792)	361 (147-713)	6 (3-12)	1,720 (1,332 to 2,135)	26.6 (22.8 to 31.0)	46.4 (39.4 to 55.3)	54.1 (50.0 to 57.2)	27.8 (24.3 to 32.2)	-12.4 (-16.3 to -8.8)	-10.9 (-21.6 to -2.1)	0.2 (-10.5 to 9.1)	-12.1 (-14.1 to -9.2)
Sweden	4 (2-6)	0 (0-1)	0 (0-0)	4 (2 to 7)	-7.2 (-8.2 to -6.4)	-1.7 (-4.3 to 0.8)	11.9 (7.4 to 15.5)	-6.9 (-7.9 to -6.1)	-6.8 (-8.0 to -5.1)	-3.8 (-7.0 to -0.8)	3.6 (0.4 to 6.7)	-6.4 (-7.6 to -4.7)
Switzerland	3 (2-5)	0 (0-0)	0 (0-0)	3 (2 to 6)	-9.9 (-10.6 to -9.1)	-5.0 (-8.0 to -2.1)	10.6 (6.1 to 14.5)	-9.7 (-10.4 to -8.9)	-7.6 (-9.0 to -6.1)	-4.0 (-7.4 to -0.1)	3.4 (0.0 to 7.3)	-7.2 (-8.6 to -5.9)
Syria	0 (0-1)	0 (0-0)	0 (0-0)	1 (0 to 1)	-7.6 (-9.7 to -5.9)	15.7 (5.1 to 30.5)	-5.7 (-13.2 to -0.3)	-6.2 (-7.0 to -4.7)	-3.3 (-9.5 to -0.2)	-4.2 (-20.6 to 6.6)	3.9 (-12.5 to 14.6)	-3.4 (-4.8 to -2.3)
Taiwan	26 (15-44)	1 (1-3)	0 (0-1)	28 (16 to 46)	10.8 (6.6 to 27.0)	37.4 (31.1 to 49.9)	8.3 (2.7 to 13.0)	11.2 (7.0 to 27.3)	1.3 (0.2 to 2.5)	-0.9 (-5.9 to 3.7)	10.8 (5.8 to 15.5)	1.3 (0.1 to 2.5)
Tajikistan	2 (1-4)	1 (0-2)	0 (0-1)	4 (2 to 6)	12.7 (8.1 to 15.4)	50.0 (40.8 to 62.4)	30.9 (22.6 to 36.0)	15.2 (13.6 to 17.0)	-17.4 (-22.2 to -10.1)	-19.3 (-28.7 to -6.1)	-8.2 (-17.6 to 5.1)	-17.1 (-18.9 to -14.7)
Tanzania	7,717 (4,107-12,913)	369 (97-1,049)	6 (2-17)	8,092 (4,300 to 13,449)	6.2 (4.7 to 7.5)	29.1 (22.3 to 37.3)	30.2 (23.8 to 35.4)	6.5 (5.0 to 7.7)	-10.4 (-12.2 to -8.6)	-9.3 (-19.4 to -0.1)	1.8 (-8.2 to 11.1)	-10.3 (-12.0 to -8.6)
Thailand	1,518 (896-2,378)	132 (36-346)	32 (9-83)	1,682 (1,024 to 2,643)	16.2 (11.3 to 20.6)	21.9 (12.3 to 33.3)	31.9 (26.3 to 36.4)	16.4 (11.4 to 20.8)	-7.5 (-9.7 to -5.8)	-3.0 (-15.8 to 7.2)	8.7 (-4.1 to 19.0)	-7.1 (-8.6 to -5.7)

	Number of deaths (all ages, both sexes) with 95% uncertainty intervals (2016)				Annualized rate of change of age-standardized rate (%) with 95% uncertainty intervals							
					1990-2006				2006-2016			
	Drug-susceptible HIV-TB	MDR HIV-TB	XDR HIV-TB	All HIV-positive TB	Drug-susceptible HIV-TB	MDR HIV-TB	XDR HIV-TB	All HIV-positive TB	Drug-susceptible HIV-TB	MDR HIV-TB	XDR HIV-TB	All HIV-positive TB
The Bahamas	5 (3-8)	0 (0-1)	0 (0-0)	5 (3 to 8)	-0.1 (-1.0 to 0.9)	17.2 (5.4 to 30.7)	22.8 (10.0 to 30.8)	0.1 (-0.7 to 1.1)	-4.6 (-6.1 to -3.1)	-1.4 (-18.0 to 14.3)	8.2 (-8.4 to 24.0)	-4.5 (-5.6 to -3.1)
The Gambia	110 (56-193)	6 (2-16)	0 (0-0)	117 (60 to 205)	13.4 (9.1 to 19.5)	27.3 (17.5 to 37.5)	24.4 (14.8 to 30.4)	13.7 (9.5 to 20.0)	-4.6 (-5.8 to -3.5)	-6.6 (-16.5 to 3.4)	4.6 (-5.3 to 14.6)	-4.7 (-5.7 to -3.8)
Timor-Leste	3 (2-4)	0 (0-0)	0 (0-0)	3 (2 to 4)	10.9 (6.3 to 23.5)	23.6 (8.3 to 44.2)	17.7 (2.7 to 26.7)	11.1 (6.5 to 23.6)	-6.6 (-8.1 to -4.5)	-14.4 (-33.7 to 2.0)	-2.6 (-22.0 to 13.8)	-6.8 (-8.1 to -5.3)
Togo	530 (286-879)	31 (7-76)	1 (0-1)	561 (309 to 938)	9.9 (7.5 to 13.4)	23.4 (15.4 to 31.7)	28.1 (18.9 to 34.3)	10.3 (8.0 to 13.8)	-9.6 (-10.9 to -8.1)	-11.3 (-22.0 to -1.1)	-0.1 (-10.9 to 10.0)	-9.7 (-10.8 to -8.4)
Tonga	0 (0-0)	0 (0-0)	0 (0-0)	0 (0 to 0)	4.5 (1.4 to 16.4)	13.6 (-11.8 to 44.8)	6.3 (-25.3 to 18.9)	4.7 (2.1 to 16.3)	-0.1 (-2.9 to 4.2)	-3.2 (-38.5 to 30.6)	8.5 (-26.7 to 42.4)	-0.1 (-2.0 to 1.9)
Trinidad and Tobago	10 (6-16)	0 (0-1)	0 (0-0)	10 (6 to 16)	1.1 (0.2 to 1.8)	6.6 (-6.8 to 21.5)	19.3 (4.1 to 27.6)	1.2 (0.4 to 1.9)	-4.8 (-5.9 to -3.4)	-9.0 (-26.3 to 8.0)	0.6 (-16.6 to 17.6)	-4.8 (-5.8 to -3.6)
Tunisia	13 (7-21)	0 (0-1)	0 (0-0)	13 (7 to 22)	7.0 (2.3 to 11.2)	27.8 (14.3 to 43.5)	2.0 (-11.6 to 10.2)	7.2 (2.5 to 11.5)	0.5 (-1.1 to 2.2)	-1.7 (-17.1 to 17.2)	6.3 (-9.1 to 25.3)	0.4 (-1.1 to 2.0)
Turkey	9 (5-15)	1 (0-2)	0 (0-0)	10 (6 to 16)	9.2 (2.5 to 35.2)	27.5 (11.5 to 58.7)	-1.5 (-12.7 to 6.1)	9.9 (3.3 to 35.8)	-4.6 (-6.5 to -1.6)	-5.1 (-19.1 to 12.7)	3.0 (-11.1 to 20.7)	-4.6 (-5.7 to -3.5)
Turkmenistan	18 (9-31)	9 (4-16)	5 (2-9)	31 (18 to 51)	-0.3 (-5.3 to 1.9)	38.1 (29.5 to 50.5)	41.1 (34.0 to 45.9)	2.9 (2.3 to 3.5)	-8.4 (-14.6 to 0.4)	-9.7 (-18.7 to 1.9)	1.4 (-7.6 to 13.0)	-7.8 (-9.0 to -6.1)
Uganda	13,642 (10,846-17,573)	923 (388-1,858)	16 (6-31)	14,580 (11,675 to 18,579)	1.0 (-1.0 to 3.4)	28.8 (21.8 to 36.6)	38.9 (33.3 to 43.1)	1.4 (-0.6 to 3.7)	-11.2 (-13.0 to -9.3)	-10.8 (-20.8 to -0.3)	0.3 (-9.6 to 10.8)	-11.2 (-12.9 to -9.3)
Ukraine	335 (167-570)	295 (162-512)	171 (93-298)	801 (472 to 1,303)	8.2 (5.2 to 10.5)	46.9 (42.8 to 51.6)	50.0 (45.8 to 53.7)	13.0 (11.8 to 14.2)	-9.0 (-13.0 to -4.5)	-10.1 (-13.3 to -6.7)	1.1 (-2.1 to 4.5)	-8.0 (-9.1 to -6.8)
United Arab Emirates	2 (1-3)	0 (0-1)	0 (0-0)	2 (1 to 3)	-0.7 (-3.6 to 7.7)	12.5 (-2.4 to 28.7)	-8.1 (-23.4 to 1.1)	-0.4 (-3.0 to 7.8)	15.5 (4.8 to 18.8)	16.0 (-3.6 to 33.4)	24.0 (4.4 to 41.4)	15.6 (5.1 to 18.3)
United Kingdom	2 (1-3)	0 (0-0)	0 (0-0)	2 (1 to 3)	-5.2 (-5.5 to -4.9)	-3.1 (-3.6 to -2.5)	-10.5 (-14.6 to -6.7)	-5.1 (-5.4 to -4.8)	-4.7 (-5.3 to -4.1)	-4.7 (-5.6 to -3.8)	2.8 (1.8 to 3.7)	-4.7 (-5.3 to -4.0)
United States	131 (77-211)	5 (3-9)	2 (1-3)	137 (81 to 222)	-10.5 (-11.0 to -9.9)	-12.1 (-12.8 to -11.4)	8.4 (4.6 to 12.1)	-10.5 (-11.1 to -9.9)	-5.6 (-6.3 to -4.9)	-6.2 (-7.0 to -5.4)	1.3 (0.5 to 2.1)	-5.6 (-6.3 to -4.9)
Uruguay	16 (9-25)	0 (0-0)	0 (0-0)	16 (9 to 25)	-0.9 (-2.1 to 1.1)	1.7 (-10.5 to 14.8)	5.5 (-6.3 to 13.0)	-0.9 (-2.0 to 1.1)	-1.4 (-2.1 to -0.6)	7.5 (-7.1 to 24.5)	14.9 (0.3 to 31.9)	-1.3 (-2.0 to -0.6)
Uzbekistan	27 (9-53)	27 (12-48)	15 (7-27)	69 (39 to 111)	0.6 (-5.4 to 3.6)	42.3 (34.1 to 54.9)	37.1 (32.1 to 40.9)	6.0 (5.4 to 6.7)	-6.9 (-18.4 to 5.1)	-8.5 (-16.7 to -1.3)	2.6 (-5.5 to 9.8)	-6.2 (-7.1 to -5.1)

	Number of deaths (all ages, both sexes) with 95% uncertainty intervals (2016)				Annualized rate of change of age-standardized rate (%) with 95% uncertainty intervals							
					1990-2006				2006-2016			
	Drug-susceptible HIV-TB	MDR HIV-TB	XDR HIV-TB	All HIV-positive TB	Drug-susceptible HIV-TB	MDR HIV-TB	XDR HIV-TB	All HIV-positive TB	Drug-susceptible HIV-TB	MDR HIV-TB	XDR HIV-TB	All HIV-positive TB
Vanuatu	1 (0-2)	0 (0-0)	0 (0-0)	1 (0 to 2)	6.2 (0.2 to 23.3)	10.8 (-16.7 to 42.5)	4.3 (-31.7 to 15.8)	6.3 (0.3 to 23.3)	1.1 (-4.2 to 11.9)	-0.7 (-34.5 to 36.8)	11.2 (-22.8 to 48.6)	1.1 (-4.2 to 11.9)
Venezuela	168 (99-267)	7 (3-16)	2 (1-3)	177 (104 to 280)	-0.8 (-1.6 to -0.1)	14.2 (9.0 to 18.9)	19.7 (13.9 to 24.3)	-0.5 (-1.2 to 0.2)	-0.7 (-1.9 to 0.3)	-2.1 (-8.0 to 3.9)	7.5 (1.6 to 13.5)	-0.7 (-1.9 to 0.2)
Vietnam	1,997 (1,122-3,041)	243 (64-614)	58 (16-150)	2,298 (1,290 to 3,475)	23.8 (19.0 to 28.1)	36.8 (27.8 to 49.2)	35.2 (30.9 to 38.8)	24.4 (19.6 to 28.6)	-6.7 (-12.0 to -2.6)	-5.1 (-18.6 to 5.4)	6.7 (-6.8 to 17.1)	-6.4 (-11.4 to -2.2)
Virgin Islands, U.S.	0 (0-1)	0 (0-0)	0 (0-0)	0 (0 to 1)	4.3 (2.9 to 6.8)	10.3 (-3.2 to 24.5)	12.0 (-2.9 to 21.0)	4.5 (3.0 to 6.8)	2.8 (1.4 to 4.1)	-2.0 (-19.1 to 15.5)	7.6 (-9.5 to 25.2)	2.7 (1.5 to 3.8)
Yemen	32 (2-92)	2 (0-10)	0 (0-1)	34 (2 to 98)	2.2 (-5.3 to 15.5)	21.3 (10.6 to 43.7)	15.4 (2.8 to 22.3)	2.8 (-4.6 to 16.1)	-7.5 (-23.5 to -0.2)	-9.7 (-34.3 to 4.0)	-1.7 (-26.3 to 12.0)	-7.6 (-23.6 to -0.6)
Zambia	10,109 (7,503-12,848)	504 (175-1,198)	9 (3-21)	10,621 (7,850 to 13,391)	8.6 (6.6 to 10.9)	23.8 (16.5 to 31.8)	41.4 (36.7 to 45.1)	8.9 (6.9 to 11.1)	-13.2 (-15.0 to -11.1)	-12.7 (-22.4 to -3.2)	-1.5 (-11.2 to 7.9)	-13.2 (-14.8 to -11.2)
Zimbabwe	11,210 (8,078-14,056)	1,110 (269-2,989)	19 (5-50)	12,338 (9,336 to 15,127)	9.3 (7.5 to 11.0)	24.5 (14.9 to 33.9)	46.7 (37.2 to 52.4)	9.7 (8.0 to 11.3)	-15.8 (-18.2 to -12.6)	-12.8 (-23.4 to -1.0)	-1.6 (-12.2 to 10.2)	-15.5 (-17.6 to -12.7)

eTable 10. Age-standardized incidence and mortality rates per 100,000 for tuberculosis among HIV-negative individuals in 2016, both sexes (Input data for Figure 3)

Location	Incidence	Lower bound for incidence	Upper bound for incidence	Mortality	Lower bound for mortality	Upper bound for mortality
Afghanistan	208.8	175.2	244.8	107.8	36.0	167.9
Albania	12.6	10.9	14.3	0.3	0.3	0.4
Algeria	51.3	42.4	61.3	4.4	1.9	6.6
American Samoa	20.7	17.4	24.7	1.3	1.1	1.6
Andorra	9.2	6.8	12.6	0.2	0.2	0.5
Angola	388.3	334.1	448.4	110.0	84.8	145.4
Antigua and Barbuda	10.5	8.6	12.9	0.6	0.5	0.7
Argentina	12.8	10.4	15.7	1.4	1.3	1.6
Armenia	34.0	29.8	38.8	2.3	2.0	2.8
Australia	4.6	3.9	5.3	0.2	0.2	0.2
Austria	7.0	5.4	9.1	0.3	0.3	0.4
Azerbaijan	120.3	104.0	137.3	6.1	4.7	8.8
Bahrain	23.2	15.9	32.2	1.7	1.4	2.1
Bangladesh	131.3	116.1	146.8	19.5	17.0	22.3
Barbados	10.4	8.4	12.6	0.6	0.6	0.7
Belarus	34.0	29.3	38.9	3.5	2.8	4.5
Belgium	8.0	6.2	10.3	0.4	0.4	0.5
Belize	33.1	27.0	40.0	7.8	6.6	9.2
Benin	137.2	113.2	164.1	53.8	42.0	68.3
Bermuda	5.2	4.0	6.7	0.1	0.1	0.1
Bhutan	146.9	126.0	170.2	10.2	6.1	16.5
Bolivia	99.0	88.9	108.7	13.5	10.0	17.9
Bosnia and Herzegovina	24.8	21.8	27.9	2.5	2.1	2.9
Botswana	530.9	397.5	712.2	59.2	29.1	88.2
Brazil	29.4	25.5	33.4	2.7	2.5	2.8
Brunei	59.4	51.1	67.5	6.7	5.7	7.7
Bulgaria	19.9	17.8	22.1	1.0	0.8	1.2

Location	Incidence	Lower bound for incidence	Upper bound for incidence	Mortality	Lower bound for mortality	Upper bound for mortality
Burkina Faso	187.3	155.0	226.6	83.8	74.0	94.1
Burundi	396.0	321.1	490.2	210.3	159.4	264.5
Cambodia	246.6	216.1	280.9	29.1	24.2	37.0
Cameroon	154.4	119.0	200.4	52.1	32.6	72.6
Canada	4.0	3.6	4.4	0.2	0.2	0.2
Cape Verde	72.6	59.7	88.8	11.0	8.1	14.4
Central African Republic	842.7	670.9	1040.0	483.8	366.8	616.1
Chad	177.3	142.4	217.7	76.7	59.7	97.0
Chile	10.9	9.0	13.0	2.2	1.7	2.8
China	78.1	73.8	82.4	2.7	2.6	3.0
Colombia	20.1	17.2	23.2	2.1	1.8	2.4
Comoros	226.7	186.9	275.5	67.1	50.0	84.4
Congo	419.8	348.1	501.1	90.3	69.0	114.0
Costa Rica	9.1	7.7	10.6	1.0	0.9	1.1
Cote d'Ivoire	180.4	144.2	225.6	65.2	44.2	85.8
Croatia	10.6	9.3	12.2	0.9	0.7	1.0
Cuba	5.7	4.8	6.8	0.3	0.2	0.3
Cyprus	8.8	6.6	11.7	0.3	0.3	0.4
Czech Republic	5.1	4.4	5.8	0.3	0.3	0.3
Democratic Republic of the Congo	489.5	418.8	566.0	204.8	148.8	271.3
Denmark	6.0	4.6	7.9	0.3	0.2	0.3
Djibouti	383.1	320.8	453.1	80.1	57.0	102.4
Dominica	36.9	30.7	43.9	4.3	3.6	5.1
Dominican Republic	44.7	37.4	52.8	4.3	3.6	5.0
Ecuador	29.2	24.9	33.5	4.5	4.0	5.1
Egypt	15.6	12.5	19.2	1.3	1.0	1.7
El Salvador	22.0	19.1	25.2	1.5	1.3	1.7
Equatorial Guinea	354.9	286.8	432.6	43.5	25.7	65.8
Eritrea	341.1	277.1	413.0	147.7	90.5	185.3

Location	Incidence	Lower bound for incidence	Upper bound for incidence	Mortality	Lower bound for mortality	Upper bound for mortality
Estonia	16.7	14.0	19.7	1.5	1.2	1.9
Ethiopia	265.4	220.1	317.1	100.0	83.4	118.5
Federated States of Micronesia	129.9	111.1	151.5	10.5	8.1	13.5
Fiji	57.1	50.9	63.9	5.7	4.4	7.2
Finland	5.3	4.1	6.9	0.5	0.4	0.5
France	7.0	5.5	9.0	0.7	0.6	0.8
Gabon	309.3	251.3	377.5	46.5	35.0	60.6
Georgia	70.1	62.3	79.2	3.8	3.0	4.9
Germany	6.3	4.8	8.2	0.3	0.2	0.3
Ghana	200.7	160.1	251.0	49.1	41.5	57.7
Greece	4.6	3.5	6.0	0.6	0.6	0.7
Greenland	46.6	40.9	52.8	7.0	5.6	9.0
Grenada	9.3	7.5	11.4	1.0	0.8	1.1
Guam	60.1	51.7	69.0	3.1	2.5	3.7
Guatemala	21.1	17.7	24.6	3.2	2.4	4.0
Guinea	201.5	161.3	243.8	94.5	71.9	114.2
Guinea-Bissau	252.7	195.0	323.1	132.3	83.4	165.3
Guyana	60.5	50.6	71.7	11.8	10.1	13.8
Haiti	86.4	72.3	102.2	18.6	12.7	24.8
Honduras	44.8	38.8	51.3	9.6	7.2	12.4
Hungary	7.8	6.8	8.9	0.4	0.4	0.5
Iceland	8.8	6.6	11.7	0.3	0.3	0.3
India	235.8	219.2	255.0	44.7	41.4	47.8
Indonesia	258.5	223.7	296.7	51.9	48.6	55.3
Iran	17.8	15.7	20.1	1.9	1.6	2.3
Iraq	51.7	35.4	71.1	7.1	5.6	8.9
Ireland	6.5	5.0	8.4	0.5	0.4	0.6
Israel	4.3	3.2	5.6	0.3	0.2	0.4
Italy	5.7	4.7	6.9	0.3	0.3	0.4

Location	Incidence	Lower bound for incidence	Upper bound for incidence	Mortality	Lower bound for mortality	Upper bound for mortality
Jamaica	7.8	6.4	9.5	0.6	0.5	0.7
Japan	11.9	10.8	13.2	1.0	0.9	1.1
Jordan	7.8	5.9	10.1	0.7	0.5	0.9
Kazakhstan	84.0	74.2	94.8	6.0	4.9	7.4
Kenya	178.8	149.5	212.5	45.1	34.8	57.6
Kiribati	290.7	257.9	326.4	64.5	53.6	75.8
Kuwait	24.4	16.8	33.6	1.7	1.3	2.1
Kyrgyzstan	113.4	100.4	127.6	9.7	8.5	11.0
Laos	136.8	113.9	163.2	40.7	34.0	51.8
Latvia	30.1	26.0	34.3	2.1	1.7	2.5
Lebanon	14.0	11.9	16.2	0.9	0.7	1.2
Lesotho	729.6	537.2	1013.1	266.6	203.7	341.7
Liberia	211.2	175.5	252.2	66.1	49.5	85.6
Libya	31.7	21.6	43.9	3.5	1.6	4.7
Lithuania	42.6	37.6	48.4	4.6	4.0	5.4
Luxembourg	7.2	5.3	9.7	0.2	0.2	0.2
Macedonia	20.7	18.1	23.7	1.3	1.2	1.5
Madagascar	155.1	123.8	198.2	23.2	18.0	29.0
Malawi	180.7	145.1	226.2	78.8	56.1	106.5
Malaysia	56.4	44.5	71.1	6.6	5.8	7.5
Maldives	52.2	41.5	66.5	4.0	3.1	4.9
Mali	72.9	60.7	87.4	32.4	24.6	45.9
Malta	6.0	4.4	8.1	0.2	0.1	0.2
Marshall Islands	205.4	178.7	234.2	10.5	7.7	12.8
Mauritania	95.3	78.1	114.2	18.8	13.5	29.4
Mauritius	19.3	15.3	24.4	1.0	0.8	1.1
Mexico	18.2	15.5	21.2	2.3	2.2	2.4
Moldova	64.4	56.4	73.1	5.1	4.2	6.5
Mongolia	159.8	141.4	180.2	10.9	8.9	13.9

Location	Incidence	Lower bound for incidence	Upper bound for incidence	Mortality	Lower bound for mortality	Upper bound for mortality
Montenegro	14.8	12.6	17.3	0.5	0.4	0.6
Morocco	183.9	127.1	256.0	47.2	34.5	92.1
Mozambique	236.7	183.3	308.1	121.7	94.6	157.3
Myanmar	178.7	138.9	231.1	37.1	32.1	44.4
Namibia	510.3	378.4	686.7	64.2	41.9	111.6
Nepal	141.8	124.4	159.1	23.8	17.1	40.8
Netherlands	5.1	3.9	6.6	0.3	0.2	0.3
New Zealand	6.0	5.1	7.0	0.2	0.2	0.3
Nicaragua	38.3	32.8	43.7	3.6	3.0	4.2
Niger	147.7	123.2	176.1	72.0	53.5	93.1
Nigeria	199.2	162.0	238.5	45.0	35.2	59.3
North Korea	276.0	246.3	308.7	9.9	8.4	11.7
Northern Mariana Islands	52.8	45.2	61.2	2.8	2.3	3.3
Norway	5.7	4.3	7.5	0.4	0.3	0.5
Oman	12.9	8.9	17.9	1.0	0.9	1.2
Pakistan	235.3	188.2	289.8	42.8	34.3	54.5
Palestine	3.8	2.5	5.3	0.1	0.1	0.2
Panama	35.9	30.5	41.6	5.3	4.6	6.2
Papua New Guinea	134.1	117.7	150.5	10.8	8.9	12.9
Paraguay	39.8	33.6	46.4	5.1	4.4	6.0
Peru	56.2	49.5	63.3	5.6	4.4	7.2
Philippines	259.9	230.6	293.9	45.3	39.3	52.1
Poland	12.8	11.2	14.4	1.0	0.9	1.3
Portugal	15.8	12.1	20.9	1.1	1.0	1.3
Puerto Rico	3.2	2.6	3.9	0.5	0.4	0.5
Qatar	34.2	23.3	47.9	1.1	0.8	1.6
Romania	60.5	53.8	67.9	3.8	3.3	4.4
Russian Federation	57.9	50.4	66.2	7.2	5.0	10.2
Rwanda	222.1	176.2	281.5	78.1	63.8	95.3

Location	Incidence	Lower bound for incidence	Upper bound for incidence	Mortality	Lower bound for mortality	Upper bound for mortality
Saint Lucia	25.8	21.5	30.8	2.7	2.5	3.0
Saint Vincent and the Grenadines	25.9	20.9	31.3	3.1	2.8	3.5
Samoa	31.5	27.2	36.7	4.4	3.3	8.1
Sao Tome and Principe	102.6	83.5	124.8	17.5	13.5	21.6
Saudi Arabia	42.3	38.7	46.2	6.7	6.0	7.5
Senegal	187.7	155.5	224.0	73.8	63.8	87.6
Serbia	18.1	15.7	20.5	1.0	0.9	1.2
Seychelles	30.6	24.1	39.5	1.7	1.4	2.0
Sierra Leone	252.5	210.0	303.0	82.0	69.9	94.4
Singapore	33.7	29.4	38.3	1.1	0.9	1.3
Slovakia	7.1	6.1	8.2	0.4	0.3	0.5
Slovenia	7.0	6.0	8.2	0.4	0.3	0.5
Solomon Islands	96.7	86.7	107.9	26.5	21.0	33.0
Somalia	392.0	331.4	461.3	203.2	157.8	266.0
South Africa	419.1	329.5	531.8	52.0	47.8	57.3
South Korea	69.9	61.9	78.3	4.9	3.8	6.2
South Sudan	213.6	173.4	261.9	116.1	86.1	159.7
Spain	8.1	6.2	10.6	0.4	0.4	0.5
Sri Lanka	40.4	33.4	48.2	3.2	2.5	4.2
Sudan	122.8	106.1	142.3	27.0	13.7	46.2
Suriname	23.7	19.3	28.6	2.2	1.9	2.5
Swaziland	562.3	412.8	774.9	115.3	74.3	163.3
Sweden	6.8	5.5	8.3	0.4	0.3	0.4
Switzerland	6.0	4.7	7.8	0.2	0.2	0.3
Syria	22.1	15.1	31.3	0.5	0.4	0.6
Taiwan	23.0	20.9	25.3	2.8	2.4	3.2
Tajikistan	78.4	69.5	87.7	6.4	5.2	8.4
Tanzania	182.0	146.7	222.5	59.1	47.0	75.8
Thailand	70.0	55.9	88.8	8.6	7.5	10.2

Location	Incidence	Lower bound for incidence	Upper bound for incidence	Mortality	Lower bound for mortality	Upper bound for mortality
The Bahamas	32.2	26.4	39.2	2.6	2.2	3.1
The Gambia	153.2	129.3	177.8	63.7	46.0	97.4
Timor-Leste	110.4	89.4	137.8	25.5	18.6	35.7
Togo	186.3	150.5	226.9	67.0	54.2	79.4
Tonga	23.9	20.4	28.4	1.9	1.6	2.4
Trinidad and Tobago	12.3	10.1	14.8	1.5	1.3	1.7
Tunisia	29.4	20.4	40.4	3.1	2.3	4.2
Turkey	24.8	21.3	28.5	1.1	0.9	1.4
Turkmenistan	153.3	133.7	174.0	9.8	8.8	11.3
Uganda	282.7	219.7	362.4	146.3	113.9	180.6
Ukraine	50.6	43.4	58.9	5.8	4.2	7.8
United Arab Emirates	14.1	12.4	16.0	2.0	1.6	2.4
United Kingdom	11.1	8.9	13.6	0.4	0.3	0.4
United States	3.1	2.8	3.4	0.2	0.2	0.2
Uruguay	16.8	14.3	19.7	1.2	1.1	1.3
Uzbekistan	66.9	59.7	74.7	9.4	8.1	10.8
Vanuatu	87.4	77.6	99.0	21.6	17.5	27.0
Venezuela	21.2	18.1	24.5	3.0	2.5	3.6
Vietnam	125.4	107.4	146.9	22.7	19.5	27.0
Virgin Islands, U.S.	12.6	10.0	15.5	0.7	0.6	0.8
Yemen	67.4	46.5	92.2	17.1	7.0	26.6
Zambia	384.8	302.0	491.5	160.2	109.1	207.8
Zimbabwe	443.5	333.6	600.8	129.8	93.0	163.2

eTable 11. Age-standardized incidence and mortality rates per 100,000 for multi-drug resistant tuberculosis among HIV-negative individuals in 2016, both sexes

Location	Incidence	Lower bound for incidence	Upper bound for incidence	Mortality	Lower bound for mortality	Upper bound for mortality
Afghanistan	13.5	2.7	40.6	17.1	2.8	46.6
Albania	0.1	0.0	0.3	0.0	0.0	0.0
Algeria	1.1	0.1	4.6	0.2	0.0	1.1
American Samoa	0.2	0.0	1.6	0.0	0.0	0.2
Andorra	0.0	0.0	0.0	0.0	0.0	0.0
Angola	2.8	1.5	5.0	2.4	1.2	4.5
Antigua and Barbuda	0.1	0.0	0.2	0.0	0.0	0.0
Argentina	0.3	0.1	1.1	0.1	0.0	0.3
Armenia	3.3	0.7	8.6	0.4	0.1	0.9
Australia	0.1	0.0	0.2	0.0	0.0	0.0
Austria	0.2	0.1	0.3	0.0	0.0	0.0
Azerbaijan	17.1	8.6	29.5	1.5	0.8	2.5
Bahrain	0.6	0.1	1.5	0.1	0.0	0.3
Bangladesh	2.9	2.2	3.6	1.2	0.9	1.6
Barbados	0.0	0.0	0.0	0.0	0.0	0.0
Belarus	10.7	8.9	12.9	1.5	1.1	1.9
Belgium	0.1	0.1	0.2	0.0	0.0	0.0
Belize	0.2	0.0	0.7	0.1	0.0	0.4
Benin	1.3	0.4	3.1	1.5	0.5	3.6
Bermuda	0.0	0.0	0.0	0.0	0.0	0.0
Bhutan	3.9	2.8	5.2	0.8	0.4	1.3
Bolivia	2.8	0.7	8.1	1.0	0.2	2.6
Bosnia and Herzegovina	0.1	0.0	0.2	0.0	0.0	0.0
Botswana	9.7	3.0	22.7	2.9	0.8	6.8
Brazil	0.4	0.4	0.5	0.1	0.1	0.1

Location	Incidence	Lower bound for incidence	Upper bound for incidence	Mortality	Lower bound for mortality	Upper bound for mortality
Brunei	0.0	0.0	0.1	0.0	0.0	0.0
Bulgaria	0.5	0.1	1.2	0.1	0.0	0.1
Burkina Faso	2.9	0.8	7.3	3.8	1.1	9.5
Burundi	8.0	2.1	20.1	12.2	3.3	30.1
Cambodia	3.6	0.6	12.4	1.1	0.2	3.4
Cameroon	2.3	0.6	6.0	2.3	0.6	6.0
Canada	0.0	0.0	0.0	0.0	0.0	0.0
Cape Verde	1.1	0.3	2.8	0.5	0.1	1.3
Central African Republic	4.6	2.5	8.1	8.0	4.2	14.1
Chad	2.8	0.8	7.3	3.6	1.0	8.8
Chile	0.1	0.0	0.2	0.0	0.0	0.1
China	3.2	2.9	3.7	0.3	0.2	0.3
Colombia	0.5	0.2	0.9	0.1	0.1	0.2
Comoros	4.5	1.3	11.5	3.9	1.1	9.1
Congo	3.1	1.6	5.4	2.0	1.0	3.6
Costa Rica	0.1	0.1	0.2	0.0	0.0	0.1
Cote d'Ivoire	4.4	1.5	10.1	4.6	1.6	10.4
Croatia	0.0	0.0	0.1	0.0	0.0	0.0
Cuba	0.1	0.0	0.2	0.0	0.0	0.0
Cyprus	0.0	0.0	0.0	0.0	0.0	0.0
Czech Republic	0.1	0.0	0.2	0.0	0.0	0.0
Democratic Republic of the Congo	3.6	1.9	6.1	4.5	2.2	8.1
Denmark	0.0	0.0	0.1	0.0	0.0	0.0
Djibouti	15.6	7.9	27.7	9.2	4.6	16.2
Dominica	0.2	0.0	0.7	0.1	0.0	0.2
Dominican Republic	0.2	0.0	0.9	0.1	0.0	0.2
Ecuador	1.5	0.4	4.0	0.6	0.2	1.2
Egypt	0.7	0.2	1.9	0.2	0.0	0.4
El Salvador	0.3	0.1	0.5	0.1	0.0	0.1

Location	Incidence	Lower bound for incidence	Upper bound for incidence	Mortality	Lower bound for mortality	Upper bound for mortality
Equatorial Guinea	2.6	1.4	4.5	1.0	0.4	1.8
Eritrea	7.1	1.9	17.4	8.9	2.5	22.0
Estonia	2.6	1.9	3.5	0.4	0.3	0.5
Ethiopia	5.5	1.6	13.3	6.0	1.8	13.7
Federated States of Micronesia	1.3	0.0	8.9	0.2	0.0	1.8
Fiji	0.1	0.0	0.5	0.0	0.0	0.1
Finland	0.1	0.1	0.1	0.0	0.0	0.0
France	0.1	0.1	0.1	0.0	0.0	0.0
Gabon	2.2	1.1	3.8	1.0	0.5	1.7
Georgia	9.5	6.9	12.6	0.9	0.6	1.3
Germany	0.1	0.1	0.2	0.0	0.0	0.0
Ghana	3.0	0.9	7.7	2.2	0.7	5.5
Greece	0.1	0.1	0.2	0.0	0.0	0.1
Greenland	0.5	0.4	0.6	0.2	0.1	0.2
Grenada	0.0	0.0	0.2	0.0	0.0	0.1
Guam	0.8	0.0	4.0	0.1	0.0	0.5
Guatemala	0.5	0.3	1.0	0.2	0.1	0.4
Guinea	3.1	0.9	8.0	4.2	1.2	10.6
Guinea-Bissau	3.8	1.0	9.7	5.8	1.5	14.1
Guyana	0.3	0.0	1.2	0.2	0.0	0.7
Haiti	0.5	0.0	1.9	0.3	0.0	1.1
Honduras	0.7	0.4	1.3	0.4	0.2	0.8
Hungary	0.2	0.0	0.6	0.0	0.0	0.1
Iceland	0.0	0.0	0.0	0.0	0.0	0.0
India	9.1	8.3	10.0	4.8	3.9	5.7
Indonesia	1.2	0.7	2.5	0.7	0.3	1.4
Iran	0.2	0.1	0.4	0.1	0.0	0.1
Iraq	0.9	0.2	2.2	0.3	0.1	0.8
Ireland	0.1	0.0	0.1	0.0	0.0	0.0

Location	Incidence	Lower bound for incidence	Upper bound for incidence	Mortality	Lower bound for mortality	Upper bound for mortality
Israel	0.2	0.1	0.3	0.0	0.0	0.0
Italy	0.1	0.1	0.2	0.0	0.0	0.0
Jamaica	0.1	0.0	0.5	0.0	0.0	0.1
Japan	0.1	0.1	0.1	0.0	0.0	0.0
Jordan	0.5	0.1	1.6	0.1	0.0	0.3
Kazakhstan	16.9	12.5	22.0	1.9	1.4	2.5
Kenya	2.0	1.5	2.7	1.4	1.0	2.0
Kiribati	3.9	0.0	28.6	1.9	0.0	14.0
Kuwait	0.4	0.1	0.7	0.1	0.0	0.1
Kyrgyzstan	28.9	12.5	50.9	3.5	2.0	5.0
Laos	0.7	0.1	2.8	0.5	0.0	2.3
Latvia	2.3	1.7	3.0	0.3	0.2	0.4
Lebanon	0.3	0.0	1.2	0.1	0.0	0.2
Lesotho	23.9	13.0	40.4	23.4	13.1	38.4
Liberia	3.4	1.0	8.1	3.1	0.9	7.3
Libya	0.6	0.0	2.5	0.2	0.0	0.7
Lithuania	5.2	4.2	6.4	1.0	0.8	1.3
Luxembourg	0.0	0.0	0.0	0.0	0.0	0.0
Macedonia	0.2	0.1	0.5	0.0	0.0	0.1
Madagascar	1.3	0.4	3.2	0.6	0.2	1.4
Malawi	1.6	0.6	3.3	2.0	0.7	4.1
Malaysia	0.4	0.2	0.8	0.1	0.1	0.3
Maldives	0.3	0.0	1.0	0.1	0.0	0.2
Mali	1.1	0.4	2.9	1.5	0.4	3.7
Malta	0.0	0.0	0.0	0.0	0.0	0.0
Marshall Islands	3.6	0.3	14.0	0.5	0.0	1.8
Mauritania	1.5	0.4	3.5	0.9	0.2	2.2
Mauritius	0.1	0.0	0.2	0.0	0.0	0.0
Mexico	0.4	0.3	0.5	0.1	0.1	0.2

Location	Incidence	Lower bound for incidence	Upper bound for incidence	Mortality	Lower bound for mortality	Upper bound for mortality
Moldova	17.5	12.6	23.1	1.9	1.4	2.6
Mongolia	5.6	1.1	16.1	0.8	0.2	2.1
Montenegro	0.0	0.0	0.1	0.0	0.0	0.0
Morocco	2.4	0.8	5.6	1.7	0.5	4.5
Mozambique	8.4	2.8	19.5	11.9	4.2	25.1
Myanmar	8.2	3.1	18.1	4.1	1.8	7.9
Namibia	21.7	12.6	35.0	7.2	3.8	13.2
Nepal	3.6	2.7	4.5	1.7	1.0	3.1
Netherlands	0.1	0.0	0.1	0.0	0.0	0.0
New Zealand	0.1	0.0	0.2	0.0	0.0	0.0
Nicaragua	0.4	0.2	0.8	0.1	0.1	0.2
Niger	2.4	0.7	6.2	3.4	1.0	8.6
Nigeria	6.3	2.7	12.8	4.0	1.7	7.9
North Korea	6.1	3.6	9.3	0.6	0.3	0.9
Northern Mariana Islands	1.3	0.1	6.4	0.2	0.0	0.8
Norway	0.1	0.1	0.2	0.0	0.0	0.0
Oman	0.2	0.1	0.5	0.0	0.0	0.1
Pakistan	8.6	6.3	11.5	4.3	3.0	6.0
Palestine	0.1	0.0	0.3	0.0	0.0	0.0
Panama	0.5	0.2	0.8	0.2	0.1	0.3
Papua New Guinea	5.5	0.9	17.5	1.0	0.2	2.8
Paraguay	0.5	0.4	0.7	0.2	0.1	0.2
Peru	2.8	2.2	3.5	0.7	0.5	1.0
Philippines	7.2	2.3	17.7	3.2	1.0	7.5
Poland	0.1	0.0	0.1	0.0	0.0	0.0
Portugal	0.2	0.1	0.3	0.0	0.0	0.0
Puerto Rico	0.0	0.0	0.1	0.0	0.0	0.0
Qatar	0.5	0.1	1.3	0.0	0.0	0.1
Romania	1.5	0.6	2.9	0.2	0.1	0.4

Location	Incidence	Lower bound for incidence	Upper bound for incidence	Mortality	Lower bound for mortality	Upper bound for mortality
Russian Federation	8.0	5.6	10.9	1.8	1.1	2.6
Rwanda	3.7	1.9	6.5	3.8	2.0	6.6
Saint Lucia	0.1	0.0	0.5	0.0	0.0	0.1
Saint Vincent and the Grenadines	0.1	0.0	0.5	0.0	0.0	0.2
Samoa	0.1	0.0	0.3	0.0	0.0	0.1
Sao Tome and Principe	1.7	0.5	4.2	0.8	0.2	2.0
Saudi Arabia	1.1	0.4	2.6	0.5	0.2	1.0
Senegal	1.7	0.7	3.4	2.0	0.8	3.7
Serbia	0.1	0.0	0.3	0.0	0.0	0.0
Seychelles	0.0	0.0	0.1	0.0	0.0	0.0
Sierra Leone	3.1	0.8	7.7	3.0	0.8	7.2
Singapore	0.2	0.1	0.3	0.0	0.0	0.0
Slovakia	0.0	0.0	0.2	0.0	0.0	0.0
Slovenia	0.0	0.0	0.0	0.0	0.0	0.0
Solomon Islands	1.1	0.0	8.7	0.7	0.0	5.1
Somalia	21.5	9.4	40.2	30.4	13.5	55.6
South Africa	8.8	4.8	15.3	3.0	1.8	4.9
South Korea	1.0	0.6	1.6	0.2	0.1	0.3
South Sudan	4.2	1.3	11.0	6.7	2.1	16.4
Spain	0.1	0.0	0.1	0.0	0.0	0.0
Sri Lanka	0.1	0.0	0.4	0.0	0.0	0.1
Sudan	2.2	0.2	9.6	1.3	0.1	5.5
Suriname	0.1	0.0	0.5	0.0	0.0	0.1
Swaziland	40.0	14.3	88.0	20.0	7.4	40.8
Sweden	0.2	0.1	0.3	0.0	0.0	0.0
Switzerland	0.1	0.1	0.2	0.0	0.0	0.0
Syria	1.5	0.2	5.6	0.1	0.0	0.2
Taiwan	0.3	0.2	0.5	0.1	0.1	0.2
Tajikistan	8.0	4.6	12.5	1.2	0.8	1.9

Location	Incidence	Lower bound for incidence	Upper bound for incidence	Mortality	Lower bound for mortality	Upper bound for mortality
Tanzania	2.4	0.7	5.6	2.2	0.7	5.3
Thailand	1.8	0.6	4.4	0.6	0.2	1.3
The Bahamas	0.4	0.1	1.3	0.1	0.0	0.3
The Gambia	2.3	0.7	5.9	2.9	0.8	7.4
Timor-Leste	0.6	0.1	2.5	0.3	0.0	1.6
Togo	2.9	0.8	7.5	3.0	0.8	7.4
Tonga	0.3	0.0	2.1	0.0	0.0	0.4
Trinidad and Tobago	0.1	0.0	0.3	0.0	0.0	0.1
Tunisia	0.2	0.1	0.5	0.1	0.0	0.2
Turkey	0.7	0.3	1.3	0.1	0.0	0.2
Turkmenistan	21.2	10.6	35.5	2.4	1.3	3.6
Uganda	5.2	2.2	11.1	7.6	3.4	14.5
Ukraine	10.8	7.3	15.2	1.9	1.2	2.7
United Arab Emirates	0.3	0.0	1.2	0.1	0.0	0.4
United Kingdom	0.1	0.1	0.1	0.0	0.0	0.0
United States	0.0	0.0	0.0	0.0	0.0	0.0
Uruguay	0.0	0.0	0.1	0.0	0.0	0.0
Uzbekistan	16.0	6.6	28.1	3.2	1.7	4.7
Vanuatu	0.1	0.0	0.9	0.1	0.0	0.5
Venezuela	0.3	0.1	0.5	0.1	0.0	0.2
Vietnam	4.5	1.4	10.3	2.0	0.6	4.4
Virgin Islands, U.S.	0.1	0.0	0.3	0.0	0.0	0.0
Yemen	1.4	0.3	3.9	1.0	0.2	2.9
Zambia	5.2	1.9	12.2	6.1	2.2	14.4
Zimbabwe	12.3	2.7	34.2	9.4	2.2	24.3

References

1. Bangalore National Tuberculosis Institute. Tuberculosis in a rural population of South India: a five-year epidemiological study. *Bull WHO* 1974; **51**(5): 473.
2. Vos T, Abajobir AA, Abate KH, et al. Global, regional, and national incidence, prevalence, and years lived with disability for 328 diseases and injuries for 195 countries, 1990-2016: a systematic analysis for the Global Burden of Disease Study 2016. *Lancet* 2017; **390**(10100): 1211-59.
3. Gakidou E, Afshin A, Abajobir AA, et al. Global, regional, and national comparative risk assessment of 84 behavioural, environmental and occupational, and metabolic risks or clusters of risks, 1990-2016: a systematic analysis for the Global Burden of Disease Study 2016. *Lancet* 2017; **390**(10100): 1345-422.
4. Mesfin YM, Hailemariam D, Biadgilign S, Kibret KT. Association between HIV/AIDS and multi-drug resistance tuberculosis: a systematic review and meta-analysis. *PLoS One* 2014; **9**(1): e82235.
5. Centers for Disease Control and Prevention (CDC). Extensively drug-resistant tuberculosis--United States, 1993-2006. *MMWR Morb Mortal Wkly Rep* 2007; **56**(11): 250-3.
6. Naghavi M, Abajobir AA, Abbafati C, et al. Global, regional, and national age-sex specific mortality for 264 causes of death, 1980-2016: a systematic analysis for the Global Burden of Disease Study 2016. *Lancet* 2017; **390**(10100): 1151-210.
7. Foreman KJ, Lozano R, Lopez AD, Murray CJL. Modeling causes of death: an integrated approach using CODEm: University of Washington; 2011.
8. Cox JA, Lukande RL, Lucas S, Nelson AM, Van Marck E, Colebunders R. Autopsy causes of death in HIV-positive individuals in sub-Saharan Africa and correlation with clinical diagnoses. *AIDS Rev* 2010; **12**(4): 183-94.
9. Ford N, Matteelli A, Shubber Z, et al. TB as a cause of hospitalization and in-hospital mortality among people living with HIV worldwide: a systematic review and meta-analysis. *J Int AIDS Soc* 2016; **19**(1).
